# Ni-Catalyzed Oxygen Transfer from N_2_O onto sp^3^-Hybridized Carbons

**DOI:** 10.1021/jacs.2c06227

**Published:** 2022-09-26

**Authors:** Shengyang Ni, Franck Le Vaillant, Ana Mateos-Calbet, Ruben Martin, Josep Cornella

**Affiliations:** † Max-Planck-Institut für Kohlenforschung, Kaiser-Wilhelm-Platz 1, 45470Mülheim an der Ruhr, Germany; ‡ Institute of Chemical Research of Catalonia (ICIQ), The Barcelona Institute of Science and Technology, Av. Països Catalans 16, 43007Tarragona, Spain; § ICREA, Passeig Lluís Companys 23, 08010Barcelona, Spain

## Abstract

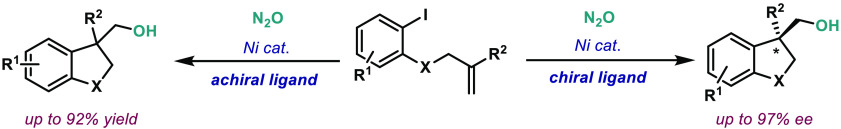

Herein we disclose a catalytic synthesis of cycloalkanols that harnesses the potential of N_2_O as an oxygen transfer agent onto sp^3^-hybridized carbons. The protocol is distinguished by its mild conditions and wide substrate scope, thus offering an opportunity to access carbocyclic compounds from simple precursors even in an enantioselective manner. Preliminary mechanistic studies suggest that the oxygen insertion event occurs at an alkylnickel species and that N_2_O is the O transfer reagent.

Nitrous oxide (N_2_O) is a gaseous molecule that contributes dramatically to global warming together with CO_2_ and CH_4_.^1^ Its large global warming potential (>300 times that of CO_2_) and long half-life in the atmosphere (ca. 100 years) have resulted in warnings against the anthropogenic emission of this gas, which has increased steeply in recent decades.^2,3^ However, from the synthetic point of view, nitrous oxide presents itself as an excellent O atom transfer (OAT) reagent: it is a potent O atom donor that releases benign N_2_, and it is relatively nontoxic to humans (laughing gas).^3^ However, N_2_O is inert,^4a^ and its poor σ-donor and π-acceptor abilities limit its activation by transition metals (Figure 1A).^4^ Therefore, forging synthetically relevant C–O bonds via homogeneous catalysis has been challenging^5,6^ and has largely relied on classical metal–oxo reactivity (epoxidations, C–H abstraction, etc.).^7^ Pioneering work by Hillhouse with transition metals and N_2_O demonstrated that certain L_2_Ni(II)–dialkyl complexes undergo O atom insertion into the Ni–C bond (Figure 1B).^6a^ Mechanistic studies on the (bipy)Ni(II)–dialkyl system performed by Hillhouse^6b^ and Cundari and Gunnoe^6e,6f^ suggested an organometallic Baeyer–Villiger-type mechanism for the O insertion step. Capitalizing on this reactivity, our group has recently disclosed the catalytic synthesis of phenols from (hetero)aryl halides using N_2_O under reductive conditions (Figure 1C, left).^8^ In this Communication, we demonstrate that this strategy can be extended to the catalytic synthesis of challenging C(sp^3^)–O bonds (Figure 1D). The protocol developed herein forges an additional C–C bond via a carbometalation event,^9,10^ which sets the stage for O insertion. Due to the resulting chiral quaternary center, we exploited a chiral bidentate ligand in the catalytic system to access enantioenriched indanols and benzofuran compounds, which are widespread motifs present in biologically relevant compounds.^11^

**Figure 1:**
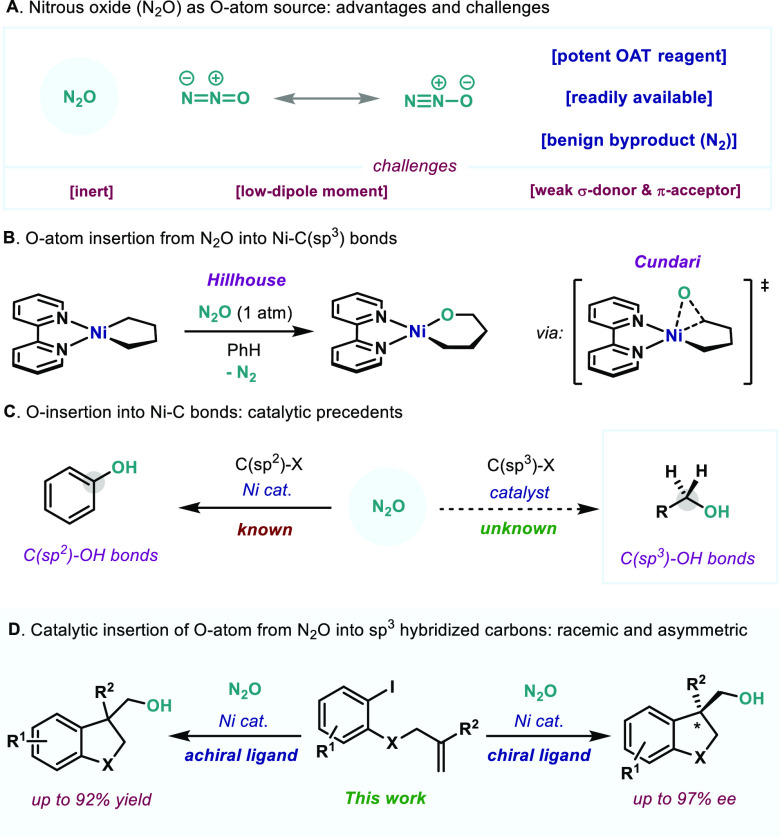
(A) Advantages and challenges of nitrous oxide. (B) Inspiration: Hillhouse’s work. (C) Catalytic formation of C(sp^2^)–OH vs C(sp^3^)–OH bonds. (D) Racemic and enantioselective Ni-catalyzed formation of primary alcohols through OAT from N_2_O.

Inspired by similar precedents on carbocyclization and C–C and C–N bond formation,^10^ we selected aryl iodide compound **1a** as the model substrate (Table 1). The use of 10 mol % NiI_2_ in combination with 15 mol % phenanthroline derivative **L1**, activated Zn, and NaI in DMSO at 25 °C resulted in an 87% isolated yield of alkanol **2a** (see the Supporting Information for full details of the reaction optimization).^12^ Bidentate bipyridine/phenanthroline derivatives as ligands were pivotal, but steric encumbrance in the form a Ph group α to the nitrogen is required to observe the desired reactivity (entries 2 and 3). Other bidentate ligands based on a pyridine–pyrazole scaffold did not lead to any conversion to **2a** (entry 4). Interestingly, ligand **L5** that previously proved crucial in the catalytic O insertion into C(sp^2^) bonds^8^ did not lead to product formation (entry 5). Lowering the pressure of N_2_O to 1.5 atm diminished the yield by *ca.* 10% (entry 6), and the substitution of Zn for Mn inhibited the reactivity (entry 7). Whereas the absence of NaI or Zn dramatically reduced the yield of **2a** (entry 8 and 9), replacement of the Ni(II) source did not influence the overall yield (entry 10). Finally, the reaction does not proceed in the absence of N_2_O (entry 11) or when the iodide in **1a** is replaced by bromide (entry 12). Whereas in the former case dimerization of **1a** was observed, recovery of the starting material is the main outcome in the latter scenario.

**Table 1:**
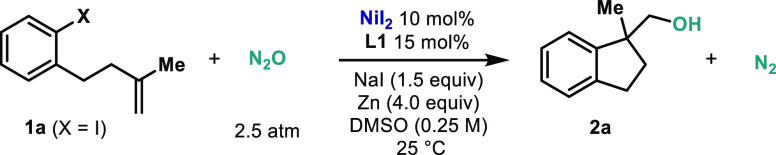

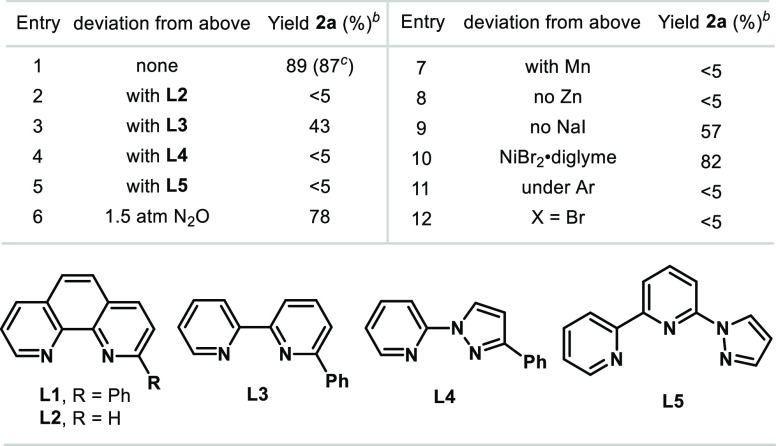
Optimization of the Ni-Catalyzed O Insertion onto C(sp^3^) Bonds^a^

a Reactions were performed with 0.1 mmol of **1a**.

b Yields were determined by ^1^H NMR spectroscopy using 1,3,5-trimethoxybenzene as an internal standard.

c Isolated yield; the reaction was performed with 0.1 mmol of **1a**.

The exploration of the scope of the catalytic protocol was performed with compounds similar to **1a**, where variations in both the aryl ring and the pendent double bond were introduced (Table 2). For example, the presence of alkyl groups in the aromatic ring did not impact the yield (**2b** and **2c**). Furthermore, benzyl alcohols protected in the form of acetal (**2d**), ether (**2e**), silyl ether (**2i**), or ester (**2f**) were well-tolerated. In the last example, lower yields were obtained, presumably due to the nucleophilic nature of the final alkoxide. Phenolic derivatives of catechol or anisole delivered alkanols **2g** and **2h** in good yields. Interestingly, the presence of halides directly attached to the aromatic moiety was also tolerated, as exemplified by the formation of **2j**–**2l** in good yields. The methyl group in the 1,1-disubstituted alkene could also be replaced, as exemplified by the compatibility of extended alkyl chains (**2m**–**2q**). Protected alcohols present in the alkyl chain of the alkene were also tolerated, as shown by acetal (**2r**) and the presence of Bn (**2s**) and Bz (**2t**). An extended aromatic substrate also reacted without apparent solubility issues (**2u**). Interestingly, a pendent alkene on the ring was also well-tolerated, without traces of potential epoxidation observed (**2v**). Finally, the linker between the aryl group and the alkene could be replaced by an O atom, leading to dihydrobenzofuranols (**2w**–**2ae**). In general, slightly lower yields were obtained compared to indanols. Nevertheless, similar substitution patterns in the aryl group and the pendent alkene were well-accommodated. Not surprisingly, when a substituent is placed *ortho* to the C–I bond, the corresponding alkanol cannot be obtained (**2af**). Unfortunately, attempts to forge six-membered rings were unsuccessful. It is also important to mention that the alkoxide generated as a product is incompatible with some functionalities, thus restricting orthogonal compatibility.

**Table 2:**
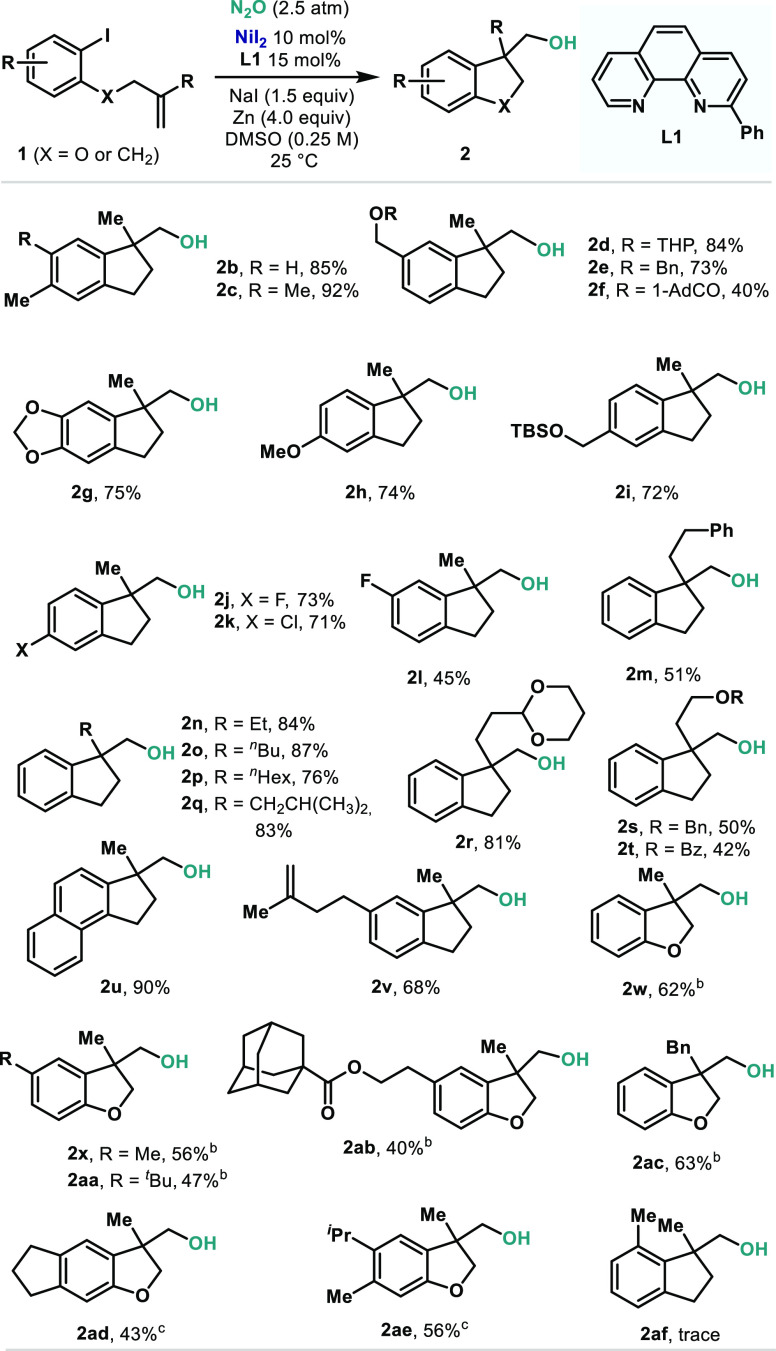
Scope of the Ni-Catalyzed Oxygen Transfer from N_2_O onto sp^3^-Hybridized Carbons^a^

a Reaction conditions: **1** (0.1 mmol), NiI_2_ (10 mol %), **L1** (15 mol %), NaI (0.15 mmol), and Zn (0.4 mmol) in DMSO (0.5 mL) at 25 °C for 40 h. Yields of isolated pure materials after preparative TLC are shown. Abbreviations: THP = 2-tetrahydropyranyl; TBS = *tert*-butyldimethylsilyl.

b DMA was used instead of DMSO.

c **L3** was used instead of **L1**.

The reaction design to obtain alkanols from **1** relies on an initial oxidative addition of the aryl iodide to the Ni catalyst, followed by a carbometalation event into the alkene. At this point, a quaternary stereogenic carbon is generated. Therefore, we envisaged that with an appropriate chiral ligand, a stereoselective carbometalation could lead to an enantioselective protocol. Re-evaluation of the reaction parameters when using chiral ligands was required, with the most noticeable modification being the replacement of DMSO with DMA. After a thorough screening, **L6** proved to be optimal to obtain high yields and high enantioselectivity (Table 3, entry 1).^10g^**L6** features an imidazolylpyridine backbone with a chiral carbon bearing a ^*t*^Bu at the 4-position of the imidazoline. N-Arylation of the ligand with a PMP group led to substantially lower levels of enantiocontrol (entry 2). When the imidazoline was replaced by oxazoline, lower yields and selectivities were obtained (entries 3–5). Finally, the addition of steric encumbrance at the *ortho* position of the pyridine moiety inhibited the reactivity (entry 6). Having identified **L6** as the optimal ligand, an exploratory scope was performed. A total of nine compounds were prepared with variations on the aryl group with alkyl groups (**2w**, **2x**, **2aa**, **2ae**, **2ad**) and an ester (**2ab**). Contrarily to the racemic protocol, dihydrofuranols proved to be more efficient in the asymmetric version than the corresponding indanols (**2a** and **2n**). The absolute configuration of the final protocol was determined based on the X-ray structure obtained for benzoylated (*S*)-**2w** and is in agreement with previous reports using chiral pyridine–oxazoline ligands.^10^

**Table 3:**
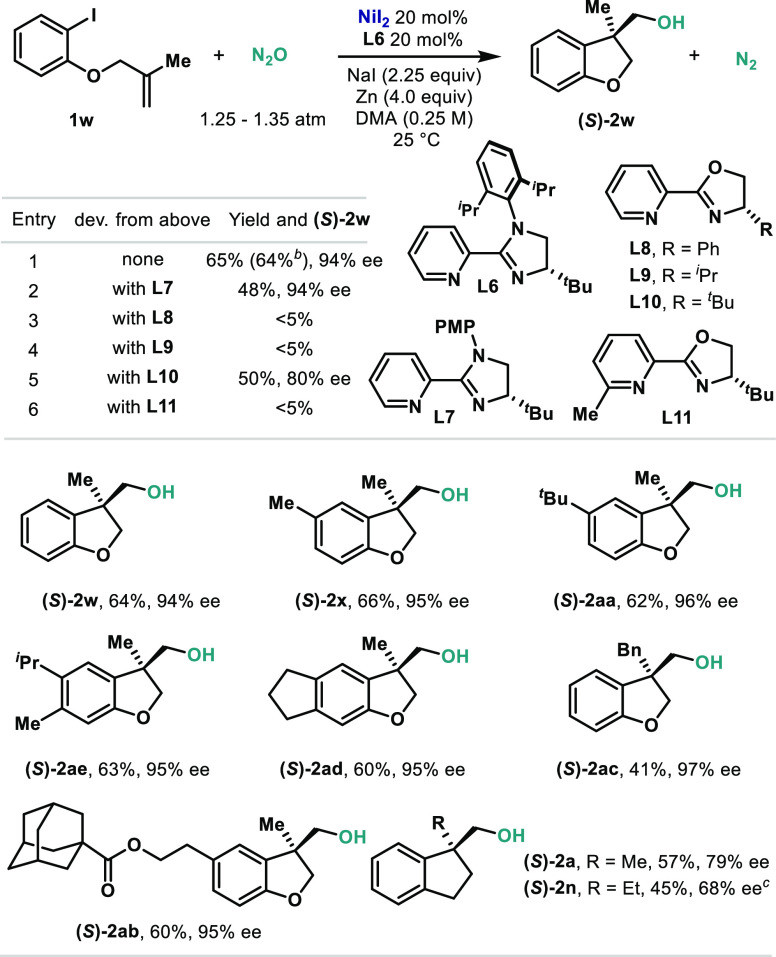
Enantioselective Carbohydroxylation of Olefins^a^

a Reactions were performed on a 0.1 mmol scale. Yields were determined by ^1^H NMR spectroscopy using 1,3,5-trimethoxybenzene as an internal standard. PMP = 4-methoxyphenyl.

b Isolated yield.

c **L10** was used instead of **L6**.

Having demonstrated the viability of forging C(sp^3^)–O bonds from N_2_O in a carbohydroxylation reaction, we conducted an investigation to gain insight into the potential intermediates as well as the origin of the O atom. As aforementioned, the reaction did not proceed under an Ar atmosphere (Table 1, entry 11). Analysis of the headspaces of three distinct reactions (**2a**, **2o**, and **2g**) clearly confirmed the presence of N_2_ (Scheme 1A). Control experiments did not indicate the presence of N_2_ in the employed N_2_O. Since the solvent in the racemic version was DMSO (Table 2), it was essential to explore the possibility of OAT from the solvent. To this end, ^18^O-labeled DMSO (25% ^18^O) was synthesized and subjected to the optimized reaction conditions. After completion of the reaction, no incorporation of ^18^O into **2a** was observed (Scheme 1B, left). More direct evidence on the origin of the O atom was obtained when ^15^N^15^N^18^O was used.^13^ In this case, compound **2a** was obtained with 46 ± 1% incorporation of ^18^O, which corresponds to the theoretical maximum (see the Supporting Information). Additionally, a series of potent O transfer reagents commonly employed in organic synthesis were tested under the optimized conditions. However, no reactivity toward the formation of **2a** was observed in any case (Scheme 1, bottom). This is in agreement with Cundari’s observations, where other sources of O were unable to engage with Ni complexes in OAT reactivity.^6e^ These experiments collectively point toward N_2_O as the O source. As mentioned previously, upon oxidative addition into the Ar–I bond, a carbometalation step ensues, thus delivering a primary alkylnickel without β hydrogens. We speculated that a similar alkylnickel intermediate would form starting from alkyl iodide **3a** (Scheme 1C).^14^ Indeed, when **3a** was subjected to the optimized reaction conditions, alcohol **4a** formed smoothly both in DMSO and DMA. In the absence of N_2_O, **3a** delivered a 73% yield of the corresponding protodeiodinated product and a 14% yield of the dimer. This clearly indicates that a similar alkylnickel intermediate is formed in both cases. Whereas the exact mechanism for the O insertion still remains elusive, direct addition of a free carbon radical to N_2_O is unlikely.^15^

**Scheme 1:**
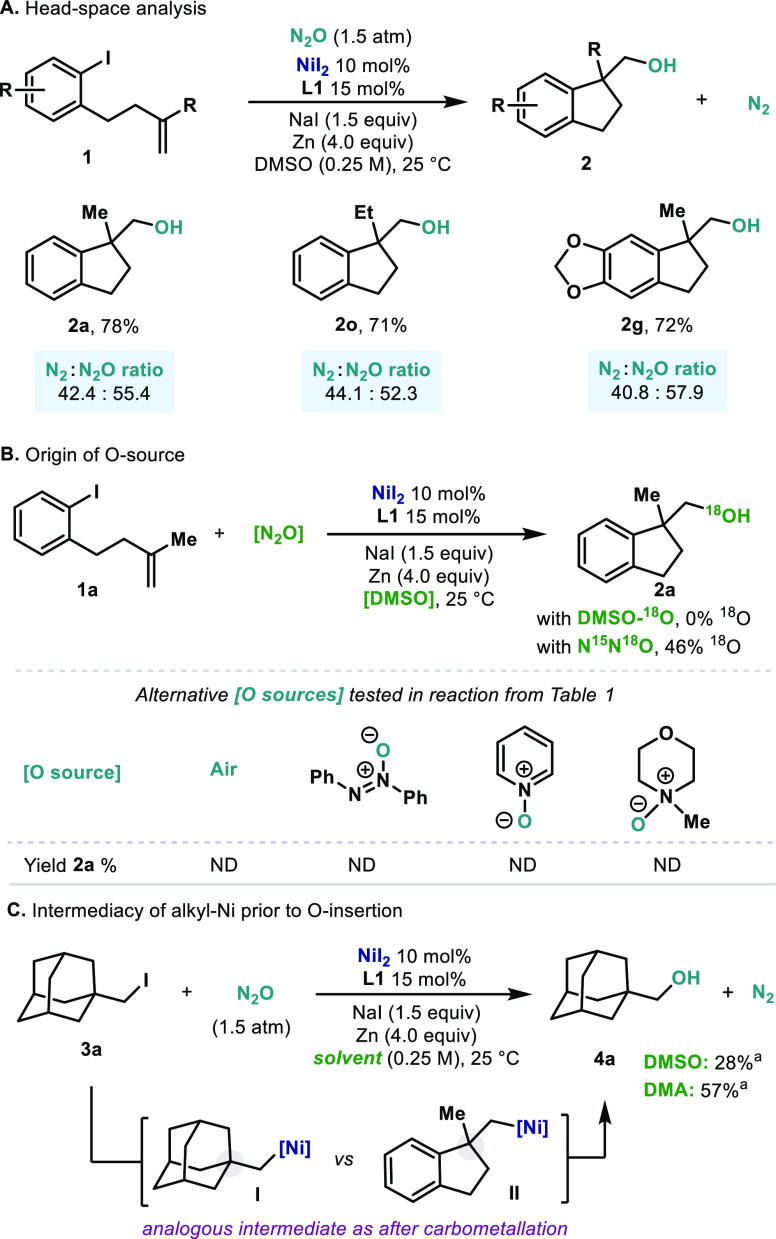
(A) Analysis of the Gaseous Headspace of the Reaction Mixture Confirms the Formation of N_2_; (B) Experiments Performed to Elucidate the Origin of the O Atom in the Final Product; (C) Involvement of an Alkylnickel Species in the Alkoxylation Step Yields were determined by ^1^H NMR spectroscopy using 1,3,5-trimethoxybenzene as an internal standard. ND = not detected.

In summary, we provide a protocol that unlocks catalytic O atom transfer from N_2_O for the formation of C(sp^3^)–O bonds under mild conditions. The protocol uses a combination of Ni and 2-phenylphenanthroline as a catalytic system in the presence of Zn and NaI as crucial reagents. The reaction engages aryl iodides bearing pendent alkenes, which upon carbometalation lead to O atom insertion into a Ni–C(sp^3^) bond. Since the carbohydroxylation method forges a quaternary stereocenter, an enantioselective protocol was also provided. For this purpose, a ligand based on an imidazolylpyridine backbone was utilized, which delivered the chiral alkanols in good yields with excellent enantioselectivities. A series of direct and indirect experiments confirmed the origin of the O atom and the uniqueness of N_2_O as the OAT reagent. These protocols add to the recent work on C(sp^2^)–O bond formation and consolidate the catalytic insertion of O atoms into M–C bonds using N_2_O as a valid strategy for the construction of oxygenated molecules in organic synthesis. Further applications of this concept are currently ongoing in our laboratory.

## Supporting Information Available

Experimental procedures and analytical data (^1^H, ^13^C, and ^19^F NMR, HRMS) for new compounds (PDF)
