# Supplementary material for: Ni-Catalyzed Oxygen Transfer from N2O onto sp3-Hybridized Carbons
Source: J Am Chem Soc. 2022 Sep 26;144(40):18223–8. doi: 10.1021/jacs.2c06227 (PMC9562464; doi:10.1021/jacs.2c06227)

Supporting Information

Ni-catalyzed Oxygen Transfer from N_2O into sp^3 Hybridized Carbons

Shengyang Ni,[♦] Franck Le Vaillant,[♦] Ana Mateos-Calbet,[♦] Ruben Martin[‡£] and Josep Cornella[♦]*

[♦]Max-Planck-Institut für Kohlenforschung, Kaiser-Wilhelm-Platz 1, Mülheim an der Ruhr, 45470, Germany

‡ Institute of Chemical Research of Catalonia (ICIQ), The Barcelona Institute of Science and Technology, Av. Països Catalans 16, 43007 Tarragona, Spain

£ ICREA, Passeig Lluís Companys, 23, 08010, Barcelona, Spain

Contents

1 General Methods 2

2 Handling of Activated Zinc 4

3 Preparation of substrates 5

4 Optimization table 16

5 Unsuccessful substrates 20

6 General Procedure for Ni-catalyzed oxygen transfer from N_2O into sp^3 hybridized carbons (*racemic*) (General Procedure A) 21

7 General Procedure for Ni-catalyzed oxygen transfer from N_2O into sp^3 hybridized carbons (*chiral*) (General Procedure B) 22

8 Graphical Procedure for Ni-catalyzed oxygen transfer from N_2O into sp^3 hybridized carbons (*racemic*) 23

9 Characterization Data 26

10 Reaction using ^{18}O-DMSO 48

11 Reaction using *in situ* formed ^{18}O-N_2O 50

12 Intermediacy of alkyl-Ni prior to O-insertion 53

13 Headspace analysis 54

14 HPLC Spectra 58

15 X-ray data 67

16 References 73

17 NMR Spectra 74

1 General Methods

Instruments

GC-MS (FID): GC-MS-QP2010 equipped (Shimadzu Europe Analytical Instruments). ESI-MS: ESQ 3000 (Bruker). Accurate mass determinations: Bruker APEX III FT-MS (7 T magnet) or MAT 95 (Finnigan). GC-TCD measurements were performed on Agilent Technologies GC 7890B with a 30 m HP-Plot 5 Å Molsieves column. Melting points were measured with an EZ-Melt Automated Melting Point Apparatus from Standford Research Systems. Specific rotations ([**α**]) were measured with a Rudolph RA Autopol IV Automatic Polarimeter at the indicated temperature with a sodium lamp (sodium D line, λ = 589 nm). Measurements were performed in an acid resistant 1 mL cell (50 mm length) with concentrations (g/(100 mL)) reported in the corresponding solvent. High performance liquid chromatography (HPLC) was performed on a Shimadzu LC-20AD liquid chromatograph SIL-20AC auto sampler, CMB-20A using Daicel columns with a chiral stationary phase. All solvents used were HPLC-grade solvents purchased from Sigma-Aldrich. The column employed and the respective solvent mixture are indicated for each experiment. NMR spectra were recorded using a Bruker AVIIIHD 300 MHz or Bruker AVneo 600 MHz NMR spectrometer. The chemical shifts (δ) are given in ppm and were measured relative to solvent residual peak as an internal standard. For ^{1}H NMR: $CDCl_3$, δ 7.26; $(CD)_3(SO)$, δ 2.50. For ^{13}C NMR: $CDCl_3$, δ 77.16; $(CD)_3(SO)$, δ 39.52. The data is being reported as (s = singlet, d = doublet, t = triplet, q = quartet, quint = quintet, m = multiplet or unresolved br s = broad signal, coupling constant(s) in Hz, integration, interpretation).

Chemicals

Unless otherwise stated, all manipulations were performed using Schlenk techniques under dry argon in heatgun-dried glassware. Unless otherwise noted, all reagents were obtained from commercial suppliers and used without further purification. N_2O was provided by Air Liquide (Distickstoffmonoxid UHP 5.0) containing less than 5 ppm N_2, 1 ppm H_2O, and 1 ppm air and O_2. Anhydrous DMA (250 mL, 99.8%) and NaI (anhydrous, free-flowing, Redi-Dri™, ReagentPlus®, ≥99%) were purchased from Sigma-Aldrich, stored directly in the glovebox, and use as received. Zinc powder (325 mesh, 99.9% (metal basis)) was purchased from Alfa Aesar, stored directly in

the glovebox, and used as received. Aryl iodide-tethered alkenes **1** were prepared according to procedures reported in the literature.[1] The ligand was synthesized according the literature.[2]

2 Handling of Activated Zinc

A round bottom flask was charged with 5 g of Zinc powder (325 mesh, 99.9% (metal basis)). 20 ml HCl (1 M) was added, and the mixture was stirred for 3 min at rt. Then, the mixture was filtered under reduced pressure using a vacuum filter funnel and washed with water (50 mL×3), acetone (50 mL×3), and ether (50 mL×3). After that, the solid was left to dry in the funnel for 3 min. The solid was transferred to a vial, dried under reduced pressure overnight, and stored in the glovebox.

3 Preparation of substrates

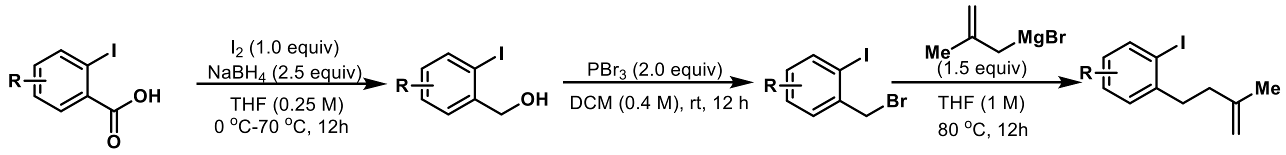

A 100 mL round-bottom flask was charged with benzoic acid (10 mmol) and dry THF (20 mL). $NaBH_4$ (2.5 equiv) was added, and the reaction mixture cooled in an ice bath. A solution of I_2 (1.0 equiv) in THF (20 mL) was added dropwise over 30 min with vigorous evolution of hydrogen. After heating to reflux for 12 h and then cooling to room temperature, MeOH was added until the solid completely dissolved. After stirring for 30 min, the solvent was removed by rotary evaporation, leaving a white paste dissolved by adding 20% aqueous KOH (20 mL). The solution was stirred for 4 h at room temperature and subsequently extracted with EtOAc. The combined organic extracts were washed with brine, dried over anhydrous Na_2SO_4, filtered, and concentrated under vacuum. The obtained benzyl alcohol was used in the next step without purification.

PBr_3 (80 mmol) was added to a solution of the corresponding alcohol (40 mmol) in dry CH_2Cl_2 (100 mL), and the reaction mixture was stirred at room temperature for 12 h. The solventvent was evaporated, and the resulting oil was treated with saturated $NaHCO_3$. The resulting aqueous phase was extracted with CH_2Cl_2. The combined organic extracts were washed with brine, dried over anhydrous Na_2SO_4, filtered, and concentrated under vacuum. The obtained benzyl bromide was used in the next step without purification.

In an oven-dried 500 mL round bottom flask under Argon, equipped with a magnetic stir bar and septum, 2-iodobenzyl bromide was dissolved in dry THF (10 mL). The solution was cooled in an ice bath (0 ^{o}C) and 2-methylallylmagnesium bromide (0.50 M in THF, 15 mmol) was added using a syringe. The stirring solution was then refluxed under argon for 2 h at which point TLC indicated that the reaction was complete. The reaction was quenched by the addition of sat. NH_4Cl solution (30 mL), extracted with MTBE (3 × 50 mL), the combined organic layers dried with Na_2SO_4 and concentrated under reduced pressure. The residue was purified by flash column chromatography on silica gel to give the product.

Compound 1c

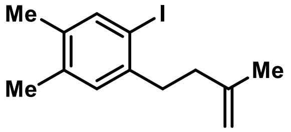

The reaction was conducted on 3.8 mmol scale. Purification by flash column chromatography (silica gel, hexanes) afforded 878 mg (overall yield: 77%) of the title compound **1c**.

Physical State: colorless oil.

R_f = 0.6 (hexanes).

^{1}H NMR (300 MHz, $CDCl_3$) δ 7.60 (s, 1H), 7.01 (s, 1H), 4.85 – 4.77 (m, 2H), 2.87 – 2.73 (m, 2H), 2.36 – 2.24 (m, 2H), 2.22 (s, 3H), 2.21 (s, 3H), 1.85 (s, 3H) ppm.

^{13}C NMR (75 MHz, $CDCl_3$) δ 145.3, 142.0, 140.1, 137.0, 136.5, 130.6, 110.4, 96.7, 39.0, 38.7, 22.7, 19.5, 18.9 ppm.

HRMS (EI-TOF): calc'd for $C_{13}H_{17}I_1$ $[M]^+$: 300.036948, found: 300.037000.

Compound 1k

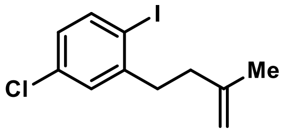

The reaction was conducted on 3.6 mmol scale. Purification by flash column chromatography (silica gel, hexanes) afforded 773.6 mg (overall yield: 70%) of the title compound **1k**.

Physical State: colorless oil.

R_f = 0.58 (hexanes).

^{1}H NMR (300 MHz, $CDCl_3$) δ 7.71 (d, J = 8.4 Hz, 1H), 7.20 (d, J = 2.5 Hz, 1H), 6.89 (dd, J = 8.4, 2.6 Hz, 1H), 4.77 (d, J = 10.4 Hz, 2H), 2.87 – 2.72 (m, 2H), 2.34 – 2.20 (m, 2H), 1.81 (s, 3H) ppm.

^{13}C NMR (75 MHz, $CDCl_3$) δ 146.6, 144.6, 140.5, 134.7, 129.4, 128.0, 111.0, 97.6, 39.4, 38.1, 22.7 ppm.

HRMS (ESI-TOF): calc'd for $C_{11}H_{13}I_1Cl_1$ $[M+H]^+$: 306.974501, found: 306.974480.

Compound 1l

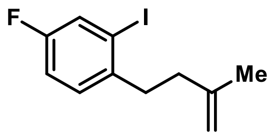

The reaction was conducted on 3.8 mmol scale. Purification by flash column chromatography (silica gel, hexanes) afforded 694.2 mg (overall yield: 63%) of the title compound **1l**.

Physical State: colorless oil.

R_f = 0.6 (hexanes).

^{1}H NMR (300 MHz, CDCl$_3$) δ 7.54 (dd, *J* = 8.1, 2.7 Hz, 1H), 7.16 (dd, *J* = 8.5, 5.9 Hz, 1H), 7.00 (td, *J* = 8.3, 2.7 Hz, 1H), 4.88 – 4.68 (m, 2H), 2.91 – 2.76 (m, 2H), 2.33 – 2.18 (m, 2H), 1.81 (s, 3H) ppm.
^{13}C NMR (75 MHz, CDCl$_3$) δ 160.5 (d, *J* = 249.3 Hz), 144.8, 140.6 (d, *J* = 3.5 Hz), 129.7 (d, *J* = 7.8 Hz), 126.2 (d, *J* = 23.4 Hz), 115.4 (d, *J* = 20.5 Hz), 110.8, 99.3 (d, *J* = 8.0 Hz), 38.5, 38.5 (d, *J* = 1.2 Hz), 22.7 ppm.
^{19}F NMR (282 MHz, CDCl$_3$) δ -115.88 ppm.
HRMS (EI-TOF): calc'd for $C_{11}H_{12}I_1F_1$ $[M]^+$: 289.996226, found: 289.996220.

Compound 1u

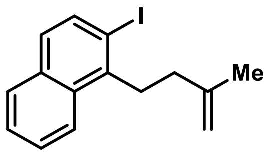

The reaction was conducted on 2.0 mmol scale. Purification by flash column chromatography (silica gel, hexanes) afforded 405.6 mg (overall yield: 63%) of the title compound **1u**.

Physical State: light yellow oil.

R$_f$ = 0.55 (hexanes).

^{1}H NMR (300 MHz, CDCl$_3$) δ 8.10 – 8.00 (m, 1H), 7.91 – 7.77 (m, 2H), 7.59 – 7.45 (m, 2H), 7.41 (d, *J* = 8.7 Hz, 1H), 5.00 – 4.82 (m, 2H), 3.50 – 3.32 (m, 2H), 2.43 – 2.24 (m, 2H), 1.92 (s, 3H) ppm.

^{13}C NMR (75 MHz, CDCl$_3$) δ 145.6, 141.1, 136.4, 133.6, 132.2, 128.9, 128.2, 127.0, 126.1, 124.5, 110.4, 99.4, 37.4, 36.8, 22.8 ppm.

HRMS (EI-TOF): calc'd for $C_{15}H_{15}I_1$ $[M]^+$: 322.021297, found: 322.021580.

Compound 1d

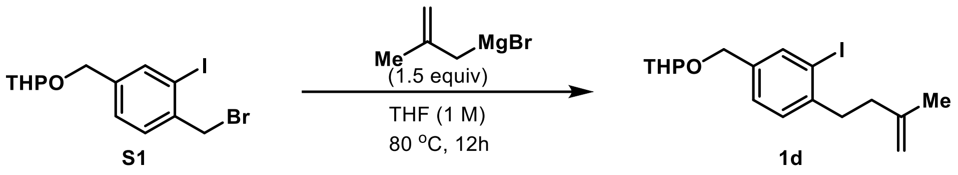

The reaction was conducted on 2.0 mmol scale. In an oven-dried 20 mL Schlenk tube under Argon, equipped with a magnetic stir bar and septum, 2-iodobenzyl bromide **S1** (2.0 mmol) was dissolved in dry THF (5 mL). The solution was cooled in an ice bath (0 °C) and 2-methylallylmagnesium bromide (0.50 M in THF, 7.5 mmol) was added using a syringe. The stirring solution was then refluxed under argon for 2 h at which point TLC indicated that the reaction was complete. The reaction was quenched by the addition of sat. NH_4Cl solution (30 mL), extracted with MTBE (3 × 50 mL), the combined organic layers dried with Na_2SO_4 and concentrated under reduced pressure. Purification by flash column chromatography (silica gel, hexanes) afforded 663.9 mg (86%) of the title compound **1d**.

Physical State: colorless oil.

R$_f$ = 0.52 (hexanes).

^{1}H NMR (300 MHz, CDCl$_3$) δ 7.81 (d, *J* = 1.7 Hz, 1H), 7.26 (dd, *J* = 7.7, 1.8 Hz, 1H), 7.17 (d, *J* = 7.8 Hz, 1H), 4.82 – 4.61 (m, 4H), 4.40 (d, *J* = 12.1 Hz, 1H), 3.97 – 3.83 (m, 1H), 3.60 – 3.49 (m, 1H), 2.88 – 2.77 (m, 2H), 2.35 – 2.20 (m, 2H), 1.94 – 1.47 (m, 9H) ppm.

^{13}C NMR (75 MHz, CDCl$_3$) δ 145.0, 143.9, 138.8, 138.1, 129.1, 127.9, 110.6, 100.5, 97.9, 67.7, 62.2, 39.2, 38.4, 30.6, 25.5, 22.7, 19.4 ppm.

HRMS (ESI-TOF): calc'd for $C_{17}H_{23}I_1O_2Na_1$ $[M+Na]^+$: 409.063496, found: 409.063620.

Compound 1v

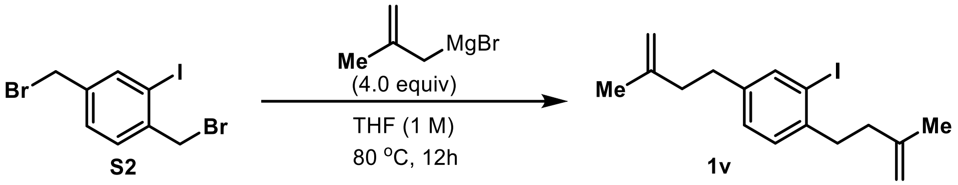

In an oven-dried 25 mL Schlenk tube under Argon, equipped with a magnetic stir bar and septum, benzyl bromide **S2** (1.0 mmol) was dissolved in dry THF (5 mL). The solution was cooled in an ice bath (0 °C) and 2-methylallylmagnesium bromide (0.50 M in THF, 4.0 mmol) was added using a syringe. The stirring solution was then refluxed under argon for 2 h at which point TLC indicated that the reaction was complete. The reaction was quenched by the addition of sat. NH_4Cl solution (30 mL), extracted with MTBE (3 × 50 mL), the combined organic layers dried with Na_2SO_4 and concentrated under reduced pressure. Purification by flash column chromatography (silica gel, hexanes) afforded 187.0 mg (55%) of the title compound **1v**.

Physical State: colorless oil.

R_f = 0.57 (hexanes).

^{1}H NMR (300 MHz, $CDCl_3$) δ 7.67 (s, 1H), 7.17 – 7.06 (m, 2H), 4.83 – 4.65 (m, 4H), 2.87 – 2.76 (m, 2H), 2.72 – 2.62 (m, 2H), 2.34 – 2.21 (m, 4H), 1.82 (s, 3H), 1.77 (s, 3H) ppm.
^{13}C NMR (75 MHz, $CDCl_3$) δ 145.2, 145.1, 142.1, 142.1, 139.3, 129.1, 128.5, 110.6, 110.5, 100.6, 39.5, 39.1, 38.5, 33.3, 22.7, 22.7 ppm.
HRMS (EI-TOF): calc'd for $C_{16}H_{21}I_1$ $[M]^+$: 340.068247, found: 340.068560.

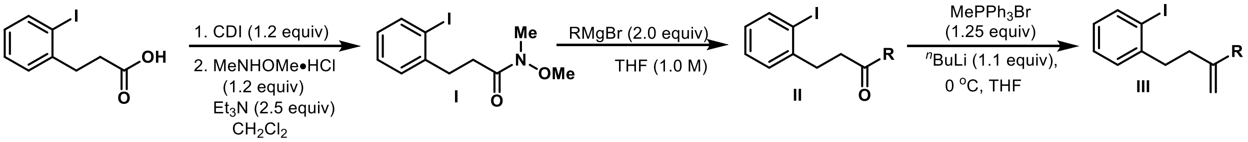

To a 250 mL round-bottomed flask equipped with a stir bar was added 3-(2-iodophenyl)propanoic acid (5 mmol, 1.0 equiv) and CH_2Cl_2 (60 mL). To this stirred solution, CDI (6 mmol, 1.2 equiv)

was added in one portion. The mixture was stirred for 1 h at room temperature. After that, N-O-dimethylhydroxylamine hydrochloride (6 mmol, 1.2 equiv) and Et_3N (12.5 mmol, 2.5 equiv) were added, and the reaction mixture was stirred overnight. After that, the reaction was quenched with 50 mL of 1M aq. HCl and stirred vigorously for 10 min. The aqueous layer was extracted with CH_2Cl_2 (3 ×15 mL). The combined organic layers were washed with HCl (1 M, 30 mL), H_2O (30 mL), sat. $NaHCO_3$ solution (2×30 mL), and brine (30 mL), dried over anhydrous Na_2SO_4, filtered and concentrated under vacuum. The residue was purified through column chromatography on silica gel (pentane : EtOAc = 10:1), affording the pure product **I**.

A solution of RMgBr (2 mmol, 2.0 equiv) in THF was added dropwise to a dry THF solution (1.0 M) of **I** (1 mmol, 1.0 equiv) under argon at 0 °C. The mixture was stirred at room temperature for 2 h, then quenched with water and the aqueous phase extracted with ethyl acetate. The combined organic layers were washed with brine, dried over anhydrous Na_2SO_4, filtered and concentrated to give the crude ketones **II**, which were used in the next step without further purification.

To a dry THF (15 mL) solution of $MePPh_3Br$ (1.25 mmol, 1.25 equiv), ^{n}BuLi (2.6 M in hexane, 1.1 mmol, 1.1 equiv) was added at 0°C. Afterwards, the mixture was stirred at room temperature for 30 min, a THF solution (1 M) of the crude ketones **II** (3.5mmol, 1.0 equiv) was added dropwise at 0°C. After stirring at room temperature overnight, the mixture was quenched with water and the aqueous layer was extracted with EtOAc. The combined organic phases were washed with brine, dried over anhydrous Na_2SO_4, filtered and concentrated in vacuum. The residue was purified by column chromatography on silica gel with pentane to give the corresponding final product.

Compound 1m

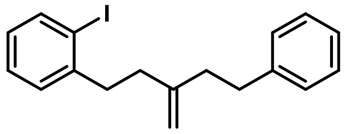

The reaction was conducted on 0.5 mmol scale. Purification by flash column chromatography (silica gel, hexanes) afforded 103.2 mg (overall yield: 57%) of the title compound **1m**.

Physical State: colorless oil.

R_f = 0.47 (hexanes).

^{1}H NMR (300 MHz, $CDCl_3$) δ 7.81 (dd, *J* = 7.9, 1.3 Hz, 1H), 7.33 – 7.14 (m, 7H), 6.93 – 6.82 (m, 1H), 4.84 (d, *J* = 2.8 Hz, 2H), 2.91 – 2.74 (m, 4H), 2.46 – 2.26 (m, 4H) ppm.

^{13}C NMR (75 MHz, $CDCl_3$) δ 148.4, 144.8, 142.3, 139.6, 129.5, 128.5, 128.5, 128.5, 127.9, 126.0, 110.2, 100.6, 39.8, 38.2, 36.9, 34.6 ppm.

HRMS (ESI-TOF): calc'd for $C_{18}H_{20}I_1$ $[M+H]^+$: 363.060422, found: 363.060690.

Compound 1o

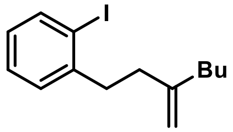

The reaction was conducted on 2.0 mmol scale. Purification by flash column chromatography (silica gel, hexanes) afforded 445.9 mg (overall yield: 71%) of the title compound **1o**.

Physical State: colorless oil.

R_f = 0.6 (hexanes).

^{1}H NMR (300 MHz, $CDCl_3$) δ 7.82 (dd, *J* = 7.9, 1.2 Hz, 1H), 7.31 – 7.18 (m, 2H), 6.88 (ddd, *J* = 7.9, 7.0, 2.1 Hz, 1H), 4.84 – 4.76 (m, 2H), 2.88 – 2.79 (m, 2H), 2.34 – 2.22 (m, 2H), 2.16 – 2.03 (m, 2H), 1.54 – 1.26 (m, 4H), 0.93 (t, *J* = 7.2 Hz, 3H) ppm.

^{13}C NMR (75 MHz, $CDCl_3$) δ 149.3, 145.0, 139.6, 129.5, 128.5, 127.8, 109.5, 100.6, 39.8, 36.7, 36.1, 30.2, 22.6, 14.2 ppm.

HRMS (ESI-TOF): calc'd for $C_{14}H_{20}I_1$ $[M+H]^+$: 315.060422, found: 315.060520.

Compound 1p

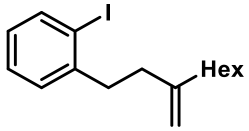

The reaction was conducted on 2.0 mmol scale. Purification by flash column chromatography (silica gel, hexanes) afforded 507.6 mg (overall yield: 74%) of the title compound **1p**.

Physical State: colorless oil.

R_f = 0.6 (hexanes).

^{1}H NMR (300 MHz, $CDCl_3$) δ 7.81 (dd, *J* = 7.9, 1.2 Hz, 1H), 7.31 – 7.18 (m, 2H), 6.96 – 6.82 (m, 1H), 4.79 (d, *J* = 2.6 Hz, 2H), 2.89 – 2.75 (m, 2H), 2.33 – 2.20 (m, 2H), 2.09 (t, *J* = 7.6 Hz, 2H), 1.52 – 1.41 (m, 2H), 1.37 – 1.23 (m, 6H), 0.95 – 0.80 (m, 3H) ppm.
^{13}C NMR (75 MHz, $CDCl_3$) δ 149.4, 145.0, 139.6, 129.5, 128.5, 127.8, 109.5, 100.6, 39.8, 36.7, 36.4, 31.9, 29.2, 28.0, 22.8, 14.3 ppm.
HRMS (ESI-TOF): calc'd for $C_{16}H_{24}I_1$ $[M+H]^+$: 343.091723, found: 343.091970.

Compound 1q

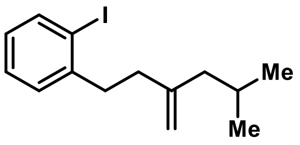

The reaction was conducted on 1.0 mmol scale. Purification by flash column chromatography (silica gel, hexanes) afforded 128.7 mg (overall yield: 41%) of the title compound **1q**.

Physical State: colorless oil.

R_f = 0.6 (hexanes).

^{1}H NMR (300 MHz, $CDCl_3$) δ 7.81 (dd, *J* = 7.9, 1.2 Hz, 1H), 7.34 – 7.16 (m, 2H), 6.94 – 6.80 (m, 1H), 4.88 – 4.68 (m, 2H), 2.86 – 2.76 (m, 2H), 2.31 – 2.15 (m, 2H), 1.97 (d, *J* = 7.2 Hz, 2H), 1.90 – 1.74 (m, 1H), 0.91 (s, 3H), 0.89 (s, 3H) ppm.
^{13}C NMR (75 MHz, $CDCl_3$) δ 148.0, 145.0, 139.6, 129.5, 128.5, 127.8, 110.9, 100.6, 46.3, 39.8, 36.4, 26.4, 22.7 ppm.
HRMS (ESI-TOF): calc'd for $C_{14}H_{20}I_1$ $[M+H]^+$: 315.060423, found: 315.060680.

Compound 1r

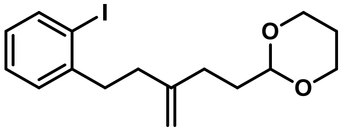

The reaction was conducted on 1.0 mmol scale. Purification by flash column chromatography (silica gel, hexanes) afforded 200.9 mg (overall yield: 54%) of the title compound **1r**.

Physical State: colorless oil.

R_f = 0.51 (hexanes).

^{1}H NMR (300 MHz, $CDCl_3$) δ 7.72 (dd, *J* = 7.9, 1.2 Hz, 1H), 7.24 – 7.06 (m, 2H), 6.86 – 6.71 (m, 1H), 4.77 – 4.68 (m, 2H), 4.47 (t, *J* = 5.1 Hz, 1H), 4.08 – 3.97 (m, 2H), 3.76 – 3.58 (m, 2H), 2.83 – 2.65 (m, 2H), 2.27 – 2.16 (m, 2H), 2.16 – 2.07 (m, 2H), 2.06 – 1.91 (m, 1H), 1.77 – 1.65 (m, 2H), 1.32 – 1.20 (m, 1H) ppm.
^{13}C NMR (75 MHz, $CDCl_3$) δ 148.1, 144.7, 139.5, 129.4, 128.4, 127.8, 109.8, 102.0, 100.6, 67.0, 39.6, 36.8, 33.5, 30.4, 26.0 ppm.
HRMS (ESI-TOF): calc'd for $C_{16}H_{21}I_1O_2Na_1$ $[M+Na]^+$: 395.047846, found: 395.047990.

Compound 1e

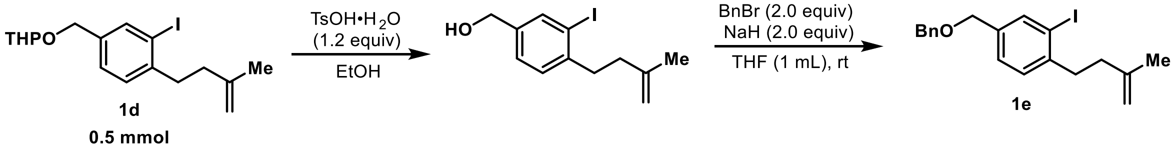

To a solution of **1d** (193 mg, 0.5 mmol) in 10 mL ethanol were added p-toluenesulfonic acid monohydrate (1.2 equiv) and the mixture was stirred for 2 hours at room temperautre. The reaction mixture was added to a saturated solution of $NaHCO_3$ and the resulting mixture extracted with dichloromethane. The combined organic layers were washed with brine and dried over anhydrous Na_2SO_4. After filtration and removal of the solvent, the crude product was used directly for the next step without further purification.

A Schlenk tube was charged with the crude mixture, and then evacuated and backfilled with argon (three times). After that, dry THF (1 mL) was added, and the mixture was stirred at 0 °C for 5 mins. NaH (1.0 mmol) was then added under Ar. Afterwards, the reaction was stirred for additional 20 min, benzyl bromide (1.0 mmol) was added using a Hamilton syringe. After stirring at room temperature overnight, the mixture was quenched with water and the aqueous layer extracted with EtOAc. The combined organic phases were washed with brine, dried over anhydrous Na_2SO_4, filtered and concentrated in vacuum. The residue was purified by column chromatography on silica gel with pentane as eluent to give 176.4 mg (overall yield: 90%) of the corresponding final product **1e**.

Physical State: colorless oil.

R_f = 0.53 (hexanes).

^{1}H NMR (300 MHz, $CDCl_3$) δ 7.73 (s, 1H), 7.30 – 7.04 (m, 7H), 4.71 – 4.62 (m, 2H), 4.45 (s, 2H), 4.36 (s, 2H), 2.86 – 2.60 (m, 2H), 2.27 – 2.11 (m, 2H), 1.72 (s, 3H) ppm.
^{13}C NMR (75 MHz, $CDCl_3$) δ 145.0, 144.0, 138.7, 138.1, 138.1, 129.2, 128.5, 127.9, 127.9, 127.8, 110.6, 100.5, 72.4, 70.9, 39.2, 38.4, 22.7 ppm.
HRMS (ESI-TOF): calc'd for $C_{19}H_{21}I_1O_1Na_1$ $[M+Na]^+$: 415.052931, found: 415.053020.

Compound 1f

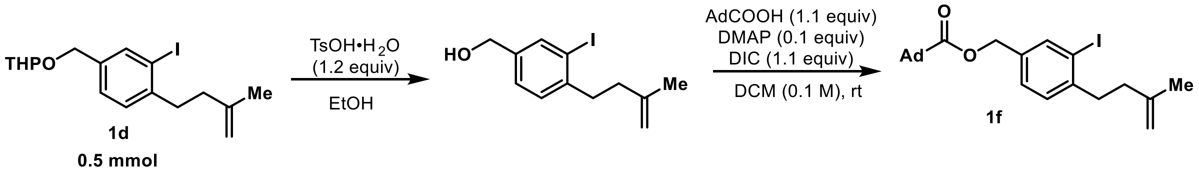

To a solution of **1d** (193 mg, 0.5 mmol**)** in 10 mL ethanol was added p-toluenesulfonic acid monohydrate (1.2 equiv), and the mixture stirred for 2 hours at room temperature. The reaction mixture was added to a saturated solution of $NaHCO_3$ and extracted with dichloromethane. The combined organic layers were washed with brine and dried over dried over anhydrous Na_2SO_4. After filtration and removal of the solvent, the crude product was used directly for the next step without further purification.

A culture tube was charged with the crude product. Dichloromethane was added (1 mL), and the mixture was stirred vigorously. DMAP (0.1 equiv) and $AdCO_2H$ (1.1 equiv) were added. DIC (1.1 equiv) was then added dropwise *via* syringe, and the mixture was stirred until the alcohol was consumed (determined by TLC). The mixture was then filtered (through a thin pad of Celite®, SiO_2, or frit funnel) and rinsed with additional CH_2Cl_2. The solvent was removed under reduced pressure, and purification of the crude mixture by column chromatography (pentane:EtOAc = 20:1) afforded 192.6 mg (overall yield: 83%) of the title compound **1f**.

Physical State: colorless oil.

R_f = 0.66 (20:1 hexanes:EtOAc).

^{1}H NMR (300 MHz, $CDCl_3$) δ 7.53 (d, *J* = 1.9 Hz, 1H), 7.06 – 6.89 (m, 2H), 4.76 (s, 2H), 4.52 (d, *J* = 5.6 Hz, 2H), 2.60 (dd, *J* = 9.5, 7.1 Hz, 2H), 2.03 (dd, *J* = 9.9, 6.6 Hz, 2H), 1.78 (s, 3H), 1.68 (s, 6H), 1.57 (s, 3H), 1.48 (s, 6H) ppm.
^{13}C NMR (75 MHz, $CDCl_3$) δ 177.5, 145.0, 144.5, 138.7, 136.3, 129.3, 127.9, 110.7, 100.4, 64.5, 40.9, 39.2, 38.9, 38.3, 36.6, 28.1, 22.7 ppm.
HRMS (ESI-TOF): calc'd for $C_{23}H_{29}I_1O_2Na_1$ $[M+Na]^+$: 487.110446, found: 487.110690.

4 Optimization table

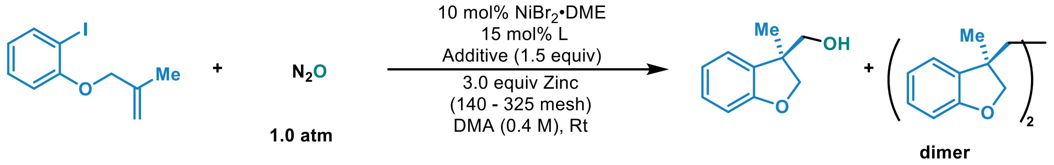

Nickel source	Ligand	Additive	N_2O	Reductant	Yield %	Yield of the dimer%
$NiBr_2$•DME	L1	NaI	1.5	Zn	10	30
$NiBr_2$•DME	L2	NaI	1.5	Zn	10	33
$NiBr_2$•DME	L2	NaI	2.0	Zn	11	36
$NiBr_2$•DME	L2	no NaI	1.5	Zn	6	messy
$NiBr_2$•DME 50 °C	L2	NaI	1.5	Zn	6	35
$Ni(acac)_2$	L2	NaI	1.5	Mn	ND	ND
$NiBr_2$•DME 20 mol%	L2	NaI	1.5	Zn	39	16

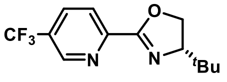

L1

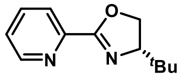

L2

Note: Yield was determined by crude ^1H NMR using 1,3,5-trimethoxybenzene as internal standard. In the initial optimization, the yield improvement was prioritized. Therefore, the ee was determined only for selected conditions. <u>If the yield of dimer is 20%, it means 40% of the starting material converted to the dimer during the reaction.</u>

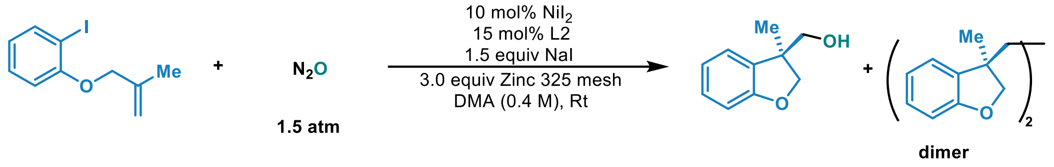

Entry	Deviation from standard conditions	Yield %	Yield of the dimer%
1	20 mol% NiI_2, 30 mol% L2	39	15
2	20 mol% NiI_2, 20 mol% L2	48 (80% ee)	16
3	20 mol% NiI_2, 22/25 mol% L2	39-45	12-17
4	DMF/DMA/DMSO/MeCN/THF/Dioxane	0-28	0-45
5	1.2/1.5/1.7/2.0 ATM N_2O	36-48	11-20
6	TMSCl, $MgCl_2$, $MgBr_2$, TBAI, TEAI, THAI, MgF, CoPc, LiBr, LiCl, CoI_2, $FeBr_2$, $CoBr_2$, $CoCl_2$	<45	0-22
7	Zinc (1.0, 1.5, 2.0, 2.5, 3.0, 3.5)	6-48	10-18
8	NaI (0.2, 0.5, 1.0, 2.0, 3.0)	27-48	27-48
9	Zinc nanopowder	48	19

Note: Different additives were added into the reaction system, but NaI seemed to be the best. In order to obtain a higher yield, further ligands were tested, please see below. <u>If the yield of dimer is 20%, it means 40% of the starting material converted to the dimer during the reaction.</u>

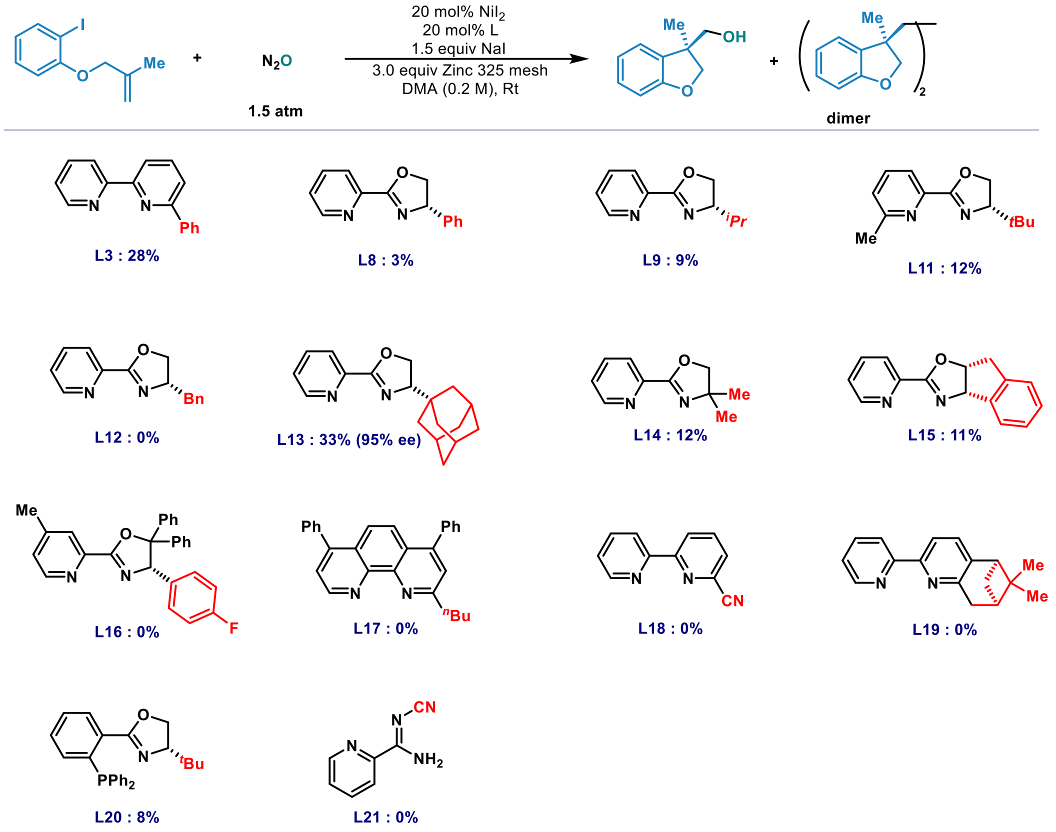

Note: In order to get a higher yield, different ligands were tested, the bpy type ligand with one arm could afford a higher yield (L3).

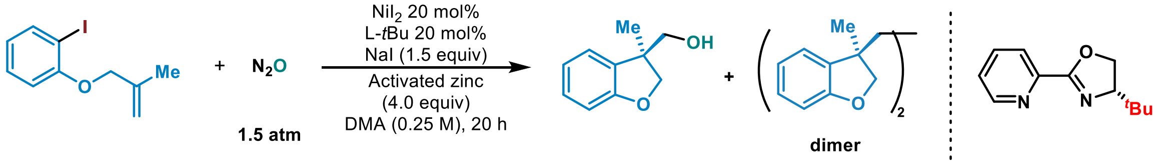

Entry	Deviation from standard conditions	Yield %	Yield of the dimer%
1	0.25, 0.3, 0.5, 0.7 mL, 1.0 mL DMA	37-46	12-19
2	2.0 atm N_2O	52	18
3	2.5 atm N_2O	44	22
4	TBAI or CsI	39, 49	12, 11
5	Zinc nanopowder	54	11
6	with additional 10 mol% terpy or dtbpy	0	35
7	30 °C	54	16
8	40 °C	42	19
9	10 mol% Ni, 10 mol% L	34, 27 SM	17
10	10 mol% Ni, 10 mol% L, 2.0 atm, 0.25 mL DMA	40	15
11	10 mol% Ni, 10 mol% L, 2.5 atm, 0.25 mL DMA	38	18
12	Mn	trace	33
13	TDAE	trace	15

Note: By changing the zinc to activated zinc, and increasing the amount of NaI to 4 equiv, a higher yield (54%) could be obtained. If reducing the catalyst loading, the reaction becomes slower, after 20 h, starting material remained. <u>If the yield of dimer is 20%, it means 40% of the starting material converted to the dimer during the reaction.</u>

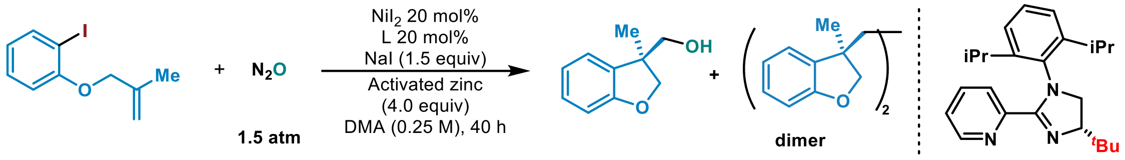

Entry	Deviation from standard conditions	Yield %	Yield of the dimer%
1	NaI 1.0, 2.0, 3.0 equiv	42-53	14-20
2	using $NiBr_2$•DME	25	23
3	using CsI, TBAI, TMAI	48, 39, 31	9, 11
4	1.0 atm, NaI 1,5 equiv	53	11
5	1.25 atm, NaI 1,5 or 3.0 equiv	55, 56	19
6	1.25 atm, NaI 2.25 equiv	65 (94% ee)	9
7	1.35 atm, NaI 2.25 equiv	65 (94% ee)	8
8	1.35 atm, NaI 2.25 equiv, DMA 0.3 mL	51	11
9	10 mol% NiI_2	20%, 61% SM	< 5%

Note: Extending the reaction time (40 h), and application of the ligand depicted, resulted in higher yield. Increasing the pressure of N_2O or adding more NaI in the reaction will promote the formation of byproduct (dimer). <u>If the yield of dimer is 20%, it means 40% of the starting material converted to the dimer during the reaction.</u>

Due to the higher catalyst loading, we kept optimizing the reaction conditions.

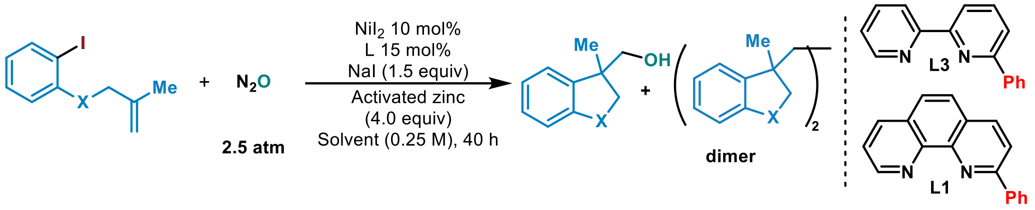

Entry	X	Ligand	Solvent	Yield %	Yield of the dimer%
1 *24h*	O	L3	DMSO	33, 35% SM	11
2	O	L1	DMF	33	10
3	O	L1	DMA	31	12
4	O	L1	DMSO	43	16
5	CH_2	L3	DMA	41	< 5%
6	CH_2	L1	DMA	0	< 5%
7	CH_2	L1	DMF	75	< 5%
8	CH_2	L1	DMSO	89	< 5%
9 *under Argon*	CH_2	L1	DMSO	0	< 5%

*Note: By changing the substrate, solvent and ligand, increasing the pressure of N_2O, we could get a 89% yield. Using this modified phenanthroline ligand (**L1**), the byproduct of dimer could be suppressed (less than 8%). <u>If the yield of dimer is 20%, it means 40% of the starting material converted to the dimer during the reaction.</u>*

5 Unsuccessful substrates

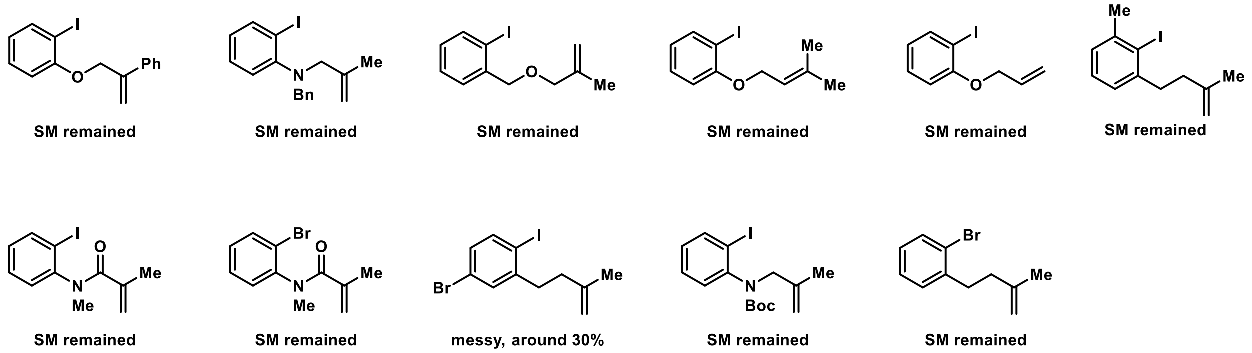

6 General Procedure for Ni-catalyzed oxygen transfer from N_2O into sp^3 hybridized carbons (*racemic*) (General Procedure A)

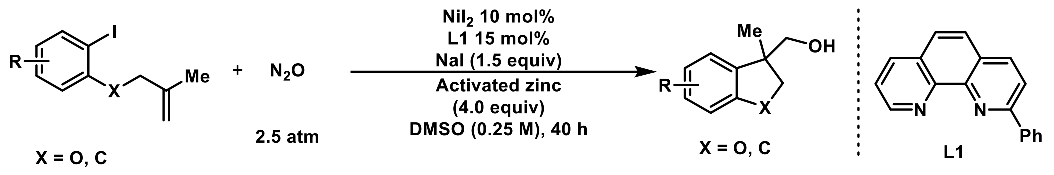

An oven- and heatgun-dried pressure Schlenk tube with a Teflon screw-cap equipped with a Teflon-coated stir bar were used. The Schlenk tube was brought into an argon-filled glovebox, and NiI_2 (0.01 mmol, 10 mol%), **L1** (0.015 mmol, 15 mol%), activated Zn (0.4 mmol, 4 equiv) and NaI (0.15 mmol, 1.5 equiv) were introduced into the pressure Schlenk. Then, outside the glovebox, using a T-connection, the Schlenk was evacuated and backfilled with N_2O (three times). Under N_2O flow, the Schlenk was opened and DMSO (0.4 mL) was added using a syringe. The substrate was added subsequently using a Hamilton syringe. The Schlenk was then closed, and the pressure of N_2O was increased to 2.4 – 2.5 atm. The reaction mixture was kept stirring at 1000 rpm, for 40 h, at room temperature. After the completion of reaction, the mixture was diluted with MTBE, washed with aqueous solution of HCl (1 M) and brine, and dried over Na_2SO_4. Upon filtration, the organic layer was concentrated under reduced pressure (water bath at 40 °C) and purified by flash column chromatography (silica gel) or preparative TLC (pTLC) to afford the desired product.

7 General Procedure for Ni-catalyzed oxygen transfer from N_2O into sp^3 hybridized carbons (*chiral*) (General Procedure B)

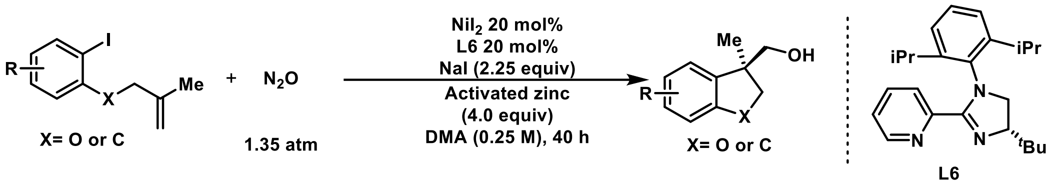

An oven- and heatgun-dried pressure Schlenk tube with Teflon screw-cap equipped with a Teflon-coated stir bar were used. The Schlenk tube was brought into an argon-filled glovebox, and NiI_2 (0.01 mmol, 0.2 equiv), activated Zn (0.4 mmol, 4 equiv) and NaI (0.225 mmol, 2.25 equiv) were introduced into the pressure Schlenk. Then, outside the glovebox, using a T-connection, the Schlenk was evacuated and backfilled with N_2O (three times). Under N_2O flow, the Schlenk was opened and a solution of **L6** (0.02 mmol, 0.2 equiv) in DMA (0.4 mL) was added using a syringe. The substrate was added subsequently using a Hamilton syringe. The Schlenk was then closed, and the pressure of N_2O was increased to 1.3 – 1.35 atm. The reaction mixture was kept stirring at 1000 rpm, for 40 h, at room temperature. After the completion of reaction, the mixture was diluted with MTBE, washed with an aqueous solution of HCl (1 M) and brine, and dried over Na_2SO_4. Upon filtration, the organic layer was concentrated under reduced pressure (water bath at 40 °C) and purified by flash column chromatography (silica gel) or preparative TLC (pTLC) to afford the desired product.

8 Graphical Procedure for Ni-catalyzed oxygen transfer from N_2O into sp^3 hybridized carbons (*racemic*)

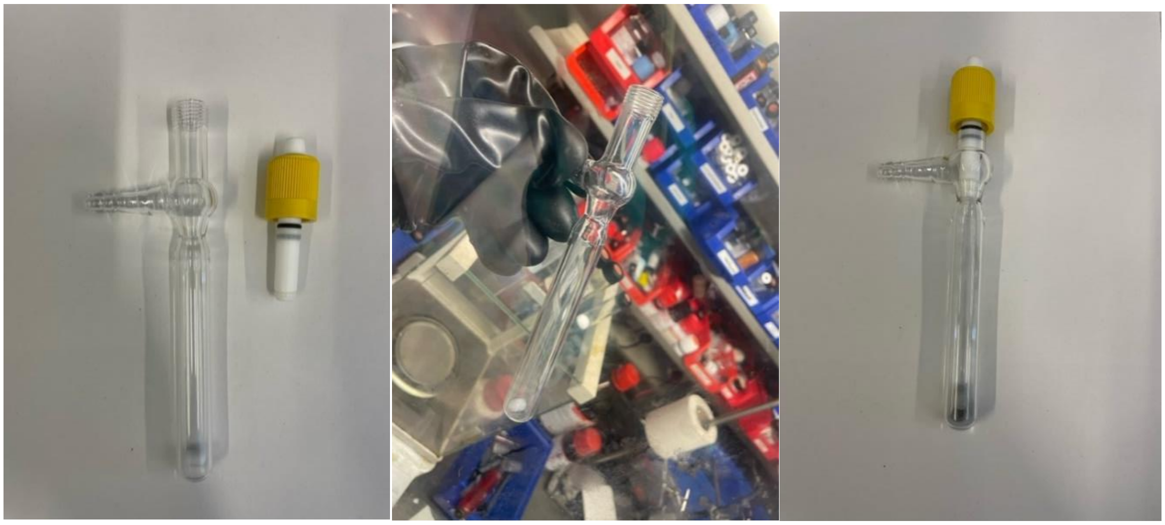

(Left) An oven- and heatgun-dried pressure Schlenk tube with a Teflon screw-cap. **(Center)** In the glovebox, NiI_2 (0.01 mmol, 10 mol%), **L1** (0.015 mmol, 15 mol%), activated Zn (0.4 mmol, 4 equiv) and NaI (0.15 mmol, 1.5 equiv) were introduced in the pressure Schlenk. **(Right)** Close the cap, and take the schlenk out.

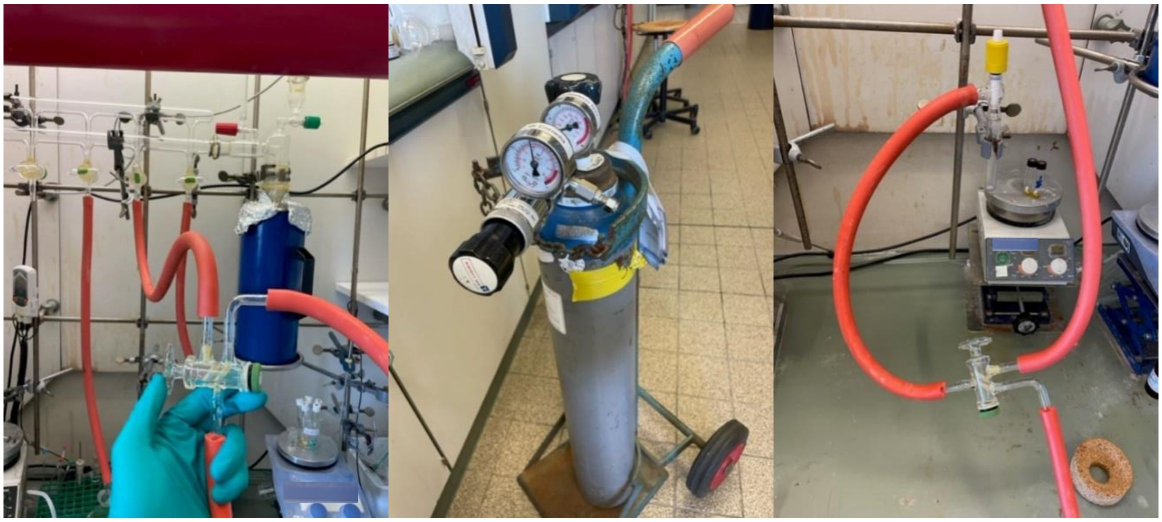

(Left) T-connection **(Center)** N_2O bottle **(Right)** connect the Schlenk line, the N_2O bottle, and the Schlenk tube.

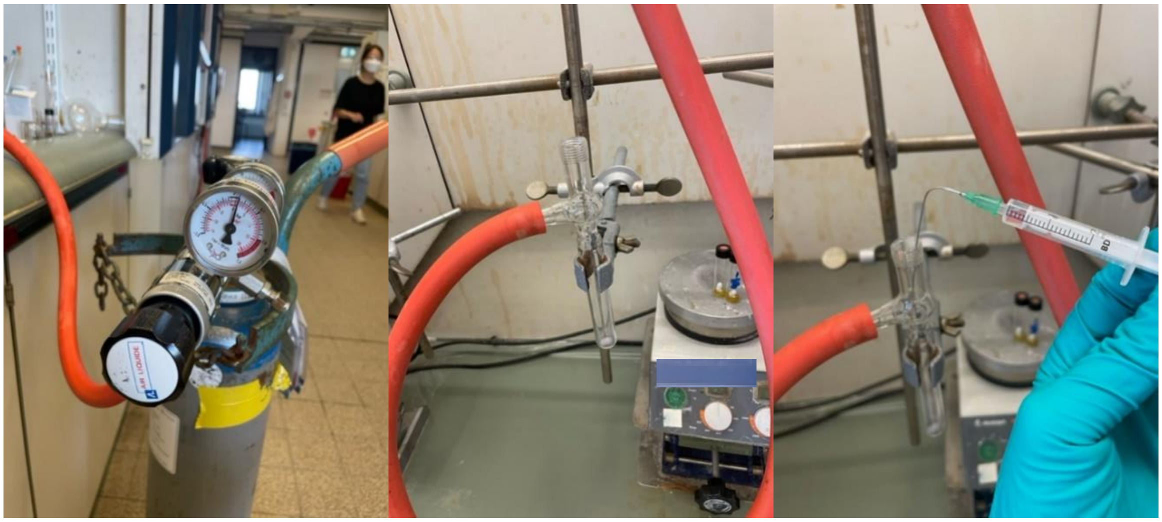

(Left) open the N_2O bottle, and the Schlenk was evacuated and backfilled with N_2O (three times). **(Center)** open the Schlenk under N_2O flow. **(Right)** adding the DMSO (0.4 mL).

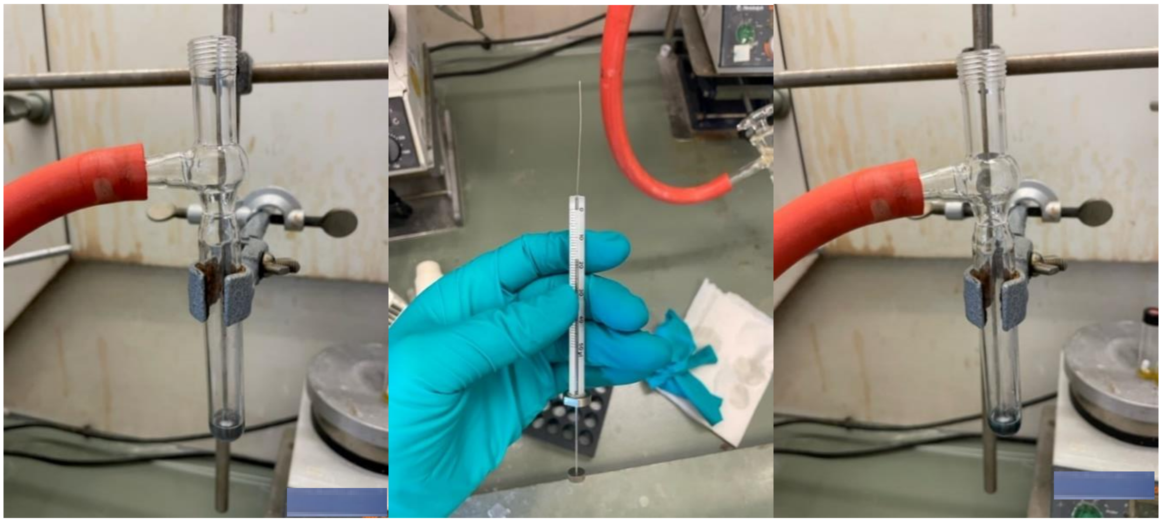

(Left) after adding the solvent. **(Center)** adding the substrate using a Hamilton syringe. **(Right)** after adding the substrate.

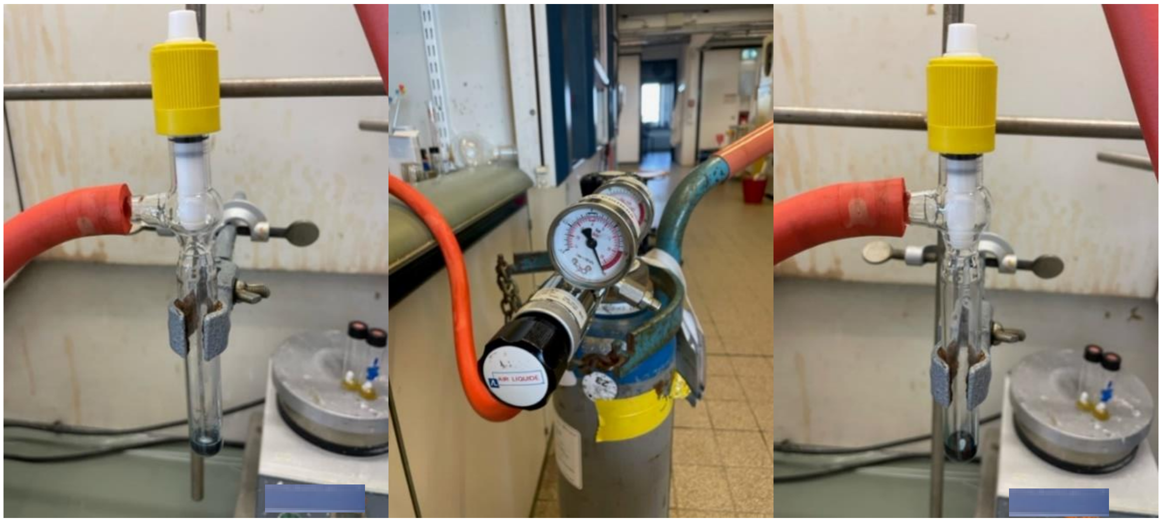

(Left) close the Schlenk. **(Center)** the pressure of N_2O was increased to 2.4 – 2.5 atm. **(Right)** close the Schlenk completely.

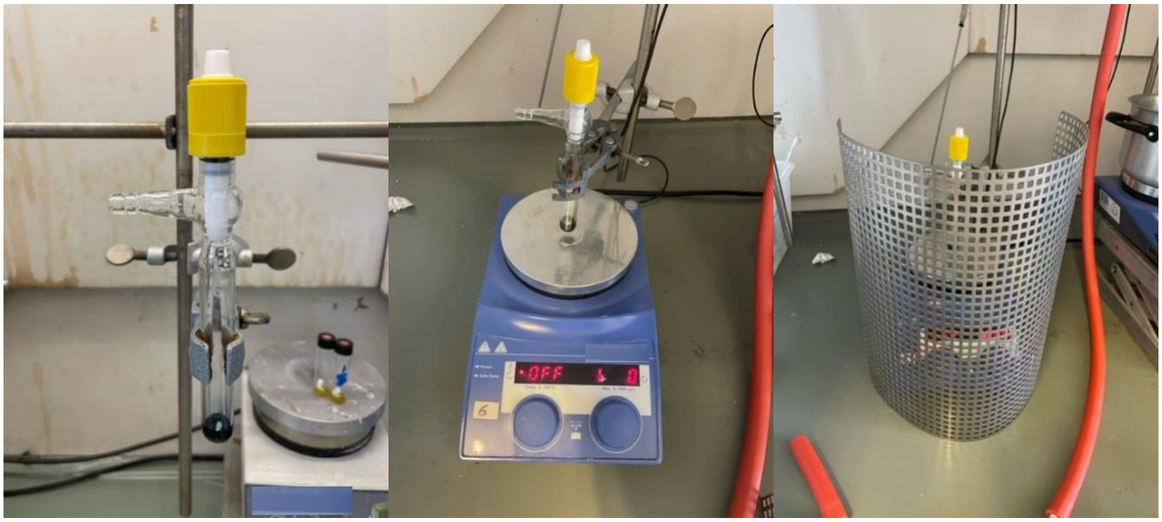

(Left) remove the Schlenk from the T-connection. **(Center)** the reaction mixture was kept stirring at 1000 rpm. **(Right)** put a blast shield for safety.

Safety note: The reaction run under pressure (2.5 atm) should be run behind a blast shield.

9 Characterization Data

Compound 2a

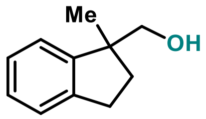

Following **General Procedure A** on 0.10 mmol scale. Purification by pTLC (3:1 hexanes:EtOAc) afforded 14.1 mg (87%) of the title compound **2a**.

Physical State: colorless oil.

R_f = 0.36 (5:1 hexanes:EtOAc).

^{1}H NMR (300 MHz, $CDCl_3$): δ 7.25 – 7.14 (m, 4H), 3.63 (d, *J* = 10.8 Hz, 1H), 3.55 (d, *J* = 10.8 Hz, 1H), 3.01 – 2.88 (m, 2H), 2.20 (ddd, *J* = 12.8, 7.6, 5.7 Hz, 1H), 1.97 – 1.77 (m, 1H), 1.29 (s, 3H) ppm.

^{13}C NMR (75 MHz, $CDCl_3$): δ 147.9, 144.5, 127.2, 126.6, 125.0, 123.1, 70.3, 49.9, 36.1, 30.5, 23.7 ppm.

HRMS (ESI-TOF): calc'd for $C_{11}H_{14}O_1Na_1$ $[M+Na]^+$: 185.093684, found: 185.093900.

Compound 2b

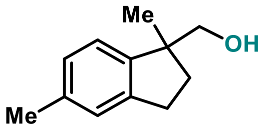

Following **General Procedure A** on 0.10 mmol scale. Purification by pTLC (3:1 hexanes:EtOAc) afforded 15.0 mg (85%) of the title compound **2b**.

Physical State: colorless oil.

R_f = 0.36 (4:1 hexanes:EtOAc).

^{1}H NMR (300 MHz, CDCl$_3$): δ 7.13 – 6.93 (m, 3H), 3.61 (d, *J* = 10.8 Hz, 1H), 3.53 (d, *J* = 10.8 Hz, 1H), 2.96 – 2.82 (m, 2H), 2.34 (s, 3H), 2.26 – 2.10 (m, 1H), 1.93 – 1.75 (m, 1H), 1.35 (br s, 1H), 1.28 (s, 3H) ppm.

^{13}C NMR (75 MHz, CDCl$_3$): δ 145.0, 144.6, 136.9, 127.4, 125.7, 122.8, 70.4, 49.5, 36.3, 30.3, 23.7, 21.4 ppm.

HRMS (EI-TOF): calc'd for $C_{12}H_{16}O_1$ $[M]^+$: 176.119565, found: 176.119600.

Compound 2c

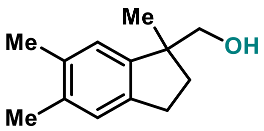

Following **General Procedure A** on 0.10 mmol scale. Purification by pTLC (3:1 hexanes:EtOAc) afforded 17.5 mg (92%) of the title compound **2c**.

Physical State: colorless oil.

R_f = 0.36 (5:1 hexanes:EtOAc).

^{1}H NMR (300 MHz, CDCl$_3$): δ 7.02 (s, 1H), 6.94 (s, 1H), 3.61 (d, *J* = 10.7 Hz, 1H), 3.53 (d, *J* = 10.7 Hz, 1H), 2.92 – 2.79 (m, 2H), 2.26 (s, 3H), 2.24 (s, 3H), 2.22 – 2.11 (m, 1H), 1.82 (dt, *J* = 12.7, 7.9 Hz, 1H), 1.27 (s, 3H) ppm.

^{13}C NMR (75 MHz, CDCl$_3$): δ 145.5, 142.0, 135.5, 134.8, 126.2, 124.1, 70.4, 49.7, 36.4, 30.0, 23.8, 20.0, 19.9 ppm.

HRMS (ESI-TOF): calc'd for $C_{13}H_{17}O_1$ $[M-H]^-$: 189.127390, found: 189.127510.

Compound 2d

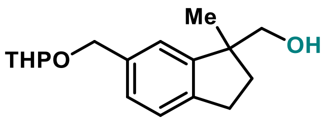

Following **General Procedure A** on 0.10 mmol scale. Purification by pTLC (3:1 hexanes:EtOAc) afforded 23.2 mg (84%) of the title compound **2d**.

Physical State: colorless oil.

R_f = 0.22 (5:1 hexanes:EtOAc).

^{1}H NMR (300 MHz, $CDCl_3$): δ 7.20 (s, 2H), 7.16 (s, 1H), 4.77 (d, *J* = 11.7 Hz, 1H), 4.71 (t, *J* = 3.7 Hz, 1H), 4.48 (d, *J* = 11.7 Hz, 1H), 4.01 – 3.87 (m, 1H), 3.67 – 3.47 (m, 3H), 2.96 – 2.85 (m, 2H), 2.29 – 2.13 (m, 1H), 1.93 – 1.80 (m, 2H), 1.78 – 1.47 (m, 6H), 1.29 (s, 3H) ppm.

^{13}C NMR (75 MHz, $CDCl_3$): δ 148.2, 144.0, 136.7, 127.2, 124.9 (d, *J* = 1.4 Hz), 122.8 (d, *J* = 2.4 Hz), 98.0 (d, *J* = 1.4 Hz), 70.3, 69.3 (d, *J* = 2.2 Hz), 62.4, 49.9, 36.3, 30.8, 30.2, 25.6, 23.7, 19.6 ppm.

HRMS (ESI-TOF): calc'd for $C_{17}H_{24}O_3Na_1$ $[M+Na]^+$: 229.161764, found: 299.161710.

Compound 2e

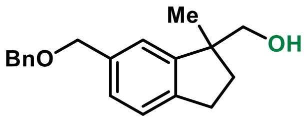

Following **General Procedure A** on 0.10 mmol scale. Purification by pTLC (3:1 hexanes:EtOAc) afforded 20.6 mg (73%) of the title compound **2e**.

Physical State: colorless oil.

R_f = 0.32 (5:1 hexanes:EtOAc).

^{1}H NMR (300 MHz, $CDCl_3$): δ 7.37 – 7.05 (m, 8H), 4.49 (s, 2H), 4.46 (s, 2H), 3.52 (q, *J* = 10.8 Hz, 2H), 2.94 – 2.78 (m, 2H), 2.19 – 2.06 (m, 1H), 1.90 – 1.70 (m, 1H), 1.43 (br s, 1H), 1.22 (s, 3H) ppm.

^{13}C NMR (75 MHz, $CDCl_3$): δ 148.3, 144.0, 138.5, 136.7, 128.5, 128.0, 127.8, 127.1, 124.9, 122.7, 72.5, 72.3, 70.4, 49.9, 36.3, 30.2, 23.7 ppm.

HRMS (ESI-TOF): calc'd for $C_{19}H_{21}O_2$ $[M-H]^-$: 281.153605, found: 281.153740.

Compound 2f

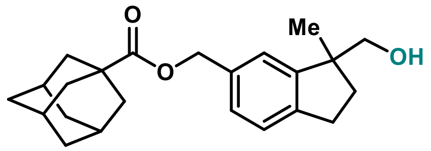

Following **General Procedure A** on 0.10 mmol scale. Purification by pTLC (3:1 hexanes:EtOAc) afforded 14.2 mg (40%) of the title compound **2f**.

Physical State: colorless oil.

R_f = 0.24 (5:1 hexanes:EtOAc).

^{1}H NMR (600 MHz, $CDCl_3$): δ 7.21 (dq, *J* = 7.6, 0.9 Hz, 1H), 7.16 (dd, *J* = 7.6, 1.6 Hz, 1H), 7.11 (s, 1H), 5.08 (s, 2H), 3.62 (d, *J* = 10.8 Hz, 1H), 3.55 (d, *J* = 10.8 Hz, 1H), 2.93 – 2.91 (m, 2H), 2.24 – 2.17 (m, 1H), 2.04 – 1.98 (m, 3H), 1.94 – 1.89 (m, 6H), 1.86 (ddd, *J* = 12.8, 8.7, 7.6 Hz, 1H), 1.76 – 1.66 (m, 6H), 1.29 (s, 3H) ppm.

^{13}C NMR (151 MHz, $CDCl_3$): δ 177.7, 148.4, 144.3, 135.1, 126.9, 125.0, 122.6, 70.3, 66.1, 49.8, 40.9, 39.0, 36.6, 36.3, 30.2, 28.1, 23.6 ppm.

HRMS (ESI-TOF): calc'd for $C_{23}H_{30}O_3Na_1$ $[M+Na]^+$: 377.208714, found: 377.208350.

Compound 2g

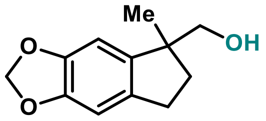

Following **General Procedure A** on 0.10 mmol scale. Purification by pTLC (3:1 hexanes:EtOAc) afforded 15.5 mg (75%) of the title compound **2g**.

Physical State: colorless oil.

R_f = 0.28 (5:1 hexanes:EtOAc).

^{1}H NMR (300 MHz, CDCl$_3$): δ 6.68 (s, 1H), 6.64 (s, 1H), 5.92 (d, J = 1.4 Hz, 1H), 5.91 (d, J = 1.4 Hz, 1H), 3.57 (d, J = 10.7 Hz, 1H), 3.50 (d, J = 10.8 Hz, 1H), 2.92 – 2.72 (m, 2H), 2.19 (ddd, J = 13.2, 7.7, 5.5 Hz, 1H), 1.85 (ddd, J = 12.8, 8.5, 7.6 Hz, 1H), 1.34 (br s, 1H), 1.23 (s, 3H) ppm.

^{13}C NMR (75 MHz, CDCl$_3$): δ 147.1, 146.8, 140.7, 137.1, 105.5, 103.7, 101.1, 70.5, 49.6, 36.6, 30.4, 23.8 ppm.

HRMS (ESI-TOF): calc'd for $C_{12}H_{14}O_3Na_1$ $[M+Na]^+$: 229.083514, found: 229.083510.

Compound 2h

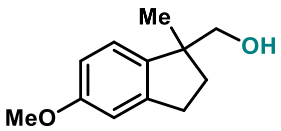

Following **General Procedure A** on 0.10 mmol scale. Purification by pTLC (3:1 hexanes:EtOAc) afforded 14.2 mg (74%) of the title compound **2h**.

Physical State: colorless oil.

R_f = 0.31 (5:1 hexanes:EtOAc).

^{1}H NMR (300 MHz, CDCl$_3$): δ 7.06 (dd, J = 8.1, 0.6 Hz, 1H), 6.83 – 6.70 (m, 2H), 3.79 (s, 3H), 3.59 (d, J = 10.7 Hz, 1H), 3.52 (d, J = 10.7 Hz, 1H), 2.94 – 2.85 (m, 2H), 2.20 (ddd, J = 13.2, 7.8, 5.6 Hz, 1H), 1.85 (ddd, J = 12.8, 8.5, 7.8 Hz, 1H), 1.39 (br s, 1H), 1.26 (s, 3H) ppm.

^{13}C NMR (75 MHz, CDCl$_3$): δ 159.4, 146.0, 140.0, 123.6, 112.5, 110.4, 70.5, 55.5, 49.2, 36.4, 30.6, 23.9 ppm.

HRMS (ESI-TOF): calc'd for $C_{12}H_{17}O_2$ $[M+H]^+$: 193.122305, found: 193.122460.

Compound 2i

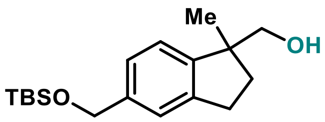

Following **General Procedure A** on 0.10 mmol scale. Purification by pTLC (3:1 hexanes:EtOAc) afforded 22.0 mg (72%) of the title compound **2i**.

Physical State: colorless oil.

R_f = 0.34 (5:1 hexanes:EtOAc).

^{1}H NMR (300 MHz, $CDCl_3$): δ 7.22 – 7.05 (m, 3H), 4.71 (s, 2H), 3.61 (d, *J* = 10.8 Hz, 1H), 3.53 (d, *J* = 10.8 Hz, 1H), 3.02 – 2.83 (m, 2H), 2.27 – 2.11 (m, 1H), 1.99 – 1.75 (m, 1H), 1.48 (br s, 1H), 1.28 (s, 3H), 0.95 (s, 9H), 0.11 (s, 6H) ppm.

^{13}C NMR (75 MHz, $CDCl_3$): δ 146.7, 144.6, 140.6, 124.7, 122.9, 122.8, 77.6, 77.2, 76.7, 70.4, 65.2, 49.7, 36.3, 30.4, 26.2, 23.7, 18.6, -5.1 ppm.

HRMS (ESI-TOF): calc'd for $C_{18}H_{30}O_2Si_1Na_1$ $[M+Na]^+$: 329.190728, found: 329.190890.

Compound 2j

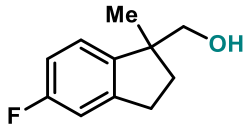

Following **General Procedure A** on 0.10 mmol scale. Purification by pTLC (3:1 hexanes:EtOAc) afforded 13.1 mg (73%) of the title compound **2j**.

Physical State: colorless oil.

R_f = 0.37 (5:1 hexanes:EtOAc).

^{1}H NMR (300 MHz, $CDCl_3$): δ 7.09 (dd, *J* = 8.1, 5.2 Hz, 1H), 6.96 – 6.80 (m, 2H), 3.60 (d, *J* = 10.7 Hz, 1H), 3.53 (d, *J* = 10.8 Hz, 1H), 2.95 – 2.84 (m, 2H), 2.28 – 2.12 (m, 1H), 1.87 (dt, *J* = 12.9, 8.0 Hz, 1H), 1.38 (br s, 1H), 1.27 (s, 3H) ppm.

^{13}C NMR (75 MHz, $CDCl_3$): δ 162.6 (d, *J* = 243.5 Hz), 146.6 (d, *J* = 7.9 Hz), 143.5 (d, *J* = 2.5 Hz), 124.0 (d, *J* = 8.9 Hz), 113.4 (d, *J* = 22.4 Hz), 111.9 (d, *J* = 21.8 Hz), 70.3, 49.3, 36.4, 30.4 (d, *J* = 2.2 Hz), 23.8 ppm.

^{19}F NMR (282 MHz, $CDCl_3$): δ -116.77 ppm.

HRMS (ESI-TOF): calc'd for $C_{11}H_{12}O_1F_1$ $[M-H]^-$: 179.086668, found: 179.086540.

Compound 2k

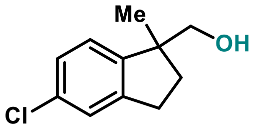

Following **General Procedure A** on 0.10 mmol scale. Purification by pTLC (3:1 hexanes:EtOAc) afforded 13.9 mg (71%) of the title compound **2k**.

Physical State: colorless oil.

R_f = 0.36 (5:1 hexanes:EtOAc).

^{1}H NMR (300 MHz, $CDCl_3$): δ 7.22 – 7.13 (m, 2H), 7.08 (d, *J* = 8.0 Hz, 1H), 3.64 – 3.56 (m, 1H), 3.52 (dt, *J* = 10.8, 0.8 Hz, 1H), 2.98 – 2.81 (m, 2H), 2.29 – 2.12 (m, 1H), 1.86 (dt, *J* = 12.9, 8.0 Hz, 1H), 1.27 (s, 3H) ppm.

^{13}C NMR (75 MHz, $CDCl_3$): δ 146.6, 146.4, 132.8, 126.7, 125.2, 124.2, 70.2, 49.5, 36.1, 30.3, 23.6 ppm.

HRMS (EI-TOF): calc'd for $C_{11}H_{13}O_1Cl_1$ $[M]^+$: 196.064943, found: 196.064980.

Compound 2l

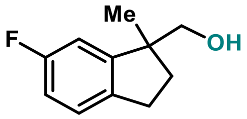

Following **General Procedure A** on 0.10 mmol scale. Purification by pTLC (3:1 hexanes:EtOAc) afforded 8.1 mg (45%) of the title compound **2l**.

Physical State: colorless oil.

R_f = 0.36 (5:1 hexanes:EtOAc).

^{1}H NMR (600 MHz, CDCl$_3$): δ 7.17 – 7.11 (m, 1H), 6.91 – 6.83 (m, 2H), 3.61 (d, *J* = 10.8 Hz, 1H), 3.55 (d, *J* = 10.8 Hz, 1H), 2.95 – 2.81 (m, 2H), 2.27 – 2.18 (m, 1H), 1.93 – 1.83 (m, 1H), 1.27 (s, 3H) ppm.

^{13}C NMR (151 MHz, CDCl$_3$): δ 162.4 (d, *J* = 243.2 Hz), 150.4 (d, *J* = 7.0 Hz), 139.5 (d, *J* = 2.5 Hz), 125.8 (d, *J* = 8.4 Hz), 114.0 (d, *J* = 22.2 Hz), 110.2 (d, *J* = 22.0 Hz), 70.2, 50.1, 36.6, 29.8, 23.5 ppm.

^{19}F NMR (282 MHz, CDCl$_3$): δ -117.06 ppm.

HRMS (EI-TOF): calc'd for $C_{11}H_{13}O_1F_1$ $[M]^+$: 180.094493, found: 180.094530.

Compound 2m

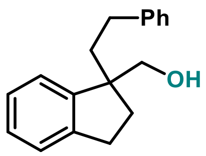

Following **General Procedure A** on 0.10 mmol scale. Purification by pTLC (3:1 hexanes: EtOAc) afforded 12.9 mg (51%) of the title compound **2m**.

Physical State: colorless oil.

R_f = 0.28 (5:1 hexanes:EtOAc).

^{1}H NMR (300 MHz, CDCl$_3$): δ 7.34 – 7.09 (m, 9H), 3.73 (d, *J* = 10.8 Hz, 1H), 3.62 (d, *J* = 10.8 Hz, 1H), 2.96 (t, *J* = 7.9 Hz, 2H), 2.60 (td, *J* = 12.9, 5.3 Hz, 1H), 2.42 (td, *J* = 12.8, 4.8 Hz, 1H), 2.21 – 1.84 (m, 4H) ppm.

^{13}C NMR (75 MHz, CDCl$_3$): δ 146.3, 145.0, 142.9, 128.5, 128.4, 127.4, 126.6, 125.9, 125.1, 123.6, 69.3, 53.8, 38.6, 33.0, 31.0, 30.8 ppm.

HRMS (ESI-TOF): calc'd for $C_{18}H_{20}O_1Na_1$ $[M+Na]^+$: 275.140634, found: 275.140570.

Compound 2n

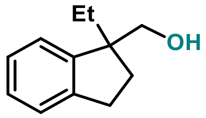

Following **General Procedure A** on 0.10 mmol scale. Purification by pTLC (3:1 hexanes:EtOAc) afforded 14.8 mg (84%) of the title compound **2n**.

Physical State: colorless oil.

R_f = 0.36 (5:1 hexanes:EtOAc).

^{1}H NMR (300 MHz, $CDCl_3$): δ 7.25 – 7.08 (m, 4H), 3.70 (d, *J* = 10.8 Hz, 1H), 3.58 (d, *J* = 10.9 Hz, 1H), 2.99 – 2.84 (m, 2H), 2.13 – 1.90 (m, 2H), 1.83 – 1.57 (m, 2H), 1.38 – 1.22 (m, 1H), 0.82 (t, *J* = 7.5 Hz, 3H) ppm.

^{13}C NMR (75 MHz, $CDCl_3$): δ 146.5, 145.1, 127.2, 126.4, 125.0, 123.6, 68.9, 53.9, 32.6, 30.8, 28.9, 8.8 ppm.

HRMS (ESI-TOF): calc'd for $C_{12}H_{16}O_1Na_1$ $[M+Na]^+$: 199.109334, found: 199.109530.

Compound 2o

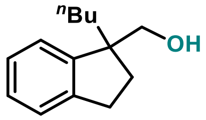

Following **General Procedure A** on 0.10 mmol scale. Purification by pTLC (3:1 hexanes:EtOAc) afforded 17.7 mg (87%) of the title compound **2o**.

Physical State: colorless oil.

R_f = 0.35 (5:1 hexanes: EtOAc).

^{1}H NMR (300 MHz, $CDCl_3$): δ 7.25 – 7.11 (m, 4H), 3.69 (d, *J* = 10.9 Hz, 1H), 3.57 (d, *J* = 10.9 Hz, 1H), 2.97 – 2.86 (m, 2H), 2.15 – 1.91 (m, 2H), 1.80 – 1.52 (m, 2H), 1.38 – 1.02 (m, 4H), 0.87 (t, *J* = 7.0 Hz, 3H) ppm.

^{13}C NMR (75 MHz, CDCl$_3$): δ 146.8, 145.0, 127.2, 126.4, 125.0, 123.5, 69.3, 53.6, 36.3, 33.1, 30.8, 26.7, 23.6, 14.2 ppm.

HRMS (ESI-TOF): calc'd for $C_{14}H_{19}O_1$ [M-H]$^-$: 203.143040, found: 203.143070.

Compound 2p

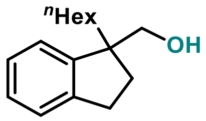

Following **General Procedure A** on 0.10 mmol scale. Purification by pTLC (3:1 hexanes:EtOAc) afforded 18.7 mg (76%) of the title compound **2p**.

Physical State: colorless oil.

R_f = 0.35 (5:1 hexanes:EtOAc).

^{1}H NMR (300 MHz, CDCl$_3$): δ 7.25 – 7.08 (m, 4H), 3.69 (d, *J* = 10.8 Hz, 1H), 3.57 (d, *J* = 10.8 Hz, 1H), 3.04 – 2.80 (m, 2H), 2.13 – 1.91 (m, 2H), 1.81 – 1.50 (m, 2H), 1.41 – 1.23 (m, 7H), 1.17 – 1.04 (m, 1H), 0.91 – 0.81 (m, 3H) ppm.

^{13}C NMR (151 MHz, CDCl$_3$): δ 146.8, 145.0, 127.2, 126.4, 125.0, 123.5, 69.3, 53.6, 36.6, 33.1, 31.9, 30.8, 30.2, 24.4, 22.8, 14.2 ppm.

HRMS (ESI-TOF): calc'd for $C_{16}H_{23}O_1$ [M-H]$^-$: 231.174340, found: 231.174030.

Compound 2q

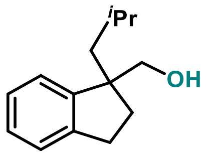

Following **General Procedure A** on 0.10 mmol scale. Purification by pTLC (3:1 hexanes:EtOAc) afforded 16.9 mg (83%) of the title compound **2q**.

Physical State: colorless oil.

R$_f$ = 0.34 (5:1 hexanes:EtOAc).

^{1}H NMR (300 MHz, CDCl$_3$): δ 7.25 – 7.08 (m, 4H), 3.68 (d, *J* = 10.8 Hz, 1H), 3.54 (d, *J* = 10.8 Hz, 1H), 2.93 (t, *J* = 7.7 Hz, 2H), 2.19 – 2.00 (m, 2H), 1.80 – 1.62 (m, 2H), 1.58 – 1.45 (m, 1H), 1.22 (br s, 1H), 0.91 (d, *J* = 6.4 Hz, 3H), 0.75 (d, *J* = 6.5 Hz, 3H) ppm.

^{13}C NMR (75 MHz, CDCl$_3$): δ 147.2, 144.8, 127.2, 126.4, 125.0, 123.7, 69.7, 53.9, 45.3, 33.2, 30.9, 25.5, 25.0, 24.7 ppm.

HRMS (ESI-TOF): calc'd for $C_{14}H_{20}O_1Na_1$ [M+Na]$^+$: 227.140634, found: 227.140650.

Compound 2r

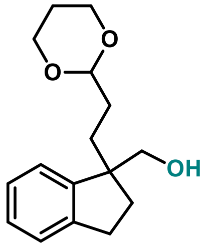

Following **General Procedure A** on 0.10 mmol scale. Purification by pTLC (3:1 hexanes:EtOAc) afforded 21.2 mg (81%) of the title compound **2r**.

Physical State: colorless oil.

R$_f$ = 0.23 (5:1 hexanes:EtOAc).

^{1}H NMR (300 MHz, CDCl$_3$): δ 7.23 – 7.11 (m, 4H), 4.46 (t, *J* = 5.0 Hz, 1H), 4.13 – 4.01 (m, 2H), 3.80 – 3.65 (m, 3H), 3.63 – 3.54 (m, 1H), 2.96 – 2.82 (m, 2H), 2.17 – 1.90 (m, 3H), 1.87 – 1.69 (m, 2H), 1.69 – 1.59 (m, 1H), 1.52 – 1.39 (m, 1H), 1.37 – 1.27 (m, 1H) ppm.

^{13}C NMR (75 MHz, CDCl$_3$): δ 146.4, 144.8, 127.3, 126.5, 125.0, 123.6, 102.7, 69.0, 67.0, 53.1, 33.0, 30.7, 30.3, 30.3, 25.9 ppm.

HRMS (EI-TOF): calc'd for $C_{16}H_{22}O_3$ [M]$^+$: 262.156345, found: 262.156540.

Compound 2s

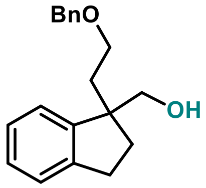

Following **General Procedure A** on 0.10 mmol scale. Purification by pTLC (3:1 hexanes:EtOAc) afforded 14.1 mg (50%) of the title compound **2s**.

Physical State: colorless oil.

R_f = 0.3 (5:1 hexanes:EtOAc).

^{1}H NMR (300 MHz, $CDCl_3$): δ 7.40 – 7.27 (m, 5H), 7.26 – 7.14 (m, 4H), 4.53 (s, 2H), 3.67 – 3.49 (m, 4H), 2.89 (ddd, J = 8.4, 6.9, 2.1 Hz, 2H), 2.20 – 2.06 (m, 2H), 1.98 – 1.80 (m, 2H) ppm.

^{13}C NMR (75 MHz, $CDCl_3$): δ 147.9, 144.0, 137.8, 128.7, 128.0, 127.2, 126.5, 125.0, 123.7, 73.6, 69.1, 67.6, 52.7, 37.1, 33.9, 30.3 ppm. (one aromatic C is missing due to overlapping)

HRMS (ESI-TOF): calc'd for $C_{19}H_{22}O_2Na_1$ $[M+Na]^+$: 305.151199, found: 305.151450.

Compound 2t

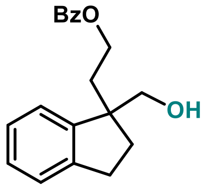

Following **General Procedure A** on 0.10 mmol scale. Purification by pTLC (3:1 hexanes:EtOAc) afforded 12.4 mg (42%) of the title compound **2t**.

Physical State: colorless oil.

R_f = 0.26 (5:1 hexanes:EtOAc).

^{1}H NMR (300 MHz, CDCl$_3$): δ 8.04 – 7.92 (m, 2H), 7.63 – 7.50 (m, 1H), 7.49 – 7.39 (m, 2H), 7.31 – 7.15 (m, 4H), 4.38 (s, 2H), 3.83 – 3.60 (m, 2H), 3.05 – 2.92 (m, 2H), 2.30 – 1.98 (m, 4H), 1.44 (br s, 1H) ppm.

^{13}C NMR (75 MHz, CDCl$_3$): δ 166.7, 145.9, 144.0, 133.1, 129.7, 128.6, 127.6, 126.6, 125.1, 123.9, 70.7, 60.0, 50.4, 39.4, 34.3, 30.6 ppm.

HRMS (ESI-TOF): calc'd for $C_{19}H_{20}O_3Na_1$ [M+Na]$^+$: 319.130464, found: 319.130320.

Compound 2u

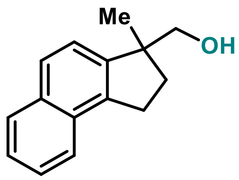

Following **General Procedure A** on 0.10 mmol scale. Purification by pTLC (3:1 hexanes:EtOAc) afforded 19.1 mg (90%) of the title compound **2u.**

Physical State: colorless oil.

R_f = 0.31 (5:1 hexanes:EtOAc).

^{1}H NMR (300 MHz, CDCl$_3$): δ 7.91 – 7.78 (m, 2H), 7.75 (d, J = 8.4 Hz, 1H), 7.58 – 7.41 (m, 2H), 7.35 (d, J = 8.4 Hz, 1H), 3.79 – 3.58 (m, 2H), 3.27 (t, J = 7.4 Hz, 2H), 2.41 (dt, J = 13.3, 6.6 Hz, 1H), 2.03 (dt, J = 12.9, 7.8 Hz, 1H), 1.37 (s, 3H) ppm.

^{13}C NMR (75 MHz, CDCl$_3$): δ 144.4, 140.1, 133.2, 130.6, 128.5, 127.5, 126.2, 125.4, 124.5, 121.5, 70.8, 50.9, 35.8, 28.9, 23.9 ppm.

HRMS (EI-TOF): calc'd for $C_{15}H_{16}O_1$ [M]$^+$: 212.119565, found: 212.119630.

Compound 2v

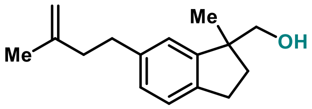

Following **General Procedure A** on 0.10 mmol scale. Purification by pTLC (3:1 hexanes:EtOAc) afforded 15.6 mg (68%) of the title compound **2v**.

Physical State: colorless oil.

R_f = 0.33 (5:1 hexanes:EtOAc).

^{1}H NMR (300 MHz, CDCl$_3$): δ 7.14 (d, *J* = 7.6 Hz, 1H), 7.06 – 6.96 (m, 2H), 4.73 (d, *J* = 8.6 Hz, 2H), 3.68 – 3.49 (m, 2H), 2.88 (t, *J* = 6.9 Hz, 2H), 2.80 – 2.70 (m, 2H), 2.38 – 2.26 (m, 2H), 2.24 – 2.14 (m, 1H), 1.85 (dt, *J* = 12.8, 8.1 Hz, 1H), 1.78 (s, 3H), 1.29 (s, 3H) ppm.

^{13}C NMR (75 MHz, CDCl$_3$): δ 148.0, 145.7, 141.9, 140.7, 127.3, 124.8, 123.0, 110.3, 70.3, 49.8, 40.1, 36.4, 34.4, 30.1, 23.6, 22.7 ppm.

HRMS (ESI-TOF): calc'd for $C_{16}H_{23}O_1$ $[M+H]^+$: 234.174340, found: 231.174530.

Compound 2w

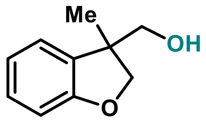

Following **General Procedure A** on 0.10 mmol scale by using DMA as the solvent. Purification by pTLC (3:1 hexanes:EtOAc) afforded 10.2 mg (62%) of the title compound **2w**.

Physical State: colorless oil.

R_f = 0.31 (5:1 hexanes:EtOAc).

^{1}H NMR (300 MHz, CDCl$_3$): δ 7.22 – 7.07 (m, 2H), 6.89 (td, *J* = 7.4, 1.0 Hz, 1H), 6.81 (d, *J* = 8.0 Hz, 1H), 4.57 (d, *J* = 8.8 Hz, 1H), 4.18 (d, *J* = 8.8 Hz, 1H), 3.67 (d, *J* = 10.8 Hz, 1H), 3.57 (d, *J* = 10.8 Hz, 1H), 1.58 (br s, 1H), 1.37 (s, 3H) ppm.

^{13}C NMR (75 MHz, CDCl$_3$): δ 160.4, 131.9, 128.9, 123.2, 120.7, 110.0, 80.2, 69.2, 47.8, 22.0 ppm.

HRMS (EI-TOF): calc'd for $C_{10}H_{12}O_2$ $[M]^+$: 164.083180, found: 164.083280.

Compound 2x

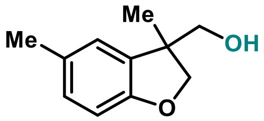

Following **General Procedure A** on 0.10 mmol scale by using DMA as the solvent. Purification by pTLC (3:1 hexanes:EtOAc) afforded 10.0 mg (56%) of the title compound **2x**.

Physical State: colorless oil.

R_f = 0.31 (5:1 hexanes:EtOAc).

^{1}H NMR (300 MHz, $CDCl_3$): δ 6.99 – 6.86 (m, 2H), 6.70 (d, *J* = 8.1 Hz, 1H), 4.55 (d, *J* = 8.8 Hz, 1H), 4.16 (d, *J* = 8.8 Hz, 1H), 3.66 (d, *J* = 10.7 Hz, 1H), 3.57 (d, *J* = 10.8 Hz, 1H), 2.30 (s, 3H), 1.48 (br s, 1H), 1.35 (s, 3H) ppm.

^{13}C NMR (75 MHz, $CDCl_3$): δ 158.3, 131.8, 130.1, 129.4, 123.7, 109.6, 80.4, 69.2, 47.9, 22.0, 21.0 ppm.

HRMS (EI-TOF): calc'd for $C_{11}H_{14}O_2$ $[M]^+$: 178.0998830, found: 178.099140.

Compound 2aa

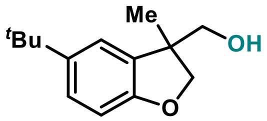

Following **General Procedure A** on 0.10 mmol scale by using DMA as the solvent. Purification by pTLC (3:1 hexanes:EtOAc) afforded 10.3 mg (47%) of the title compound **2aa**.

Physical State: colorless oil.

R_f = 0.32 (5:1 hexanes:EtOAc).

^{1}H NMR (300 MHz, $CDCl_3$): δ 7.19 (dd, *J* = 8.4, 2.1 Hz, 1H), 7.12 (d, *J* = 2.1 Hz, 1H), 6.74 (d, *J* = 8.4 Hz, 1H), 4.56 (d, *J* = 8.8 Hz, 1H), 4.17 (d, *J* = 8.8 Hz, 1H), 3.68 (d, *J* = 10.8 Hz, 1H), 3.58 (d, *J* = 10.8 Hz, 1H), 1.38 (s, 3H), 1.30 (s, 9H) ppm.

^{13}C NMR (75 MHz, CDCl$_3$): δ 158.2, 143.9, 131.3, 125.9, 119.9, 109.2, 80.5, 69.3, 48.0, 34.5, 31.9, 21.9 ppm.

HRMS (EI-TOF): calc'd for $C_{14}H_{20}O_2$ $[M]^+$: 220.145780, found: 220.146020.

Compound 2ab

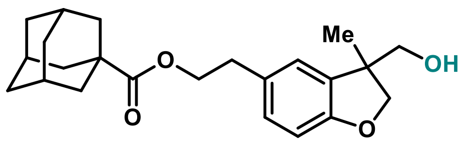

Following **General Procedure A** on 0.10 mmol scale by using DMA as the solvent. Purification by pTLC (3:1 hexanes:EtOAc) afforded 14.8 mg (40%) of the title compound **2ab**.

Physical State: colorless oil.

R_f = 0.2 (5:1 hexanes:EtOAc).

^{1}H NMR (300 MHz, CDCl$_3$): δ 6.99 (dd, *J* = 4.3, 2.4 Hz, 2H), 6.83 – 6.61 (m, 1H), 4.56 (d, *J* = 8.8 Hz, 1H), 4.39 – 4.25 (m, 1H), 4.24 – 4.12 (m, 2H), 3.66 (d, *J* = 10.8 Hz, 1H), 3.56 (d, *J* = 10.8 Hz, 1H), 2.86 (t, *J* = 6.8 Hz, 2H), 1.99 (s, 3H), 1.85 – 1.79 (m, 6H), 1.76 – 1.60 (m, 7H), 1.36 (s, 3H) ppm.

^{13}C NMR (75 MHz, CDCl$_3$): δ 177.9, 159.2, 132.1, 130.3, 129.6, 123.9, 109.6, 80.5, 69.2, 64.9, 47.9, 40.8, 39.0, 36.6, 34.9, 28.1, 22.0 ppm.

HRMS (ESI-TOF): calc'd for $C_{23}H_{29}O_4$ $[M-H]^-$: 369.207135, found: 369.207550.

Compound 2ac

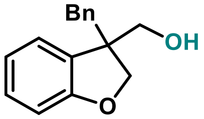

Following **General Procedure A** on 0.10 mmol scale by using DMA as the solvent. Purification by pTLC (3:1 hexanes:EtOAc) afforded 15.1 mg (63%) of the title compound **2ac**.

Physical State: colorless oil.

R_f = 0.27 (5:1 hexanes:EtOAc).

^{1}H NMR (300 MHz, $CDCl_3$): δ 7.31 – 7.13 (m, 4H), 7.09 – 6.99 (m, 3H), 6.88 (td, *J* = 7.4, 1.0 Hz, 1H), 6.77 (d, *J* = 8.0 Hz, 1H), 4.47 (d, *J* = 9.1 Hz, 1H), 4.35 (d, *J* = 9.1 Hz, 1H), 3.87 – 3.59 (m, 2H), 3.05 (d, *J* = 3.1 Hz, 2H), 1.45 (s, 1H) ppm.

^{13}C NMR (75 MHz, $CDCl_3$): δ 160.7, 137.0, 130.4, 129.2, 128.4, 126.8, 124.2, 120.5, 110.1, 78.1, 66.8, 52.2, 41.3 ppm. (one aromatic C is missing due to overlapping)

HRMS (EI-TOF): calc'd for $C_{16}H_{16}O_2$ $[M]^+$: 240.114480, found: 240.114510.

Compound 2ad

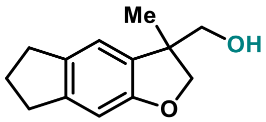

Following **General Procedure A** on 0.10 mmol scale by using L3 as the ligand. Purification by pTLC (3:1 hexanes:EtOAc) afforded 8.8 mg (43%) of the title compound **2ad.**

Physical State: colorless oil.

R_f = 0.28 (5:1 hexanes:EtOAc).

^{1}H NMR (300 MHz, $CDCl_3$): δ 6.95 (s, 1H), 6.68 (s, 1H), 4.56 (d, *J* = 8.8 Hz, 1H), 4.18 (d, *J* = 8.8 Hz, 1H), 3.66 (d, *J* = 10.8 Hz, 1H), 3.56 (d, *J* = 10.7 Hz, 1H), 2.83 (q, *J* = 6.8 Hz, 4H), 2.07 (p, *J* = 7.4 Hz, 2H), 1.50 (br s, 1H), 1.35 (s, 3H) ppm.

^{13}C NMR (151 MHz, $CDCl_3$): δ 159.4, 145.4, 136.3, 129.9, 118.8, 106.2, 80.7, 69.3, 47.6, 33.2, 32.3, 26.2, 22.1 ppm.

HRMS (EI-TOF): calc'd for $C_{13}H_{16}O_2$ $[M]^+$: 204.114480, found: 204.114650.

Compound 2ae

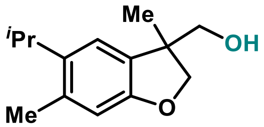

Following **General Procedure A** on 0.10 mmol scale by using **L3** as the ligand. Purification by pTLC (3:1 hexanes:EtOAc) afforded 12.3 mg (56%) of the title compound **2ae**.

Physical State: colorless oil.

R_f = 0.3 (5:1 hexanes:EtOAc).

^{1}H NMR (300 MHz, $CDCl_3$): δ 6.97 (s, 1H), 6.61 (s, 1H), 4.53 (d, *J* = 8.7 Hz, 1H), 4.15 (d, *J* = 8.7 Hz, 1H), 3.66 (d, *J* = 10.8 Hz, 1H), 3.56 (d, *J* = 10.8 Hz, 1H), 3.21 – 2.99 (m, 1H), 2.29 (s, 3H), 1.48 (br s, 1H), 1.36 (s, 3H), 1.22 (s, 3H), 1.19 (s, 3H) ppm.

^{13}C NMR (75 MHz, $CDCl_3$): δ 158.2, 139.4, 136.0, 129.4, 119.1, 111.5, 80.5, 69.3, 47.9, 29.1, 23.7, 23.7, 22.0, 19.8 ppm.

HRMS (EI-TOF): calc'd for $C_{14}H_{20}O_2$ $[M]^+$: 220.145780, found: 220.146000.

Compound (*S*)-2w

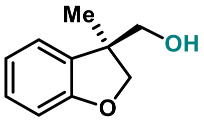

Following **General Procedure B** on 0.10 mmol scale. Purification by pTLC (3:1 hexanes:EtOAc) afforded 10.5 mg (64%) of the title compound **(*S*)-2w**.

$[\alpha]_D^{20}$ = -11.3 (*c* = 1.0, $CHCl_3$).

HPLC (IC-3, ^{n}heptan: ^{i}propanol = 98:2, 1.0 mL/min, 298 K, 220 nm): t_{R1} = 11.92 min, t_{R2} = 12.94 min, ee = 94%.

Compound (*S*)-2x

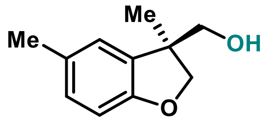

Following **General Procedure B** on 0.10 mmol scale. Purification by pTLC (3:1 hexanes:EtOAc) afforded 11.8 mg (66%) of the title compound **(*S*)-2x**.

$[\alpha]_D^{20}$ = -6.2 (c = 1.0, $CHCl_3$).

HPLC (IC-3, ^{n}heptan: ^{i}propanol = 95:5, 1.0 mL/min, 298 K, 220 nm): t_{R1} = 5.50 min, t_{R2} = 6.11 min, ee = 95%.

Compound (*S*)-2aa

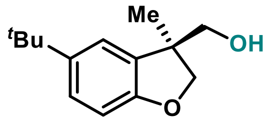

Following **General Procedure B** on 0.10 mmol scale. Purification by pTLC (3:1 hexanes:EtOAc) afforded 13.6 mg (62%) of the title compound **(*S*)-2aa**.

$[\alpha]_D^{20}$ = -4.5 (c = 0.5, $CHCl_3$).

HPLC (IC-3, ^{n}heptan: ^{i}propanol = 95:5, 1.0 mL/min, 298 K, 220 nm): t_{R1} = 3.72 min, t_{R2} = 4.43 min, ee = 96%.

Compound (*S*)-2ae

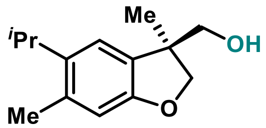

Following **General Procedure B** on 0.10 mmol scale. Purification by pTLC (3:1 hexanes:EtOAc) afforded 13.9 mg (63%) of the title compound **(*S*)-2ae**.

$[\alpha]_D^{20}$ = +2.0 (c = 0.75, $CHCl_3$).

HPLC (IB-N3, ^{n}heptan: ^{i}propanol = 98:2, 1.0 mL/min, 298 K, 220 nm): t_{R1} = 6.54 min, t_{R2} = 7.05 min, ee = 95%.

Compound (*S*)-2ad

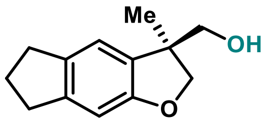

Following **General Procedure B** on 0.10 mmol scale. Purification by pTLC (3:1 hexanes:EtOAc) afforded 12.2 mg (60%) of the title compound **(*S*)-2ad**.

$[\alpha]_D^{20}$ = +3.8 (c = 0.5, $CHCl_3$).

HPLC (IC-3, ^{n}heptan: ^{i}propanol = 95:5, 1.0 mL/min, 298 K, 220 nm): t_{R1} = 6.10 min, t_{R2} = 7.59 min, ee = 95%.

Compound (*S*)-2ac

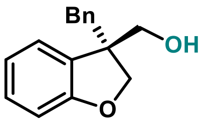

Following **General Procedure B** on 0.10 mmol scale. Purification by pTLC (3:1 hexanes:EtOAc) afforded 9.8 mg (41%) of the title compound **(*S*)-2ac**.

$[\alpha]_D^{20}$ = -24.0 (c = 1.0, $CHCl_3$).

HPLC (IC-3, ^{n}heptan: ^{i}propanol = 95:5, 1.0 mL/min, 298 K, 220 nm): t_{R1} = 5.74 min, t_{R2} = 6.48 min, ee = 97%.

Compound (*S*)-2ab

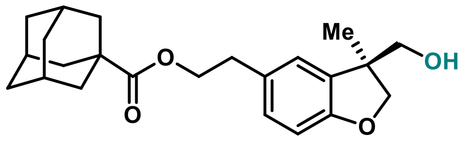

Following **General Procedure B** on 0.10 mmol scale. Purification by pTLC (3:1 hexanes:EtOAc) afforded 22.2 mg (60%) of the title compound ***(S)*-2ab**.

$[\alpha]_D^{20}$ = -10.4 (*c* = 1.0, $CHCl_3$).

HPLC (IC-3, ^{n}heptan: ^{i}propanol = 98:2, 1.0 mL/min, 298 K, 220 nm): t_{R1} = 6.81 min, t_{R2} = 8.63 min, ee = 95%.

Compound (*S*)-2a

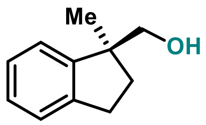

Following **General Procedure B** on 0.10 mmol scale. Purification by pTLC (3:1 hexanes:EtOAc) afforded 9.2 mg (57%) of the title compound ***(S)*-2a**.

$[\alpha]_D^{20}$ = -6.0 (*c* = 1.0, $CHCl_3$).

HPLC (IC-3, ^{n}heptan: ^{i}propanol = 98:2, 1.0 mL/min, 298 K, 220 nm): t_{R1} = 5.82 min, t_{R2} = 6.30 min, ee = 79%.

Compound (*S*)-2n

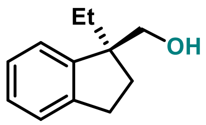

Following **General Procedure B** on 0.10 mmol scale by using **L10** as the ligand. Purification by pTLC (3:1 hexanes:EtOAc) afforded 7.9 mg (45%) of the title compound ***(S)*-2n**.

$[\alpha]_D^{20}$ = -2.0 (*c* = 1.0, $CHCl_3$).

HPLC (IB-N3, ^{n}heptan: ^{i}propanol = 98:2, 1.0 mL/min, 298 K, 220 nm): t_{R1} = 6.51 min, t_{R2} = 6.89 min, ee = 68%.

Compound (*R*)-2w-1

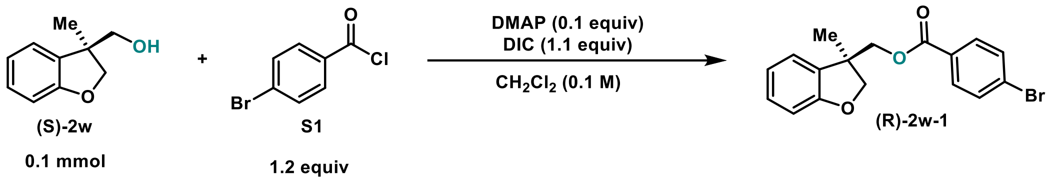

A culture tube was charged with **(*S*)-2w** (0.1 mmol, 1.0 equiv), DMAP (0.01 mmol, 0.1 equiv) and **S1** (0.12 mmol, 1.2 equiv). CH_2Cl_2 was added (1 mL), and the mixture was stirred vigorously. DIC (0.11 equiv, 1.1 equiv) was then added dropwise *via* syringe, and the mixture was stirred until the alcohol was consumed (determined by TLC). The mixture was then filtered (through a thin pad of Celite®) and rinsed with additional CH_2Cl_2. The solvent was removed under reduced pressure, and purification of the crude mixture by column chromatography (pentane:EtOAc=10:1) afforded 32.9 mg (95%) of the title compound **(*R*)-2w-1**.

Physical State: white solid.

m.p.: 63 – 65 °C.

R_f = 0.43 (10:1 hexanes:EtOAc).

^{1}H NMR (300 MHz, $CDCl_3$): δ 7.87 – 7.79 (m, 2H), 7.62 – 7.52 (m, 2H), 7.23 – 7.13 (m, 2H), 6.90 (td, *J* = 7.4, 1.0 Hz, 1H), 6.83 (dd, *J* = 8.3, 0.8 Hz, 1H), 4.60 (d, *J* = 9.0 Hz, 1H), 4.35 (s, 2H), 4.26 (d, *J* = 9.0 Hz, 1H), 1.49 (s, 3H) ppm.

^{13}C NMR (75 MHz, $CDCl_3$): δ 165.8, 160.1, 132.0, 131.4, 131.2, 129.2, 128.9, 128.4, 123.4, 120.9, 110.1, 80.4, 70.5, 46.0, 22.3 ppm.

$[\alpha]_D^{20}$ = +3.0 (*c* = 1.0, $CHCl_3$).

HRMS (EI-TOF): calc'd for $C_{17}H_{15}O_3Br_1$ $[M]^+$: 346.019920, found: 346.020320.

10 Reaction using ^{18}O-DMSO

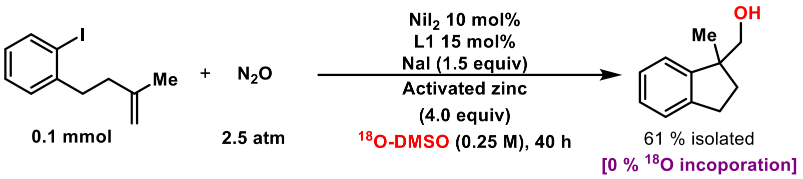

An oven- and heatgun-dried pressure Schlenk tube equipped with a Teflon screw-cap and a Teflon-coated stir bar was used. The Schlenk was brought into an argon-filled glovebox, and NiI_2 (0.01 mmol, 10 mol%), **L1** (0.015 mmol, 15 mol%), activated Zn (0.4 mmol, 4 equiv) and NaI (0.15 mmol, 1.5 equiv) were introduced into the pressure Schlenk. Then, outside the glovebox, using a T-connection, the Schlenk was then evacuated and backfilled with N_2O (three times). Under N_2O flow, the Schlenk was opened and DMSO (0.3 mL) was added using a syringe. Then, ^{18}O-DMSO (92% labeled) (0.1 mL) was added using a syringe. The substrate **1a** (0.1 mmol) was added subsequently using a Hamilton syringe. After that, the reaction mixture was kept stirring at 1000 rpm, for 40 h, at room temperature. After the completion of reaction, the reaction was first diluted using 2 mL of MTBE. The mixture was then quenched with an aqueous solution of HCl (1 M), and extracted 3 times with MTBE. The combined organic layers were washed with brine, dried over $MgSO_4$, filtered and concentrated under reduced pressure. The crude was purified by pTLC affording 9.9 mg (61% yield) of the final product. ***According to the MS analysis (see below), 0 % of ^{18}O in 2a was obtained.***

Note: The labeled ^{18}O-DMSO was synthesized following a procedure previously reported in the literature.[3] Due to the impurity in the labeled ^{18}O-DMSO (the impurity was also observed in the previous literature), if using the labeled ^{18}O-DMSO directly, only trace amount of product could be detected.

No.	MW.	Comment
1	162	Your proposed structure is possible. Compare U23693:156486a-00 NIS-NA-040-01 In this measurement there are no indications for (18)O labeling.

1.12.2021
File: 156725a-00.raw
Analyse: NIS-NA-078-01
COP: Shengyang Ni

Messung:	GC-MS
Ionisierung:	GC-EI
Spektrometer:	Q Exactive GC Orbitrap
Säule:	MS 63 ZB1HT
Länge:	30+3
Temp.:	35-15-300-3
GC-Nr.:	-
ELNA-Nr.:	34530
Auswerter:	Haupt (2243)

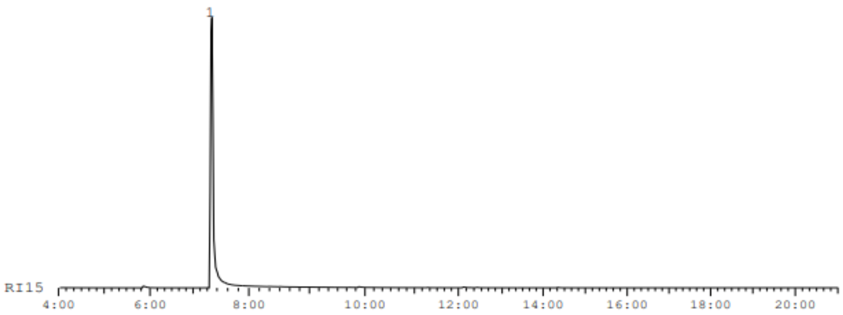

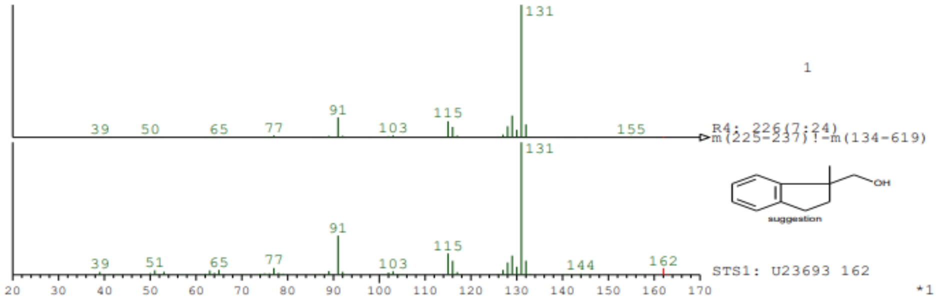

11 Reaction using *in situ* formed ^{18}O-N_2O

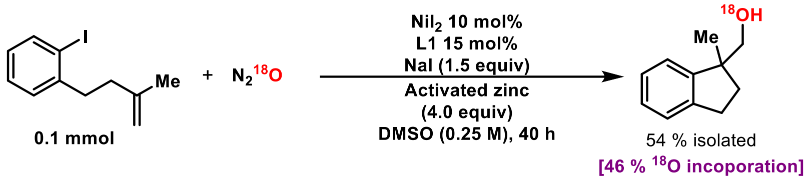

The labeled ^{18}O-N_2O was synthesized following a slightly modified procedure.[4] A reaction tube (3 mL) equipped with a Teflon-coated stir bar was used. $HONH_2 \cdot HCl$ (100 mg) was introduced into the reaction tube. After that, the tube was closed with a septum screw cap and a syringe (20 mL) with a needle pierced through the septum. Then, the reaction tube was evacuated and refilled with argon (three times). After that, H_2O (0.5 mL) was added using a syringe, while stirring. After 2 min, $Na^{15}N^{18}O_2$ (60.0 mg, 90% ^{18}O-labeled) dissolved in 0.6 mL H_2O was added drop-by-drop using a syringe. Around 15 mL *^{18}O-N_2O* gas could be collected by the syringe, which was used directly for the next step.

An oven- and heatgun-dried reaction tube was equipped with a Teflon-coated stir bar. The reaction tube was brought into an argon filled glovebox, NiI_2 (0.01 mmol, 10 mol%), **L1** (0.015 mmol, 15 mol%), activated Zn (0.4 mmol, 4 equiv) and NaI (0.15 mmol, 1.5 equiv) were introduced in the reaction tube, and a septum screw-cap employed to close it. Once outside the glovebox, the reaction tube was evacuated and removed from the vacuum line. The septum screw cap of the reaction tube was then pierced with the syringe containing the *^{18}O-N_2O*, and 15 mL gas were introduced into the reaction tube. DMSO (0.4 mL) was then added using a syringe. The substrate **1a** (0.1 mmol) was added using Hamilton syringe. After that, the reaction mixture was kept stirring at 1000 rpm, for 40 h, at RT.

After the completion of the reaction, the reaction was first diluted using 2 mL of MTBE. The mixture was quenched with an aqueous solution of HCl (1 M), and extracted 3 times with MTBE. The combined organic layers were washed with brine, dried over $MgSO_4$, filtered and concentrated under reduced pressure. The crude was purified by pTLC affording 8.9 mg (54% yield) of the final

product. ***According to the MS analysis (see below), 46% of ^{18}O incorporation into 2a was obtained.***

No.	MW.	Comment
1	162	MW 162(16O) and MW 164(18O) are included.<br>Compare U23693:156486a-00 NIS-NA-040-01<br>Ref.-spectrum: OU3122:

15.12.2021
File: 156962a-00.raw
Analyse: NIS-NA-080-01
COP: Shengyang Ni

Messung: GC-MS
Ionisierung: GC-EI
Spektrometer: Q Exactive GC Orbitrap
Säule: MS 63 ZB1HT
Länge: 30+3
Temp.: 35-15-300-3
GC-Nr.: -
ELNA-Nr.: 34760

Auswerter: Haupt (2243)

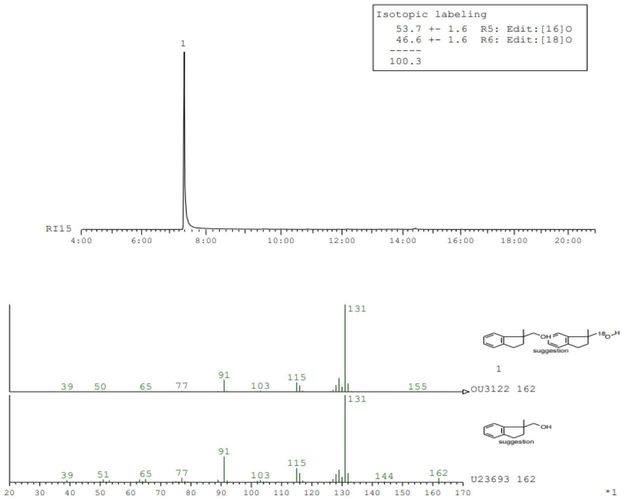

HRMS (EI-TOF): calc'd for $C_{11}H_{14}{}^{18}O_1$ [M]$^+$: 164.108159, found: 164.108080.

Mass to be matched (m/z): 162.103860 Charge: 1

Mass Tolerance: ±0.050000
Restriction of atom numbers:

C	H	O
1-150	max 150	1-5

Number of calculated Formulas: 5

Formula	Diff.(ppm)	theor. m/z
C11 H14 O1	0.34	162.103915
C7 H14 O4	-93.77	162.088660
C8 H18 O3	130.69	162.125045
C10 H10 O2	-224.12	162.067530
C5 H22 O5	261.04	162.146175

Suggestion:
C11H14O1 MW: 162

15.12.2021
File: 156962a-00.raw
Analyse: NIS-NA-080-01
COP: Shengyang Ni

Messung: GC-MS (HRMS)
Ionisierung: GC-EI
Spektrometer: Q Exactive GC Orbitrap
Säule: MS 63 ZB1HT
Länge: 30+3
Temp.: 35-15-300-3
GC-Nr.: -
ELNA-Nr.: 34760

Auswerter: Haupt (2243)

Mass to be matched (m/z): 164.108080 Charge: 1

Mass Tolerance: ±0.050000
Restriction of atom numbers:

C	H	18O
1-150	max 150	1-5

Number of calculated Formulas: 7

Formula	Diff.(ppm)	theor. m/z
C11 H14.18O1	0.48	164.108159
C8 H14.18O3	-9.77	164.106477
C5 H14.18O5	-20.02	164.104795
C6 H20.18O4	271.20	164.152586
C9 H20.18O2	281.45	164.154268
C10 H8.18O2	-290.74	164.060368
C7 H8.18O4	-300.99	164.058685

Suggestion:
C11H14[18]O1 MW: 164

^{13}C NMR of the ^{18}O-labelled compound

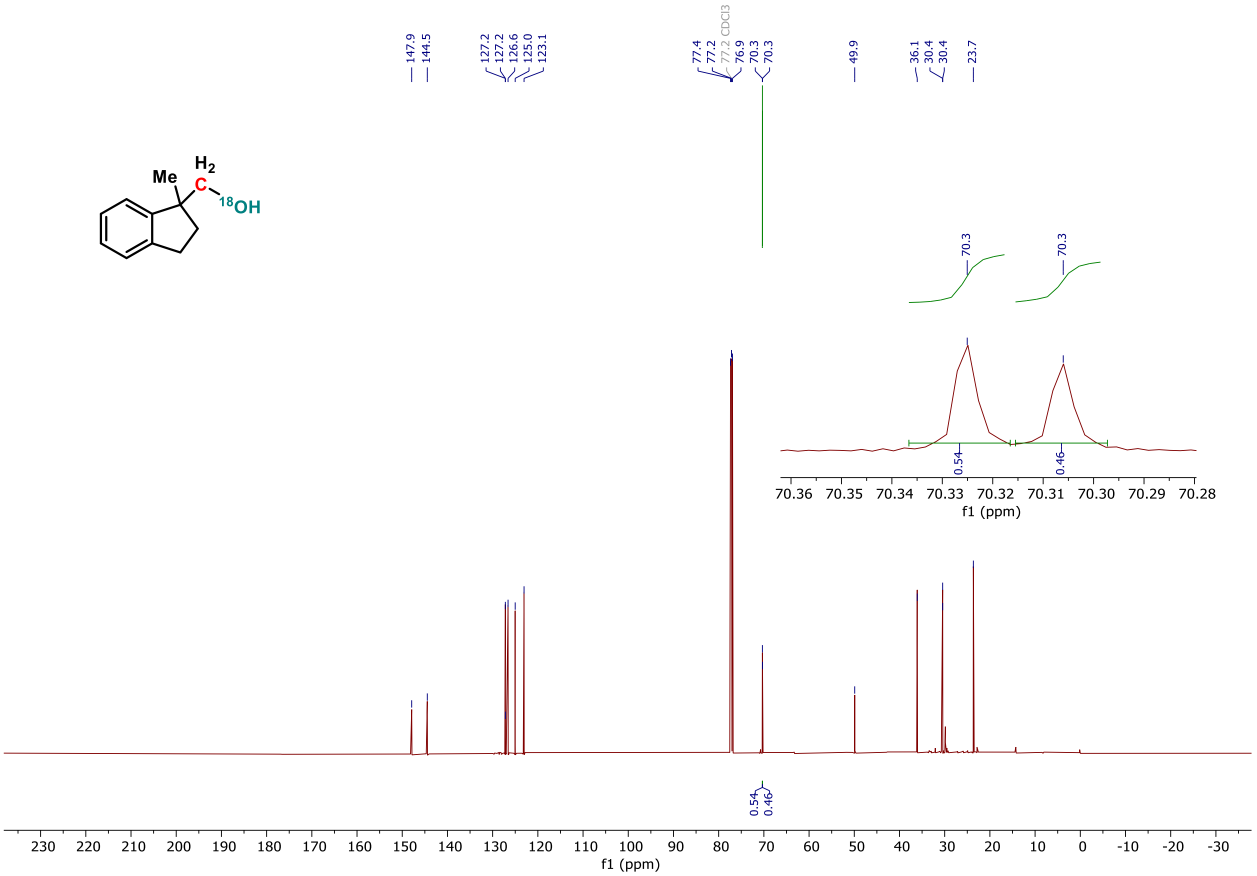

The CH$_2$OH group shows two ^{13}C peaks at around 70.3 ppm. These are the ^{13}C-^{16}O and the ^{13}C-^{18}O peaks with some isotopic shift differences. Their relative ratio is 54:46, which matches almost perfectly data from the MS.

Mechanism of N_2O formation

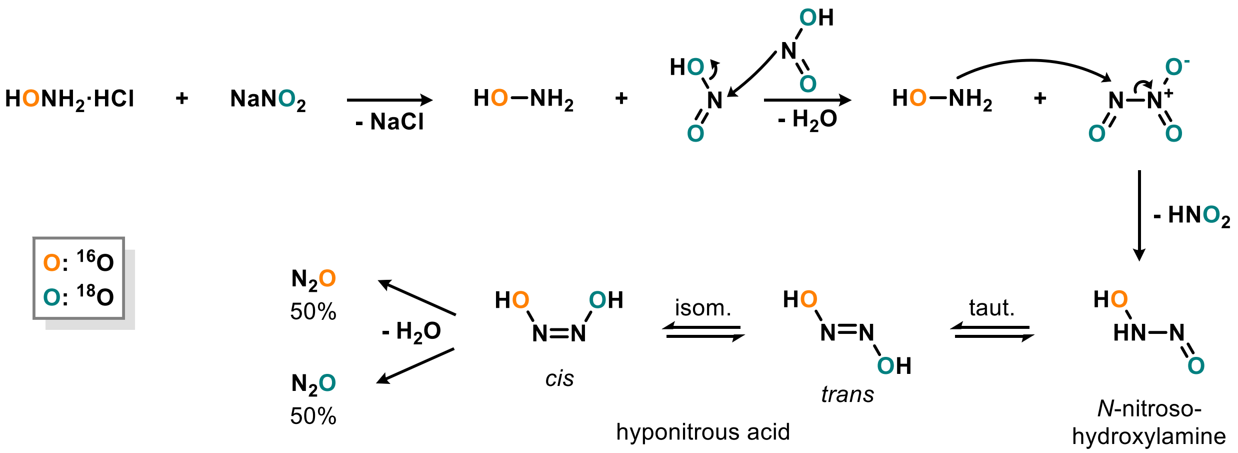

The mechanism of the formation of N_2O from hydroxylamine hydrochloride and sodium nitrite is reported in the literature as depicted above. Initially, nitrous acid is formed from the salt metathesis of the hydrochloride and the sodium nitrite. The anhydric form of nitrous acid N_2O_3 acts then as the nitrosation agent reacting with hydroxylamine. The thus formed *N*-nitrosohydroxylamine tautomerizes to hyponitrous acid, of which its *cis*-isomer decomposes to N_2O upon loss of a water molecule. Importantly, in the hypothetical reaction of unlabelled hydroxylamine hydrochloride with 100% ^{18}O-labelled sodium nitrite, N_2O would be formed from a symmetric intermediate *cis*-hyponitrous acid, containing both a labelled ^{18}O-atom and a non-labelled ^{16}O-atom. Since the dehydration could occur at both sites, the resulting N_2O gas would be ^{18}O-labelled only at 50 %. In the present experiment the employed sodium nitrite was 90% ^{18}O-labelled, therefore the thus formed N_2O gas was expected to be 45% ^{18}O-labelled.

12 Intermediacy of alkyl-Ni prior to O-insertion

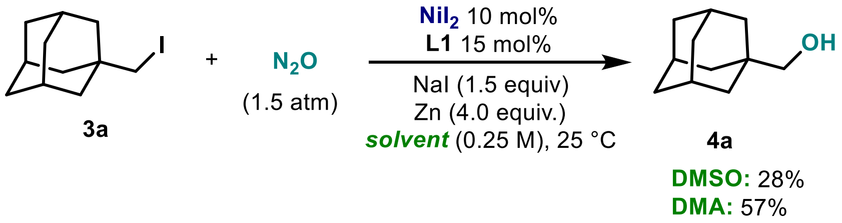

Note: without N_2O, **4a** couldn't be formed in the reaction, only the protodeiodinative product could be detected in 73% ^{1}H NMR yield (around 14% dimer).

13 Headspace analysis

General procedure of the reactions for the GC measurement of the headspace

To support the hypothesis that N_2O is the source of oxygen in the final products, we performed analysis of the headspace after the reaction of several substrates. The detection of significant amounts of N_2 would support the N_2O activation. Such analyses are easily achieved by gas chromatography using a thermal conductivity detector (GC-TCD), typically used for gas analysis. To facilitate the GC measurement, the reaction was conducted in a vial equipped with a screw septum cap following the standard condition in **General Procedure A**, and the pressure was reduced to 1.5 bar. This vial was found to be suitable and provided only slightly diminished yield for the optimized substrates.

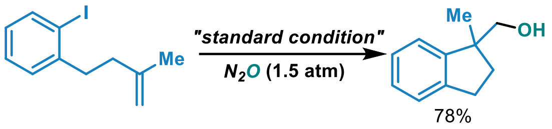

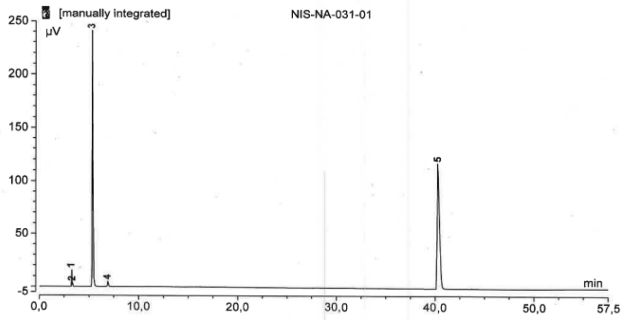

Sample:	**NIS-NA-031-01**	Instrument:	**GC_113**
Sequenz:	**8490 NIS-NA VD**	Measured:	**14.10.21 15:35**
Sequenz date:	**14.10.21**	Processing M.:	**LEF-LA**
		Report-File:	**031-01**

Zuordnung siehe PAY-PA-642-01 20/7477 und LEF-LA-442-01 20/7680

No.	Ret.Time min	area-% %	Peak Name
1	3,27	1,16	Argon (keine Basislinientrennung)
2	3,34	0,24	O2 (keine Basislinientrennung)
3	5,30	42,43	N2
4	6,89	0,82	
5	40,24	55,35	N2O

Instrument parameters:

Column:	27,7 m	HP-Plot 5A Molsieve 0,32/10,0df G/764
Temperature:	220/ 30, 10 min iso	6/min 250 12/min 320, 5 min iso/ 250
Gas:	0,50 bar	He
Sample size:	250,0 µL	

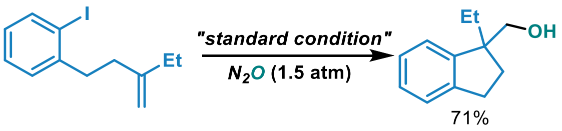

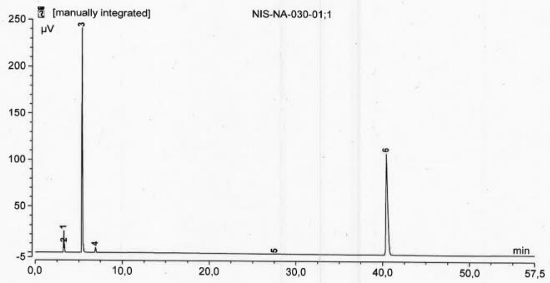

Sample:	NIS-NA-030-01;1	Instrument:	GC_113
Sequenz:	8489 NIS-NA VD	Measured:	15.10.21 15:32
Sequenz date:	14.10.21	Processing M.:	LEF-LA
		Report-File:	030-01

Zuordnung siehe PAY-PA-642-01 20/7477 und LEF-LA-442-01 20/7680

No.	Ret.Time min	area-% %	Peak Name
1	3,27	1,85	Argon (keine Basislinientrennung)
2	3,35	0,77	O2 (keine Basislinientrennung)
3	5,32	44,13	N2
4	6,90	0,92	
5	27,54	0,04	
6	40,39	52,29	N2O

Instrument parameters:

Column:	27,7 m	HP-Plot 5A Molsieve 0,32/10,0df G/764
Temperature:	220/ 30, 10 min iso 6/min 250 12/min 320, 5 min iso/ 250	
Gas:	0,50 bar	He
Sample size:	250,0 µL	

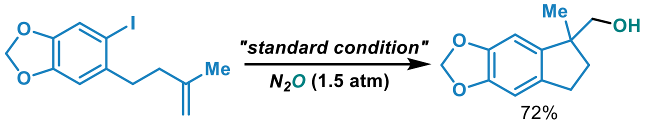

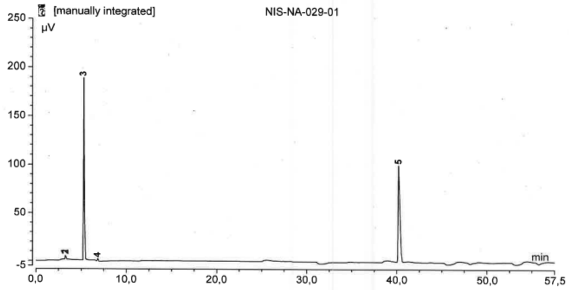

Sample:	NIS-NA-029-01	Instrument:	GC_113
Sequenz:	8488 NIS-NA VD	Measured:	14.10.21 11:12
Sequenz date:	14.10.21	Processing M.:	LEF-LA
		Report-File:	029-01

Zuordnung siehe PAY-PA-642-01 20/7477 und LEF-LA-442-01 20/7680

No.	Ret.Time min	area-% %	Peak Name
1	3,25	0,37	Argon (keine Basislinientrennung)
2	3,32	0,31	O2 (keine Basislinientrennung)
3	5,29	40,84	N2
4	6,84	0,60	
5	40,18	57,87	N2O

Instrument parameters:

Column:	27,7 m	HP-Plot 5A Molsieve 0,32/10,0df G/764
Temperature:	220/ 30, 10 min iso 6/min 250 12/min 320, 5 min iso/ 250	
Gas:	0,50 bar	He
Sample size:	250,0 µL	

Control experiment 1 (without addition of the substrate) :

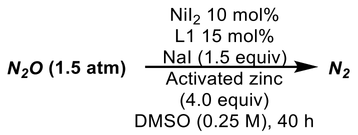

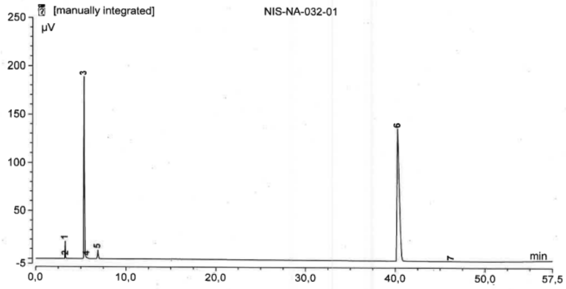

Sample:	**NIS-NA-032-01**	Instrument:	**GC_113**
Sequenz:	**8491 NIS-NA VD**	Measured:	**15.10.21 08:11**
Sequenz date:	**15.10.21**	Processing M.:	**LEF-LA**
		Report-File:	**032-01**

Zuordnung siehe PAY-PA-642-01 20/7477 und LEF-LA-442-01 20/7680

No.	Ret.Time min	area-% %	Peak Name
1	3,26	1,31	Argon (keine Basislinientrennung)
2	3,33	0,11	O2 (keine Basislinientrennung)
3	5,32	28,95	N2
4	5,66	0,40	
5	6,88	1,38	
6	40,23	67,77	N2O
7	46,20	0,08	

Instrument parameters:

Column:	27,7 m	HP-Plot 5A Molsieve 0,32/10,0df G/764
Temperature:	220/ 30, 10 min iso 6/min 250 12/min 320, 5 min iso/ 250	
Gas:	0,50 bar	He
Sample size:	250,0 µL	

Control experiment 2 (without addition of the nickel catalyst and ligand) :

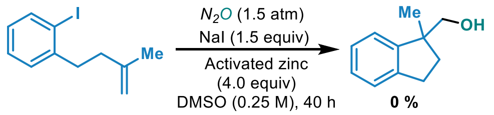

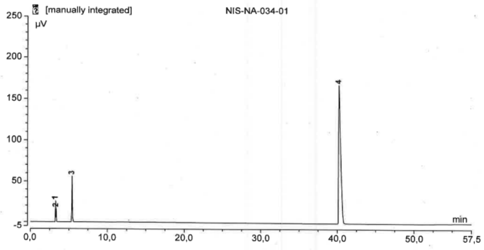

Sample:	**NIS-NA-034-01**	Instrument:	**GC_113**
Sequenz:	**8493 NIS-NA VD**	Measured:	**15.10.21 11:52**
Sequenz date:	**15.10.21**	Processing M.:	**LEF-LA**
		Report-File:	**034-01**

Zuordnung siehe PAY-PA-642-01 20/7477 und LEF-LA-442-01 20/7680

No.	Ret.Time min	area-% %	Peak Name
1	3,27	1,77	Argon (keine Basislinientrennung)
2	3,34	1,21	O2 (keine Basislinientrennung)
3	5,40	6,80	N2
4	40,18	90,21	N2O

Instrument parameters:

Column:	27,7 m	HP-Plot 5A Molsieve 0,32/10,0df G/764
Temperature:	220/ 30, 10 min iso 6/min 250 12/min 320, 5 min iso/ 250	
Gas:	0,50 bar	He
Sample size:	250,0 µL	

Control experiment 3 (only the zinc and NaI) :

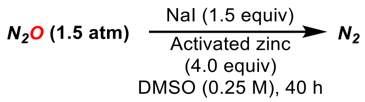

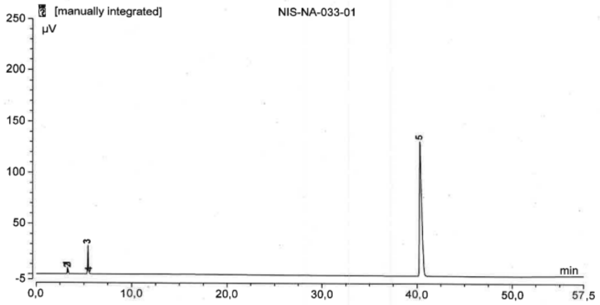

Sample:	NIS-NA-033-01	Instrument:	GC_113
Sequenz:	8492 NIS-NA VD	Measured:	15.10.21 10:10
Sequenz date:	15.10.21	Processing M.:	LEF-LA
		Report-File:	033-01

Zuordnung siehe PAY-PA-642-01 20/7477 und LEF-LA-442-01 20/7680

No.	Ret.Time min	area-% %	Peak Name
1	3,27	0,68	Argon (keine Basislinientrennung)
2	3,34	0,50	O2 (keine Basislinientrennung)
3	5,41	5,21	N2
4	5,57	0,00	
5	40,28	93,62	N2O

Instrument parameters:

Column:	27,7 m	HP-Plot 5A Molsieve 0,32/10,0df G/764
Temperature:	220/ 30, 10 min iso 6/min 250 12/min 320, 5 min iso/ 250	
Gas:	0,50 bar	He
Sample size:	250,0 μL	

Control experiment 4 (pure N_2O) :

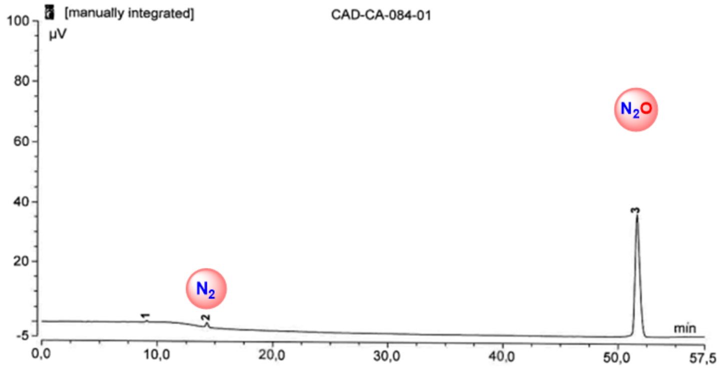

Sample:	CAD-CA-084-01	Instrument:	GC_311
Sequenz:	8134 CAD-CA VD	Measured:	22.03.21 13:01
Sequenz date:	22.03.21	Processing M.:	CAD-CA
		Report-File:	084-01

N2O
Zuordnung siehe PAY-PA-642-01 20/7477 und LEF-LA-442-01 20/7680

No.	Ret.Time min	area-% %	Peak Name
1	9,09	0,45	O2
2	14,29	1,84	N2
3	51,62	97,71	N2O

14 HPLC Spectra

Compound (S)-2w

Gerät : NP-1

Operator : Be
Sample Name : NIS-NA-005-01
Vial # : 11
Injection Volume : 2 uL
Data File Name : NIS-NA-005-01-05.lcd
Method File Name : Ni.lcm

Data Acquired: 5/28/2021 12:41:45 PM

2.0 µL NIS-NA-005-01; Racemat (10 mg in 2 mL i-Propanol)
davon 50/150 µL n-Heptan
150 mm Chiralpak IC-3, 4.6 mm i.D.
n-Heptan / i-Propanol = 98:2
1.0 mL / min, 9.2 MPa, 298 K
UV, 220 nm

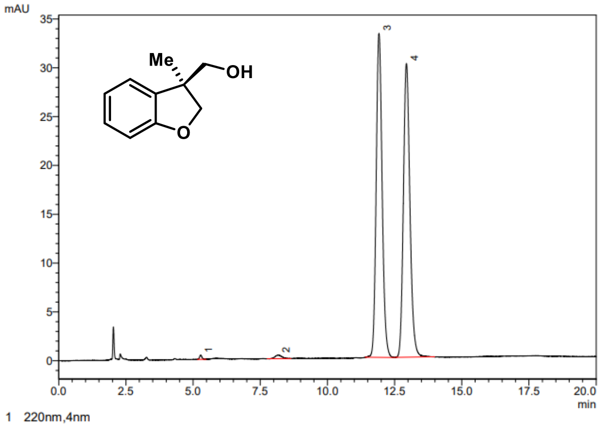

1 220nm,4nm

PDA Ch1 220nm

Peak #	Ret. Time	Area %	Name
1	5.28	0.25	
2	8.17	0.63	
3	11.92	49.55	1.Enantiomer
4	12.94	49.57	2. Enantiomer
Total		100.00	

Gerät : NP-1

Operator : Be
Sample Name : NIS-NA-15-3
Vial # : 3
Injection Volume : 2 uL
Data File Name : NIS-NA-15-3-01.lcd
Method File Name : Ni.lcm

Data Acquired: 5/28/2021 11:58:35 AM

2.0 µL NIS-NA-15-3; chirale Probe (GS in 1 mL i-Propanol)
davon 50/150 µL n-Heptan
150 mm Chiralpak IC-3, 4.6 mm i.D.
n-Heptan / i-Propanol = 98:2
1.0 mL / min, 9.1 MPa, 298 K
UV, 220 nm

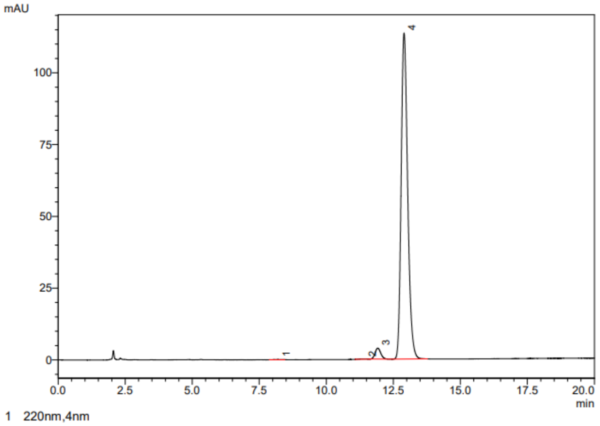

1 220nm,4nm

PDA Ch1 220nm

Peak #	Ret. Time	Area %	Name
1	8.20	0.05	
2	11.42	0.09	
3	11.92	2.89	1.Enantiomer
4	12.90	96.97	2. Enantiomer
Total		100.00	

ee = 94.2 %

Data1 : W:\Workgroup_Geräte\NP-1\Ni\NIS-NA-15-1-01.lcd
Data2 : W:\Workgroup_Geräte\NP-1\Ni\NIS-NA-15-2-01.lcd
Data3 : W:\Workgroup_Geräte\NP-1\Ni\NIS-NA-15-4-01.lcd
Data4 : W:\Workgroup_Geräte\NP-1\Ni\NIS-NA-15-3-01.lcd
Data5 :* W:\Workgroup_Geräte\NP-1\Ni\NIS-NA-005-01-05.lcd

150 mm Chiralpak IC-3, 4.6 mm i.D.,
n-Heptan / i-Propanol = 98:2
1.0 mL / min, 298 K, UV, 220 nm

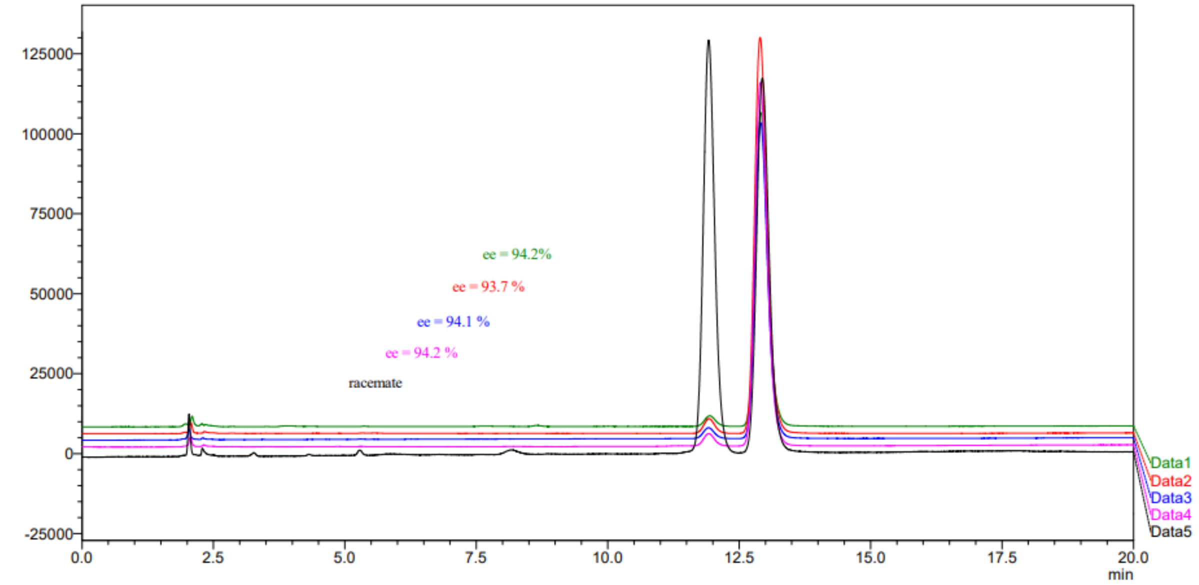

Compound (S)-2ab

Gerät	: NP-1
Operator	: Be
Sample Name	: NIS-NA-072-Rac
Vial #	: 1
Injection Volume	: 1 uL
Data File Name	: NIS-NA-072-Rac-IC-iProp-03.lcd
Method File Name	: Ni.lcm

Data Acquired: 12/2/2021 1:51:23 PM

1.0 µL NIS-NA-072-Rac; Racemat (GS in 200 µL i-Propanol)
davon 50/150 µL n-Heptan
150 mm Chiralpak IC-3, 4.6 mm i.D.,
n-Heptan / i-Propanol = 92:8
1.0 mL / min, 9.7 MPa, 298 K
UV, 220 nm

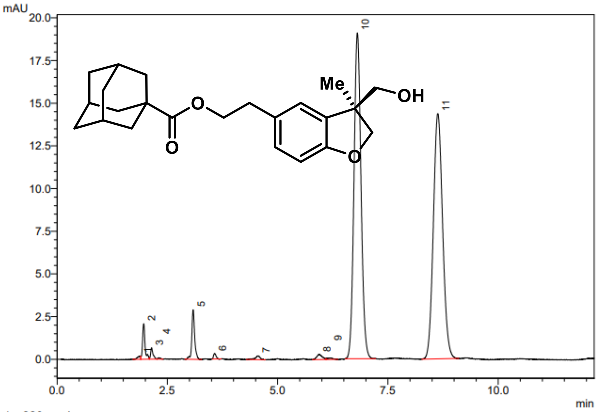

1 220nm,4nm

PDA Ch1 220nm

Peak #	Ret. Time	Area %	Name
1	1.88	0.20	
2	1.96	1.79	
3	2.14	0.56	
4	2.30	0.07	
5	3.08	2.89	
6	3.57	0.27	
7	4.56	0.39	
8	5.93	0.57	
9	6.18	0.16	
10	6.81	46.70	1. Enantiomer
11	8.63	46.40	2. Enantiomer
Total		100.00	

Gerät	: NP-1
Operator	: Be
Sample Name	: NIS-NA-072-01
Vial #	: 2
Injection Volume	: 1 uL
Data File Name	: NIS-NA-072-01-IC-iProp-01.lcd
Method File Name	: Ni.lcm

Data Acquired: 12/2/2021 2:05:06 PM

1.0 µL NIS-NA-072-01; chirale Probe (GS in 200 µL i-Propanol)
davon 50/150 µL n-Heptan
150 mm Chiralpak IC-3, 4.6 mm i.D.,
n-Heptan / i-Propanol = 92:8
1.0 mL / min, 9.5 MPa, 298 K
UV, 220 nm

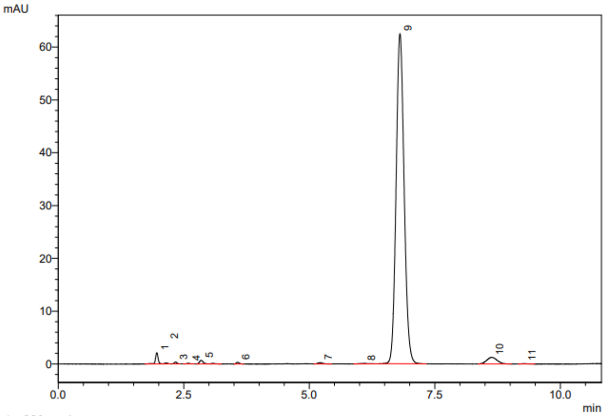

1 220nm,4nm

PDA Ch1 220nm

Peak #	Ret. Time	Area %	Name
1	1.96	1.05	
2	2.15	0.10	
3	2.34	0.16	
4	2.59	0.04	
5	2.85	0.58	
6	3.57	0.19	
7	5.22	0.21	
8	6.07	0.12	
9	6.81	95.03	1. Enantiomer
10	8.63	2.46	2. Enantiomer
11	9.28	0.06	
Total		100.00	

ee = 94.9 %

Data1 :* W:\Workgroup_Geräte\NP-1\Ni\NIS-NA-072-01-IC-iProp-01.lcd
Data2 :* W:\Workgroup_Geräte\NP-1\Ni\NIS-NA-072-Rac-IC-iProp-03.lcd

150 mm Chiralpak IC-3, 4.6 mm i.D.,
n-Heptan / i-Propanol = 92:8
1.0 mL / min, 298 K, UV, 220 nm

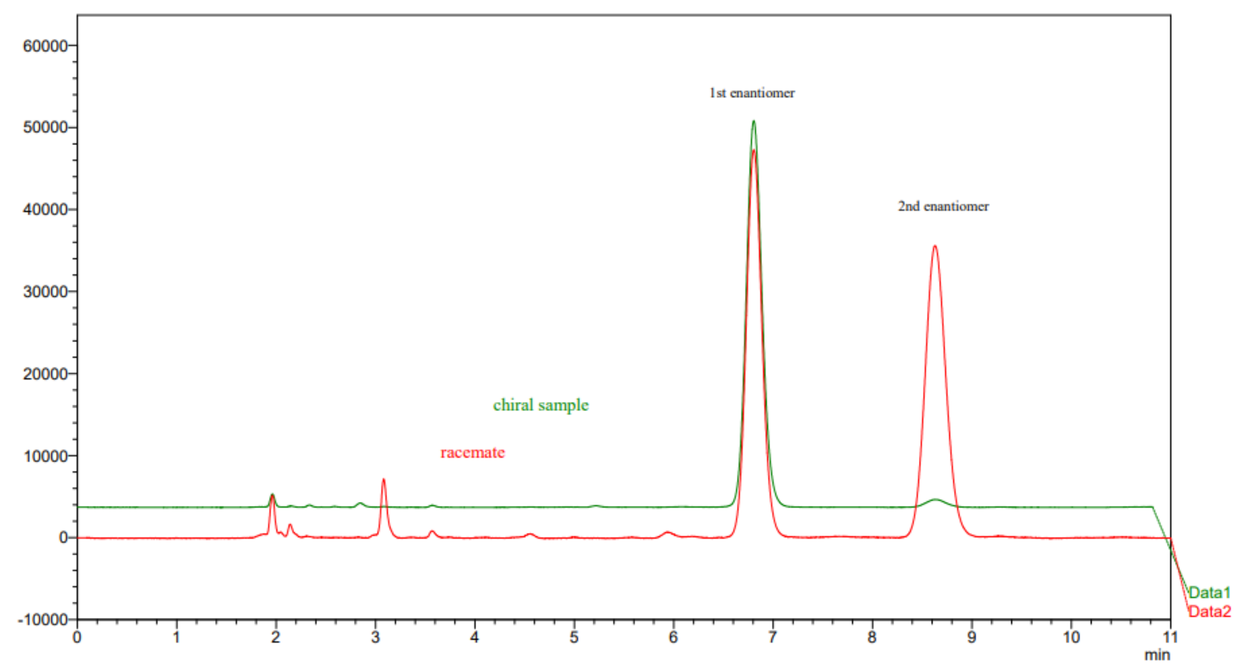

Compound (S)-2ac

Operator : Ri
Sample Name : NIS-NA-049-01
Vial # : 4
Injection Volume : 3 uL
Data File Name : NIS-NA-049-01-R-01.lcd
Method File Name : Ni.lcm

Data Acquired: 11/22/2021 2:24:26 PM

3 µL NIS-NA-049-01 Racemat
(in 200 µL 2-Propanol; 50 µL Lösung in 150 µL n-Heptan)
150 mm Chiralpak IC-3, 4.6 mm i.D.
n-Heptan/ 2-Propanol = 95:5
1.0 mL/min, 9.3 MPa, 298 K
UV, 220 nm

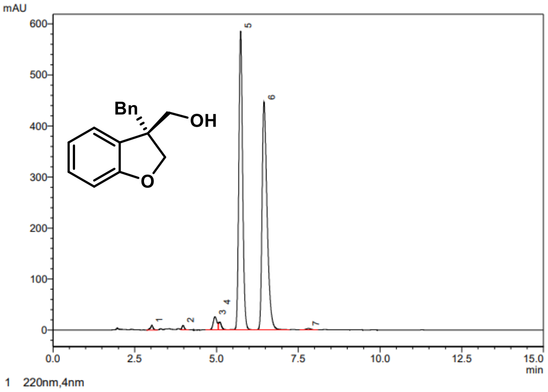

1 220nm,4nm

PDA Ch1 220nm

Peak #	Ret. Time	Area %	Name
1	3.02	0.53	
2	3.98	0.46	
3	4.95	2.12	
4	5.10	0.98	
5	5.74	47.62	1. Enantiomer
6	6.45	47.99	2. Enantiomer
7	7.81	0.29	
Total		100.00	

Operator : Ri
Sample Name : NIS-NA-049-01
Vial # : 14
Injection Volume : 3 uL
Data File Name : NIS-NA-049-01-01.lcd
Method File Name : Ni.lcm

Data Acquired: 11/22/2021 3:03:30 PM

3 µL NIS-NA-049-01 Probe
(in 200 µL 2-Propanol; 50 µL Lösung in 150 µL n-Heptan)
150 mm Chiralpak IC-3, 4.6 mm i.D.
n-Heptan/ 2-Propanol = 95:5
1.0 mL/min, 9.3 MPa, 298 K
UV, 220 nm

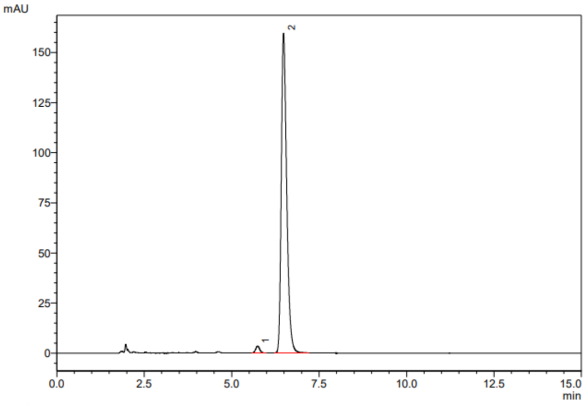

1 220nm,4nm

PDA Ch1 220nm

Peak #	Ret. Time	Area %	Name
1	5.74	1.57	1. Enantiomer
2	6.48	98.43	2. Enantiomer
Total		100.00	

ee= 96,9%

Chromatogram
Data1 : W:\Workgroup_Geräte\NP-1\Ni\NIS-NA-049-01-R-01.lcd racemate
Data2 :* W:\Workgroup_Geräte\NP-1\Ni\NIS-NA-049-01-01.lcd ee-sample

150 mm Chiralpak IC-3, 4.6 mm i.D.
n-Heptan/ 2-Propanol = 95:5
1 ml/min, 298 K, UV 220nm

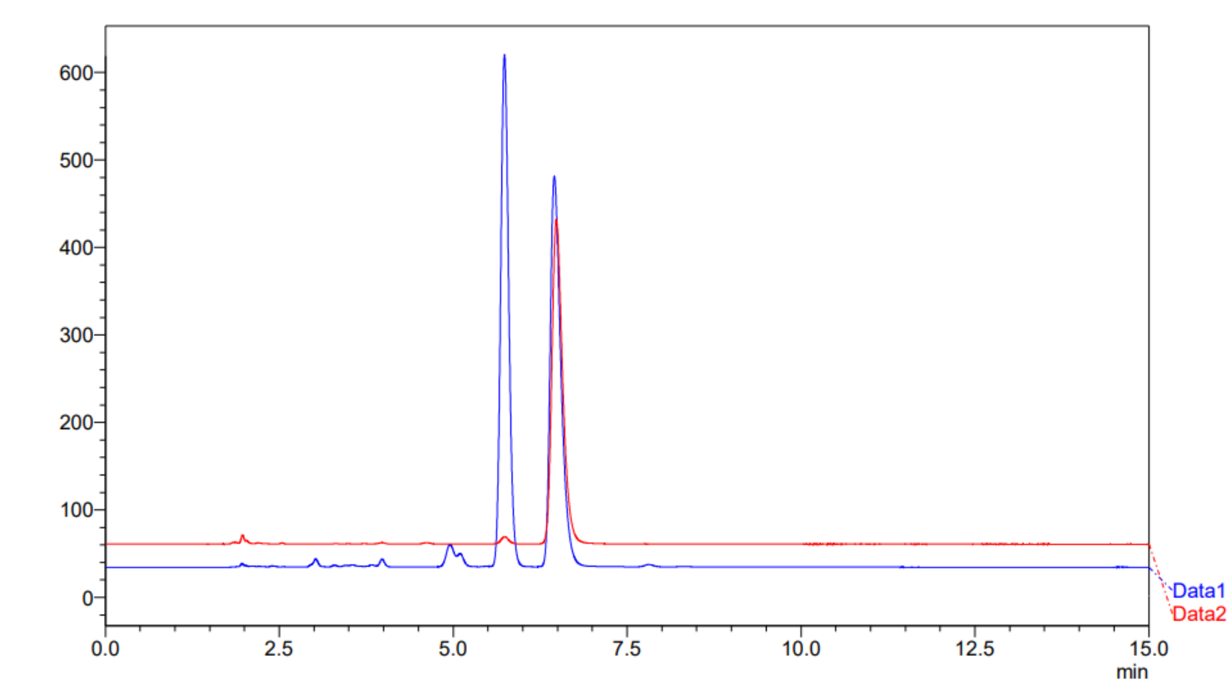

Compound (S)-2ad

Gerät : NP-1

Operator : Ri
Sample Name : NIS-NA-042-01
Vial # : 2
Injection Volume : 3 uL
Data File Name : NIS-NA-042-01-R-01.lcd
Method File Name : Ni.lcm

Data Acquired: 11/22/2021 9:01:47 AM

3 µL NIS-NA-042-01 Racemat
(in 200 µL 2-Propanol; 50 µL Lösung in 150 µL n-Heptan)
150 mm Chiralpak IC-3, 4.6 mm i.D.
n-Heptan/ 2-Propanol = 95:5
1.0 mL/min, 9.3 MPa, 298 K
UV, 220 nm

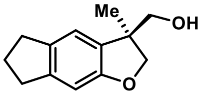

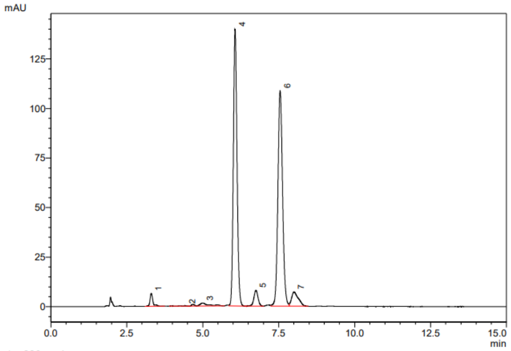

1 220nm,4nm

PDA Ch1 220nm

Peak #	Ret. Time	Area %	Name
1	3.31	1.67	
2	4.43	0.14	
3	5.00	1.47	
4	6.06	44.91	1. Enantiomer
5	6.75	2.93	
6	7.55	44.59	2. Enantiomer
7	8.01	4.29	
Total		100.00	

Gerät : NP-1

Operator : Ri
Sample Name : NIS-NA-042-01
Vial # : 12
Injection Volume : 3 uL
Data File Name : NIS-NA-042-01-01.lcd
Method File Name : Ni.lcm

Data Acquired: 11/22/2021 3:21:59 PM

3 µL NIS-NA-042-01 Probe
(in 200 µL 2-Propanol; 50 µL Lösung in 150 µL n-Heptan)
150 mm Chiralpak IC-3, 4.6 mm i.D.
n-Heptan/ 2-Propanol = 95:5
1.0 mL/min, 9.3 MPa, 298 K
UV, 220 nm

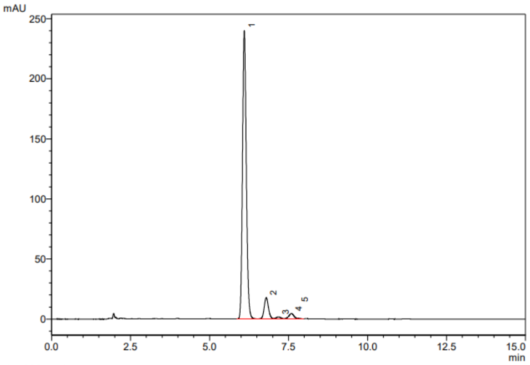

1 220nm,4nm

PDA Ch1 220nm

Peak #	Ret. Time	Area %	Name
1	6.10	89.42	1. Enantiomer
2	6.79	7.46	
3	7.18	0.77	
4	7.59	2.22	2. Enantiomer
5	7.78	0.13	
Total		100.00	

ee = 95.2%

Chromatogram
Data1 :* W:\Workgroup_Geräte\NP-1\Ni\NIS-NA-042-01-R-01.lcd racemate
Data2 :* W:\Workgroup_Geräte\NP-1\Ni\NIS-NA-042-01-01.lcd ee-sample

150 mm Chiralpak IC-3, 4.6 mm i.D.
n-Heptan/ 2-Propanol = 95:5
1 ml/min, 298 K, UV 220nm

mV

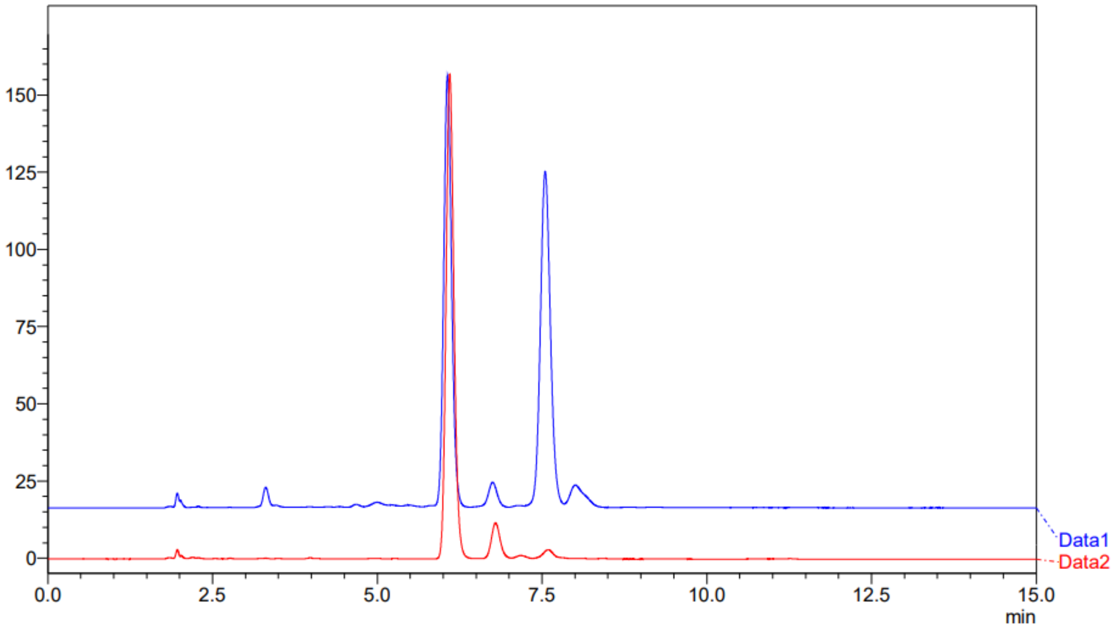

Compound (S)-2ae

Gerät : NP-1

Operator : Ke
Sample Name : NIS-NA-041-rac
Vial # : 11
Injection Volume : 3 uL
Data File Name : NIS-NA-041-rac-IB-N3-07.lcd
Method File Name : Ni.lcm

Data Acquired: 11/23/2021 1:45:26 PM

3 µL NIS-NA-041-rac (GS in 200 µL 2-Propanol)
50 µL in 150 µL n-Heptan
150 mm Chiralpak IB-N3, 4.6 mm i.D.
n-Heptan/2-Propanol = 98:2
1.0 mL / min, 8.7 MPa, 298 K
220 nm

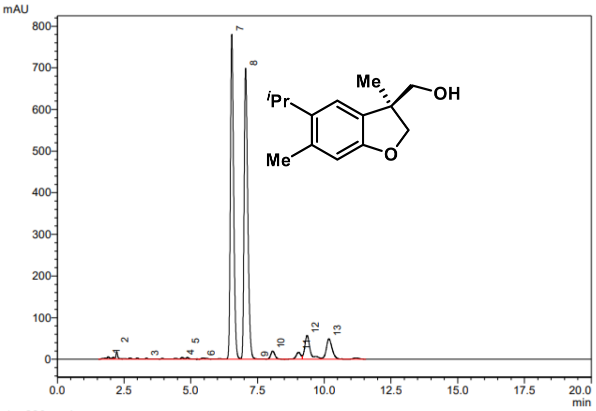

1 220nm,4nm

PDA Ch1 220nm

Peak #	Ret. Time	Area %	Name
1	1.90	0.55	
2	2.22	0.83	
3	3.34	0.49	
4	4.68	0.30	
5	4.87	0.25	
6	5.46	0.39	
7	6.54	42.30	1. Enantiomer
8	7.05	42.20	2. Enantiomer
9	7.46	0.09	
10	8.07	1.34	
11	9.04	1.27	
12	9.36	5.02	
13	10.17	4.96	
Total		100.00	

Gerät : NP-1

Operator : Ke
Sample Name : NIS-NA-041-01
Vial # : 21
Injection Volume : 3 uL
Data File Name : NIS-NA-041-01-IB-N3-03.lcd
Method File Name : Ni.lcm

Data Acquired: 11/23/2021 2:07:56 PM

3 µL NIS-NA-041-01 (GS in 200 µL 2-Propanol)
50 µL in 150 µL n-Heptan
150 mm Chiralpak IB-N3, 4.6 mm i.D.
n-Heptan/2-Propanol = 98:2
1.0 mL / min, 8.7 MPa, 298 K
220 nm

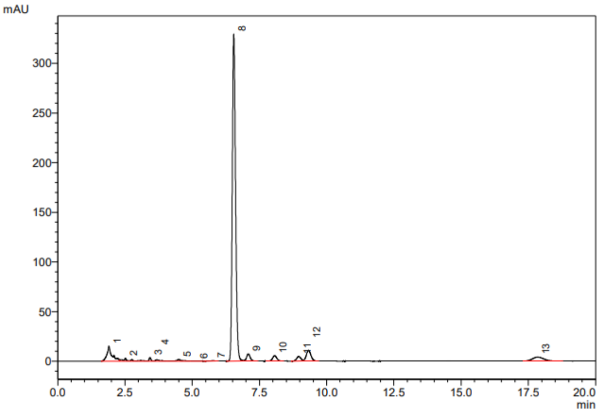

1 220nm,4nm

PDA Ch1 220nm

Peak #	Ret. Time	Area %	Name	
1	1.90	5.58		
2	2.51	1.02		
3	3.43	0.82		
4	3.69	0.48		
5	4.50	0.66		
6	5.13	0.12		
7	5.78	0.10		
8	6.55	79.22	1. Enantiomer	ee = 95.2%
9	7.09	1.93	2. Enantiomer	
10	8.07	1.66		
11	8.96	1.50		
12	9.33	3.62		
13	17.85	3.28		
Total		100.00		

Data1 : W:\Workgroup_Geräte\NP-1\Ni\NIS-NA-041-rac-IB-N3-07.lcd racemate
Data2 : W:\Workgroup_Geräte\NP-1\Ni\NIS-NA-041-01-IB-N3-03.lcd ee-sample

150 mm Chiralpak IB-N3, 4.6 mm i.D.
n-Heptan/2-Propanol = 98:2
1.0 mL/min, 298 K, 220 nm

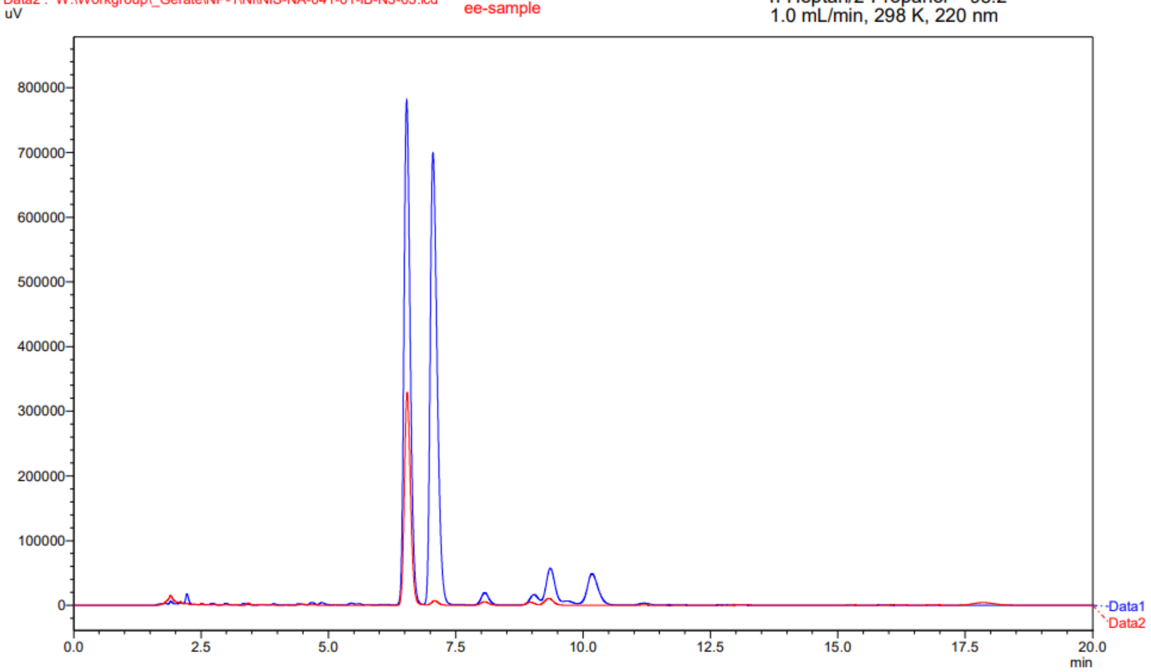

Compound (S)-2x

Gerät : NP-1

Operator : Be
Sample Name : NIS-NA-045-R
Vial # : 1
Injection Volume : 2 uL
Data File Name : NIS-NA-045-R-03.lcd
Method File Name : Ni.lcm

Data Acquired: 11/17/2021 2:53:00 PM

2.0 µL NIS-NA-045-R; Racemat (GS in 0.5 mL i-Propanol)
davon 50/150 µL n-Heptan
150 mm Chiralpak IC-3, 4.6 mm i.D.,
n-Heptan / i-Propanol = 95:5
1.0 mL / min, 9.3 MPa, 298 K
UV, 220 nm

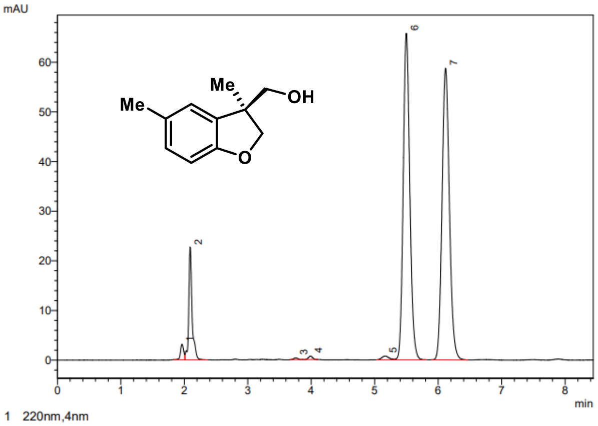

1 220nm,4nm

PDA Ch1 220nm

Peak #	Ret. Time	Area %	Name
1	1.96	1.02	
2	2.09	7.89	
3	3.75	0.15	
4	3.99	0.30	
5	5.16	0.57	
6	5.50	45.01	1.Enantiomer
7	6.11	45.06	2. Enantiomer
Total		100.00	

Gerät : NP-1

Operator : Be
Sample Name : NIS-NA-045-01
Vial # : 2
Injection Volume : 2 uL
Data File Name : NIS-NA-045-01-02.lcd
Method File Name : Ni.lcm

Data Acquired: 11/17/2021 3:02:49 PM

2.0 µL NIS-NA-045-01; chirale Probe (GS in 0.5 mL i-Propanol)
davon 50/150 µL n-Heptan
150 mm Chiralpak IC-3, 4.6 mm i.D.,
n-Heptan / i-Propanol = 95:5
1.0 mL / min, 9.3 MPa, 298 K
UV, 220 nm

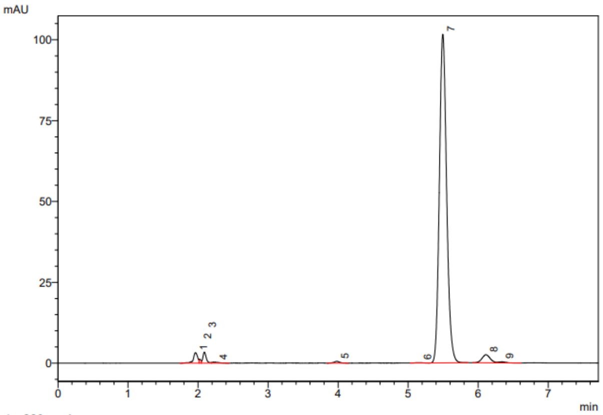

1 220nm,4nm

PDA Ch1 220nm

Peak #	Ret. Time	Area %	Name
1	1.96	1.72	
2	2.02	0.24	
3	2.09	1.41	
4	2.25	0.21	
5	3.98	0.38	
6	5.16	0.11	
7	5.49	93.04	1.Enantiomer
8	6.11	2.56	2. Enantiomer
9	6.33	0.33	
Total		100.00	

ee = 94.6 %

Data1 :* W:\Workgroup_Geräte\NP-1\Ni\NIS-NA-045-R-03.lcd
Data2 :* W:\Workgroup_Geräte\NP-1\Ni\NIS-NA-045-01-02.lcd
uV

150 mm Chiralpak IC-3, 4.6 mm i.D.,
n-Heptan / i-Propanol = 95:5
1.0 mL / min, 298 K, UV, 220 nm

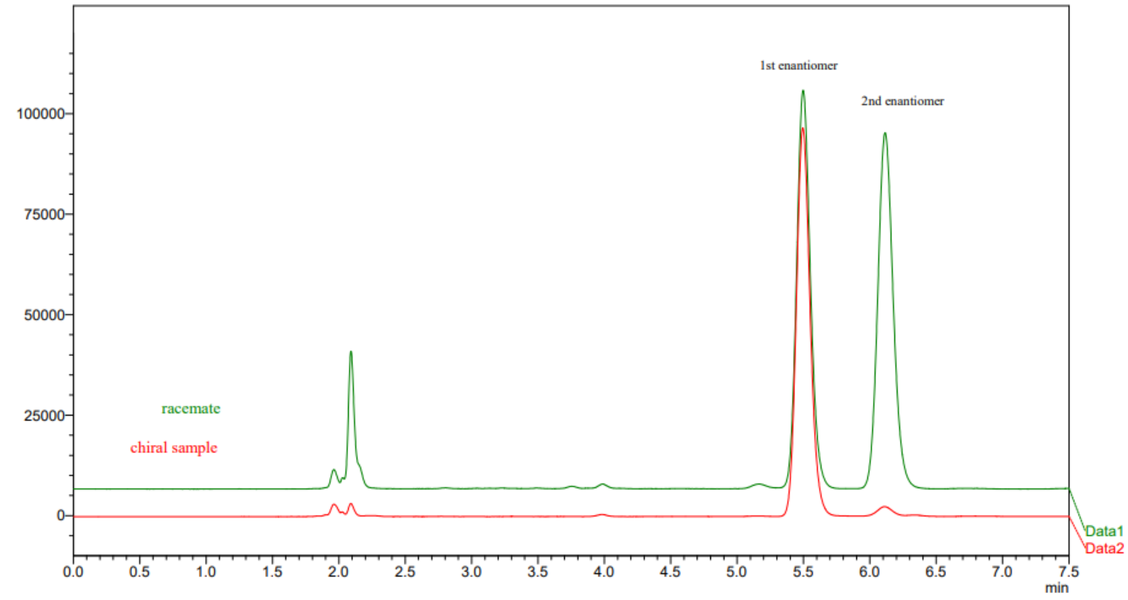

Compound (S)-2aa

Gerät : NP-1

Operator : St
Sample Name : NIS-NA-037-R
Vial # : 1
Injection Volume : 5 uL
Data File Name : NIS-NA-037-R-Prop-IC-05.lcd
Method File Name : Ni.lcm

Data Acquired: 11/4/2021 10:13:27 AM

5 µL NIS-NA-037-R
1µL in 1mL n-Heptan
150 mm Chiralpak IC-3, 4.6 mm i.D.
n-Heptan/2-Propanol = 95:5
1.0 mL/min, 9.3 MPa, 298 K
UV, 220 nm

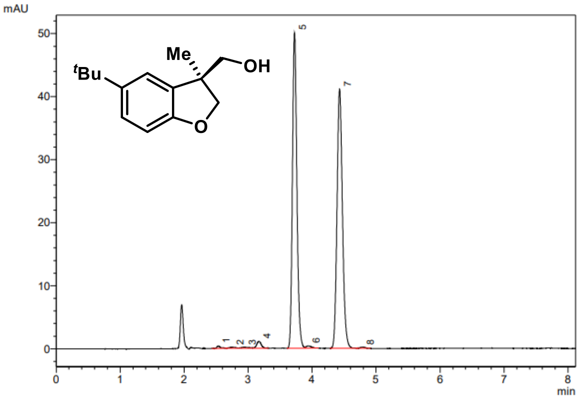

1 220nm,4nm

PDA Ch1 220nm

Peak #	Ret. Time	Area %	Name
1	2.53	0.32	
2	2.75	0.24	
3	2.94	0.33	
4	3.17	1.08	
5	3.72	48.61	1. enantiomer
6	3.94	0.39	
7	4.43	48.81	2. enantiomer
8	4.79	0.22	
Total		100.00	

Gerät : NP-1

Operator : St
Sample Name : NIS-NA-037-01
Vial # : 4
Injection Volume : 5 uL
Data File Name : NIS-NA-037-01-01.lcd
Method File Name : Ni.lcm

Data Acquired: 11/4/2021 10:22:28 AM

5 µL NIS-NA-037-01
1µL in 1mL n-Heptan
150 mm Chiralpak IC-3, 4.6 mm i.D.
n-Heptan/2-Propanol = 95:5
1.0 mL/min, 9.3 MPa, 298 K
UV, 220 nm

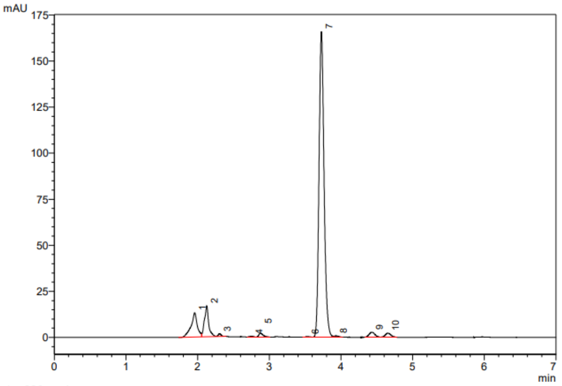

1 220nm,4nm

PDA Ch1 220nm

Peak #	Ret. Time	Area %	Name
1	1.96	7.93	
2	2.13	6.83	
3	2.31	0.37	
4	2.75	0.14	
5	2.88	0.84	
6	3.53	0.14	
7	3.73	80.72	1. enantiomer
8	3.93	0.27	
9	4.43	1.54	2. enantiomer
10	4.65	1.22	
Total		100.00	

ee = 96.3%

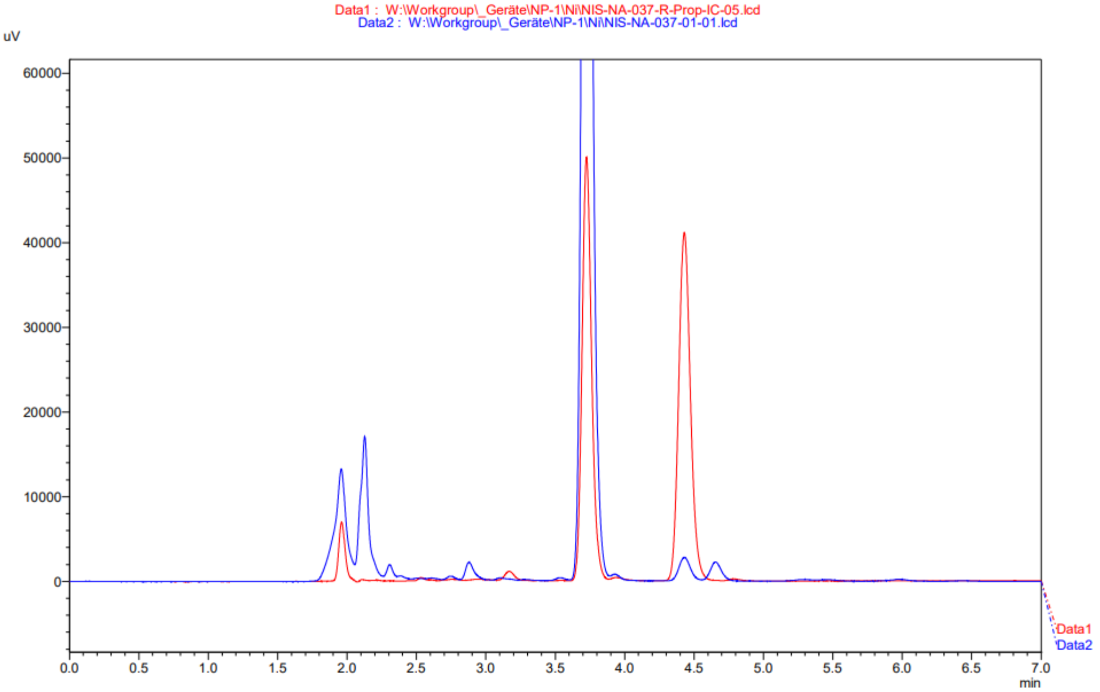

Compound (S)-2a

Gerät : NP-2

Operator : Hi
Sample Name : NIS-NA-021-Rac
Vial # : 1
Injection Volume : 1 uL
Data File Name : NIS-NA-021-Rac-IC-06.lcd
Method File Name : Ni.lcm

Data Acquired: 9/23/2021 3:16:38 PM

Säulenauswahlventil
<<Oven>>
Valve 2/R Position : 0

1 μ NIS-NA-021-Rac (in n-Heptamn/2-Propanol 1:1)
150 mm Chiralpak IC-3, 4.6 mm i.D.
n-Heptan : 2-Propanol = 98:2
1.0 ml/min, 7.1 MPa, 298 K
UV 220 nm

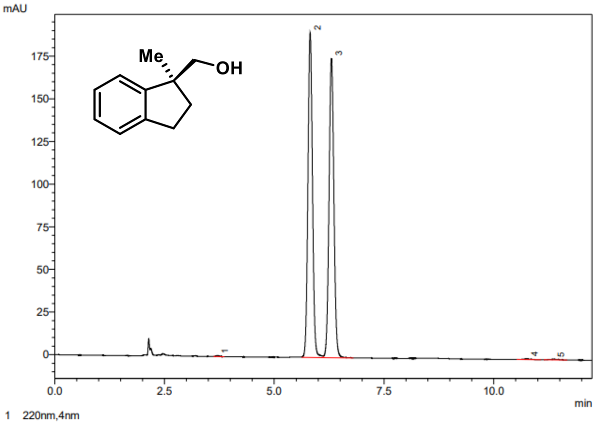

1 220nm,4nm

PDA Ch1 220nm

Peak #	Ret. Time	Area %	Name
1	3.71	0.11	
2	5.82	49.78	1st enantiomer
3	6.30	49.70	2nd enantiomer
4	10.76	0.21	
5	11.36	0.20	
Total		100.00	

Gerät : NP-2

Operator : Hi
Sample Name : NIS-NA-021-01
Vial # : 2
Injection Volume : 1 uL
Data File Name : NIS-NA-021-01-IC-04.lcd
Method File Name : Ni.lcm

Data Acquired: 9/23/2021 2:54:22 PM

Säulenauswahlventil
<<Oven>>
Valve 2/R Position : 0

1 μ NIS-NA-021-01 (in n-Heptan/2-Propanol 1:1)
150 mm Chiralpak IC-3, 4.6 mm i.D.
n-Heptan : 2-Propanol = 98:2
1.0 ml/min, 7.1 MPa, 298 K
UV 220 nm

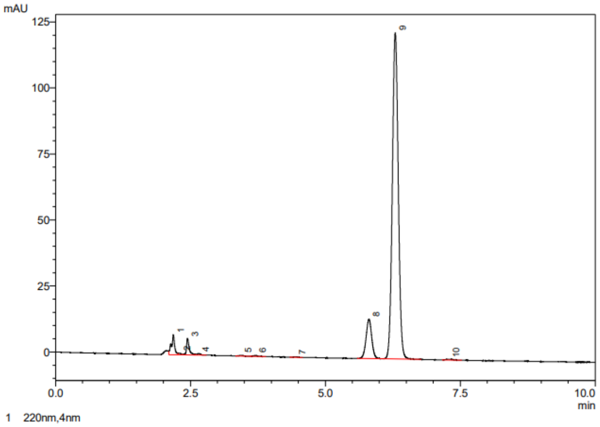

1 220nm,4nm

PDA Ch1 220nm

Peak #	Ret. Time	Area %	Name
1	2.18	2.80	
2	2.30	0.18	
3	2.45	1.96	
4	2.65	0.27	
5	3.43	0.15	
6	3.70	0.17	
7	4.44	0.11	
8	5.81	9.68	1st enantiomer
9	6.30	84.47	2nd enantiomer
10	7.29	0.22	
Total		100.00	

= 79.4 % ee

Data1 : W:\Workgroup_Geräte\NP-2\Ni\NIS-NA-021-01-IC-04.lcd
Data2 :* W:\Workgroup_Geräte\NP-2\Ni\NIS-NA-021-Rac-IC-06.lcd

150 mm Chiralpak IC-3, 4.6 mm i.D.
n-Heptan:2-Propanol = 98:2
1.0 ml/min, 7.1 MPa, 298 K, 220 nm

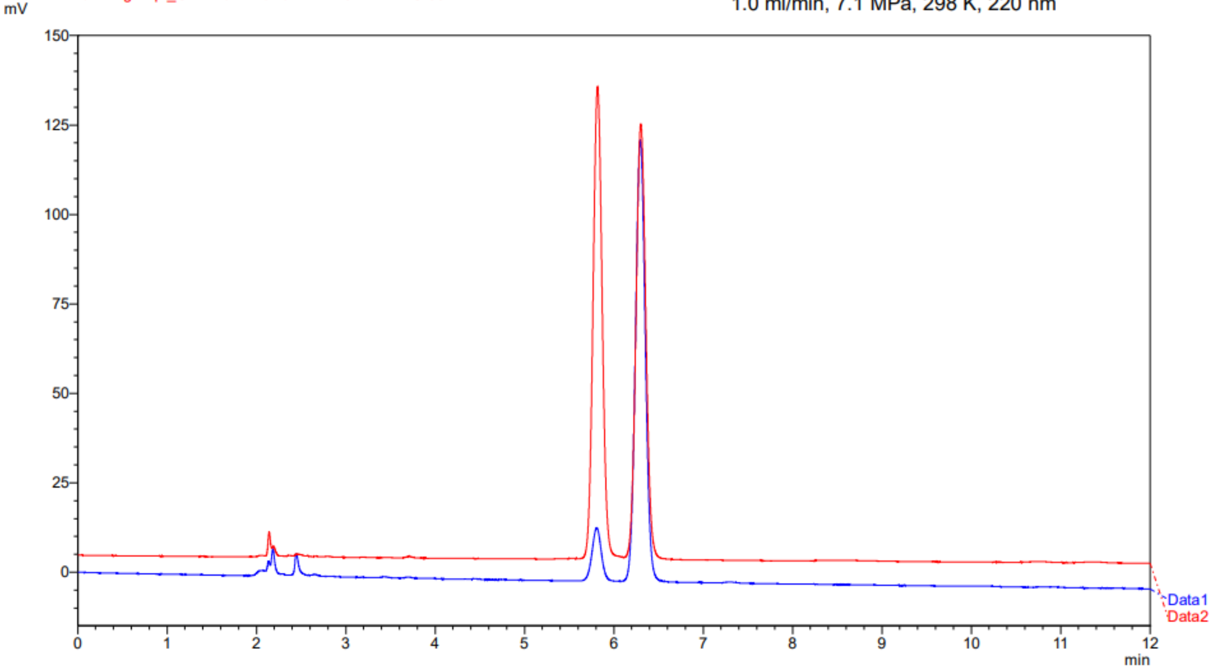

Compound (S)-2n

Gerät : NP-1

Operator : Ke
Sample Name : NIS-NA-046-rac
Vial # : 12
Injection Volume : 5 uL
Data File Name : NIS-NA-046-rac-IB-N3-08.lcd
Method File Name : Ni.lcm

Data Acquired: 11/23/2021 2:35:45 PM

Säulenauswahlventil
<<Oven>>
Valve 2/R Position : 0

5 µL NIS-NA-046-rac (GS in 200 µL 2-Propanol)
50 µL in 150 µL n-Heptan
150 mm Chiralpak IB-N3, 4.6 mm i.D.
n-Heptan/2-Propanol = 98:2
1.0 mL / min, 8.7 MPa, 298 K
220 nm

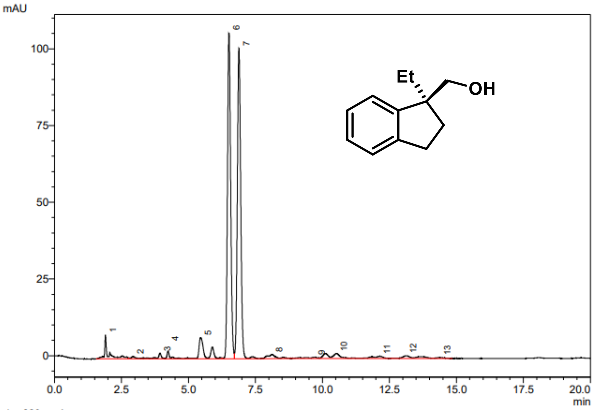

1 220nm,4nm

PDA Ch1 220nm

Peak #	Ret. Time	Area %	Name	Separation Factor	Resolution(DAB)
1	1.90	3.23		--	--
2	2.93	0.67		4.40	6.00
3	3.94	0.77		1.76	5.09
4	4.24	0.97		1.13	2.11
5	5.45	5.07		1.46	5.97
6	6.51	40.58	1. Enantiomer	1.27	4.34
7	6.89	40.67	2. Enantiomer	1.08	1.68
8	8.12	1.89		1.23	3.04
9	9.70	0.80		1.24	3.04
10	10.53	2.42		1.10	1.90
11	12.14	1.15		1.18	2.52
12	13.12	1.52		1.09	1.48
13	14.40	0.26		1.11	2.62
Total		100.00			

For good separation:

Separation Factor 1-10 AND R > 1,5

Gerät : NP-1

Operator : Ke
Sample Name : NIS-NA-046-01
Vial # : 22
Injection Volume : 5 uL
Data File Name : NIS-NA-046-01-IB-N3-04.lcd
Method File Name : Ni.lcm

Data Acquired: 11/23/2021 2:57:46 PM

5 µL NIS-NA-046-01 (GS in 200 µL 2-Propanol)
50 µL in 150 µL n-Heptan
150 mm Chiralpak IB-N3, 4.6 mm i.D.
n-Heptan/2-Propanol = 98:2
1.0 mL / min, 8.7 MPa, 298 K
220 nm

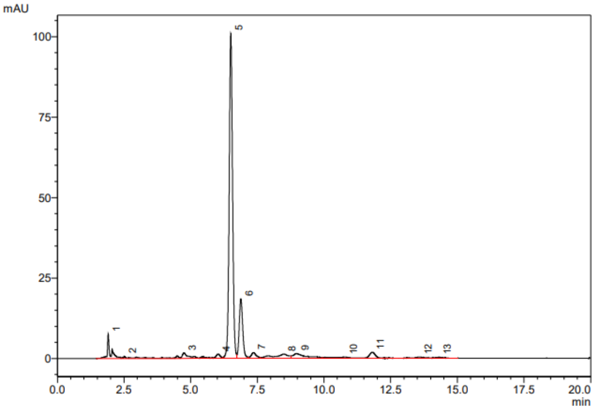

1 220nm,4nm

PDA Ch1 220nm

Peak #	Ret. Time	Area %	Name
1	1.91	4.00	
2	2.51	1.35	
3	4.75	2.43	
4	6.03	1.77	
5	6.50	64.03	1. Enantiomer
6	6.88	12.29	2. Enantiomer
7	7.36	1.77	
8	8.49	3.66	
9	8.97	3.65	
10	10.80	1.50	
11	11.81	2.23	
12	13.59	0.75	
13	14.31	0.57	
Total		100.00	

ee = 67.8 %

Data1 : W:\Workgroup_Geräte\NP-1\Ni\NIS-NA-046-rac-IB-N3-08.lcd racemate
Data2 : W:\Workgroup_Geräte\NP-1\Ni\NIS-NA-046-01-IB-N3-04.lcd ee-sample

150 mm Chiralpak IB-N3, 4.6 mm i.D.
n-Heptan/2-Propanol = 98:2
1.0 mL/min, 298 K, 220 nm

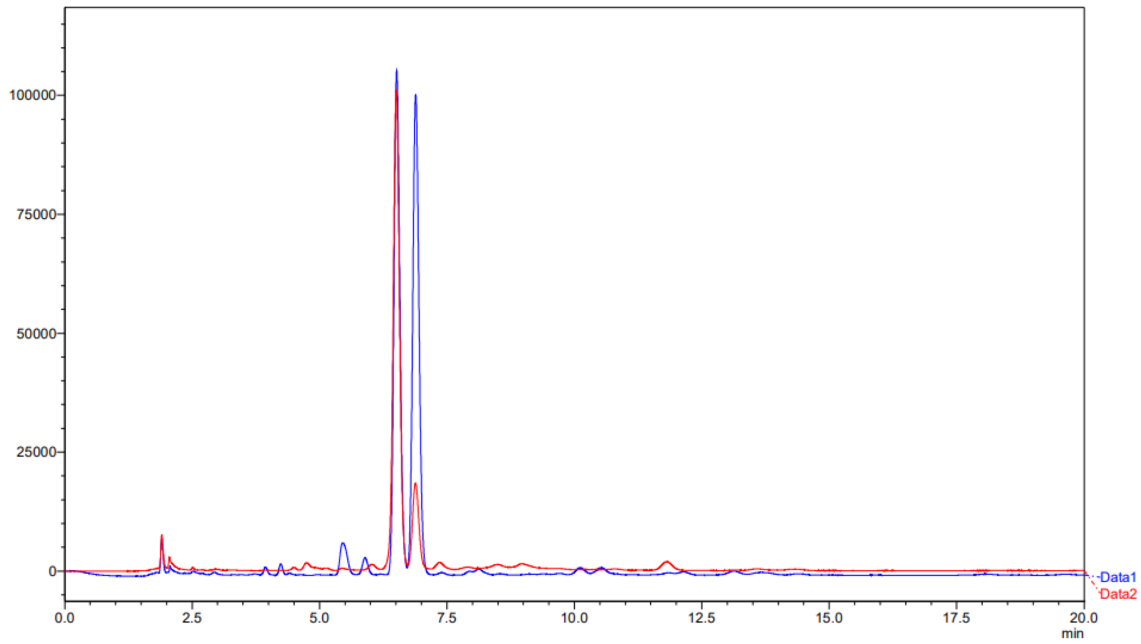

15 X-ray data

Single crystal structure analysis of (*R*)-(3-methyl-2,3-dihydrobenzofuran-3-yl)methyl 4-bromobenzoate

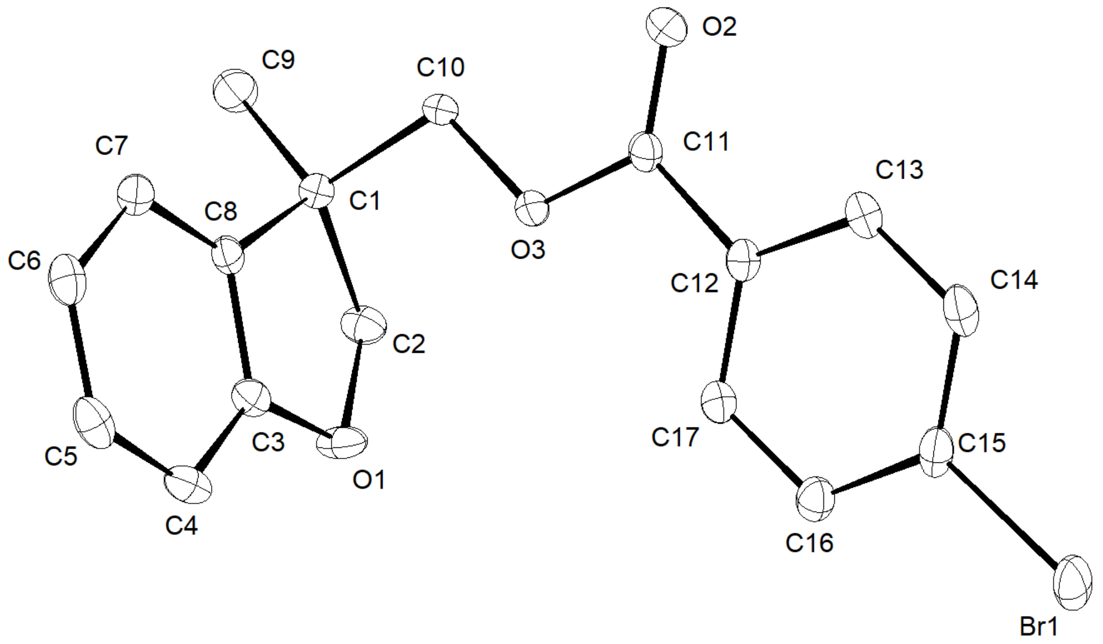

Figure S1. The molecular structure of **(*R*)-(3-methyl-2,3-dihydrobenzofuran-3-yl)methyl 4-bromobenzoate**. H atoms have been removed for clarity.

X-ray Crystal Structure Analysis of (*R*)-(3-methyl-2,3-dihydrobenzofuran-3-yl)methyl 4-bromobenzoate:

C_{17} H_{15} Br O_3, M_r = 347.20 g mol^{-1}, colourless block, crystal size 0.25 x 0.221 x 0.11 mm^3, monoclinic, space group $P2_1$ [4], a = 9.9412(8) Å, b = 6.0550(5) Å, c = 12.2322(10) Å, β = 93.423(3)°, V = 734.99(10) Å^3, T = 100(2) K, Z = 2, D_{calc} = 1.569 g·cm^3, λ = 0.71073 Å, μ(*Mo-K$_\alpha$*) = 2.803 mm^{-1}, analytical absorption correction ($T_{\min}$ = 0.59954, $T_{\max}$ = 0.76492), Bruker-AXS Kappa Mach3 with APEX-II detector and IµS microfocus Mo-anode X-ray source, 1.668 < θ < 30.508°, 23785 measured reflections, 4506 independent reflections, 4452 reflections with $I > 2\sigma(I)$, R_{int} = 0.0180. The structure was solved by *SHELXT* and refined by full-matrix least-squares (*SHELXL*) against F^2 to R_1 = 0.0222 [$I > 2\sigma(I)$], wR_2 = 0.0539, 191 parameters, 1 restraints and absolute structure parameter Flack (x) = -0.0032(19).

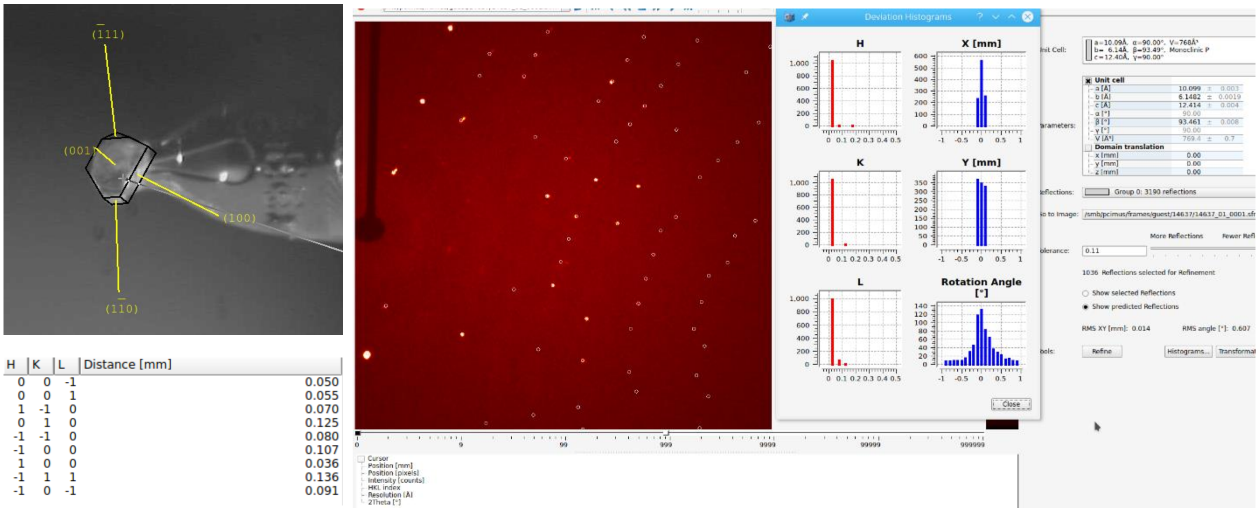

Figure S2. Crystal faces and unit cell determination/refinement ***(R)*-(3-methyl-2,3-dihydrobenzofuran-3-yl)methyl 4-bromobenzoate**.

INTENSITY STATISTICS FOR DATASET

Resolution	#Data	#Theory	%Complete	Redundancy	Mean I	Mean I/s	Rmerge	Rsigma
Inf - 2.82	71	71	100.0	8.51	131.95	126.55	0.0192	0.0076
2.82 - 1.88	167	167	100.0	9.11	64.95	131.65	0.0161	0.0073
1.88 - 1.48	240	240	100.0	9.19	45.29	125.54	0.0154	0.0075
1.48 - 1.29	241	241	100.0	9.23	29.64	116.97	0.0154	0.0077
1.29 - 1.17	245	245	100.0	8.76	23.40	105.18	0.0167	0.0083
1.17 - 1.09	230	230	100.0	8.26	23.71	100.60	0.0168	0.0088
1.09 - 1.03	218	218	100.0	6.46	16.82	83.18	0.0192	0.0107
1.03 - 0.97	281	281	100.0	5.33	13.57	69.11	0.0187	0.0126
0.97 - 0.94	189	189	100.0	4.73	12.04	63.34	0.0208	0.0142
0.94 - 0.89	311	311	100.0	4.30	10.01	54.19	0.0229	0.0160
0.89 - 0.86	229	229	100.0	3.91	8.63	47.08	0.0241	0.0181
0.86 - 0.84	182	182	100.0	3.88	8.54	45.35	0.0241	0.0184
0.84 - 0.81	307	307	100.0	3.77	7.69	43.43	0.0255	0.0196
0.81 - 0.79	224	224	100.0	3.54	6.19	35.22	0.0287	0.0230
0.79 - 0.77	234	234	100.0	3.46	4.66	30.12	0.0329	0.0277
0.77 - 0.75	278	278	100.0	3.30	5.39	31.67	0.0326	0.0270
0.75 - 0.74	174	174	100.0	3.35	4.63	28.83	0.0363	0.0302
0.74 - 0.72	316	316	100.0	3.30	4.52	26.21	0.0346	0.0309
0.72 - 0.71	174	175	99.4	3.11	4.12	24.82	0.0392	0.0347
0.71 - 0.70	203	204	99.5	3.09	3.71	22.83	0.0426	0.0378
0.70 - 0.69	199	219	90.9	2.39	3.56	19.67	0.0419	0.0445
0.95 - 0.85	606	717	84.5	6.44	21.27	62.39	0.0278	0.0212
Inf - 0.85	2405	2531	95.0	9.72	57.85	80.30	0.0270	0.0128

Complete .cif-data of the compound are available under the CCDC number **CCDC-2173277**.

Table S1. Crystal data and structure refinement of ***(R)*-(3-methyl-2,3-dihydrobenzofuran-3-yl)methyl 4-bromobenzoate**.

Identification code	14637	
Empirical formula	$C_{17} H_{15} Br O_3$	
Color	colourless	
Formula weight	347.20 g·mol^{-1}	
Temperature	100(2) K	
Wavelength	0.71073 Å	
Crystal system	Monoclinic	
Space group	$P2_1$, (no. 4)	
Unit cell dimensions	a = 9.9412(8) Å	α= 90°.
	b = 6.0550(5) Å	β= 93.423(3)°.
	c = 12.2322(10) Å	γ = 90°.
Volume	734.99(10) Å^3	
Z	2	
Density (calculated)	1.569 Mg·m^{-3}	
Absorption coefficient	2.803 mm^{-1}	
F(000)	352 e	
Crystal size	0.25 x 0.221 x 0.11 mm^3	
θ range for data collection	1.668 to 30.508°.	
Index ranges	$-14 \leq h \leq 14, -8 \leq k \leq 8, -17 \leq l \leq 17$	
Reflections collected	23785	
Independent reflections	4506 [R_{int} = 0.0180]	
Reflections with $I>2\sigma(I)$	4452	
Completeness to θ = 25.242°	100.0 %	
Absorption correction	Gaussian	
Max. and min. transmission	0.76492 and 0.59954	
Refinement method	Full-matrix least-squares on F^2	
Data / restraints / parameters	4506 / 1 / 191	
Goodness-of-fit on F^2	1.066	
Final R indices [$I>2\sigma(I)$]	R_1 = 0.0222	wR^2 = 0.0537
R indices (all data)	R_1 = 0.0226	wR^2 = 0.0539
Absolute structure parameter	-0.0032(19)	
Extinction coefficient	n/a	

Largest diff. peak and hole	1.094 and -0.805 $e\cdot Å^{-3}$

Table S2. Bond lengths [Å] and angles [°] of ***(R)*-(3-methyl-2,3-dihydrobenzofuran-3-yl)methyl 4-bromobenzoate**.

Br(1)-C(15)	1.889(2)	O(1)-C(2)	1.455(3)
O(1)-C(3)	1.363(2)	O(2)-C(11)	1.210(3)
O(3)-C(10)	1.446(2)	O(3)-C(11)	1.336(2)
C(1)-C(2)	1.549(3)	C(1)-C(8)	1.515(3)
C(1)-C(9)	1.528(3)	C(1)-C(10)	1.526(3)
C(2)-H(2A)	0.9900	C(2)-H(2B)	0.9900
C(3)-C(4)	1.388(3)	C(3)-C(8)	1.386(3)
C(4)-H(4)	0.9500	C(4)-C(5)	1.394(3)
C(5)-H(5)	0.9500	C(5)-C(6)	1.389(4)
C(6)-H(6)	0.9500	C(6)-C(7)	1.400(3)
C(7)-H(7)	0.9500	C(7)-C(8)	1.381(3)
C(9)-H(9A)	0.9800	C(9)-H(9B)	0.9800
C(9)-H(9C)	0.9800	C(10)-H(10A)	0.9900
C(10)-H(10B)	0.9900	C(11)-C(12)	1.487(3)
C(12)-C(13)	1.394(3)	C(12)-C(17)	1.393(3)
C(13)-H(13)	0.9500	C(13)-C(14)	1.383(3)
C(14)-H(14)	0.9500	C(14)-C(15)	1.387(3)
C(15)-C(16)	1.388(3)	C(16)-H(16)	0.9500
C(16)-C(17)	1.389(3)	C(17)-H(17)	0.9500
C(3)-O(1)-C(2)	106.72(15)	C(11)-O(3)-C(10)	114.96(15)
C(8)-C(1)-C(2)	99.80(16)	C(8)-C(1)-C(9)	113.39(16)
C(8)-C(1)-C(10)	111.66(16)	C(9)-C(1)-C(2)	112.78(19)
C(10)-C(1)-C(2)	112.06(17)	C(10)-C(1)-C(9)	107.20(17)
O(1)-C(2)-C(1)	107.63(16)	O(1)-C(2)-H(2A)	110.2
O(1)-C(2)-H(2B)	110.2	C(1)-C(2)-H(2A)	110.2
C(1)-C(2)-H(2B)	110.2	H(2A)-C(2)-H(2B)	108.5
O(1)-C(3)-C(4)	124.25(19)	O(1)-C(3)-C(8)	113.23(17)
C(8)-C(3)-C(4)	122.5(2)	C(3)-C(4)-H(4)	121.5
C(3)-C(4)-C(5)	117.0(2)	C(5)-C(4)-H(4)	121.5
C(4)-C(5)-H(5)	119.3	C(6)-C(5)-C(4)	121.4(2)
C(6)-C(5)-H(5)	119.3	C(5)-C(6)-H(6)	119.9

C(5)-C(6)-C(7)	120.2(2)	C(7)-C(6)-H(6)	119.9
C(6)-C(7)-H(7)	120.5	C(8)-C(7)-C(6)	119.0(2)
C(8)-C(7)-H(7)	120.5	C(3)-C(8)-C(1)	109.09(17)
C(7)-C(8)-C(1)	131.07(18)	C(7)-C(8)-C(3)	119.84(19)
C(1)-C(9)-H(9A)	109.5	C(1)-C(9)-H(9B)	109.5
C(1)-C(9)-H(9C)	109.5	H(9A)-C(9)-H(9B)	109.5
H(9A)-C(9)-H(9C)	109.5	H(9B)-C(9)-H(9C)	109.5
O(3)-C(10)-C(1)	107.99(15)	O(3)-C(10)-H(10A)	110.1
O(3)-C(10)-H(10B)	110.1	C(1)-C(10)-H(10A)	110.1
C(1)-C(10)-H(10B)	110.1	H(10A)-C(10)-H(10B)	108.4
O(2)-C(11)-O(3)	123.89(19)	O(2)-C(11)-C(12)	124.04(19)
O(3)-C(11)-C(12)	112.07(17)	C(13)-C(12)-C(11)	118.0(2)
C(17)-C(12)-C(11)	122.19(19)	C(17)-C(12)-C(13)	119.8(2)
C(12)-C(13)-H(13)	119.7	C(14)-C(13)-C(12)	120.7(2)
C(14)-C(13)-H(13)	119.7	C(13)-C(14)-H(14)	120.6
C(13)-C(14)-C(15)	118.8(2)	C(15)-C(14)-H(14)	120.6
C(14)-C(15)-Br(1)	119.04(17)	C(14)-C(15)-C(16)	121.5(2)
C(16)-C(15)-Br(1)	119.42(18)	C(15)-C(16)-H(16)	120.4
C(15)-C(16)-C(17)	119.3(2)	C(17)-C(16)-H(16)	120.4
C(12)-C(17)-H(17)	120.0	C(16)-C(17)-C(12)	119.9(2)
C(16)-C(17)-H(17)	120.0		

16 References

[1] a) Jin, Y.; Wang, C., Nickel-Catalyzed Asymmetric Reductive Arylalkylation of Unactivated Alkenes. *Angew. Chem., Int. Ed.* **2019,** *58*, 6722-6726; b) Jin, Y.; Wang, C., Ni-catalysed reductive arylalkylation of unactivated alkenes. *Chem. Sci.* **2019,** *10*, 1780-1785; c) He, J.; Xue, Y.; Han, B.; Zhang, C.; Wang, Y.; Zhu, S., Nickel-Catalyzed Asymmetric Reductive 1,2-Carboamination of Unactivated Alkenes. *Angew. Chem., Int. Ed.* **2020,** *59*, 2328-2332; d) Jin, Y.; Yang, H.; Wang, C., Nickel-Catalyzed Asymmetric Reductive Arylbenzylation of Unactivated Alkenes. *Org. Lett.* **2020,** *22*, 2724-2729;

[2] a) Wang, T.; Chen, F.; Qin, J.; He, Y.-M.; Fan, Q.-H., Asymmetric Ruthenium-Catalyzed Hydrogenation of 2- and 2,9-Substituted 1,10-Phenanthrolines. *Angew. Chem., Int. Ed.* **2013,** *52*, 7172-7176; b) Martinez, J. F.; La Porte, N. T.; Chaudhuri, S.; Sinopoli, A.; Bae, Y. J.; Sohail, M.; Batista, V. S.; Wasielewski, M. R., Effect of Electronic Coupling on Electron Transfer Rates from Photoexcited Naphthalenediimide Radical Anion to Re(bpy)$(CO)_3$X. *J. Phys. Chem. C* **2019,** *123*, 10178-10190; c) He, J.; Bai, Z.-Q.; Yuan, P.-F.; Wu, L.-Z.; Liu, Q., Highly Efficient Iridium-Based Photosensitizers for Thia-Paternò–Büchi Reaction and Aza-Photocyclization. *ACS Catal.* **2021,** *11*, 446-455; d) Cerveri, A.; Giovanelli, R.; Sella, D.; Pedrazzani, R.; Monari, M.; Nieto Faza, O.; López, C. S.; Bandini, M., Enantioselective CO_2 Fixation Via a Heck-Coupling/Carboxylation Cascade Catalyzed by Nickel. *Chem. Eur. J.* **2021,** *27*, 7657-7662.

[3] Fenselau, A. H.; Moffatt, J. G., Sulfoxide-Carbodiimide Reactions. III.[1] Mechanism of the Oxidation Reaction. *J. Am. Chem. Soc.* **1966,** *88*, 1762-1765.

[4] Bothner-By, A.; Friedman, L., The Reaction of Nitrous Acid with Hydroxylamine. *J. Chem. Phys.* **1952,** *20*, 459-462.

17 NMR Spectra

Compound 1c ^{1}H NMR

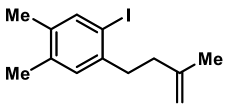

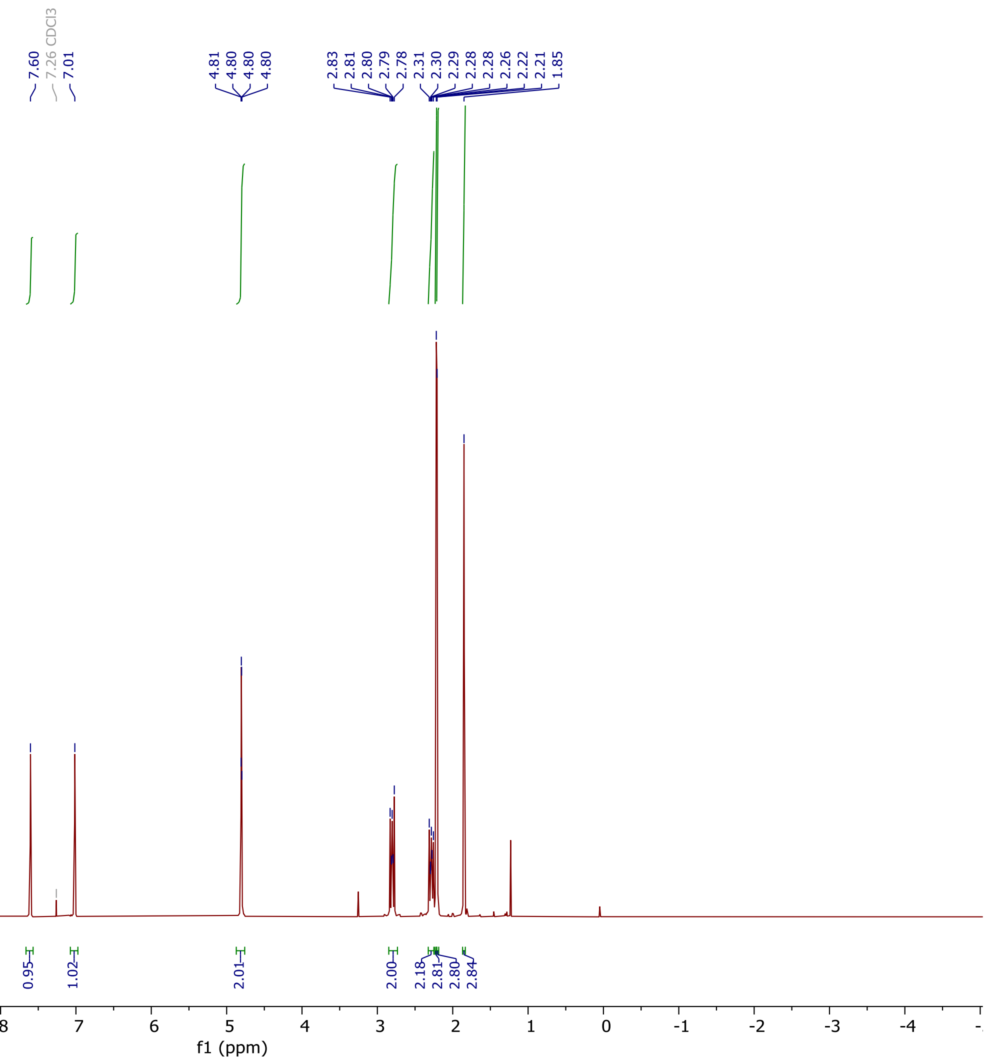

Compound 1c ^{13}C NMR

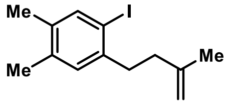

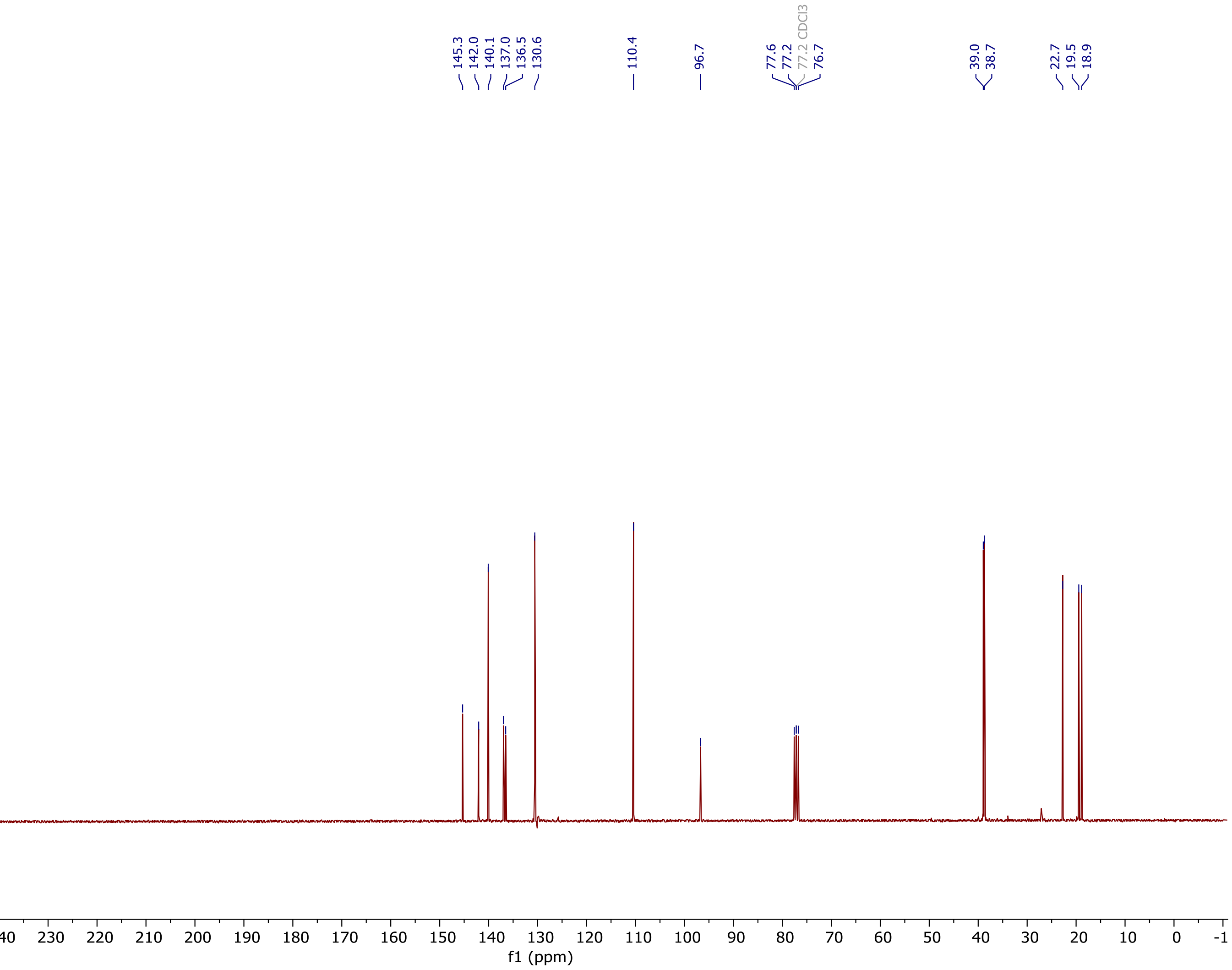

Compound 1d ^{1}H NMR

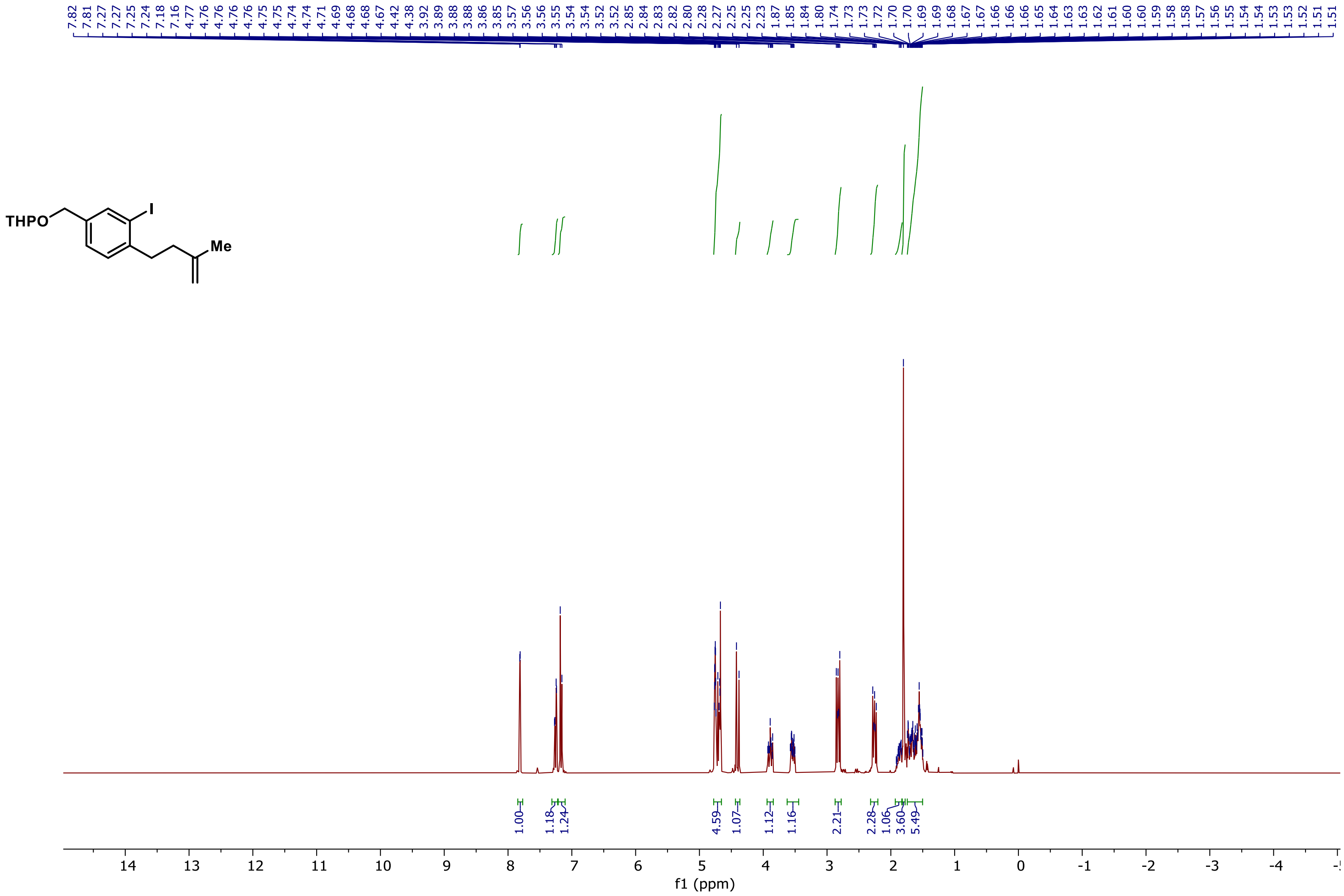

Compound 1d ^{13}C NMR

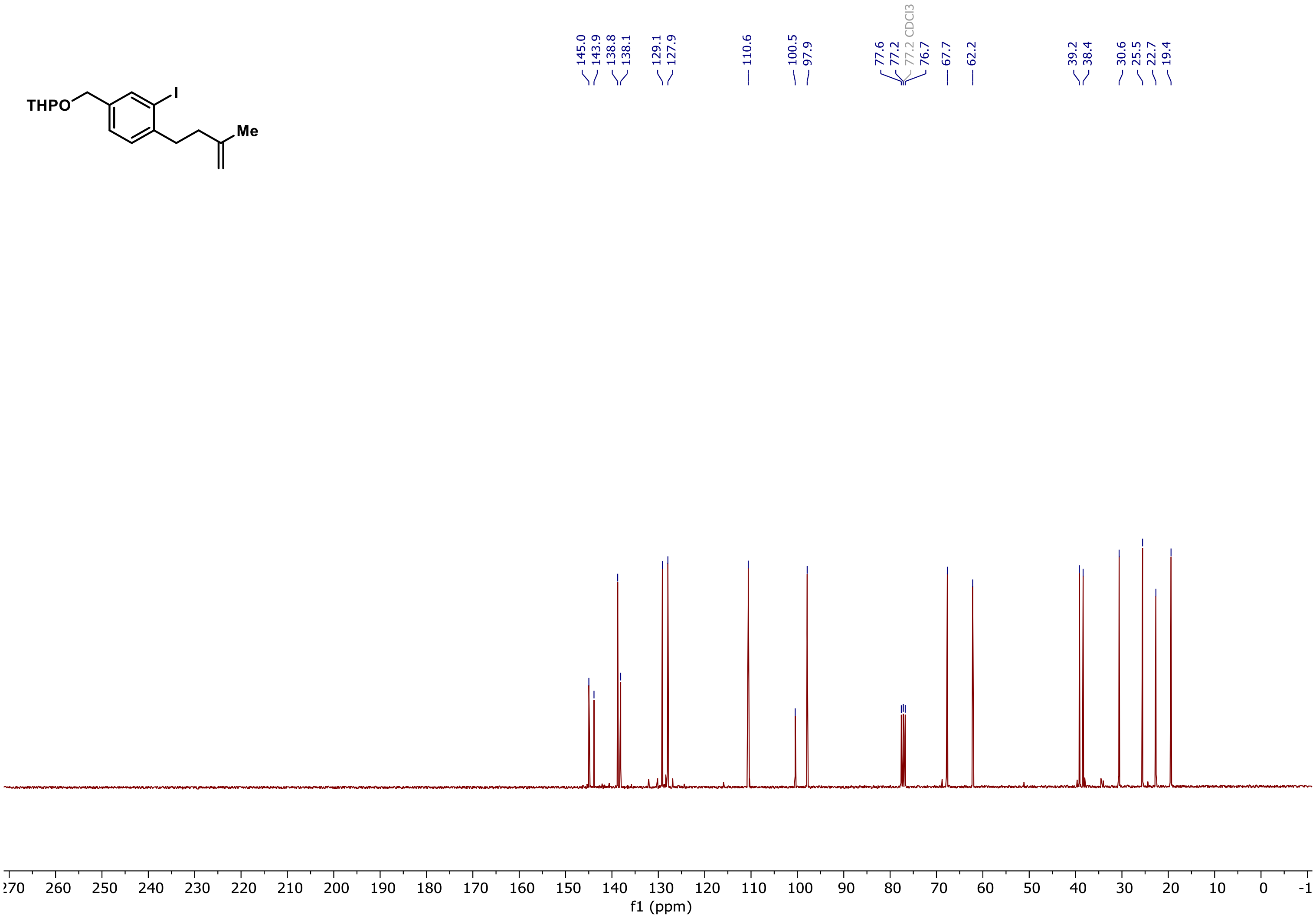

Compound 1e ^{1}H NMR

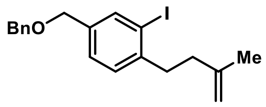

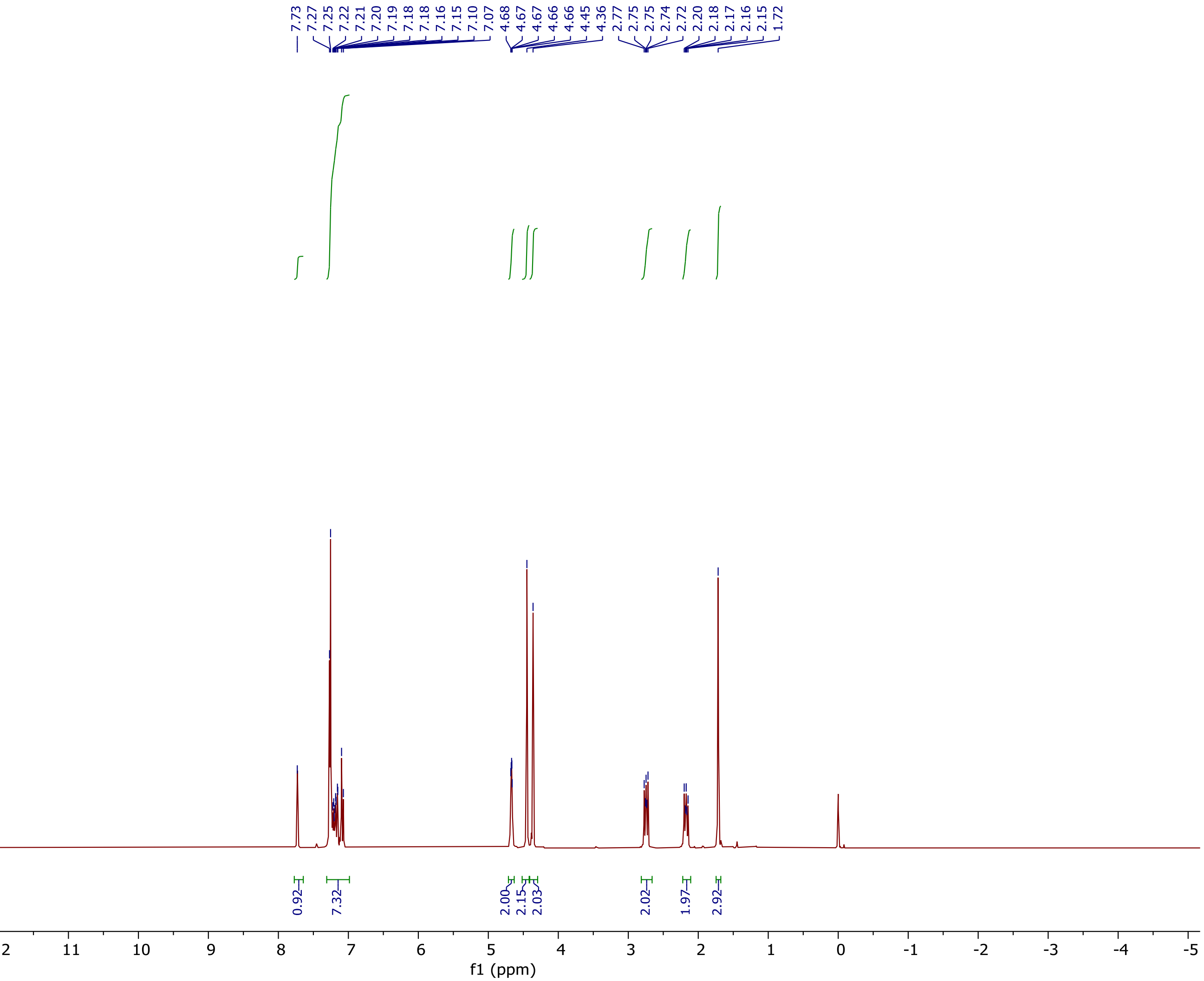

Compound 1e ^{13}C NMR

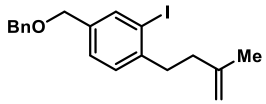

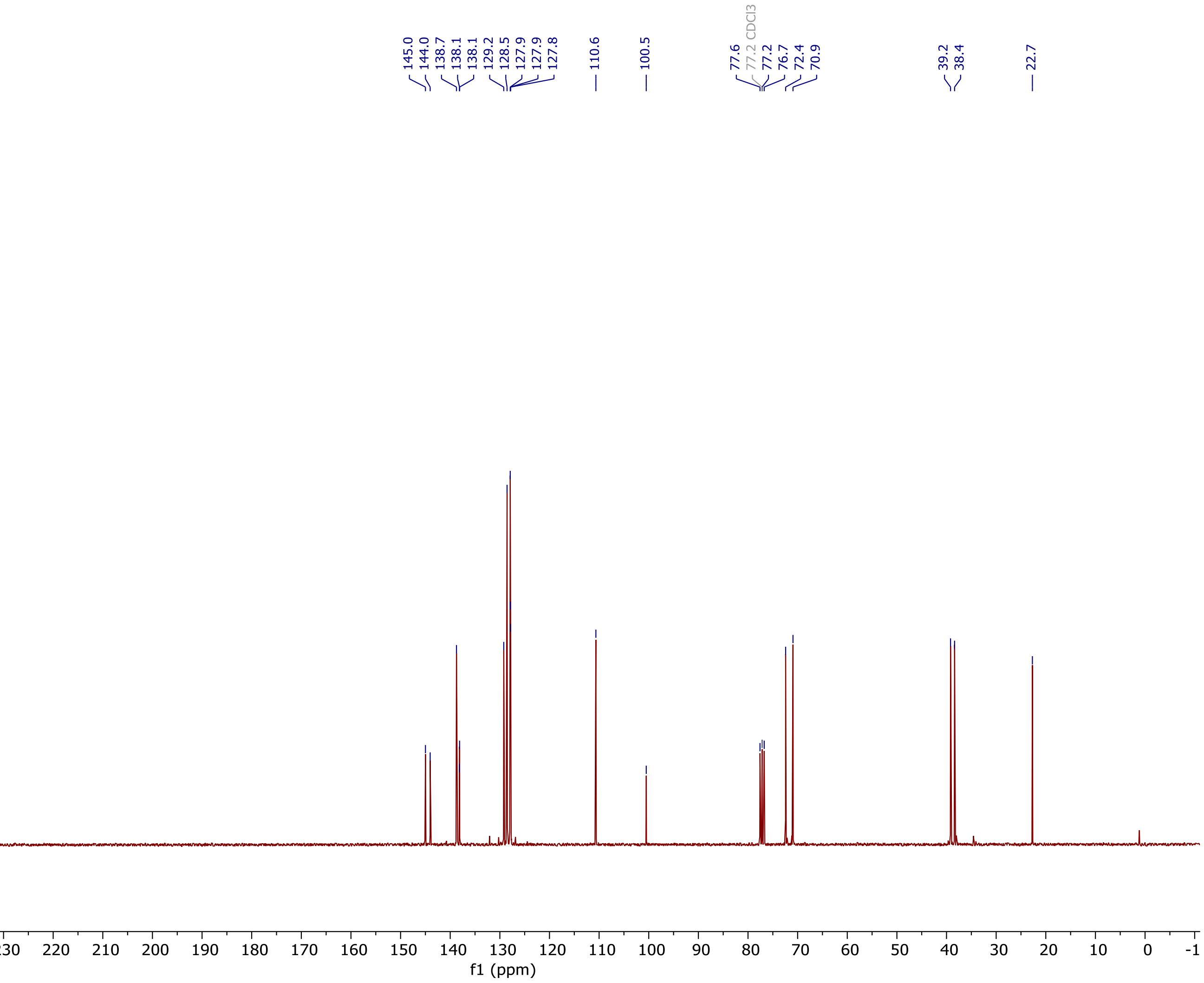

Compound 1f ^{1}H NMR

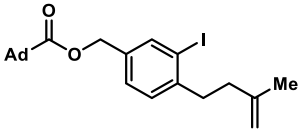

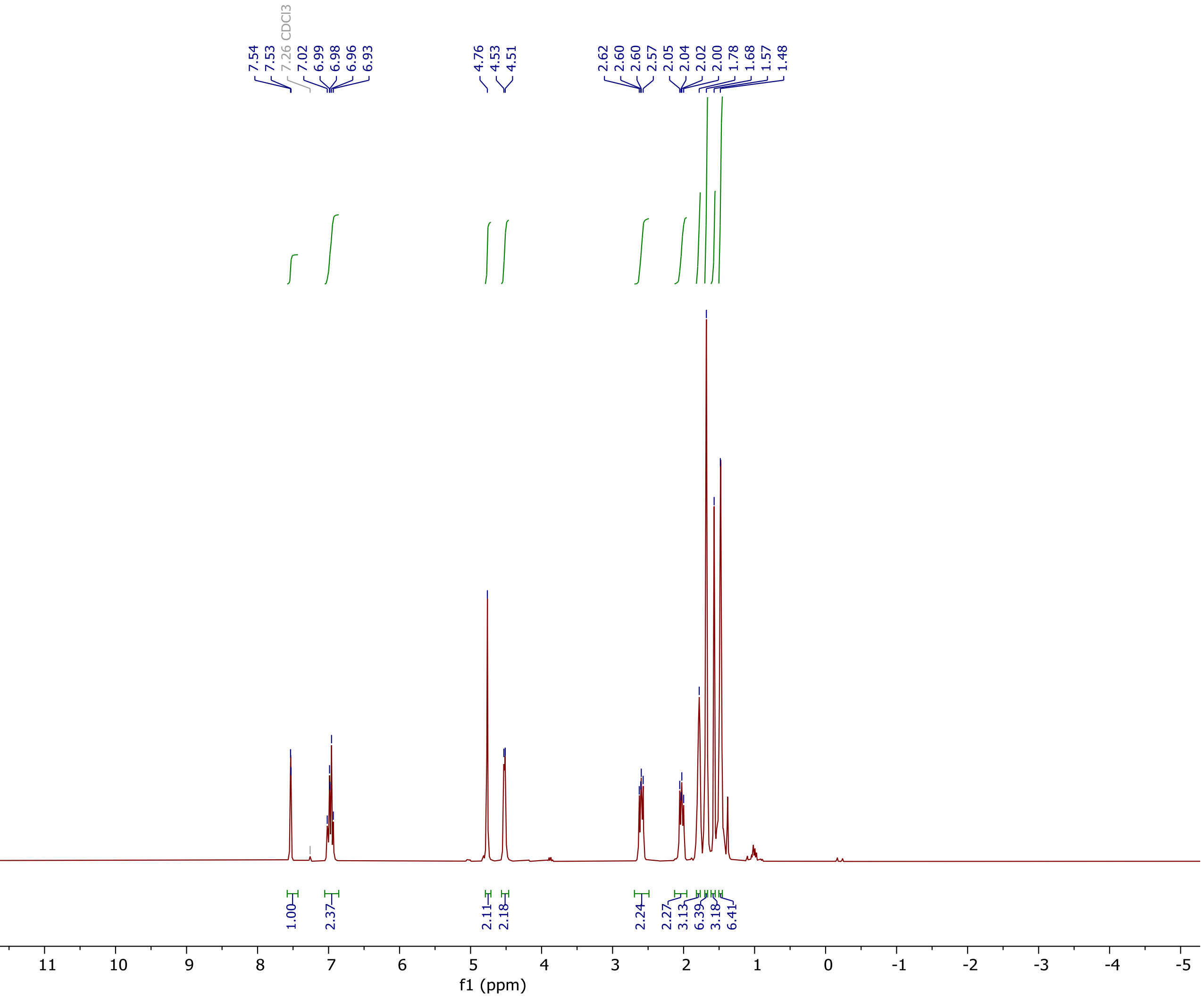

Compound 1f ^{13}C NMR

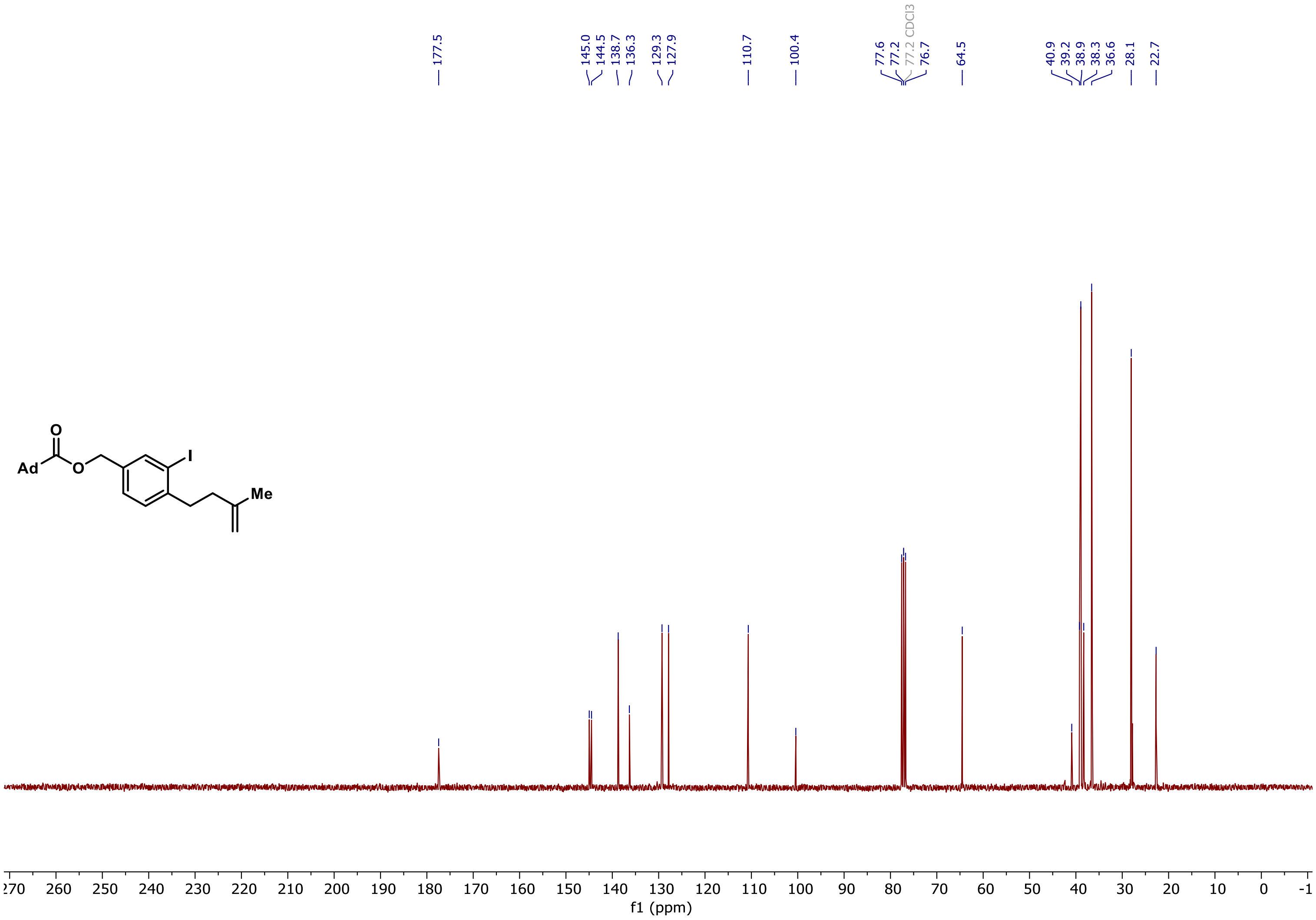

Compound 1K ¹H NMR

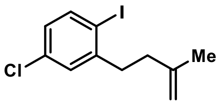

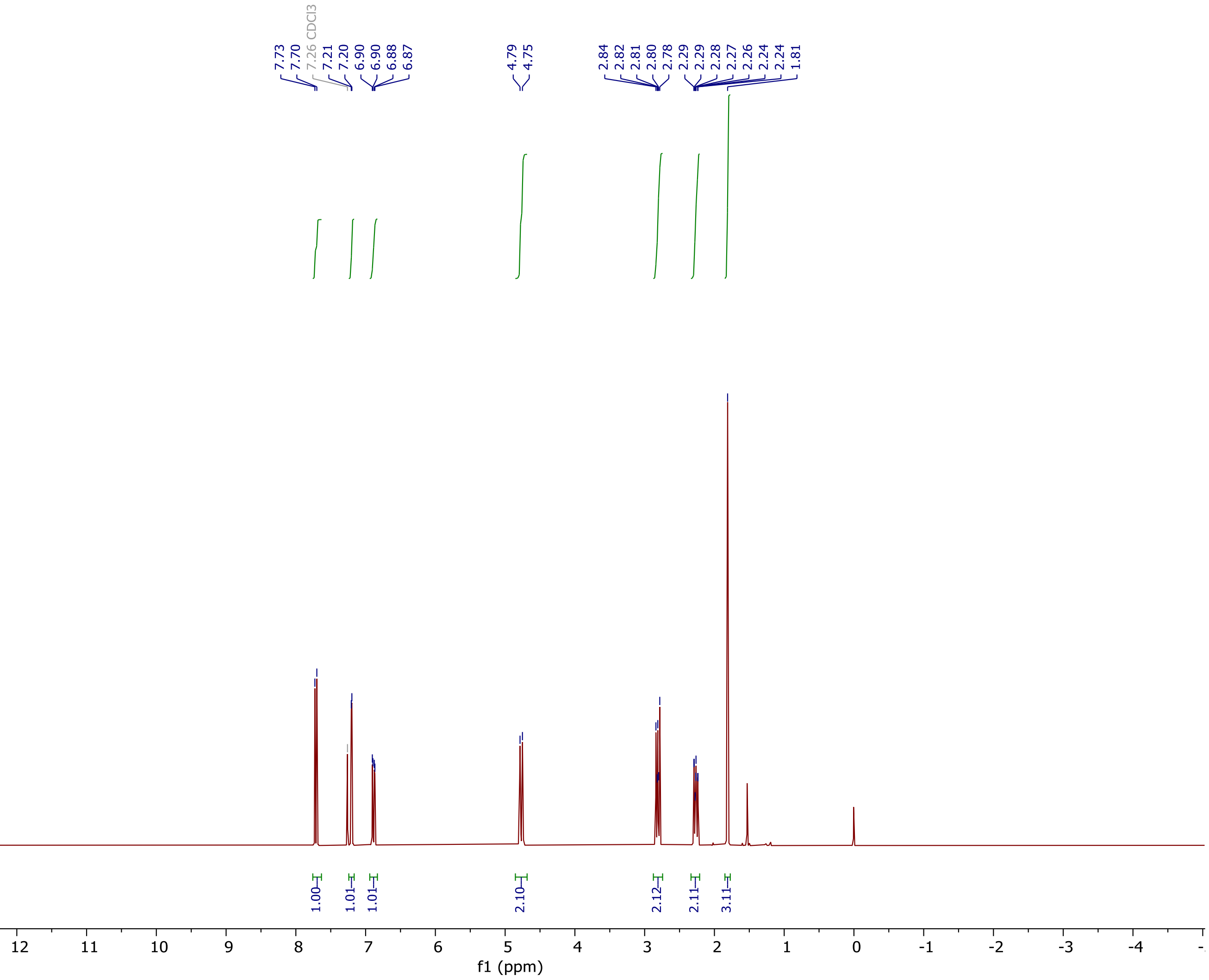

Compound 1k ^{13}C NMR

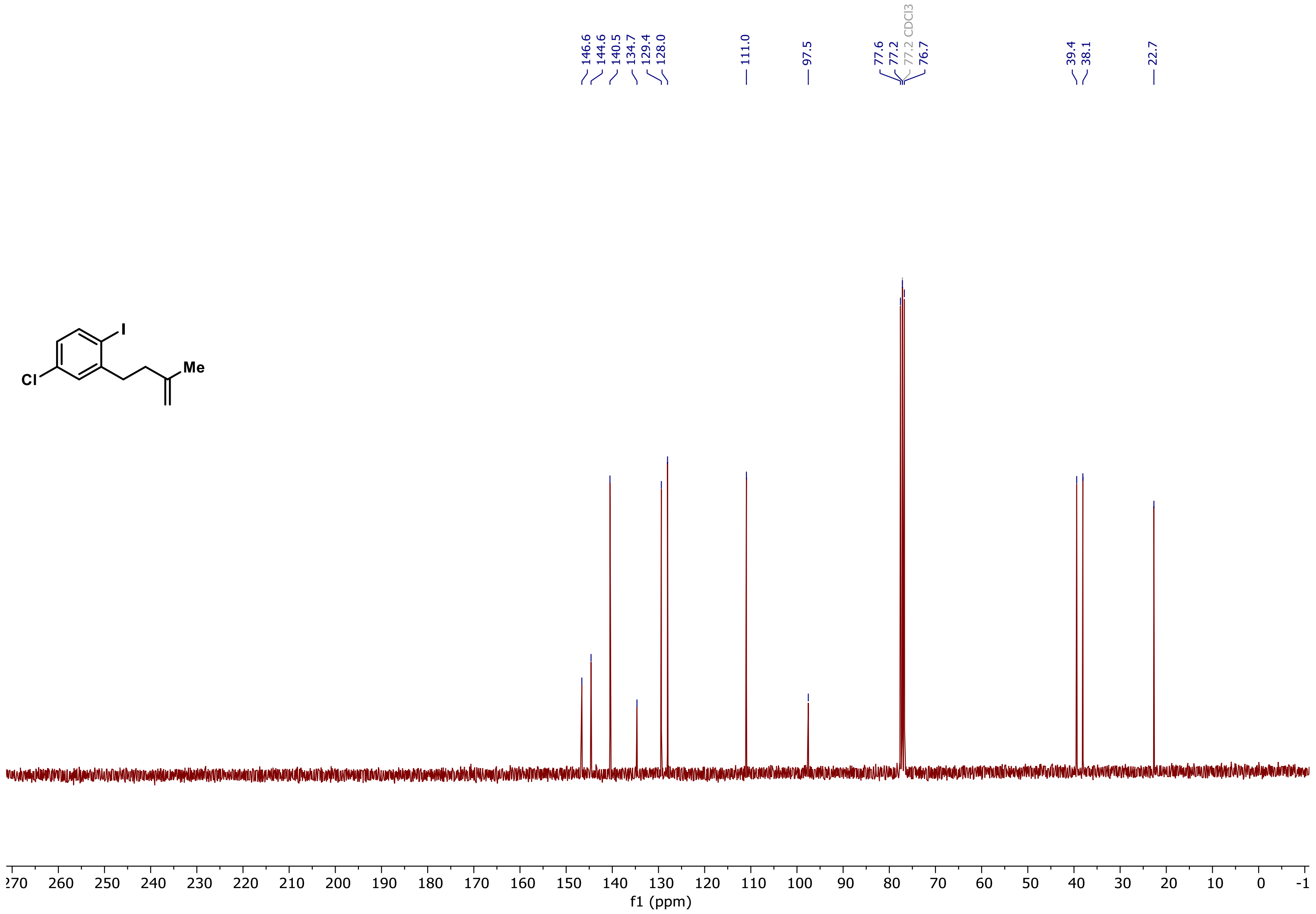

Compound 1l ^{1}H NMR

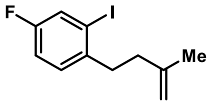

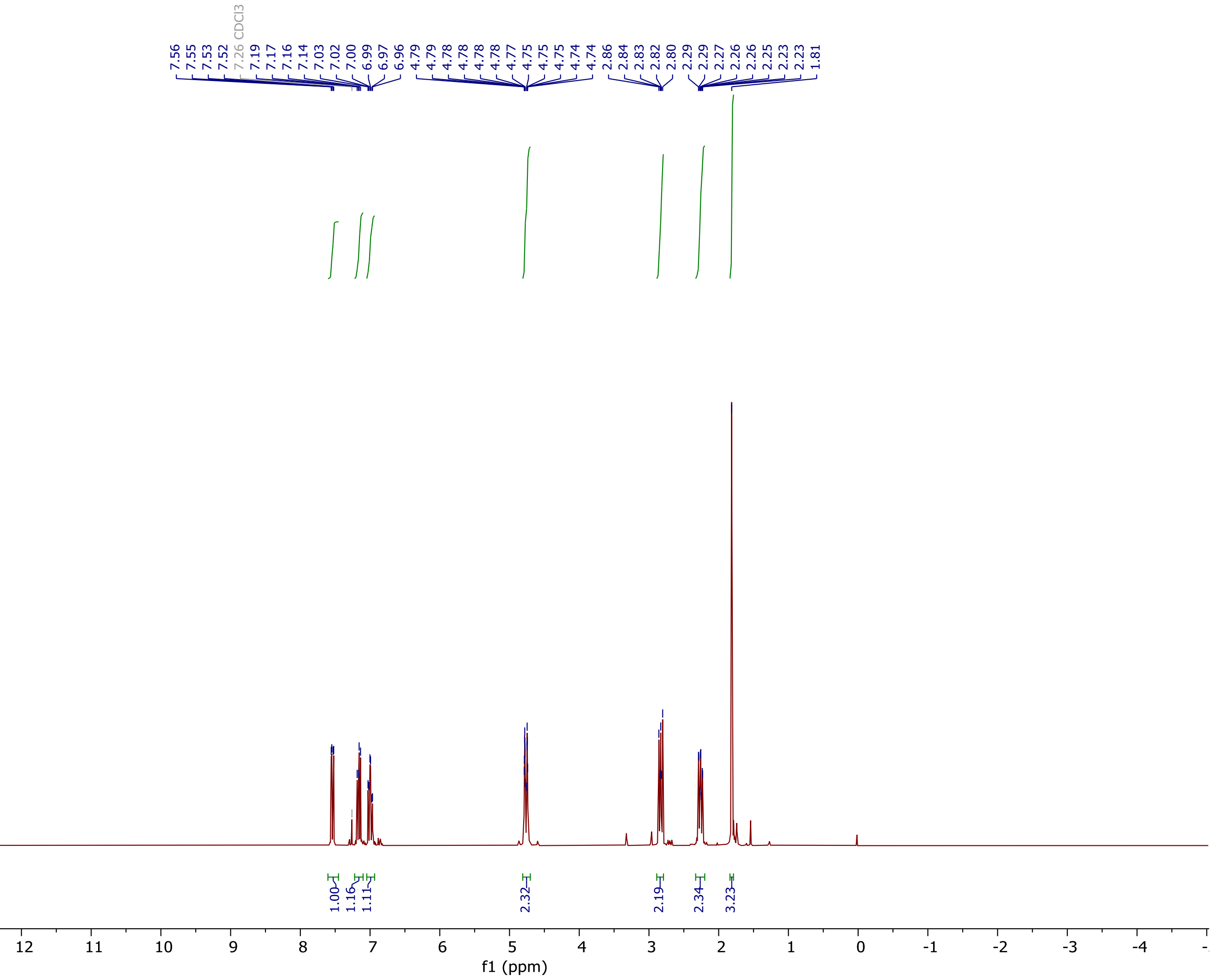

Compound 1l ^{13}C NMR

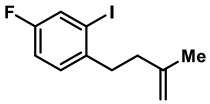

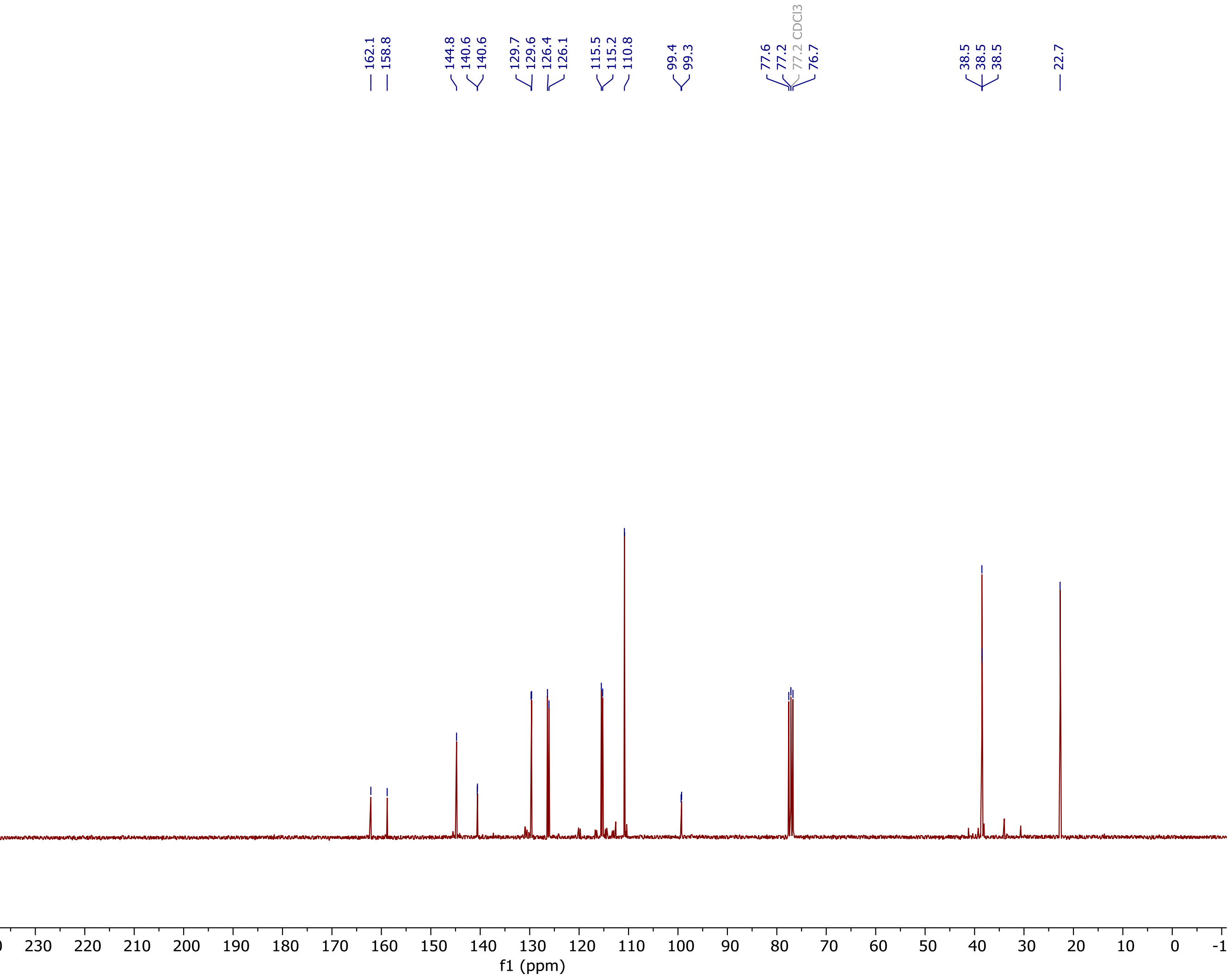

Compound 1l ^{19}F NMR

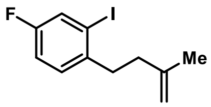

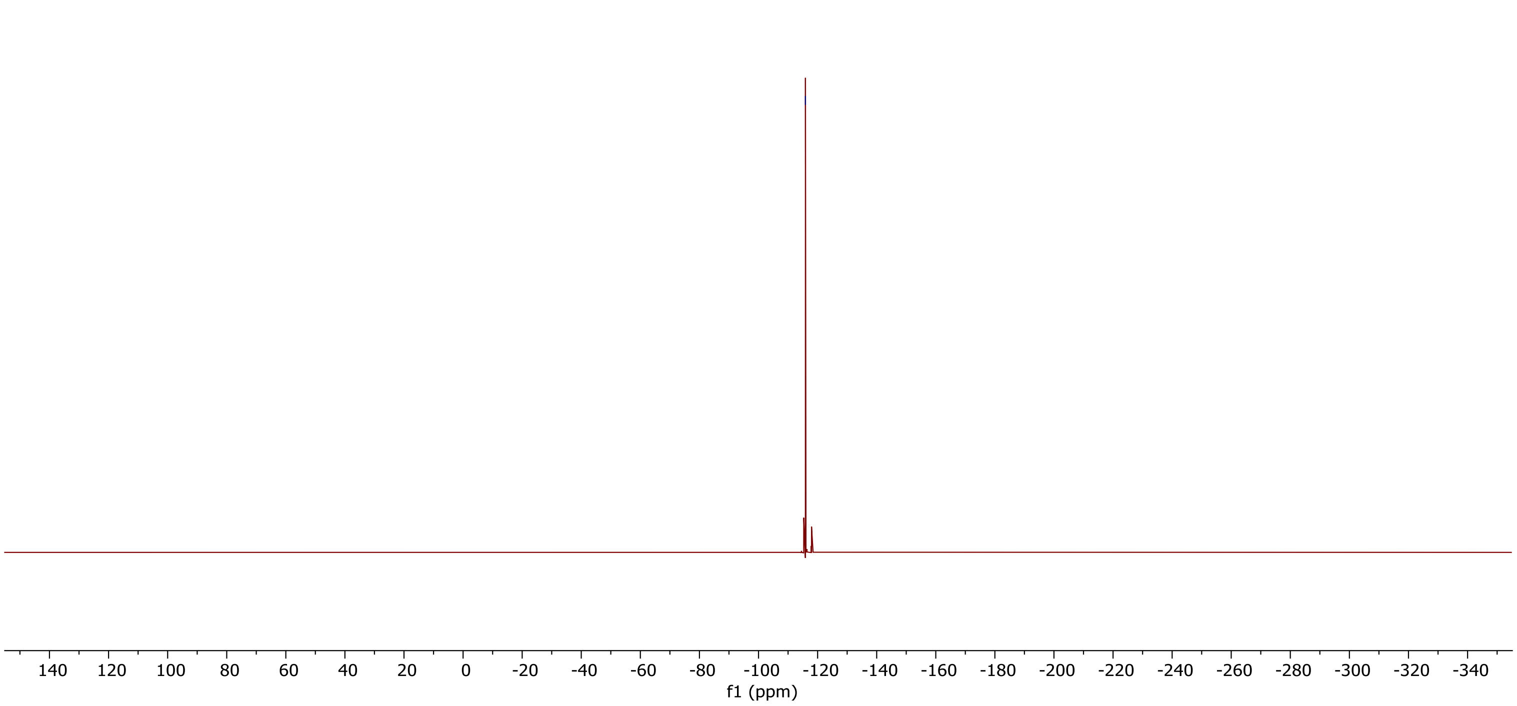

Compound 1m ^{1}H NMR

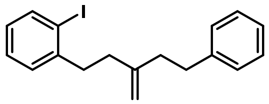

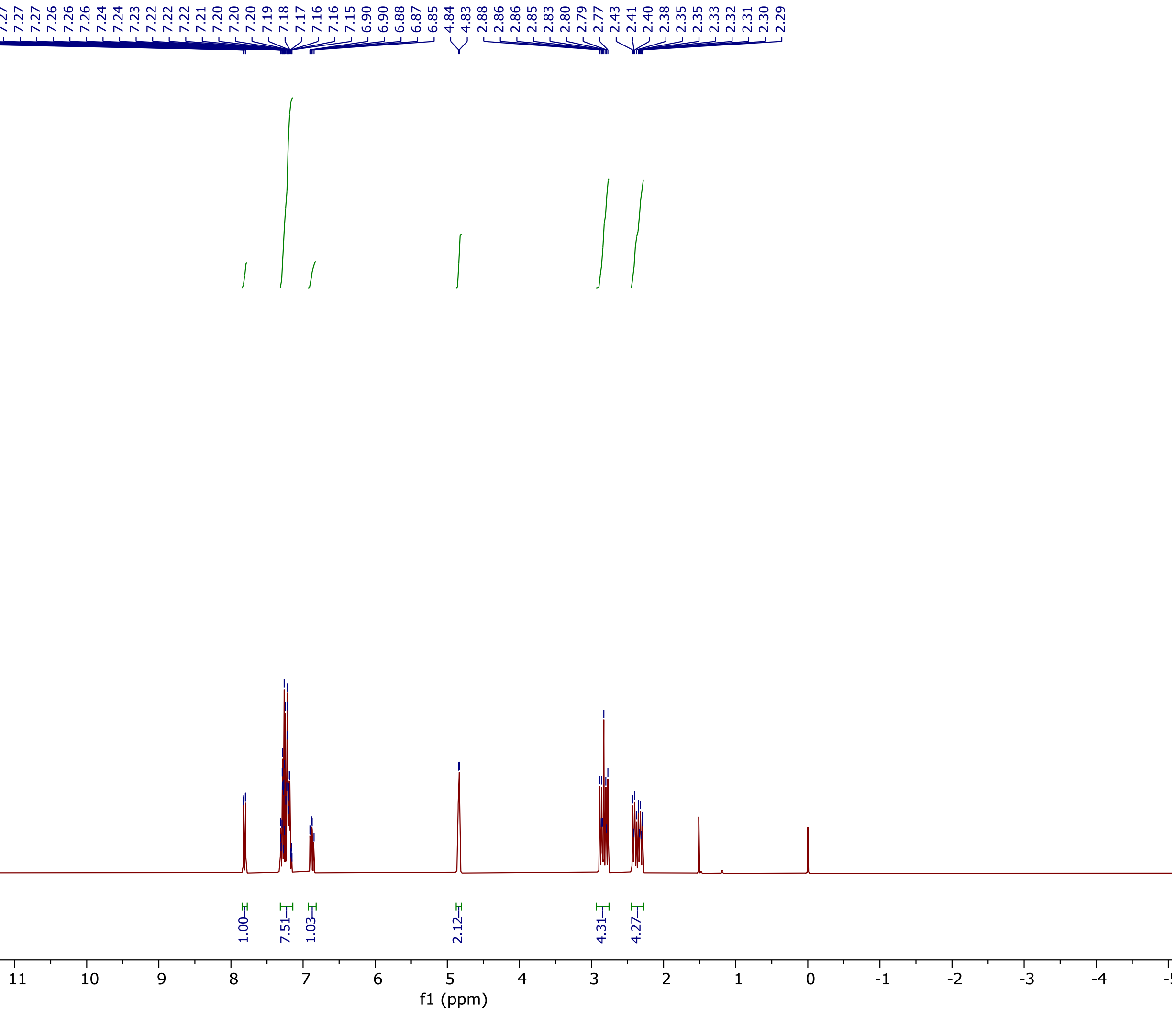

Compound 1m ^{13}C NMR

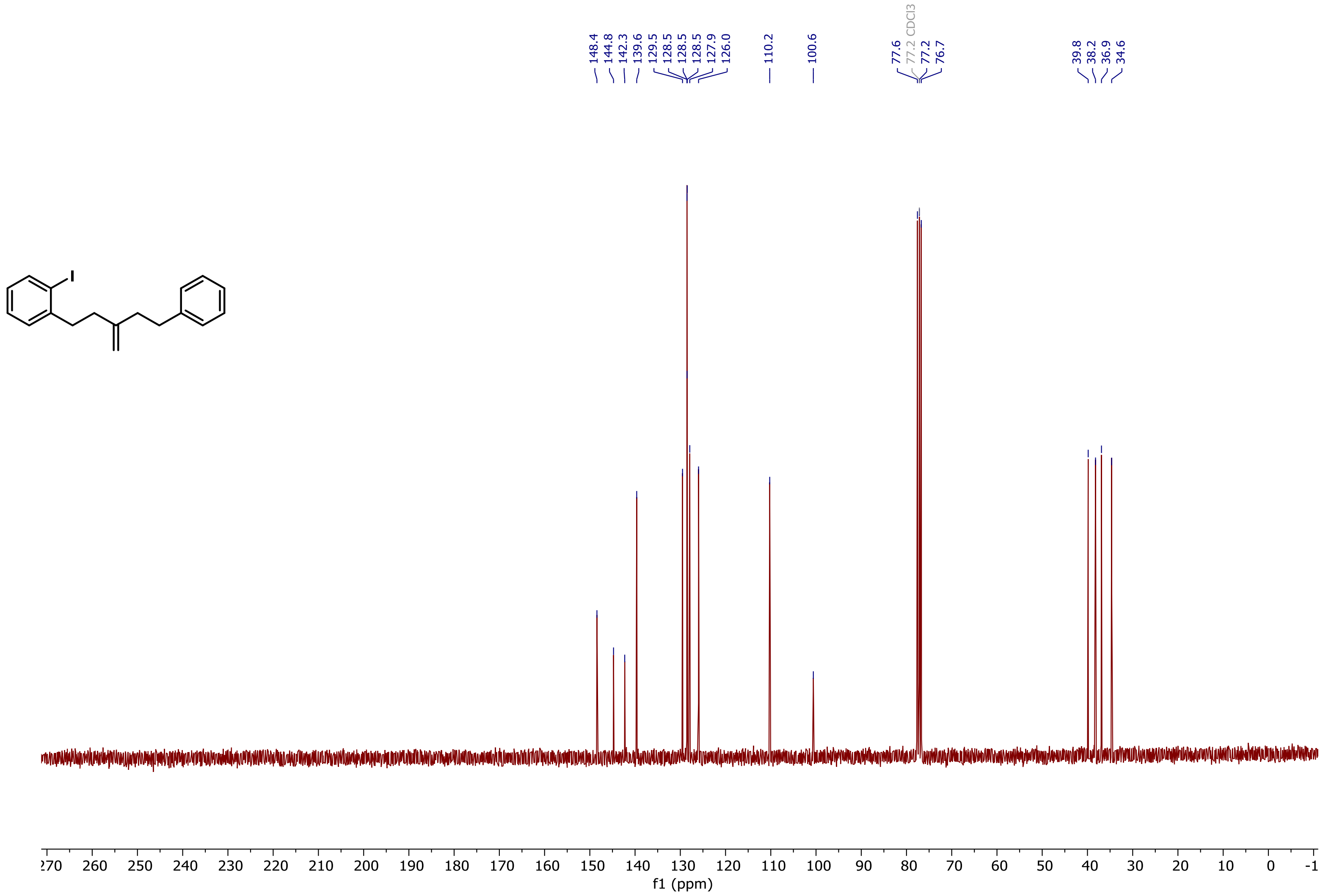

Compound 1o ¹H NMR

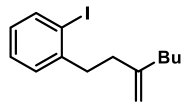

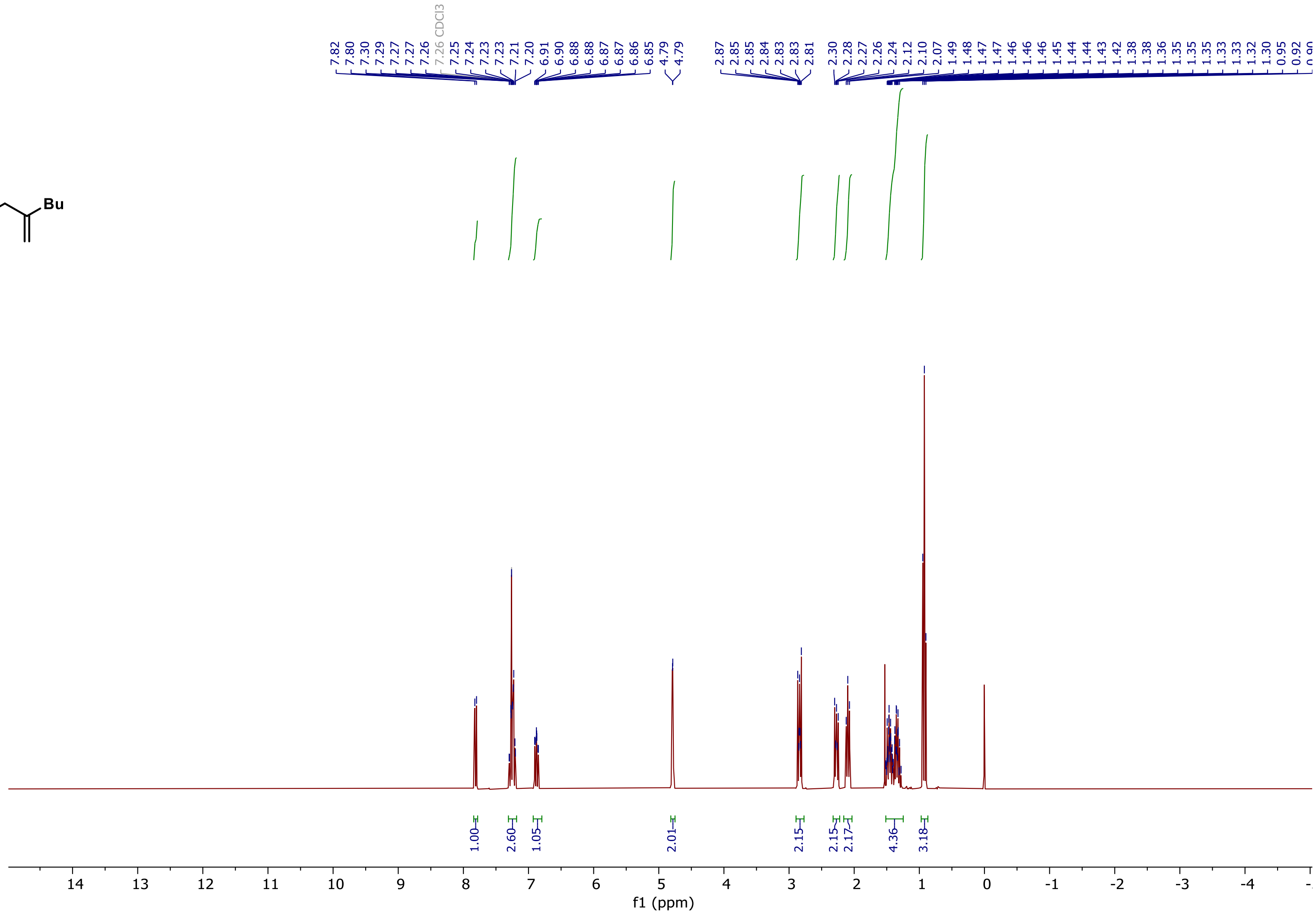

Compound 1o ^{13}C NMR

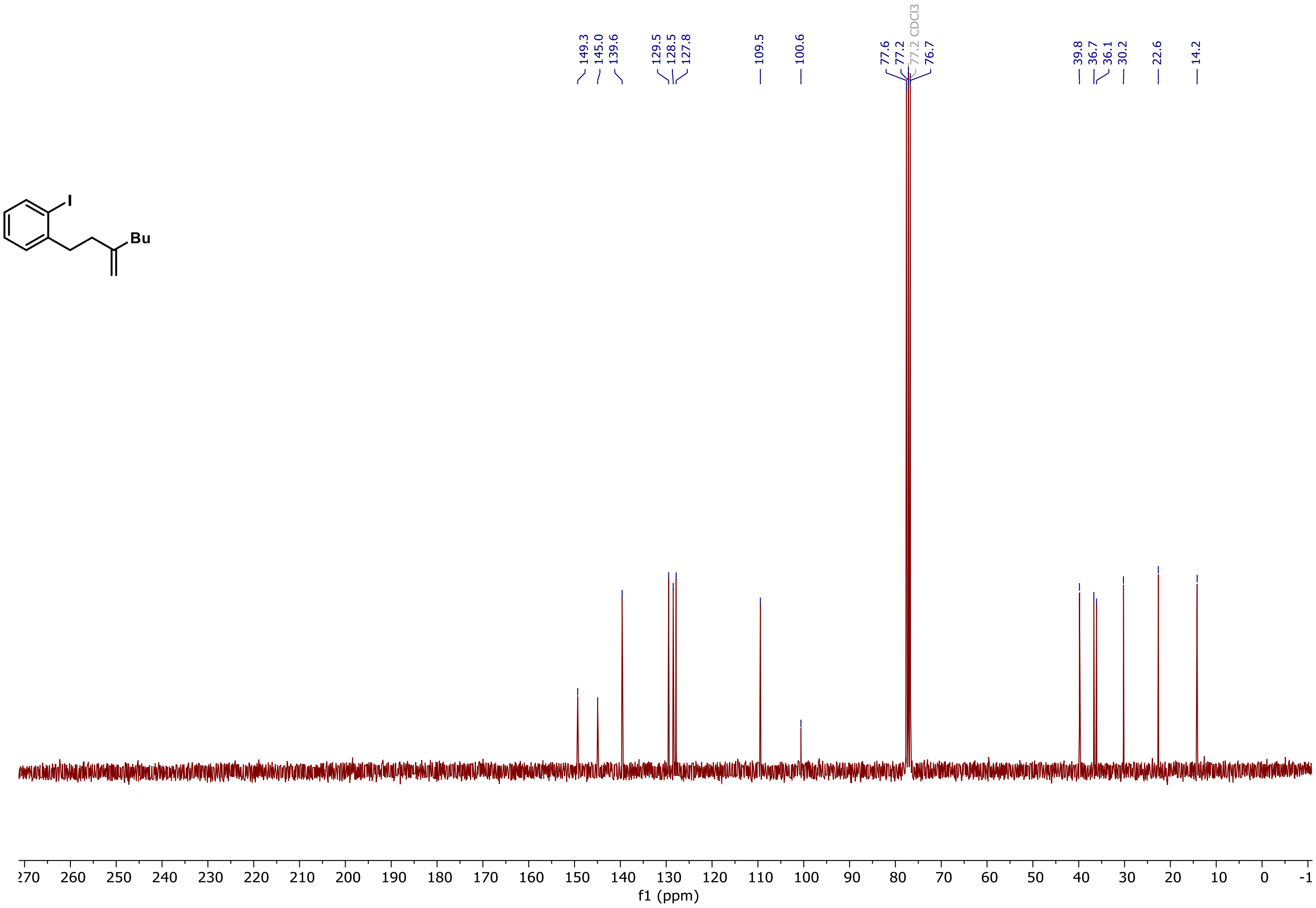

Compound 1p ¹H NMR

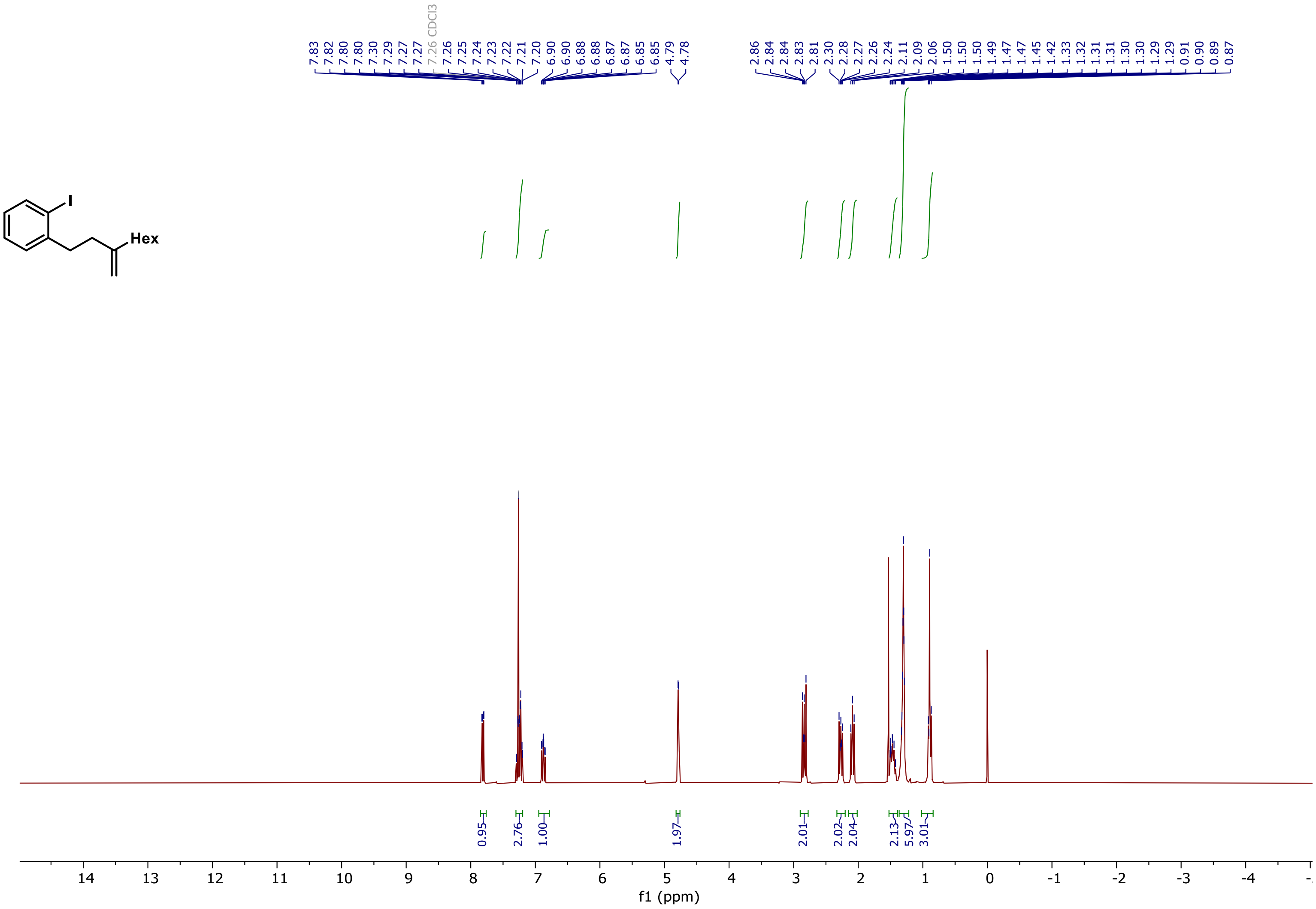

Compound 1p ^{13}C NMR

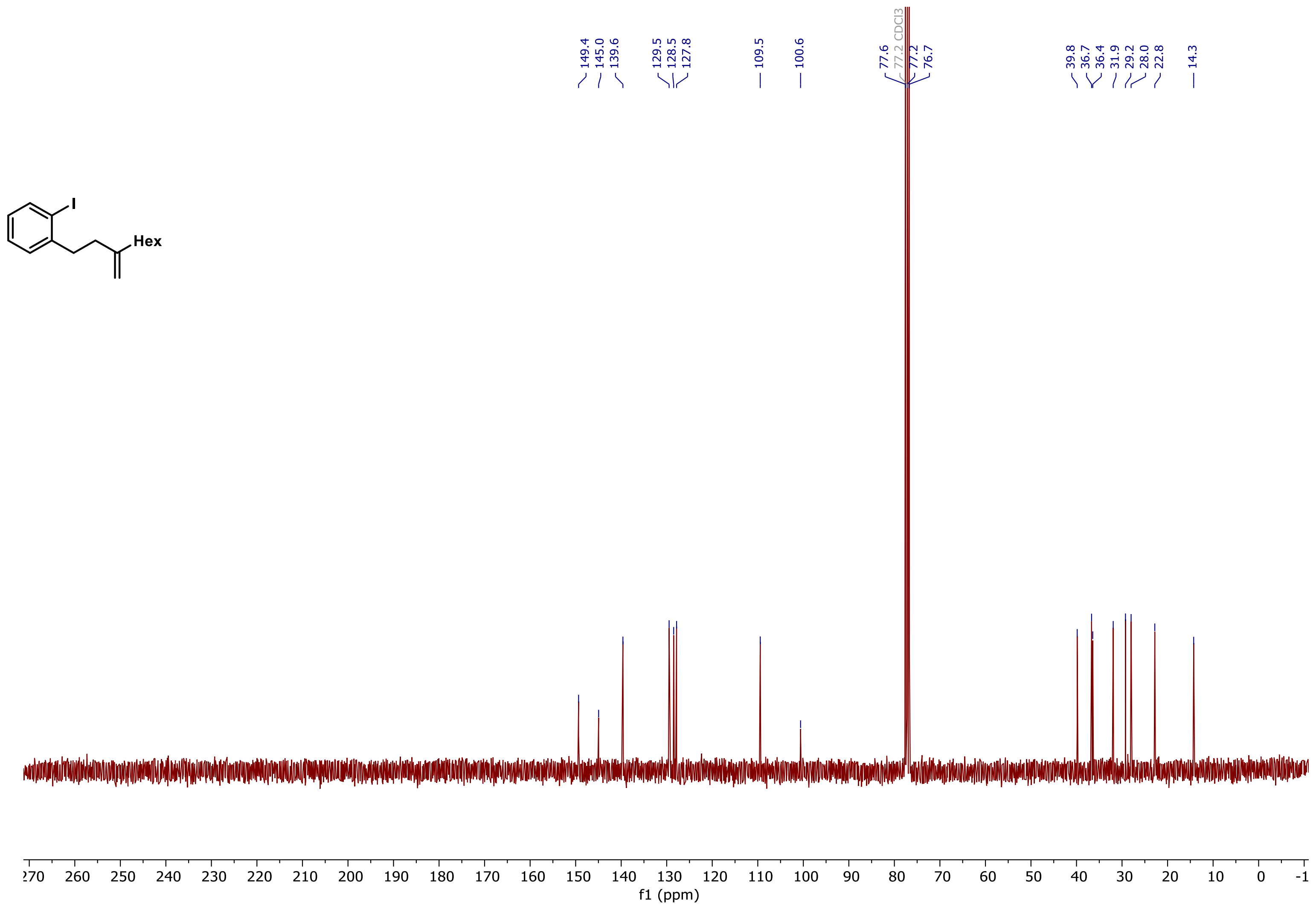

Compound 1q ^{1}H NMR

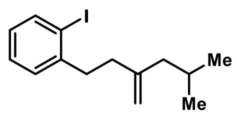

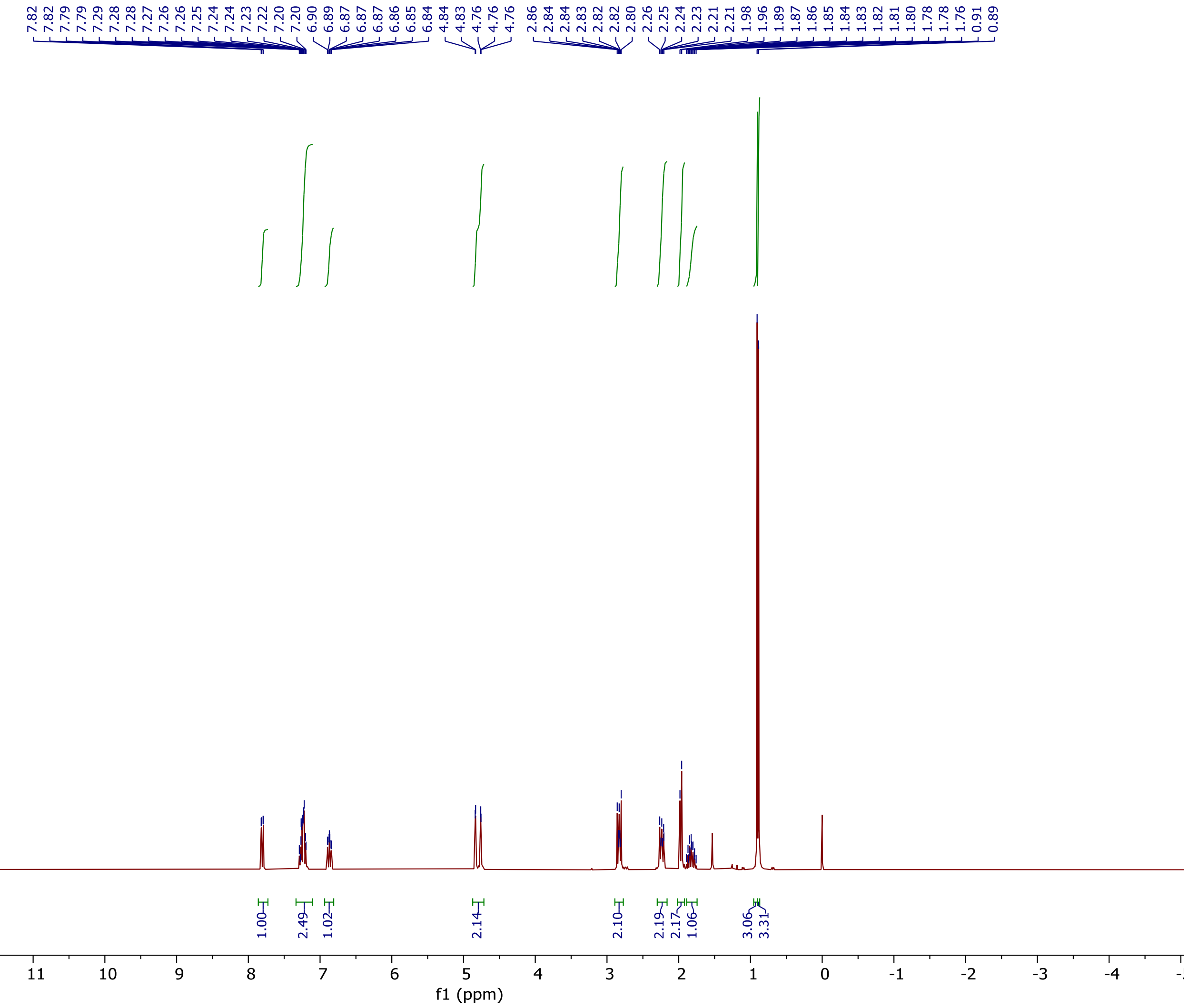

Compound 1q ^{13}C NMR

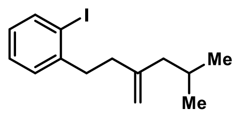

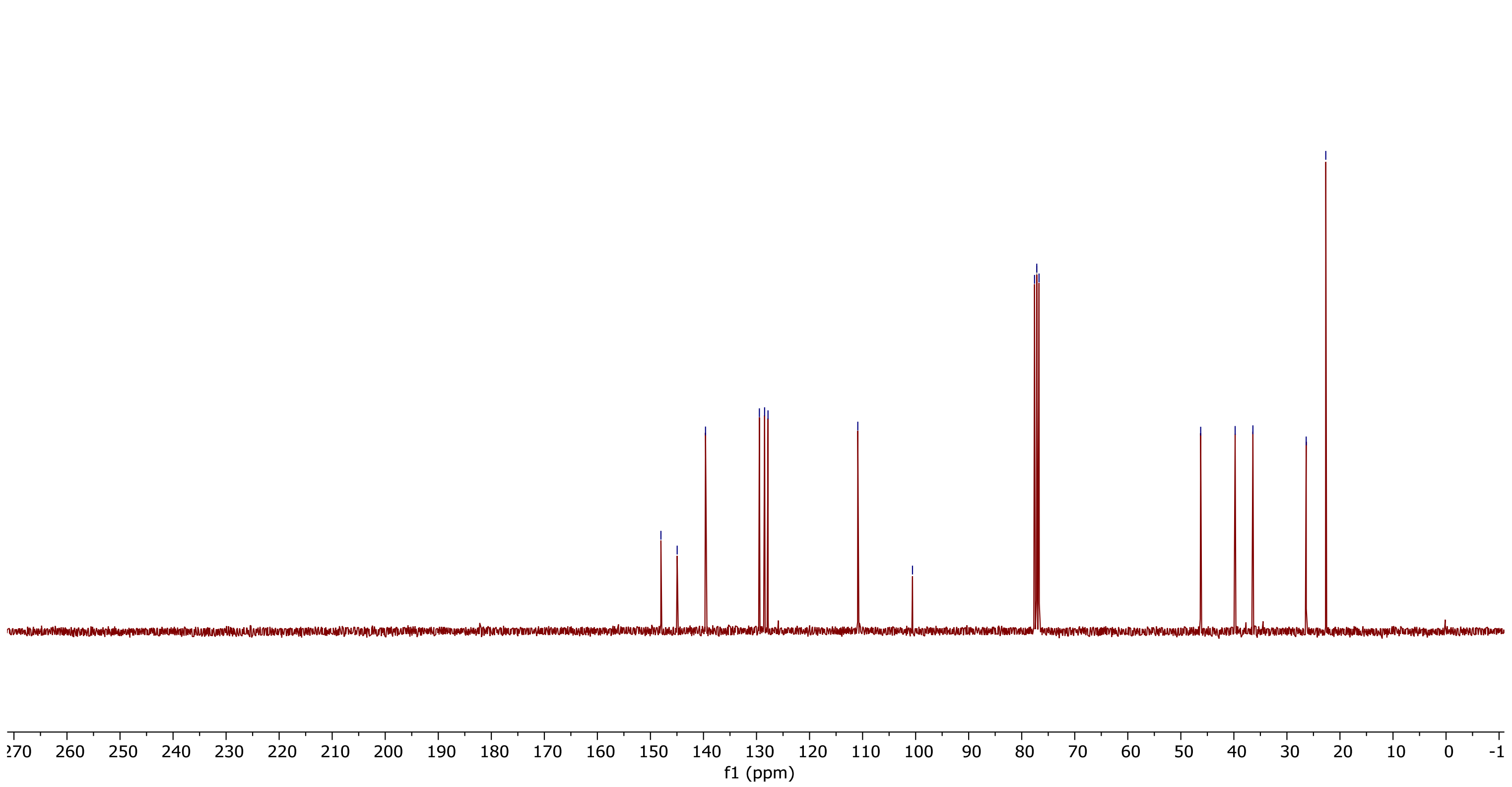

Compound 1r ¹H NMR

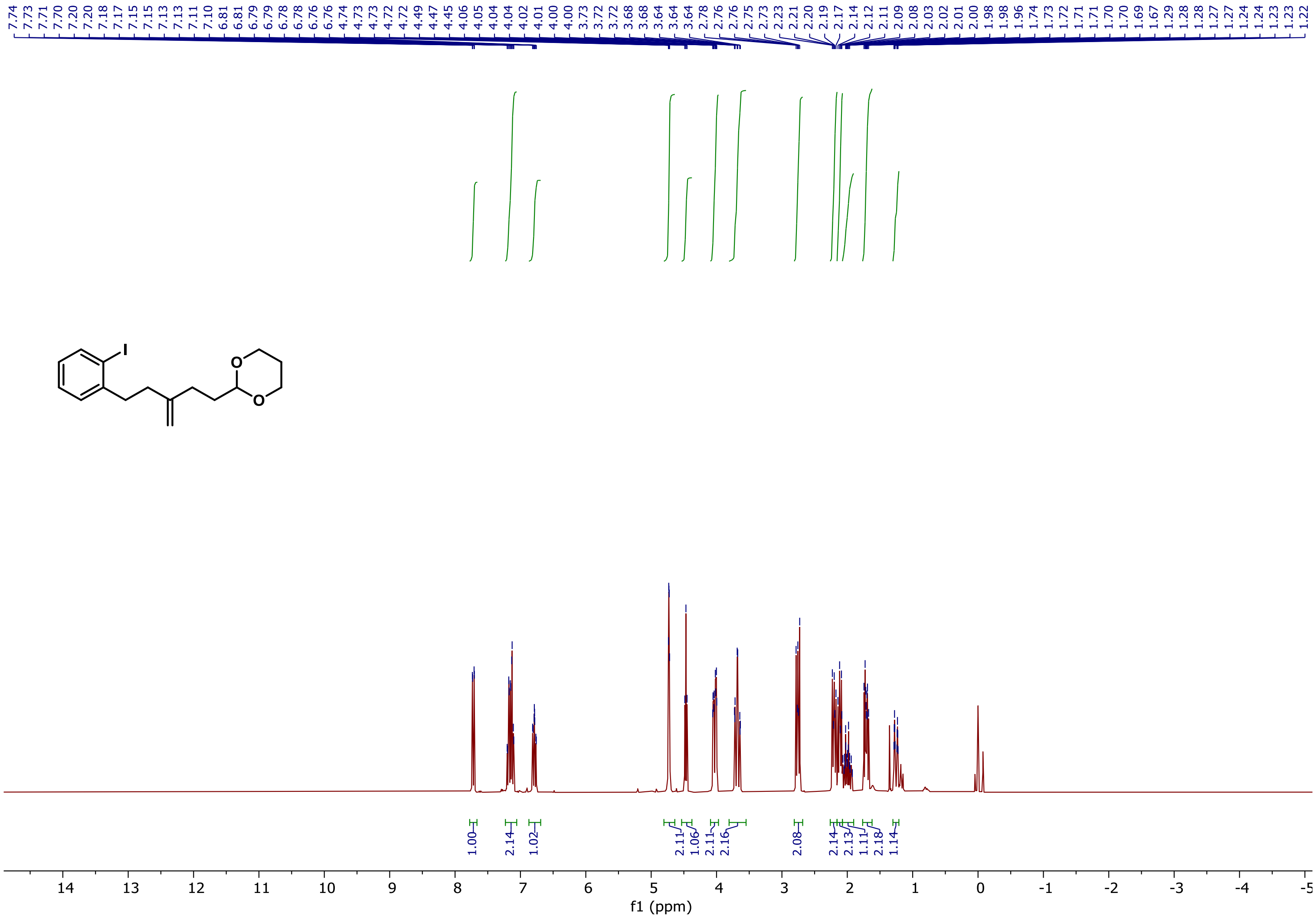

Compound 1r ^{13}C NMR

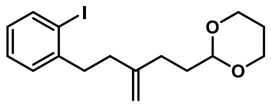

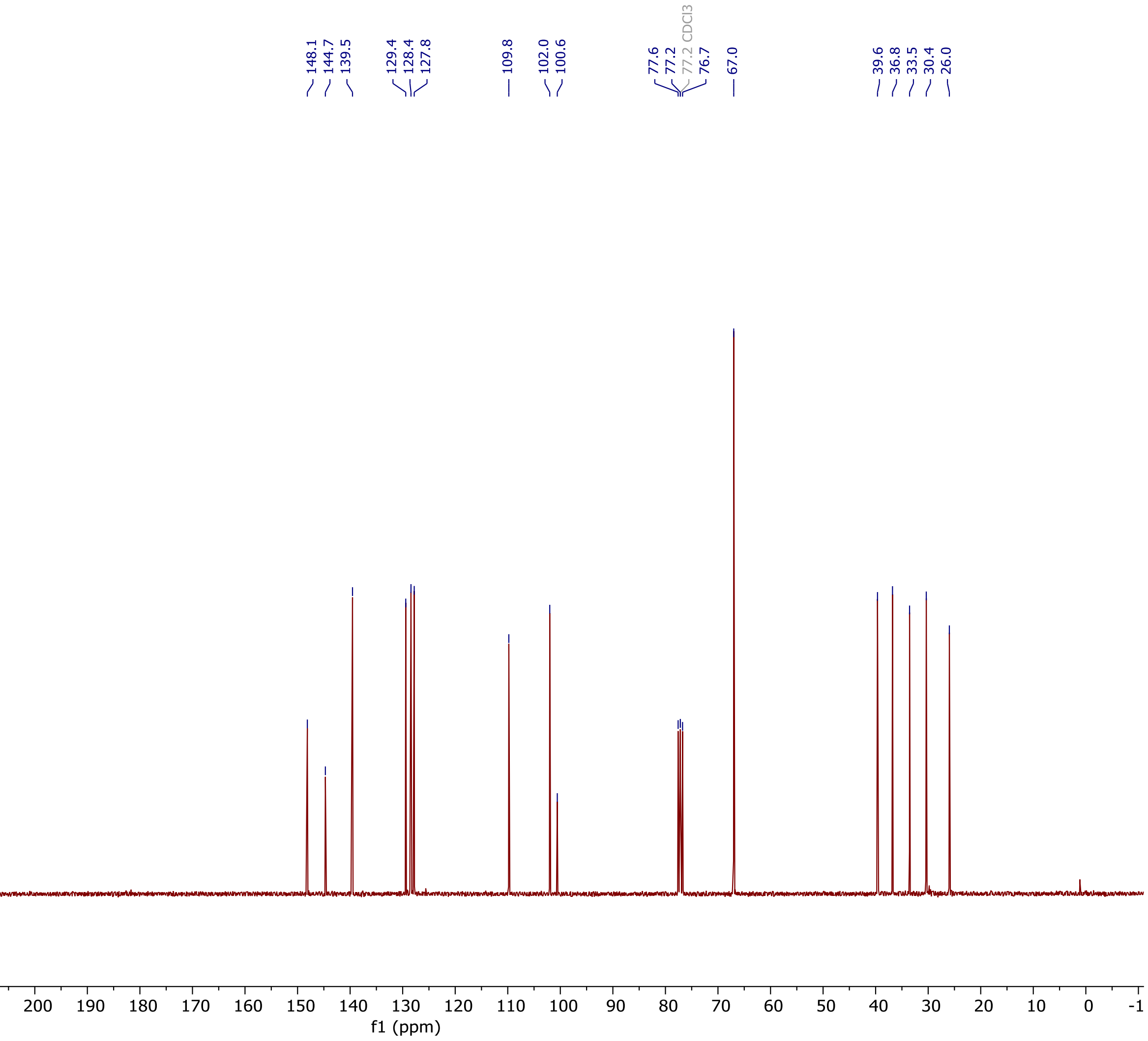

Compound 1u ¹H NMR

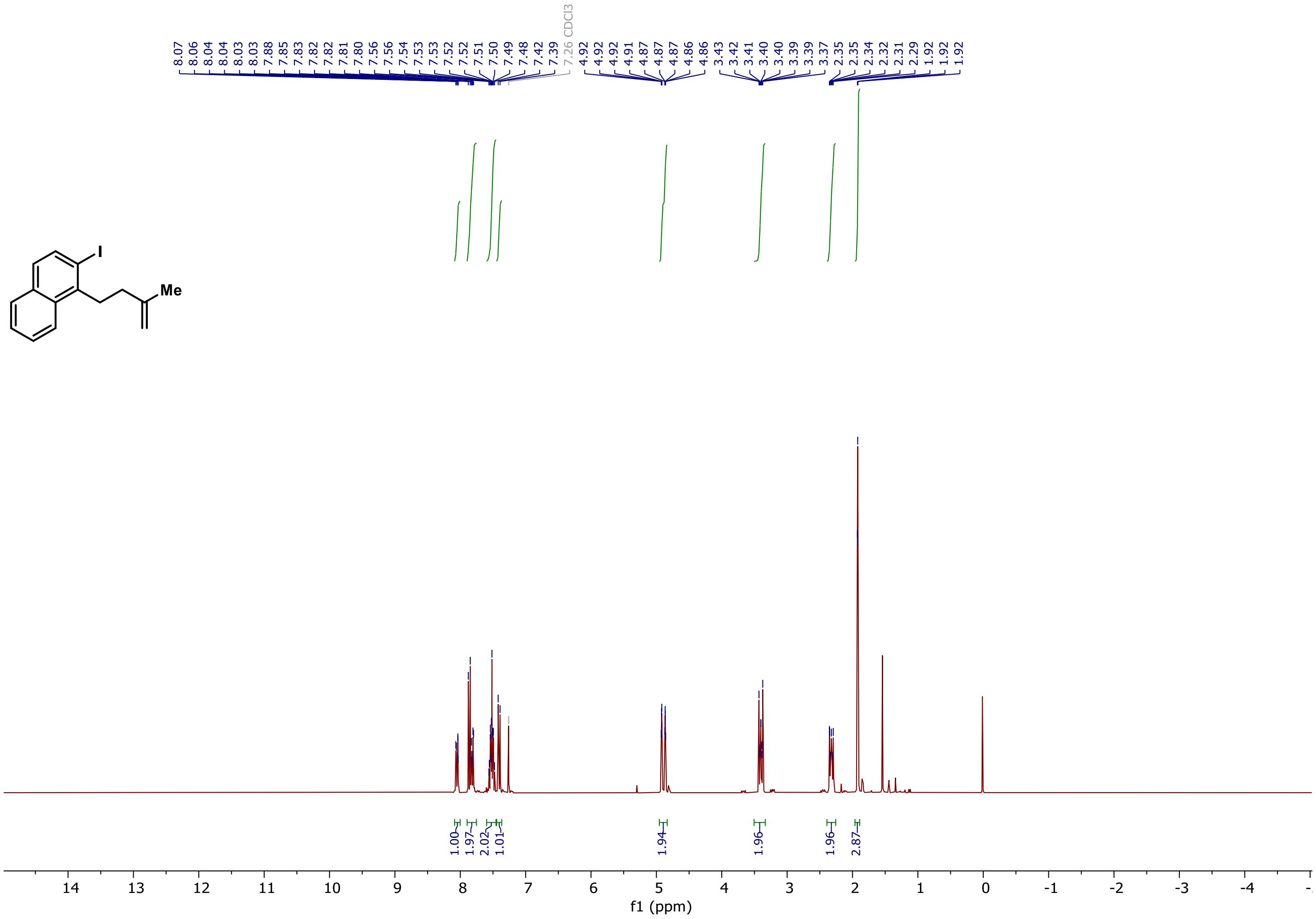

Compound 1u ^{13}C NMR

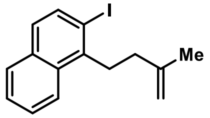

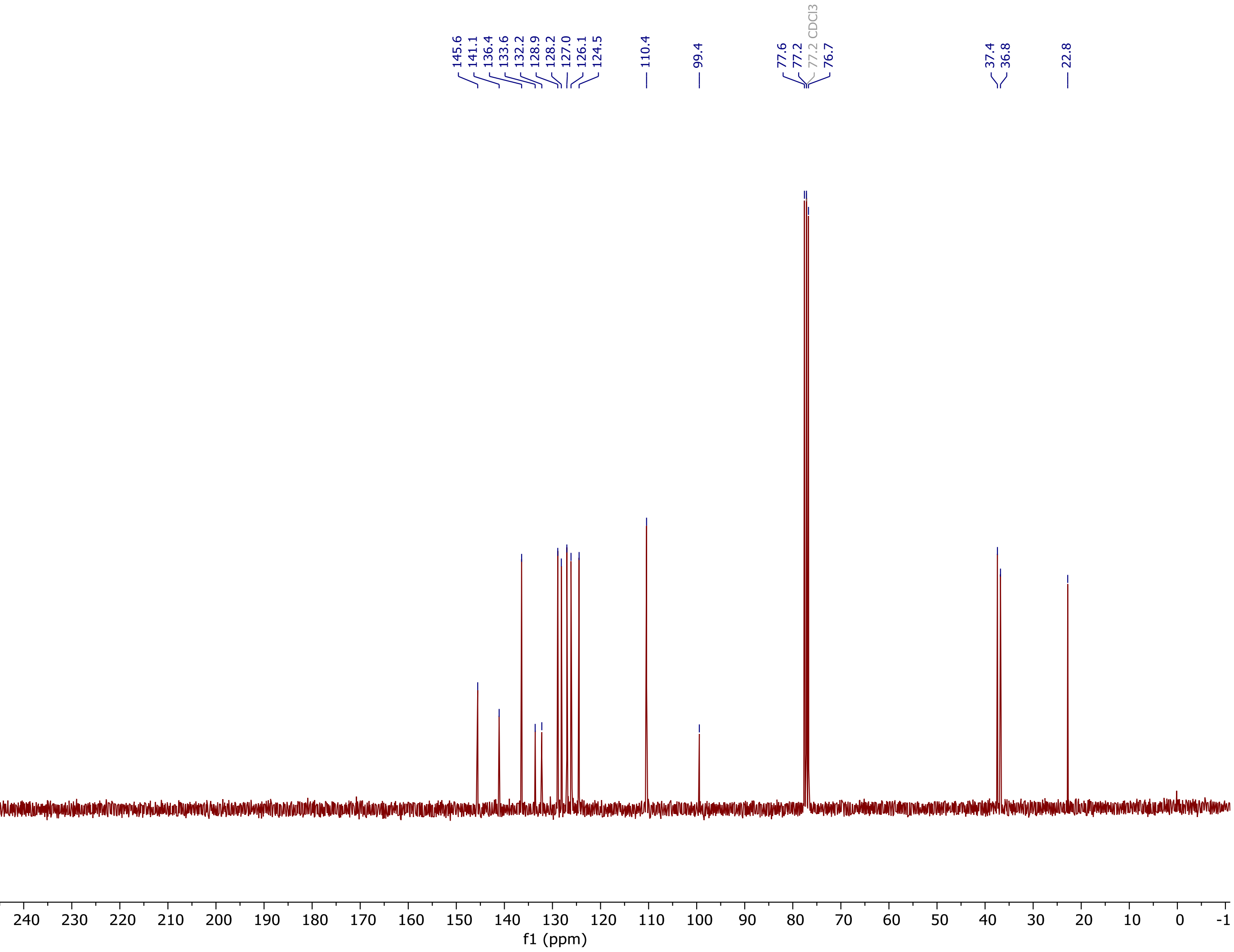

Compound 1v ¹H NMR

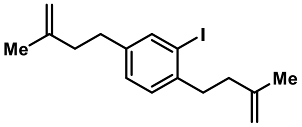

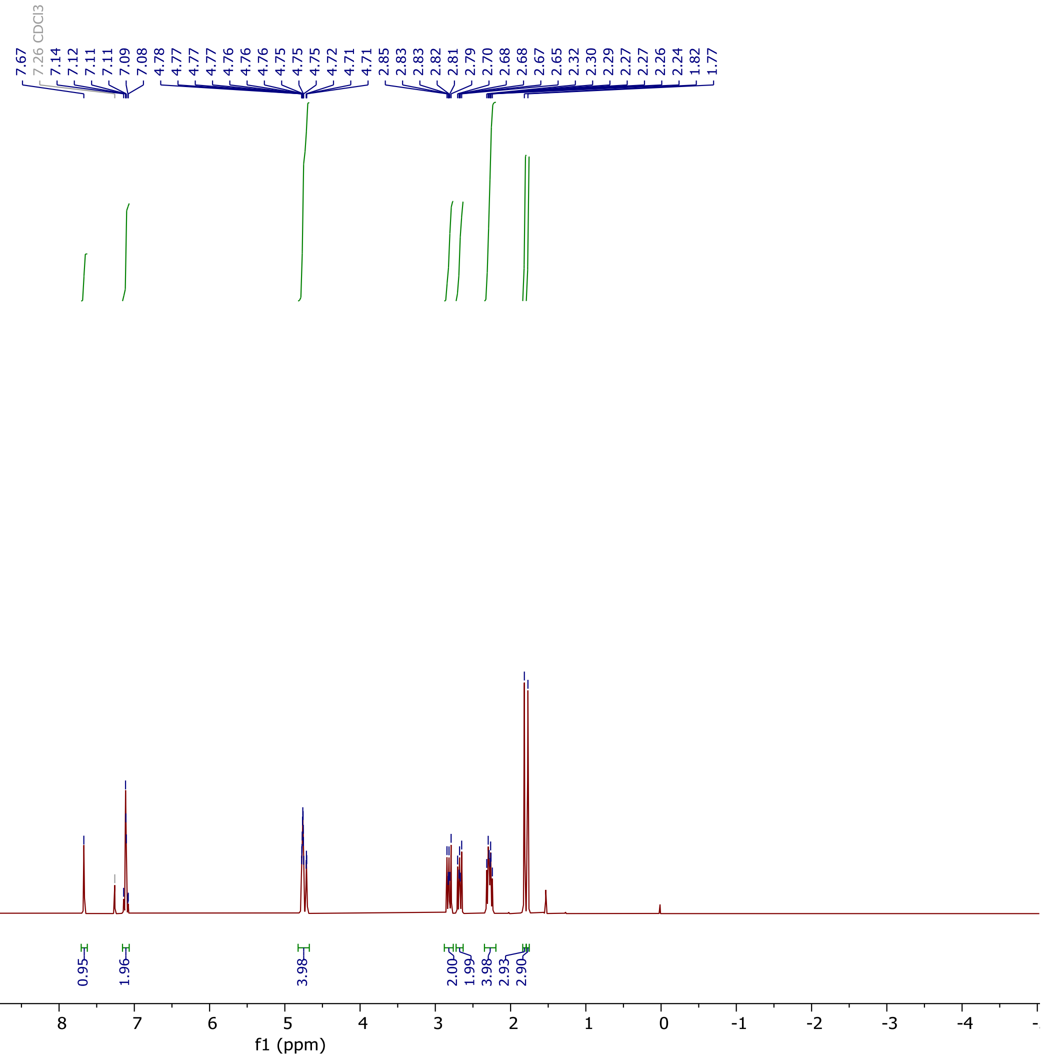

Compound 1v ^{13}C NMR

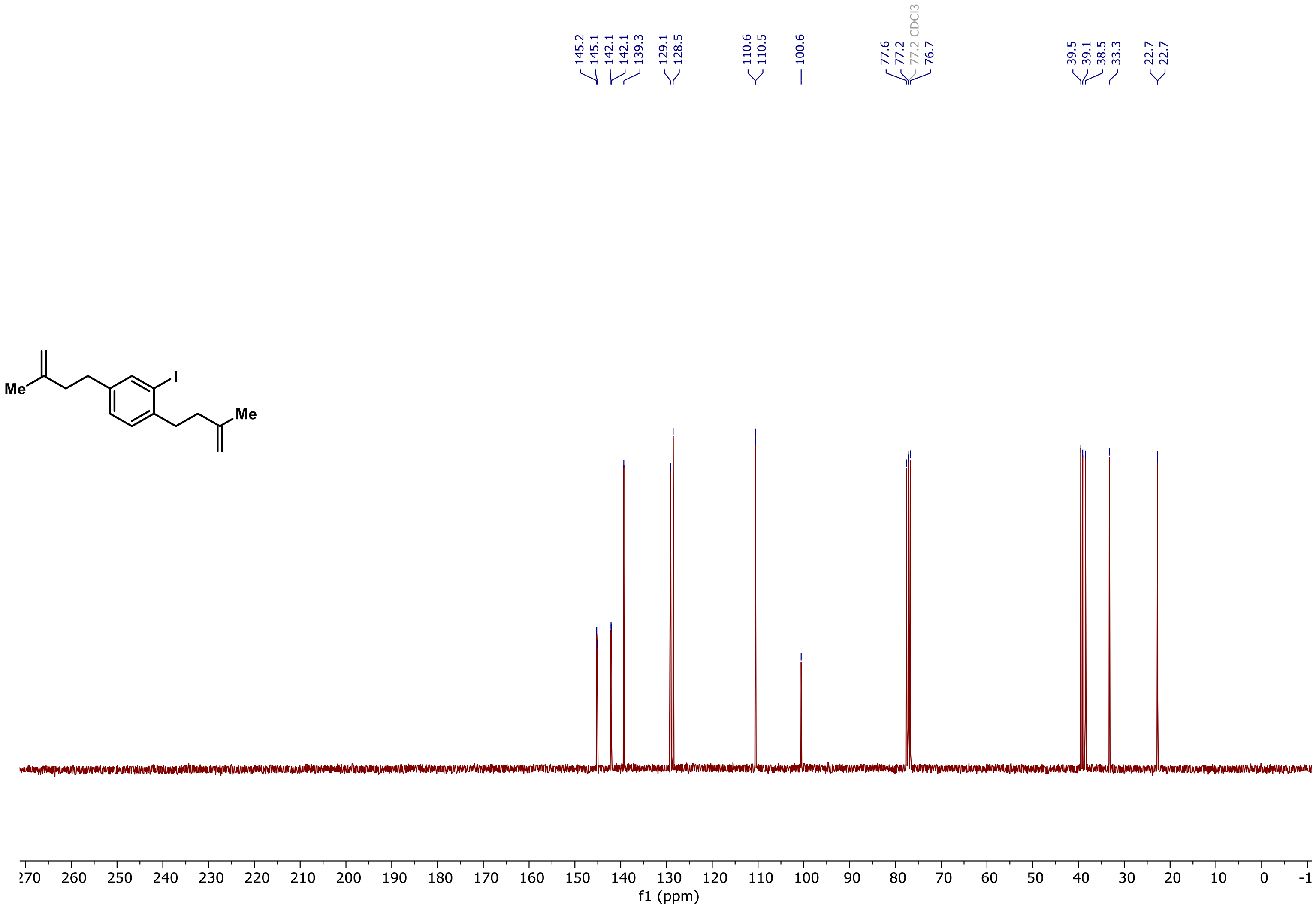

Compound 2a ¹H NMR

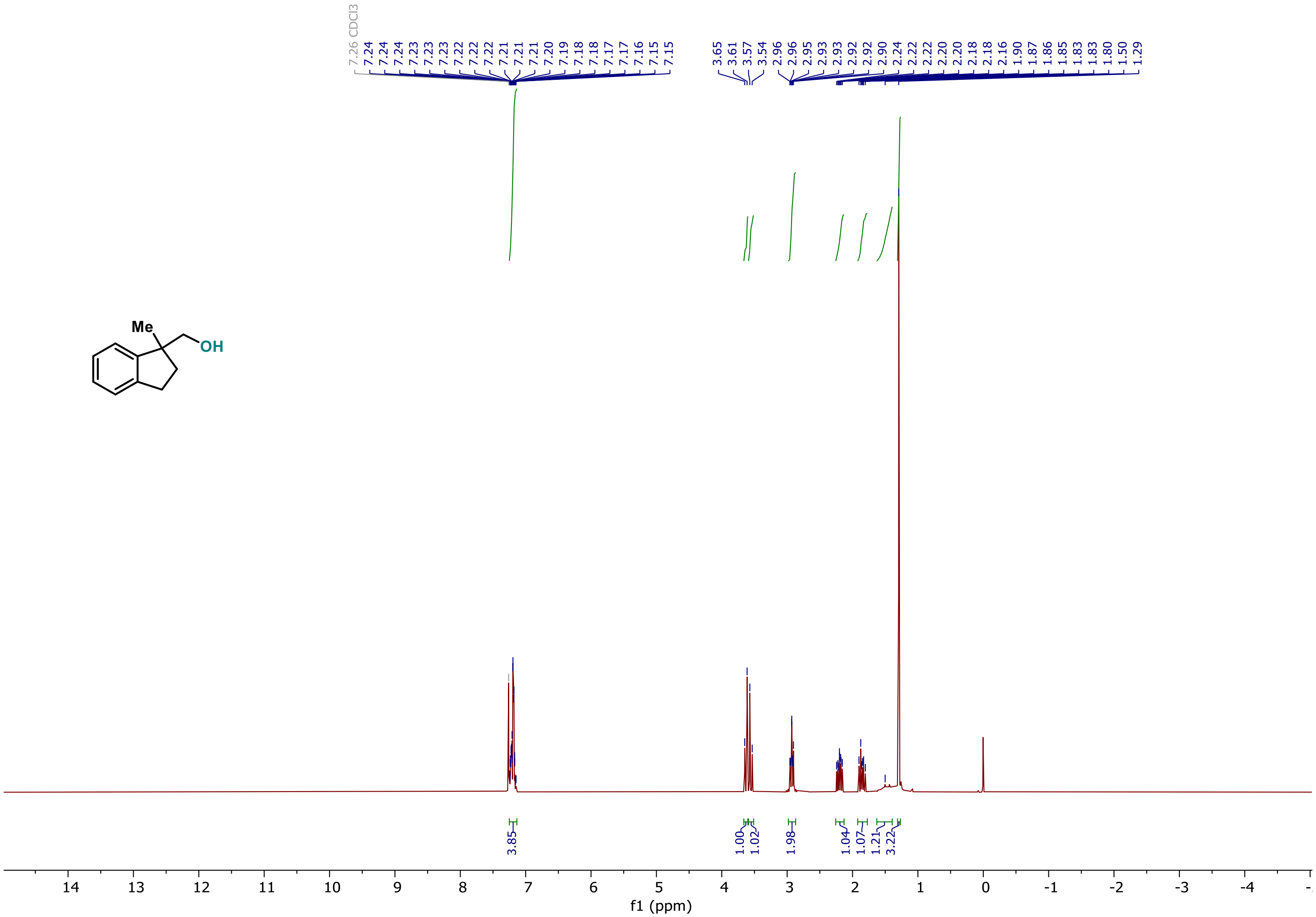

Compound 2a ^{13}C NMR

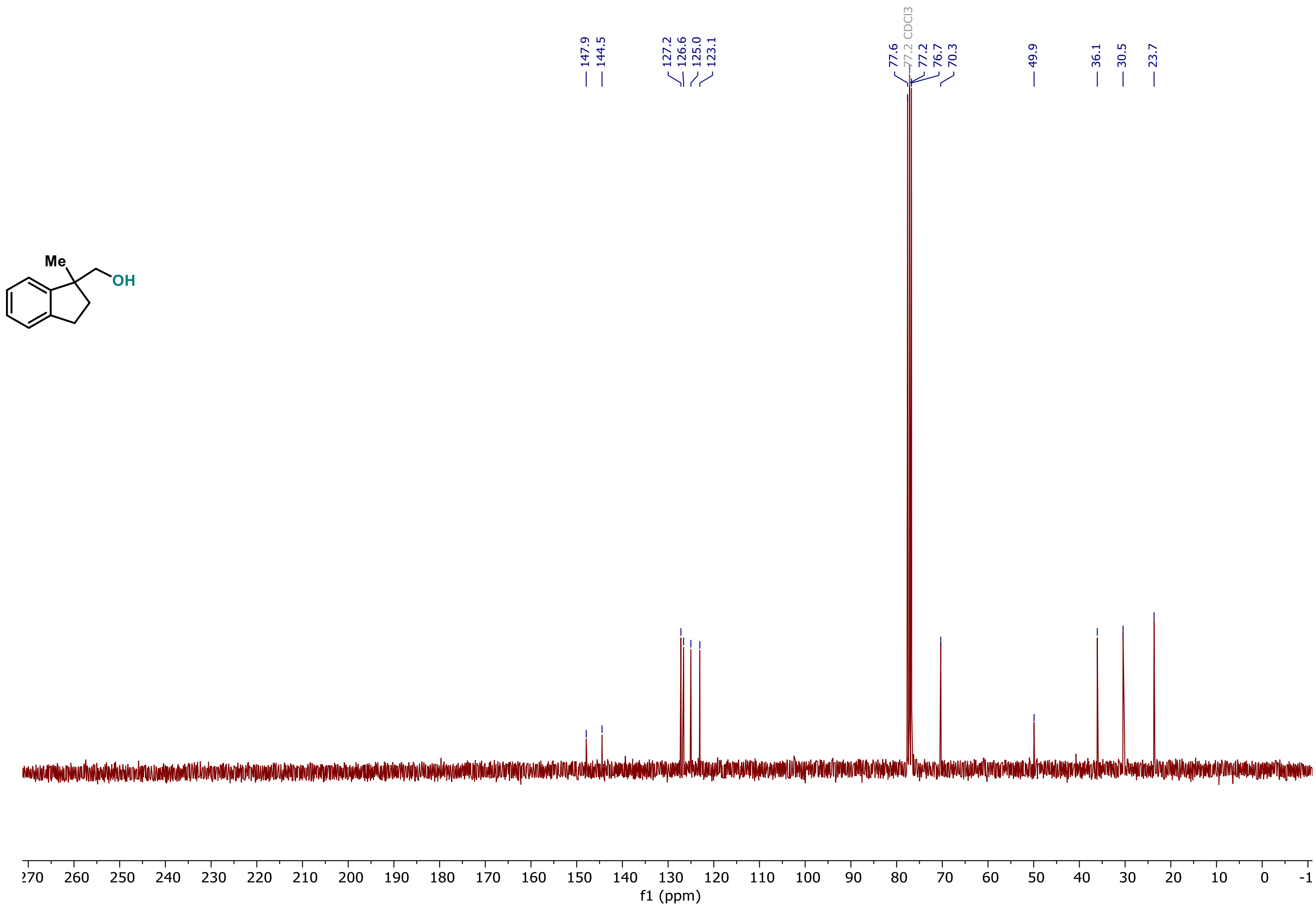

Compound 2b ^{1}H NMR

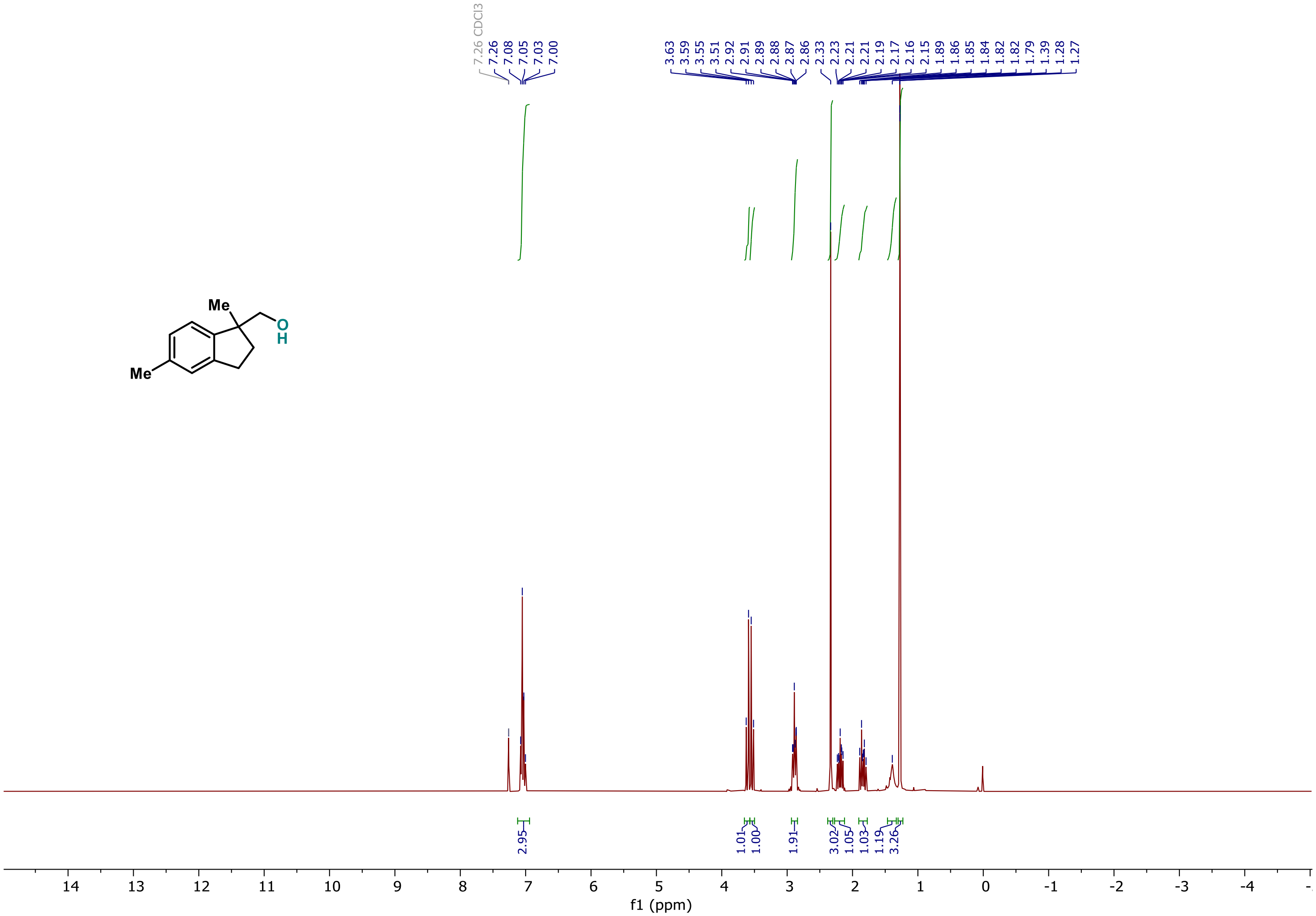

Compound 2b ^{13}C NMR

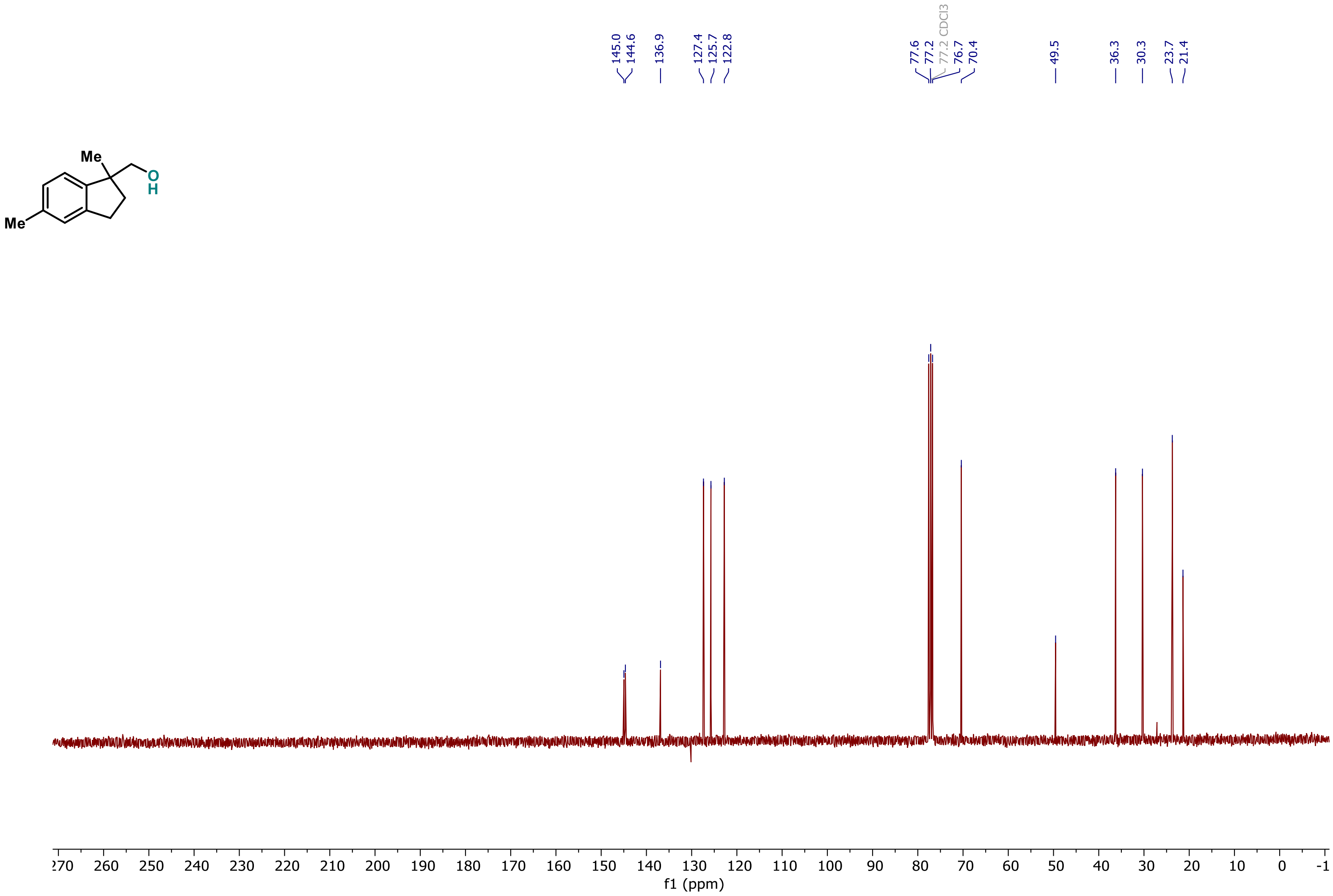

Compound 2c ¹H NMR

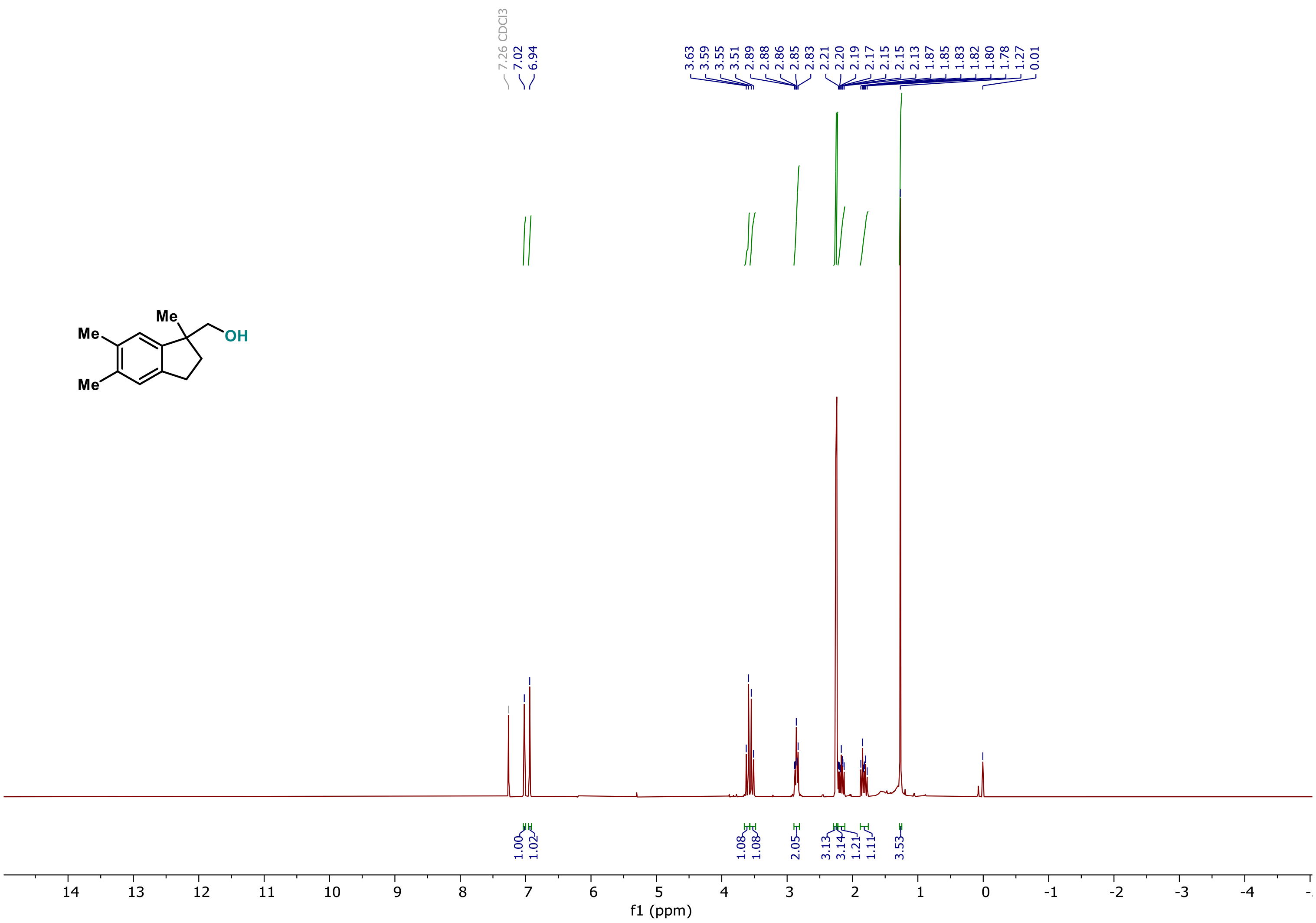

Compound 2c ^{13}C NMR

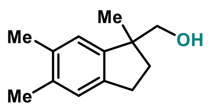

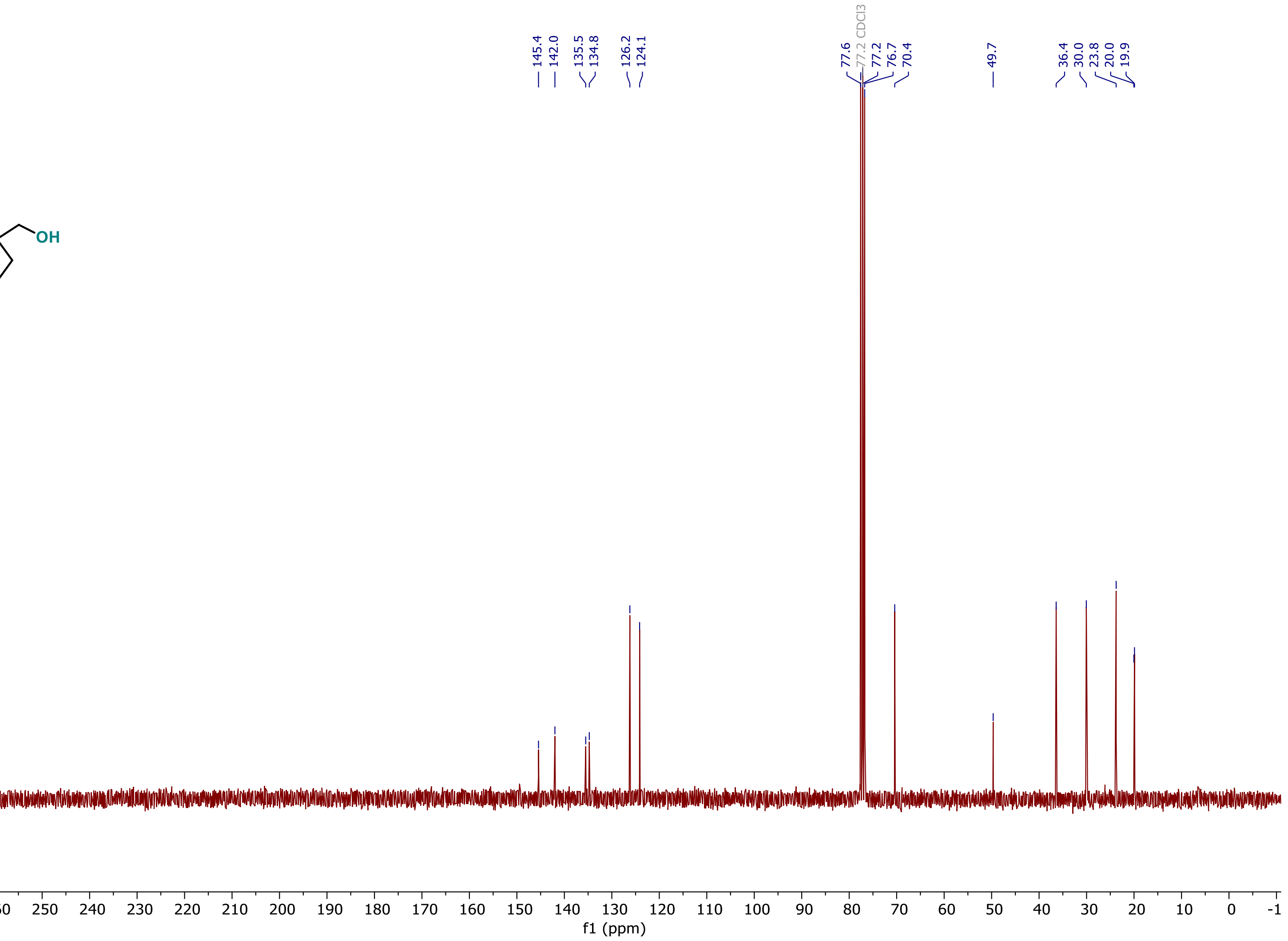

Compound 2d ¹H NMR

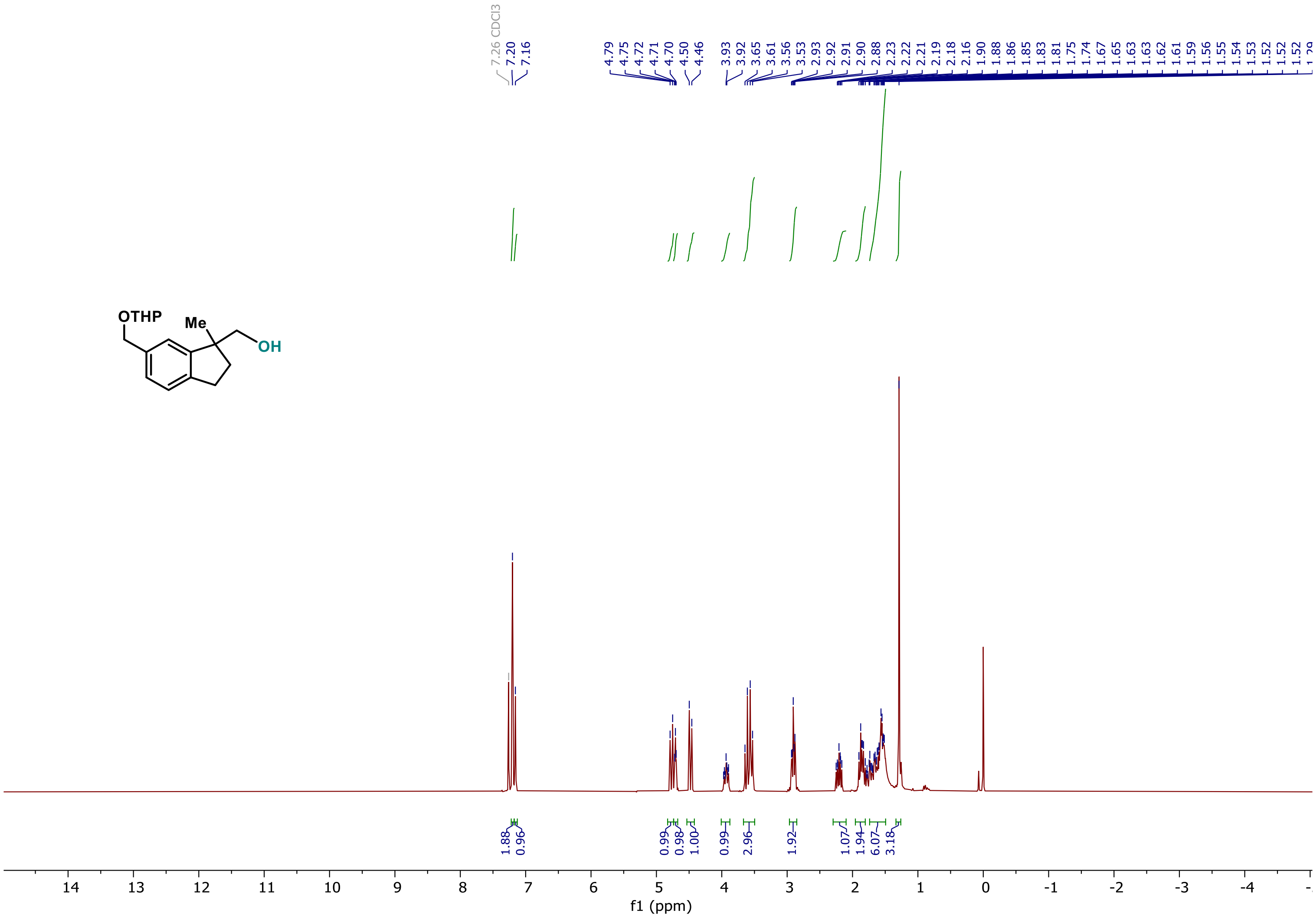

Compound 2d ^{13}C NMR

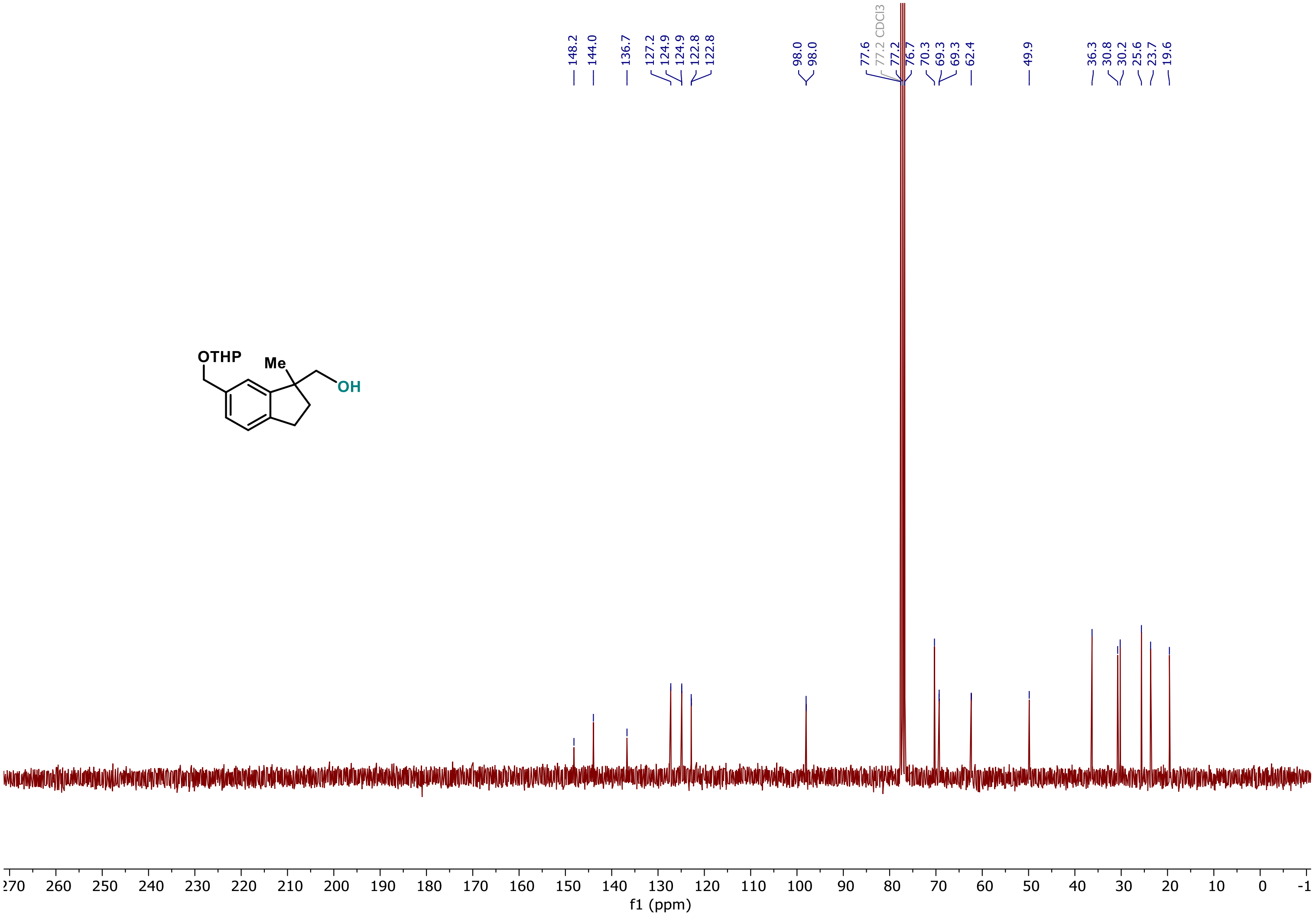

Compound 2e ^{1}H NMR

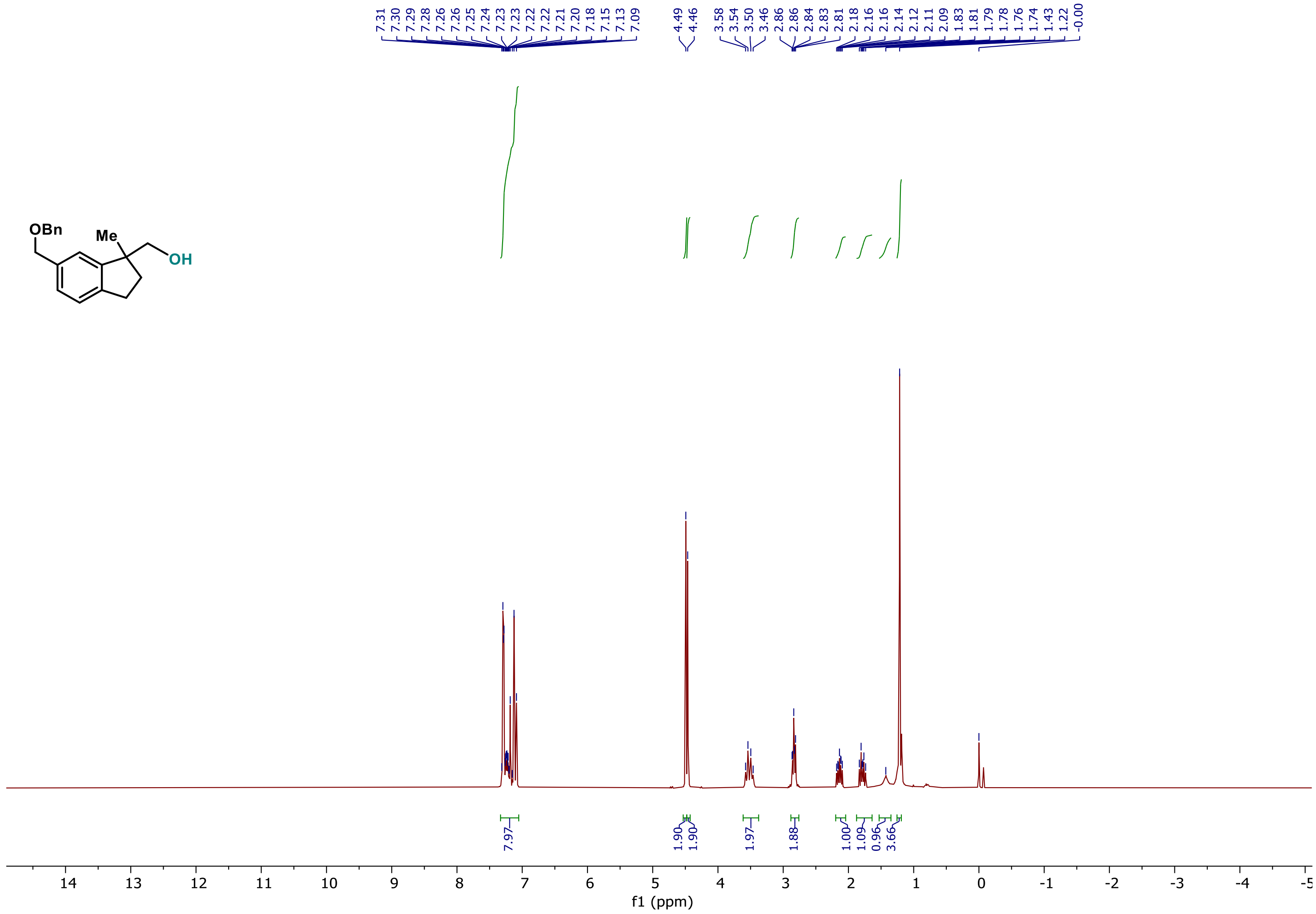

Compound 2e ^{13}C NMR

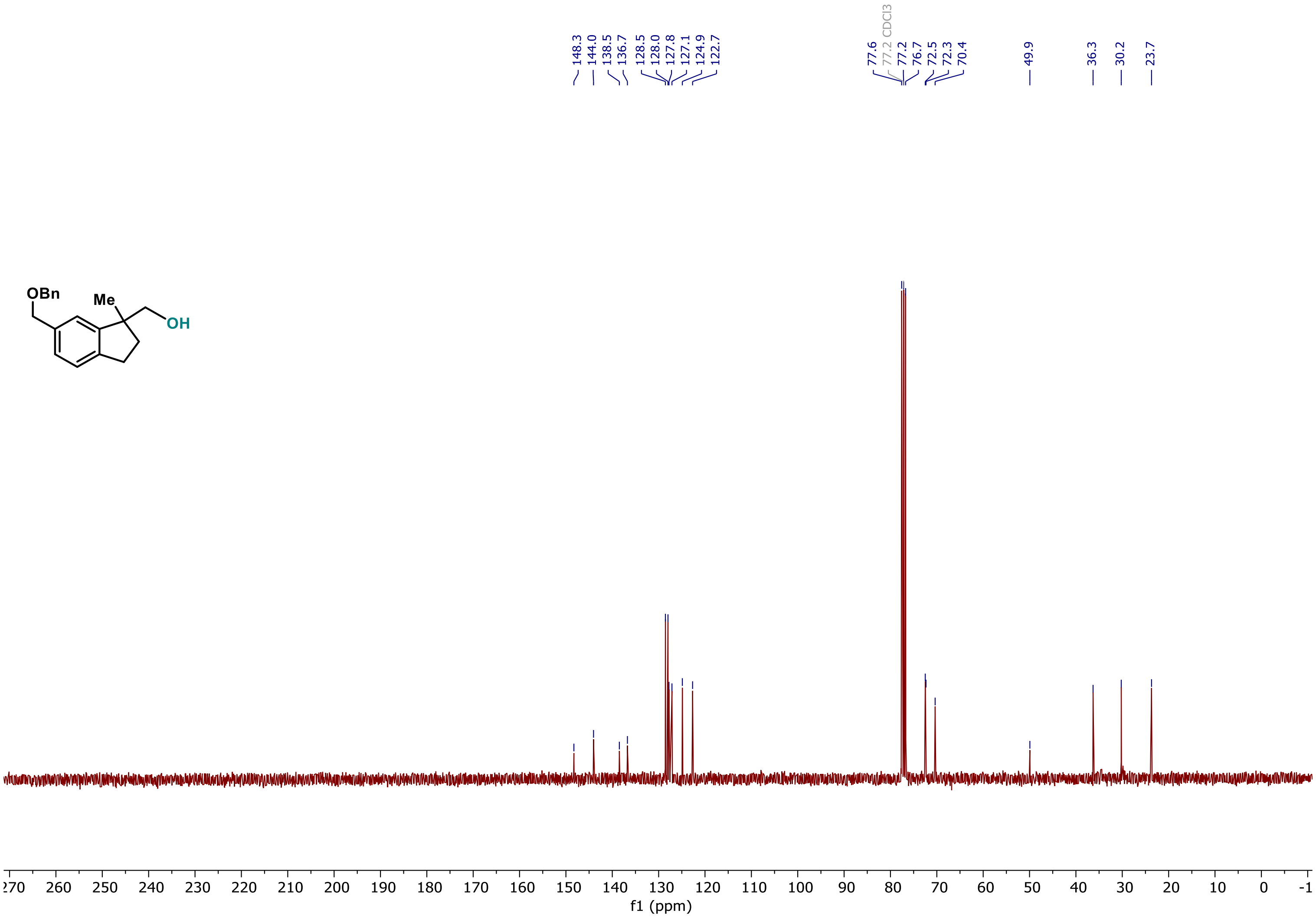

Compound 2f ¹H NMR

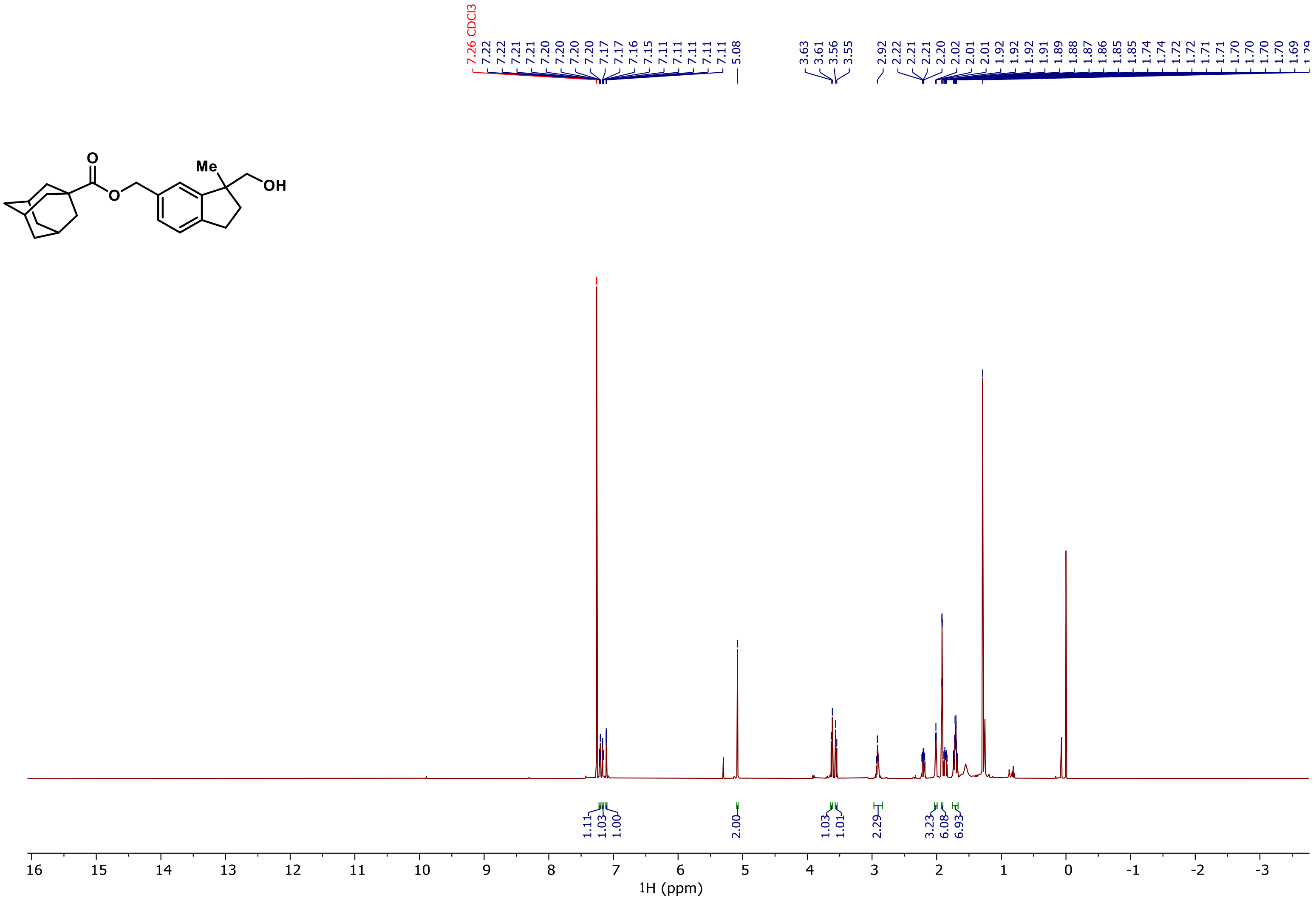

Compound 2f ^{13}C NMR

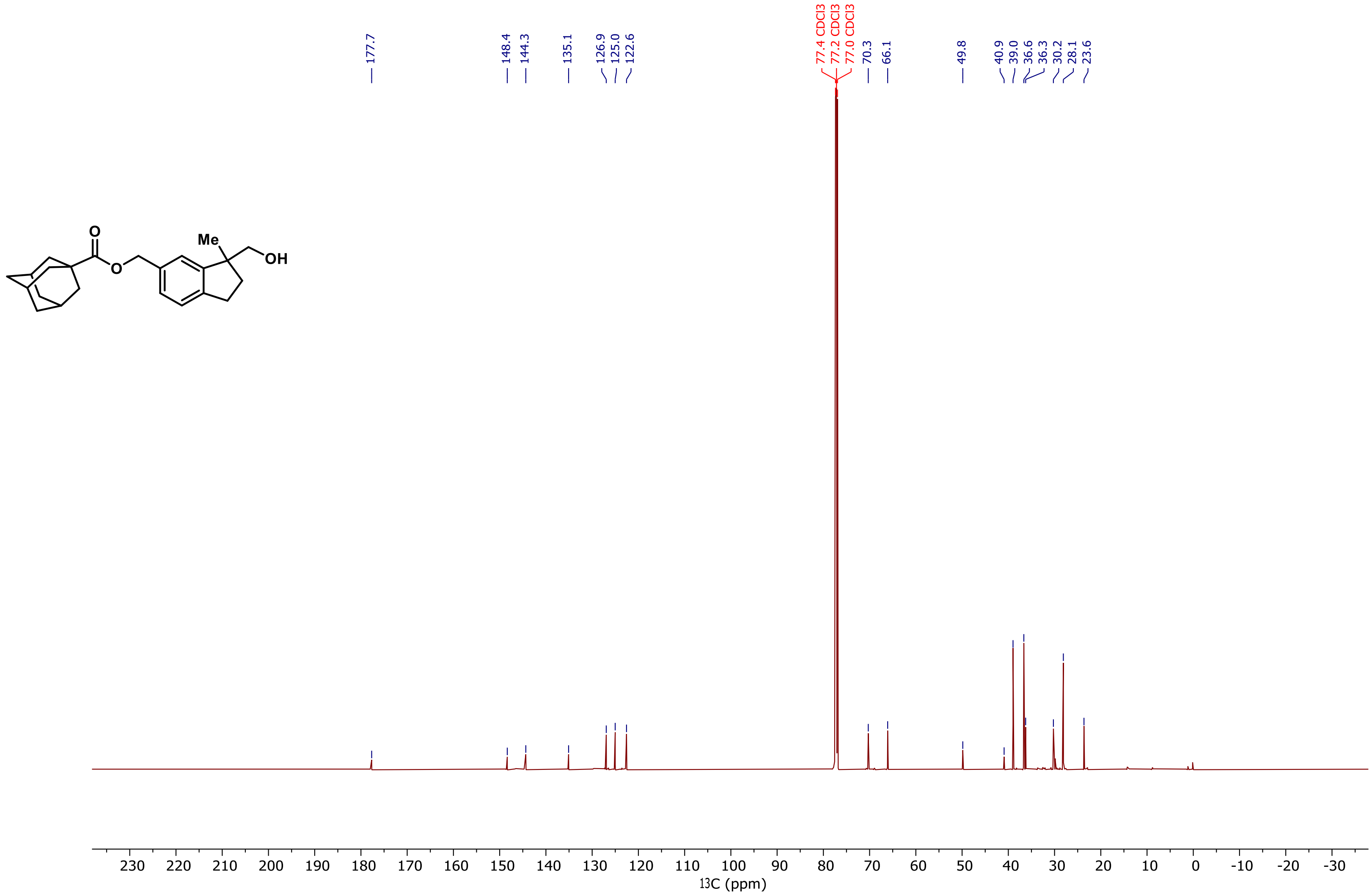

Compound 2f HSQC

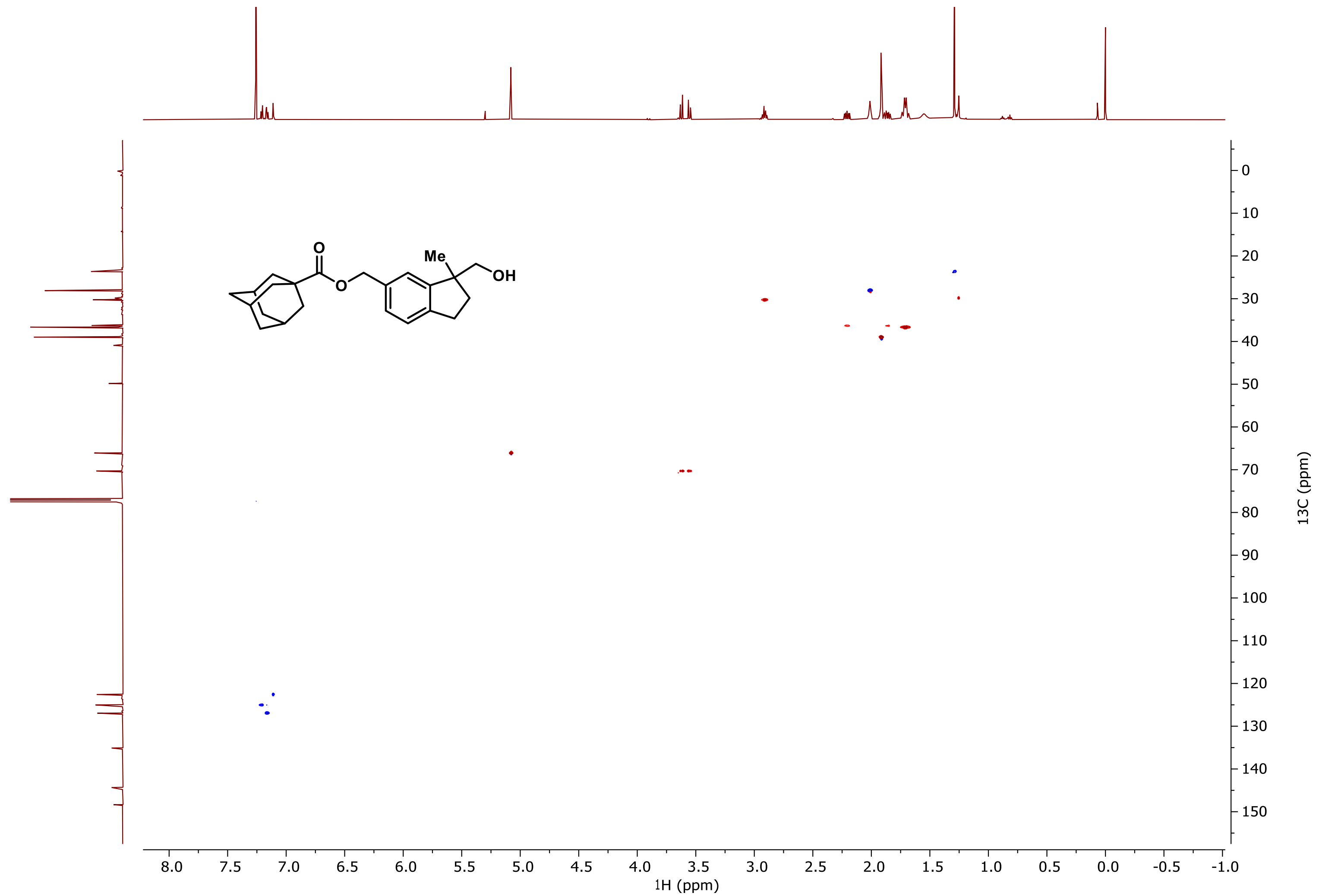

HSQC-EDITED — NIS-NA-048-01 (7233) — CDCl3; 298.0 K; 5 mg; 5 mm — 1H-13C (hsqcedetgpsisp2.3) — AV600neo (cryoBBO) — 12.11.21 21:30:03 — hsqcedetgpsisp2.3

Compound 2f COSY

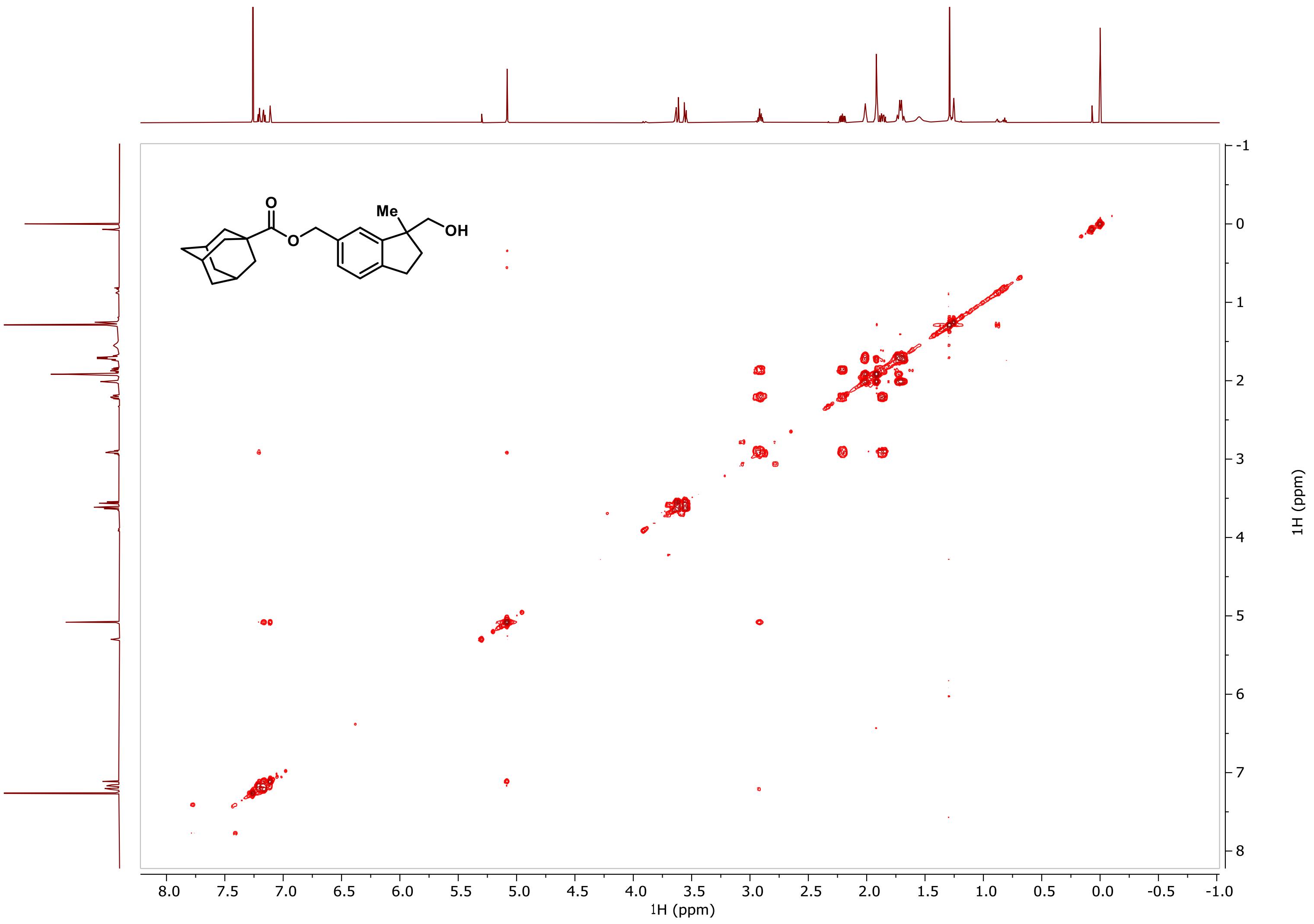

Compound 2f HMBC

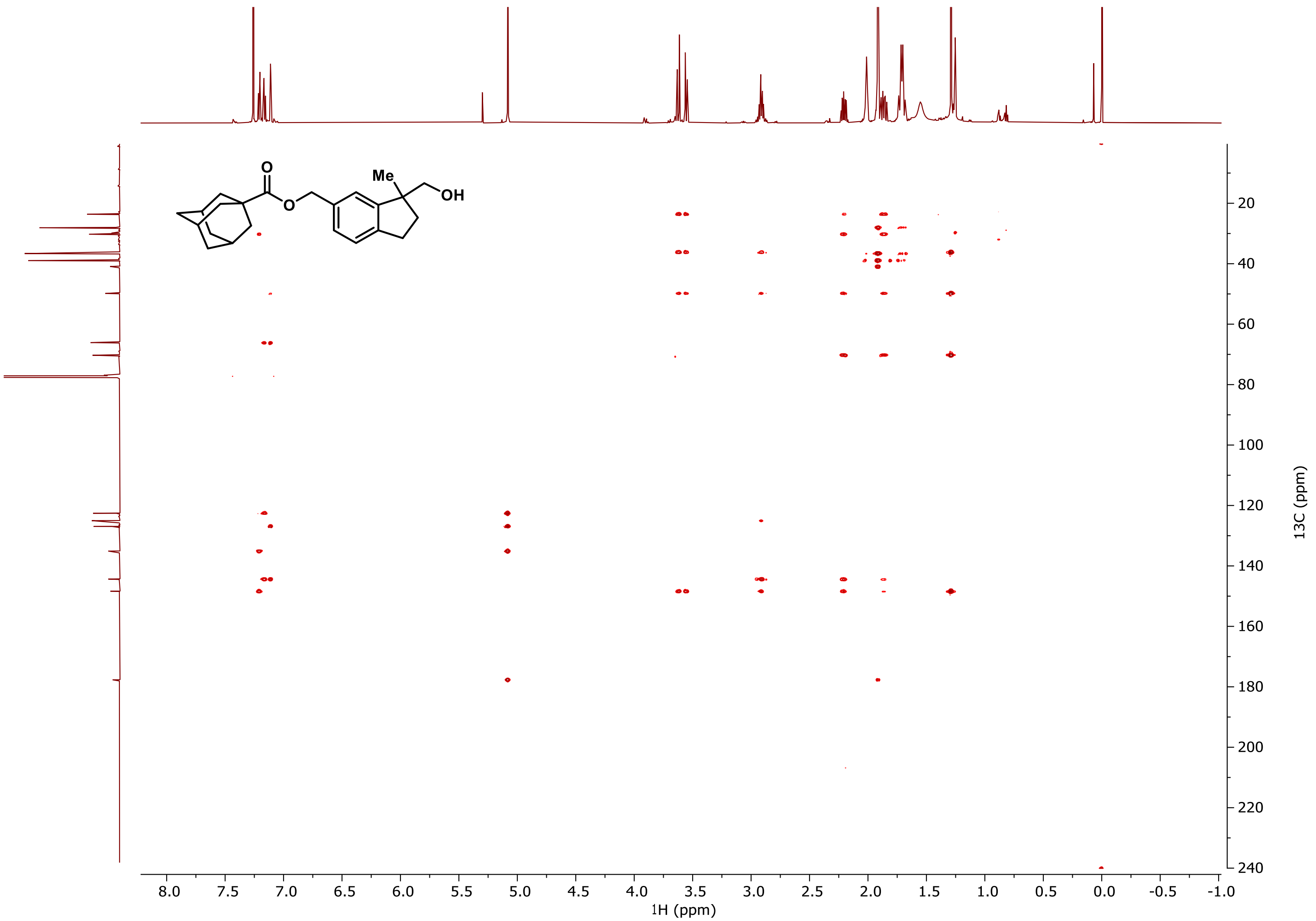

Compound 2g ¹H NMR

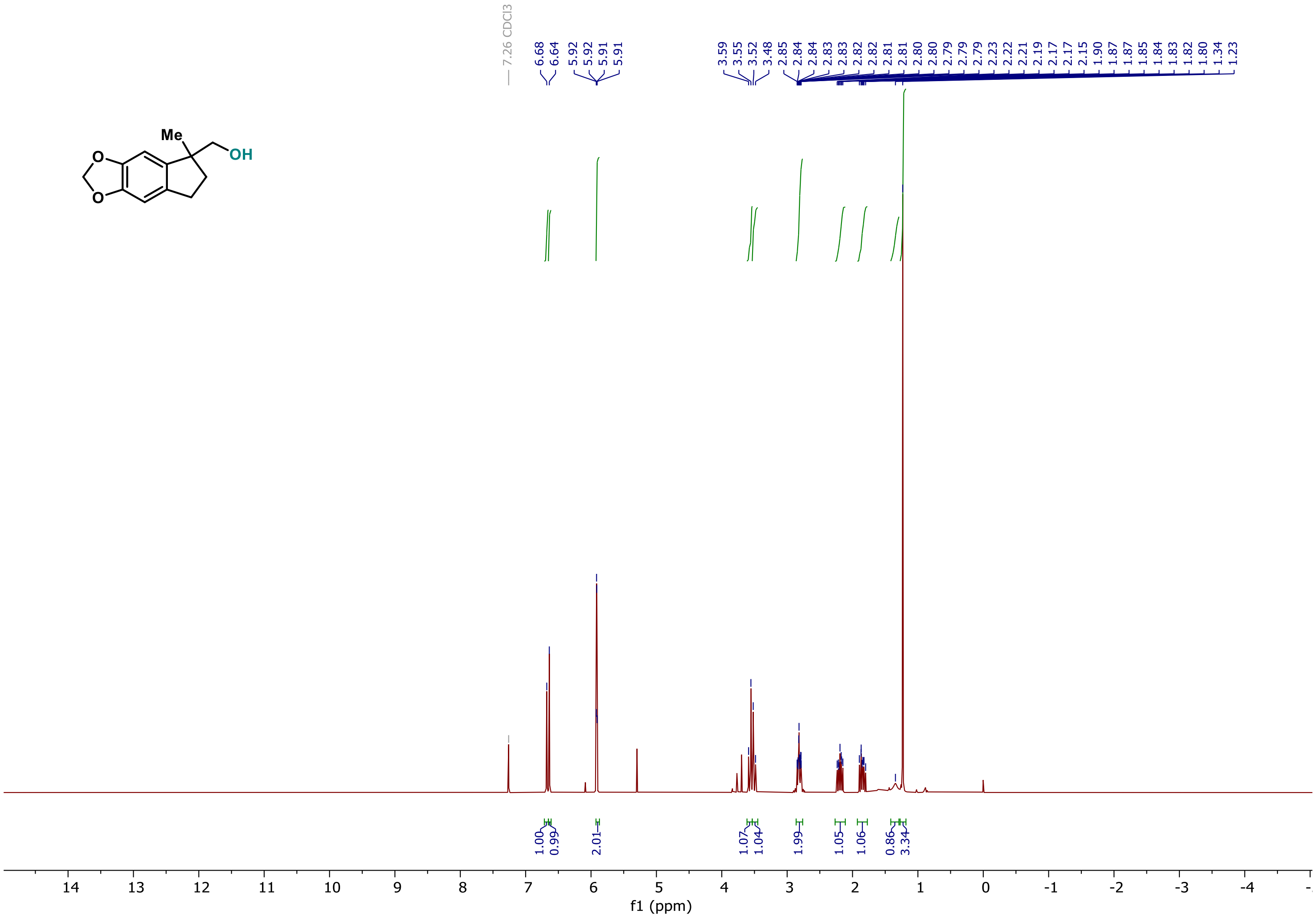

Compound 2g ^{13}C NMR

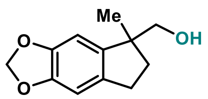

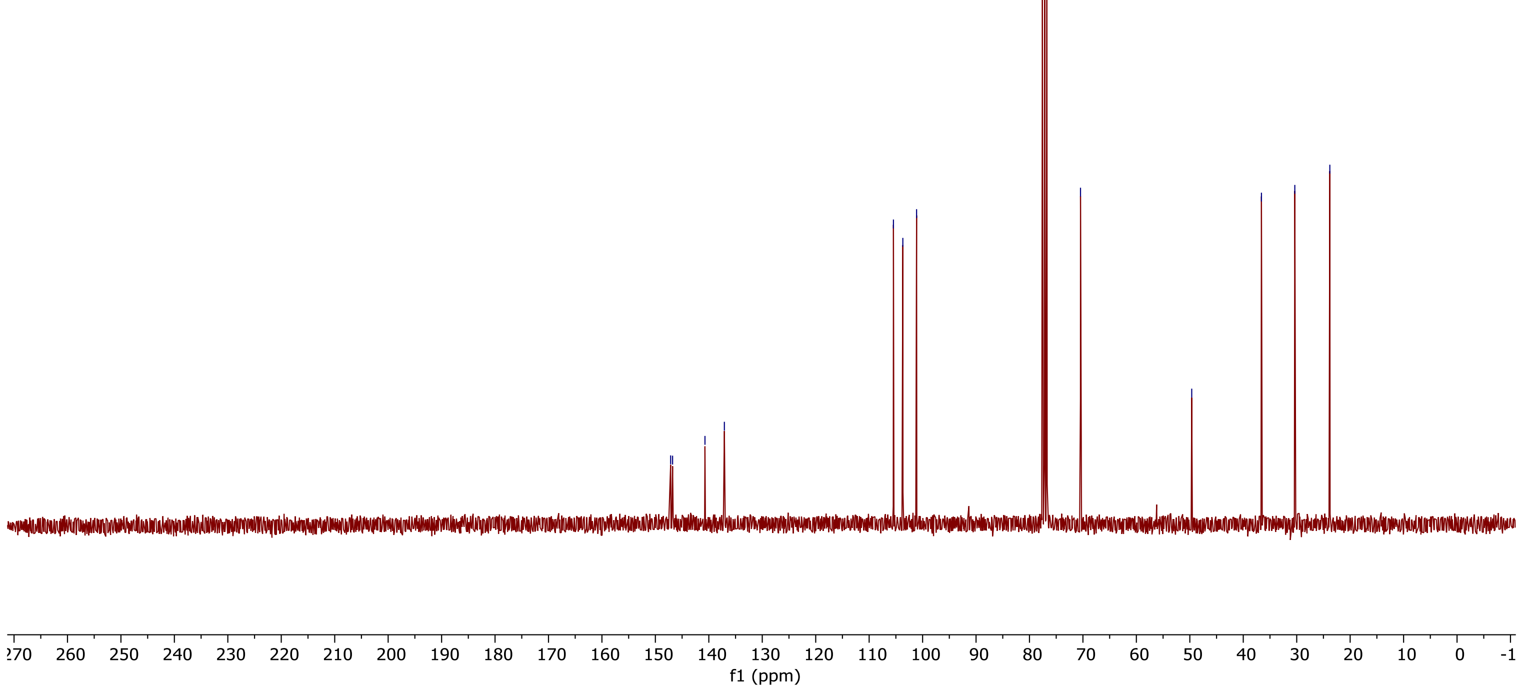

Compound 2h ^{1}H NMR

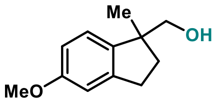

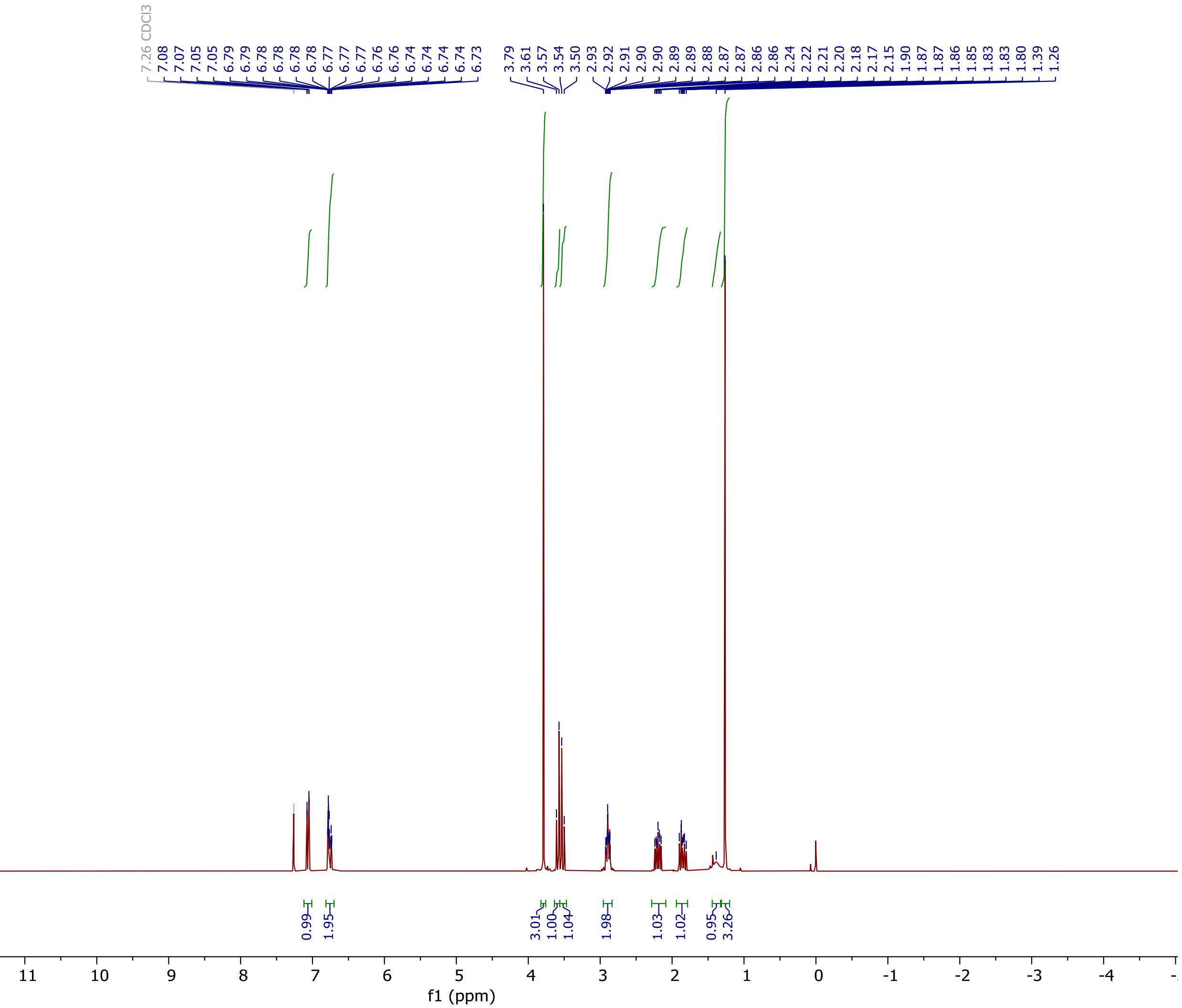

Compound 2h ^{13}C NMR

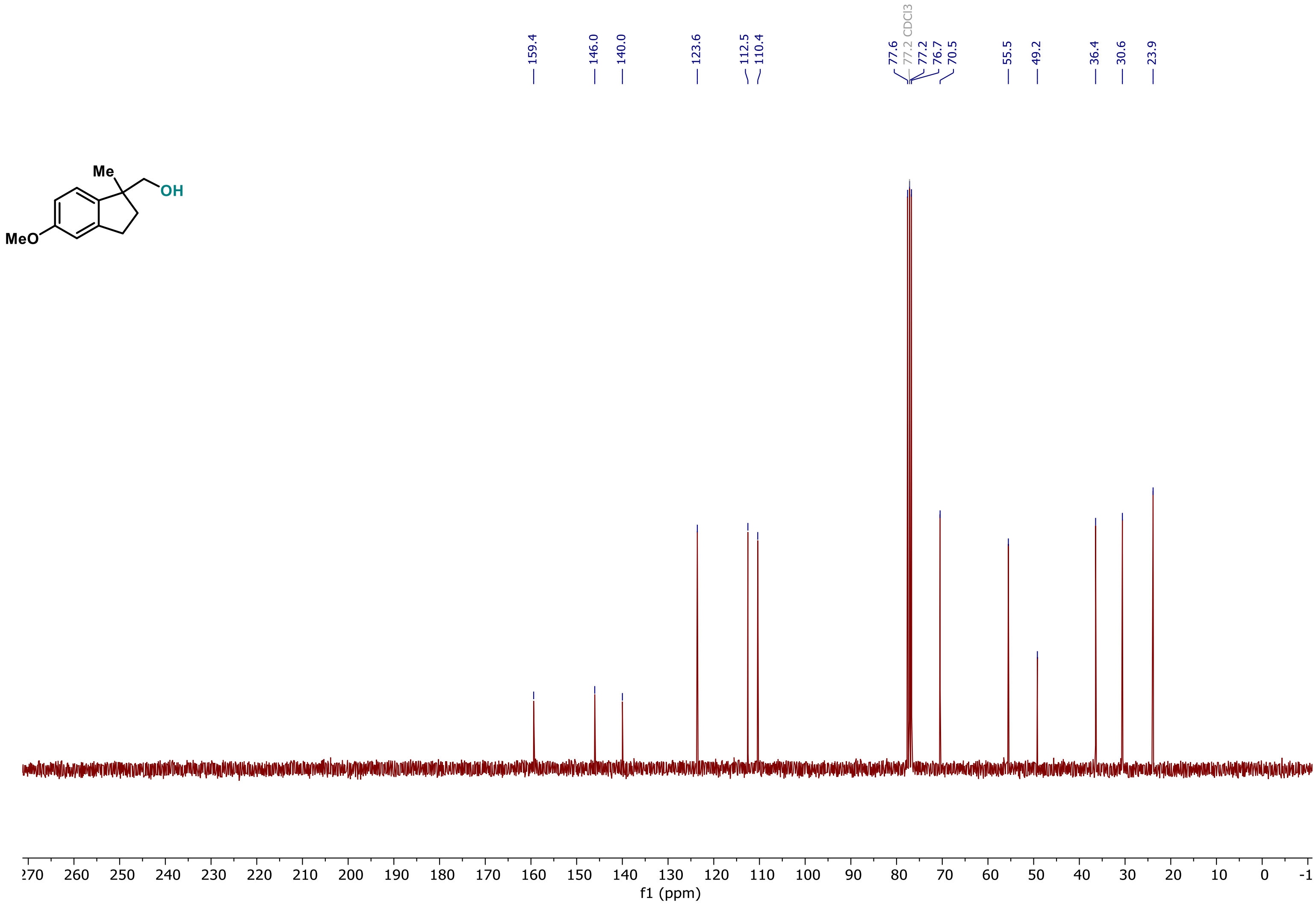

Compound 2i ¹H NMR

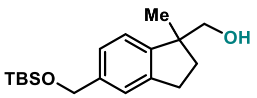

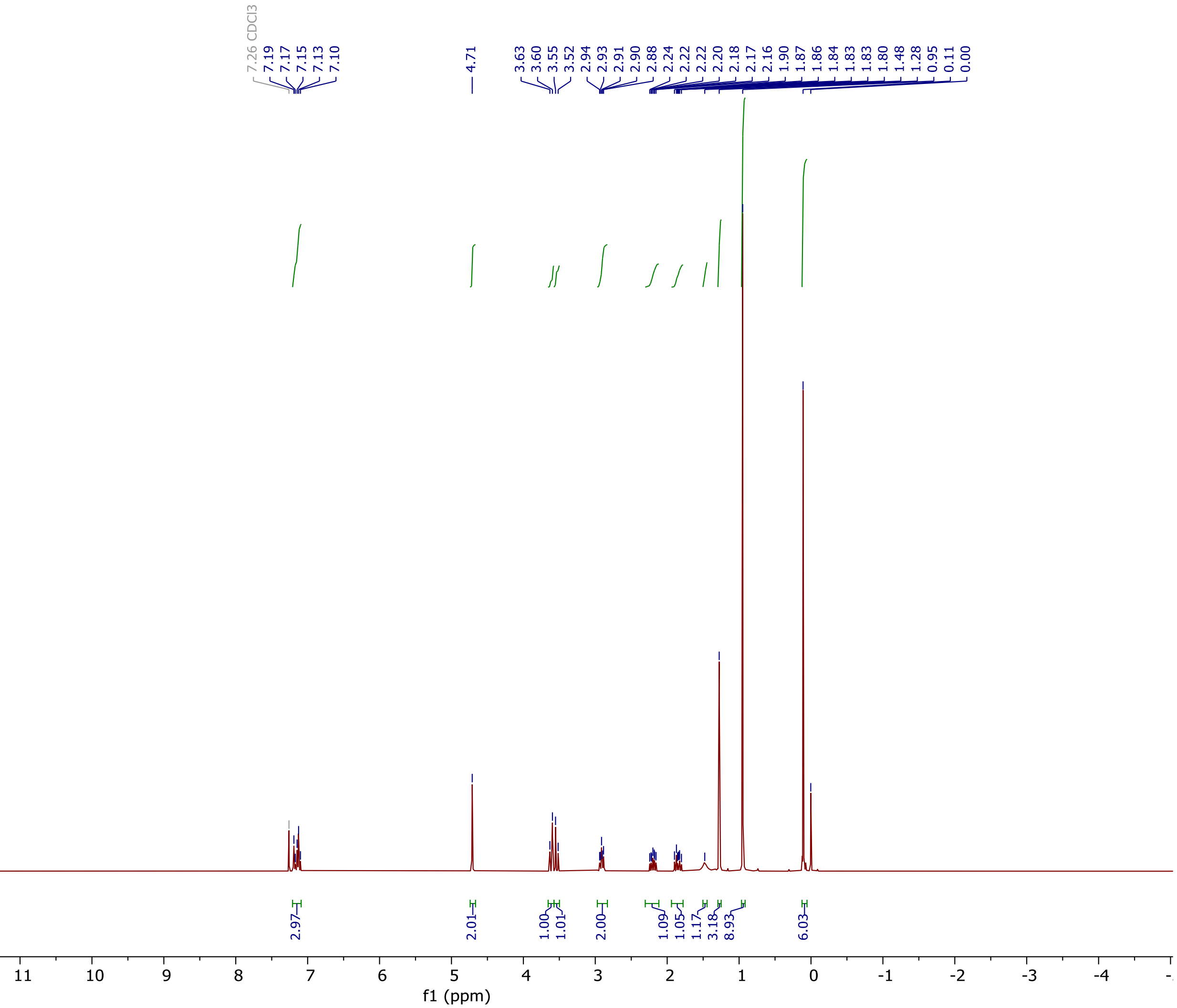

Compound 2i ^{13}C NMR

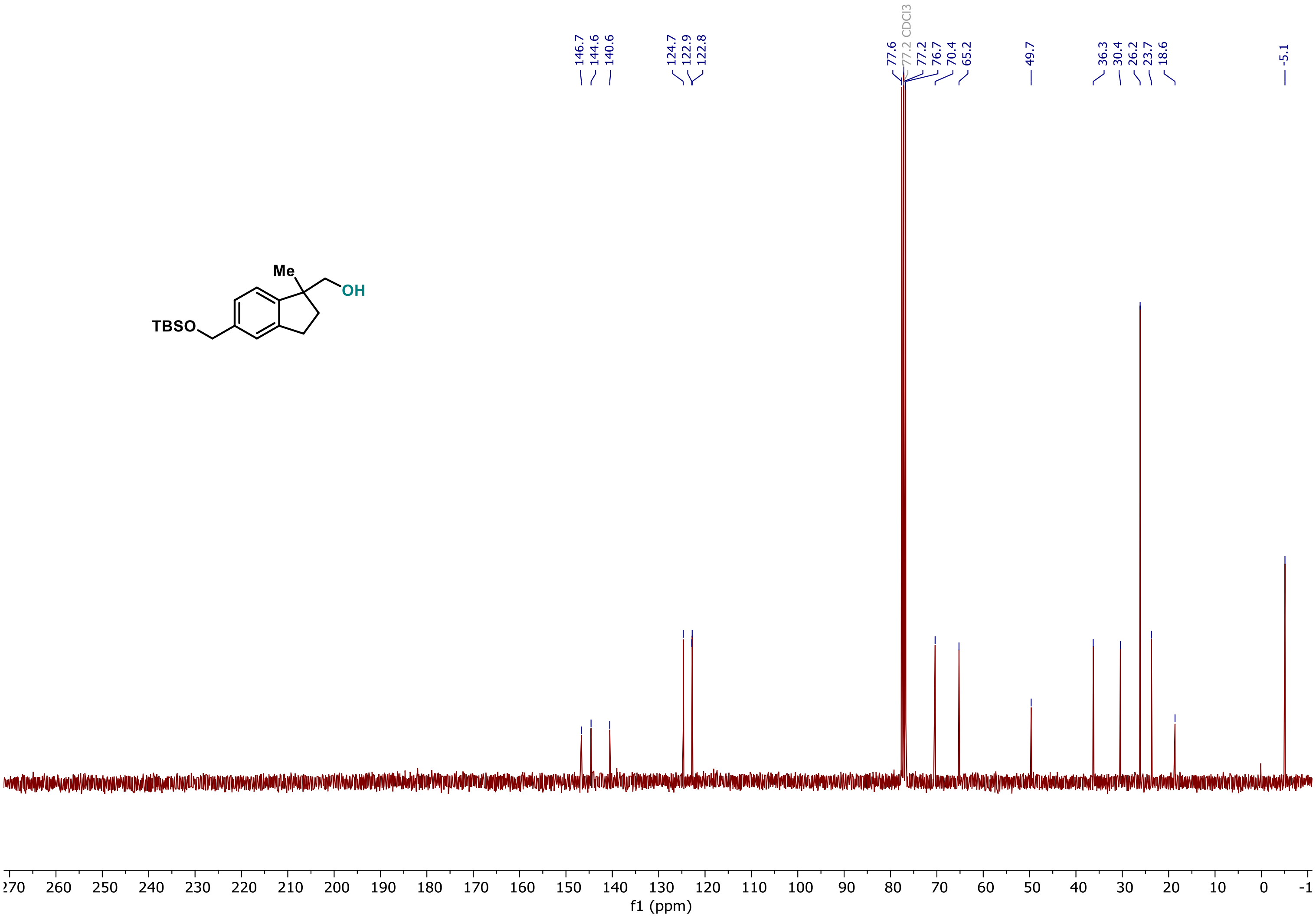

Compound 2j ^{1}H NMR

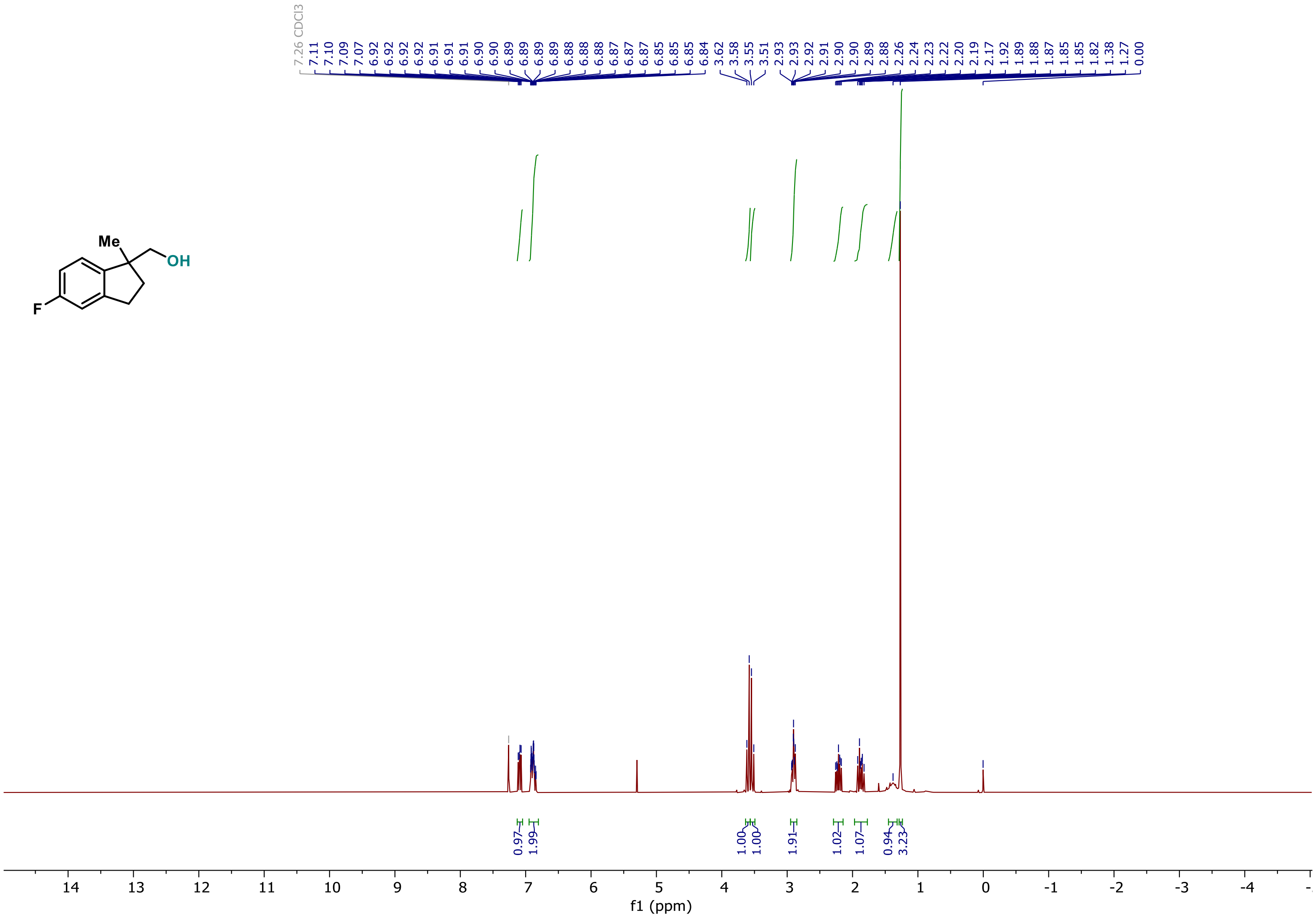

Compound 2j ^{13}C NMR

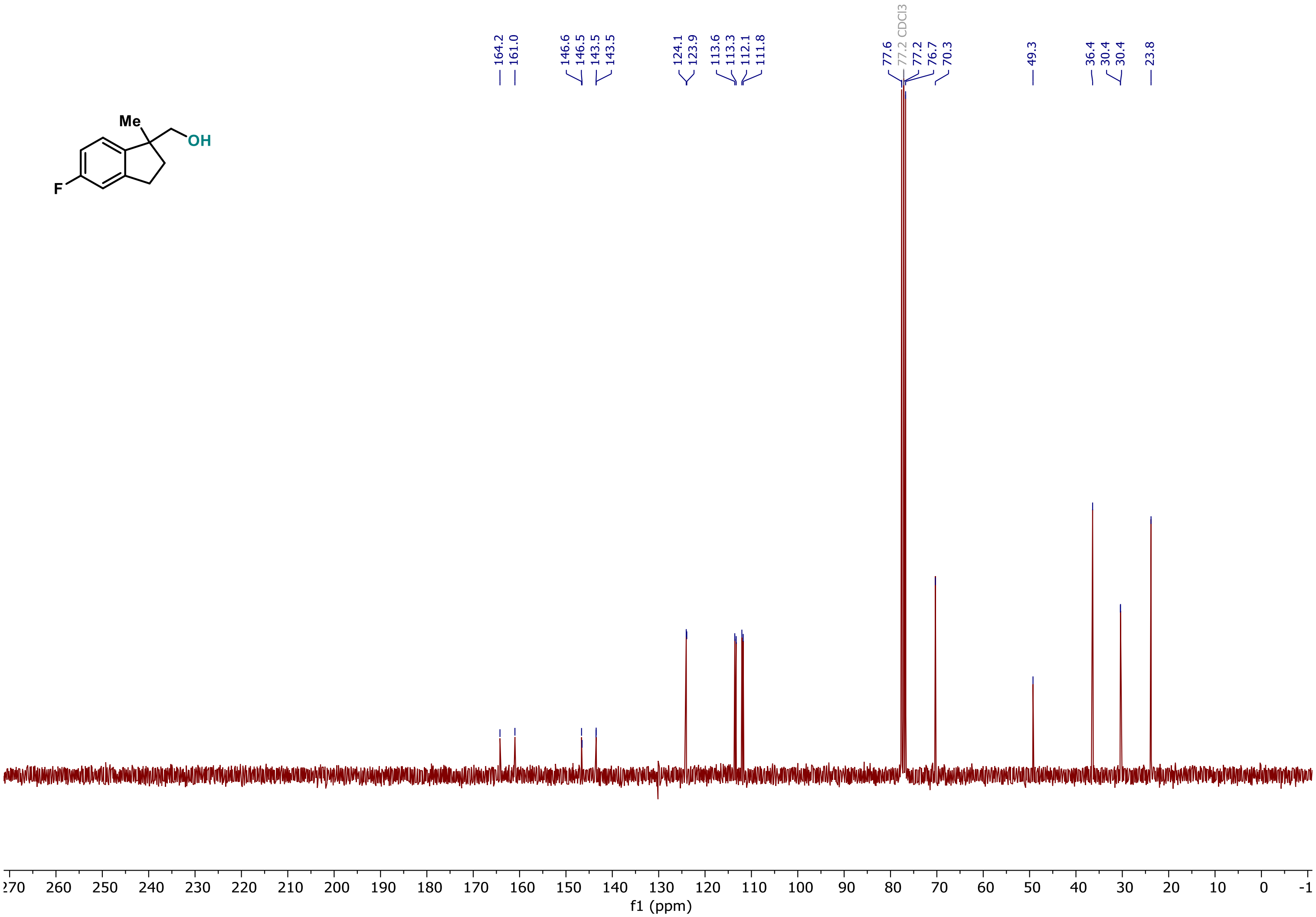

Compound 2j ^{19}F NMR

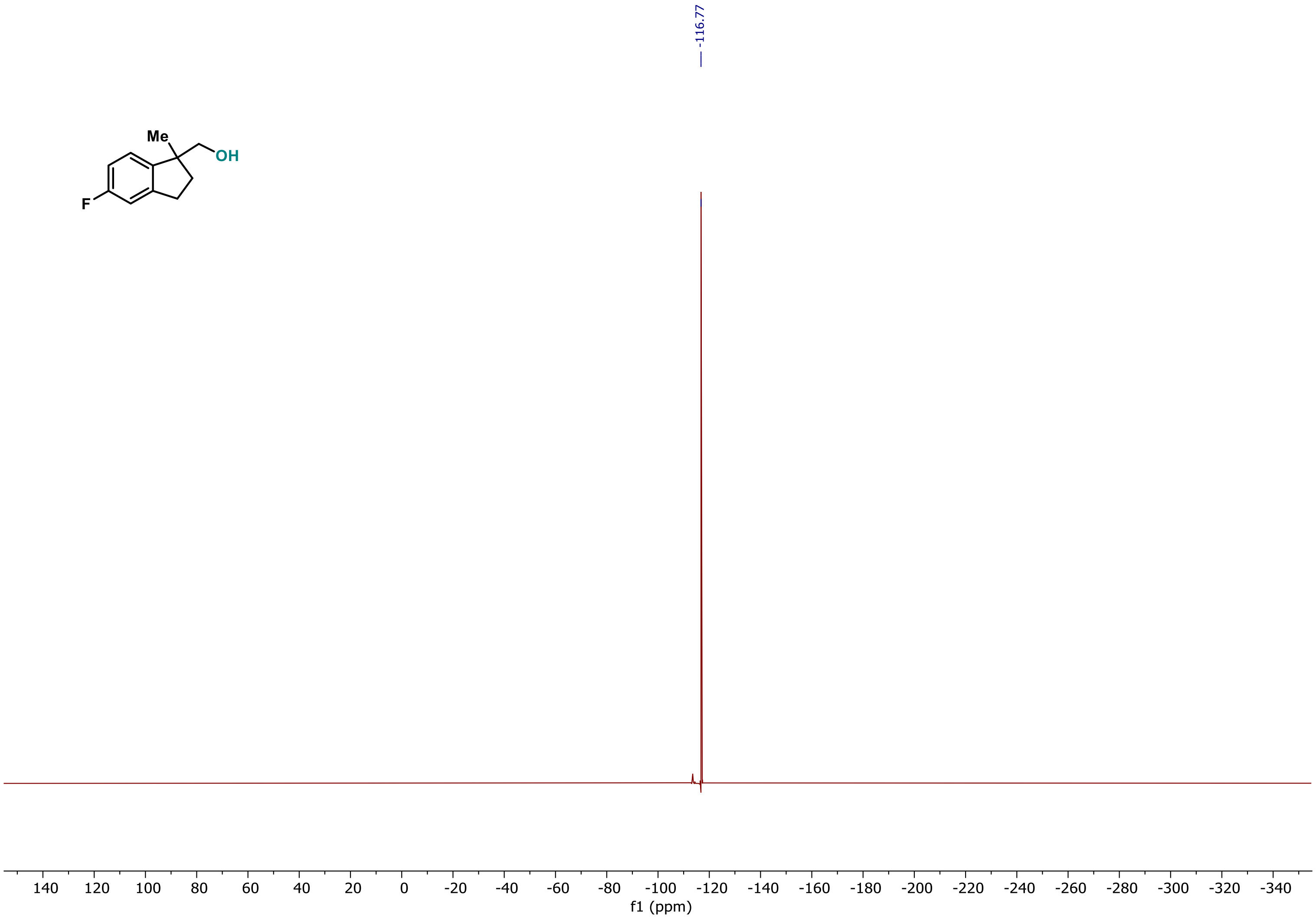

Compound 2k ¹H NMR

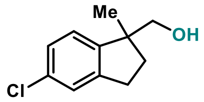

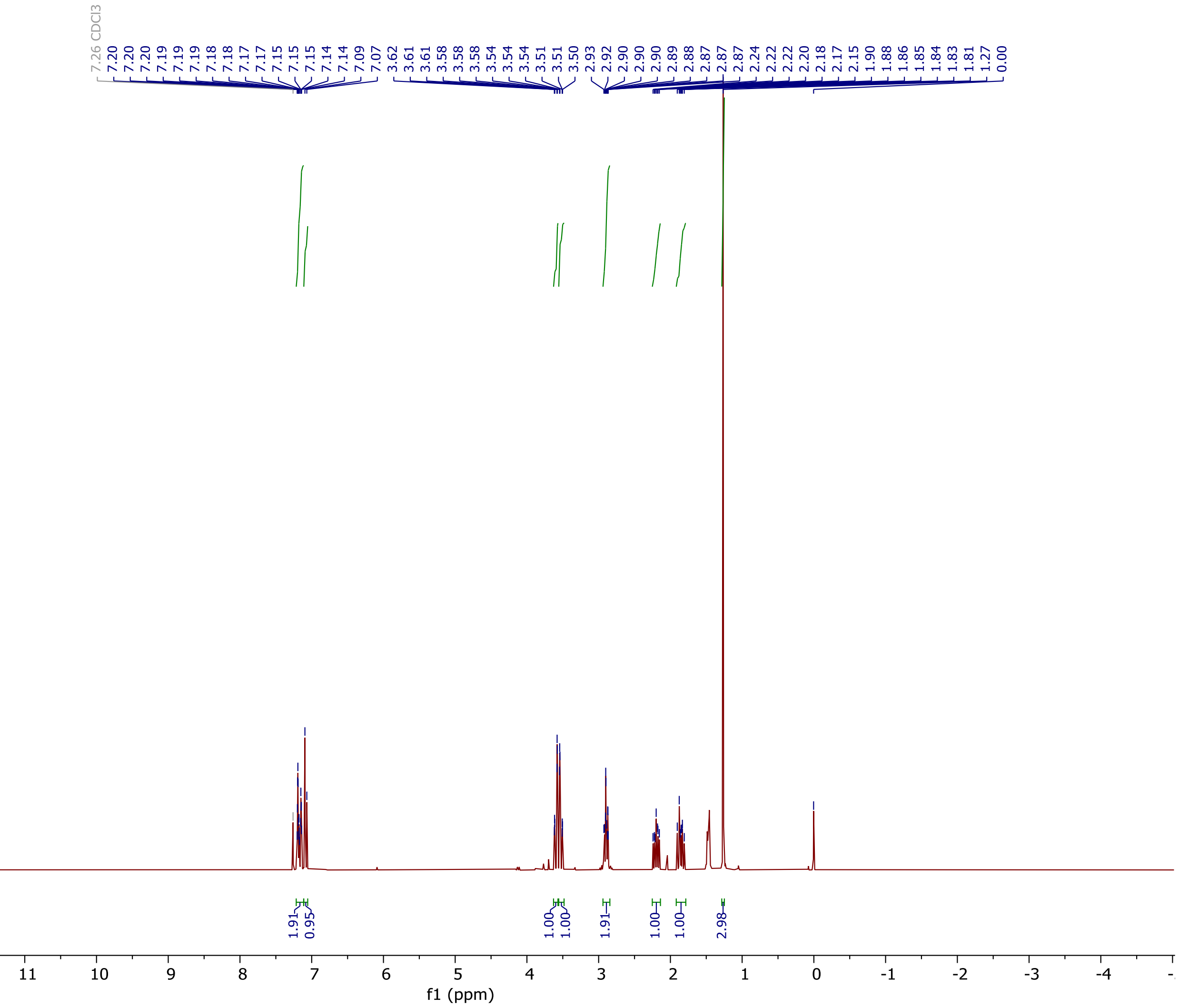

Compound 2k ^{13}C NMR

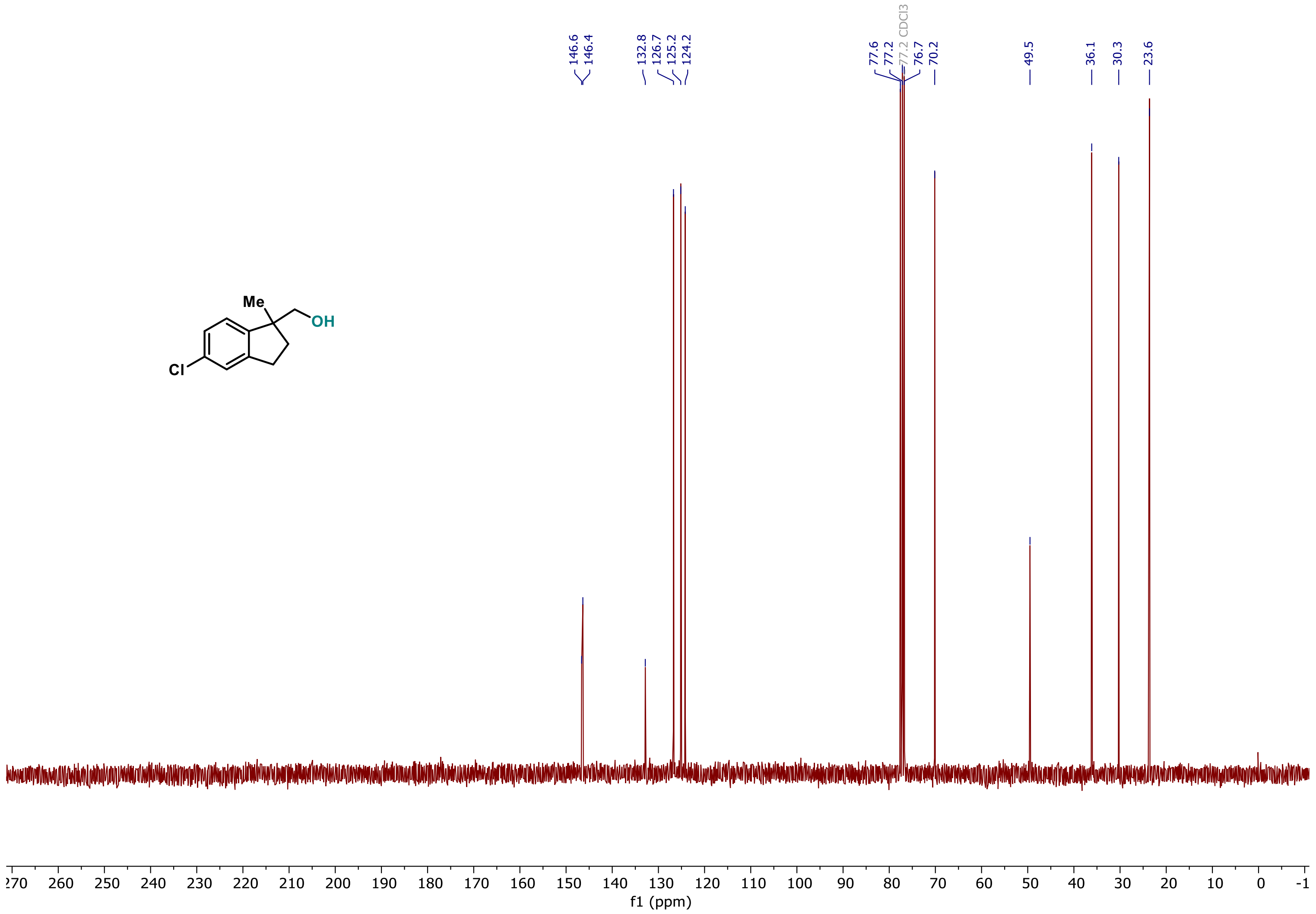

Compound 2l ¹H NMR

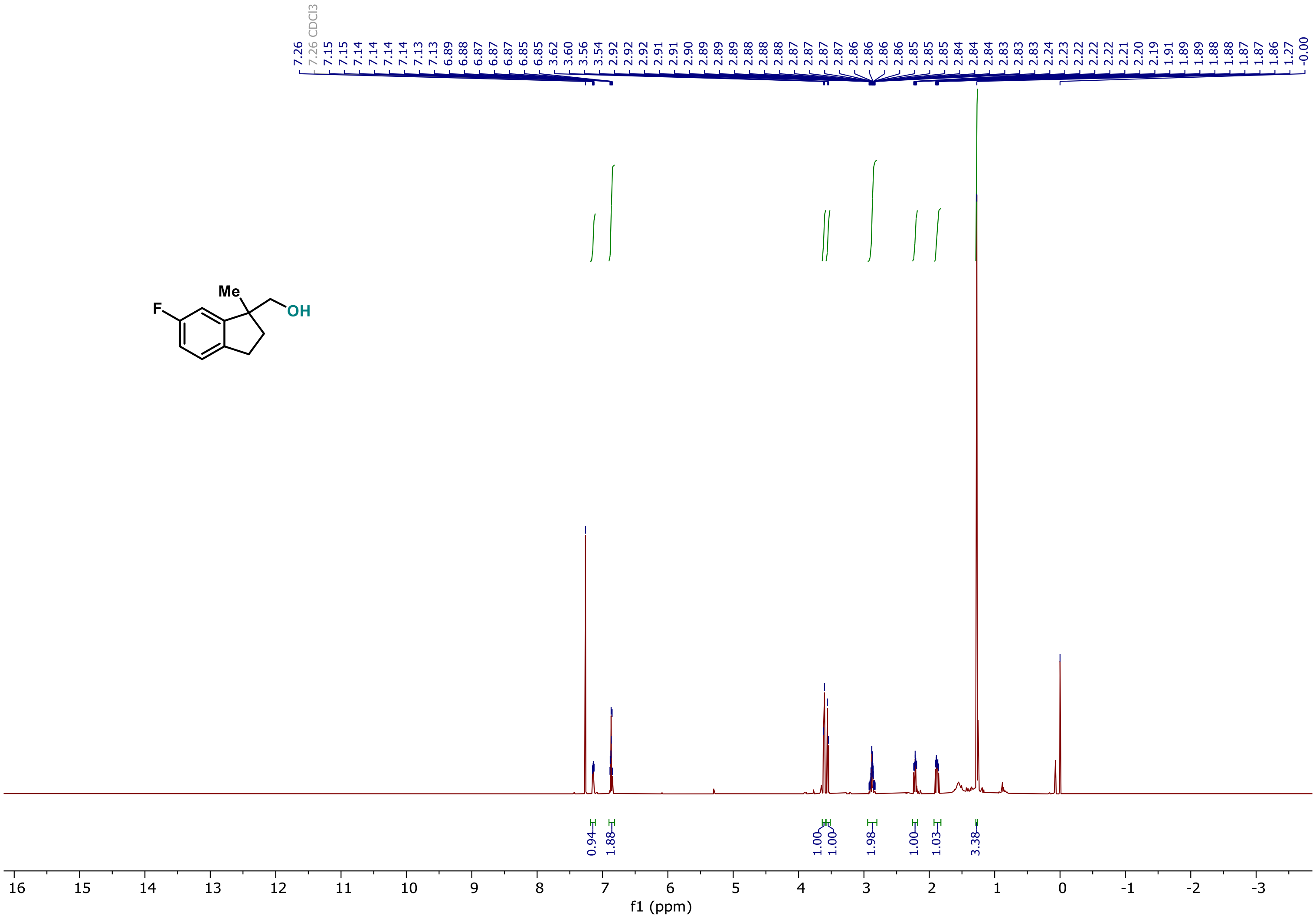

Compound 2l ^{13}C NMR

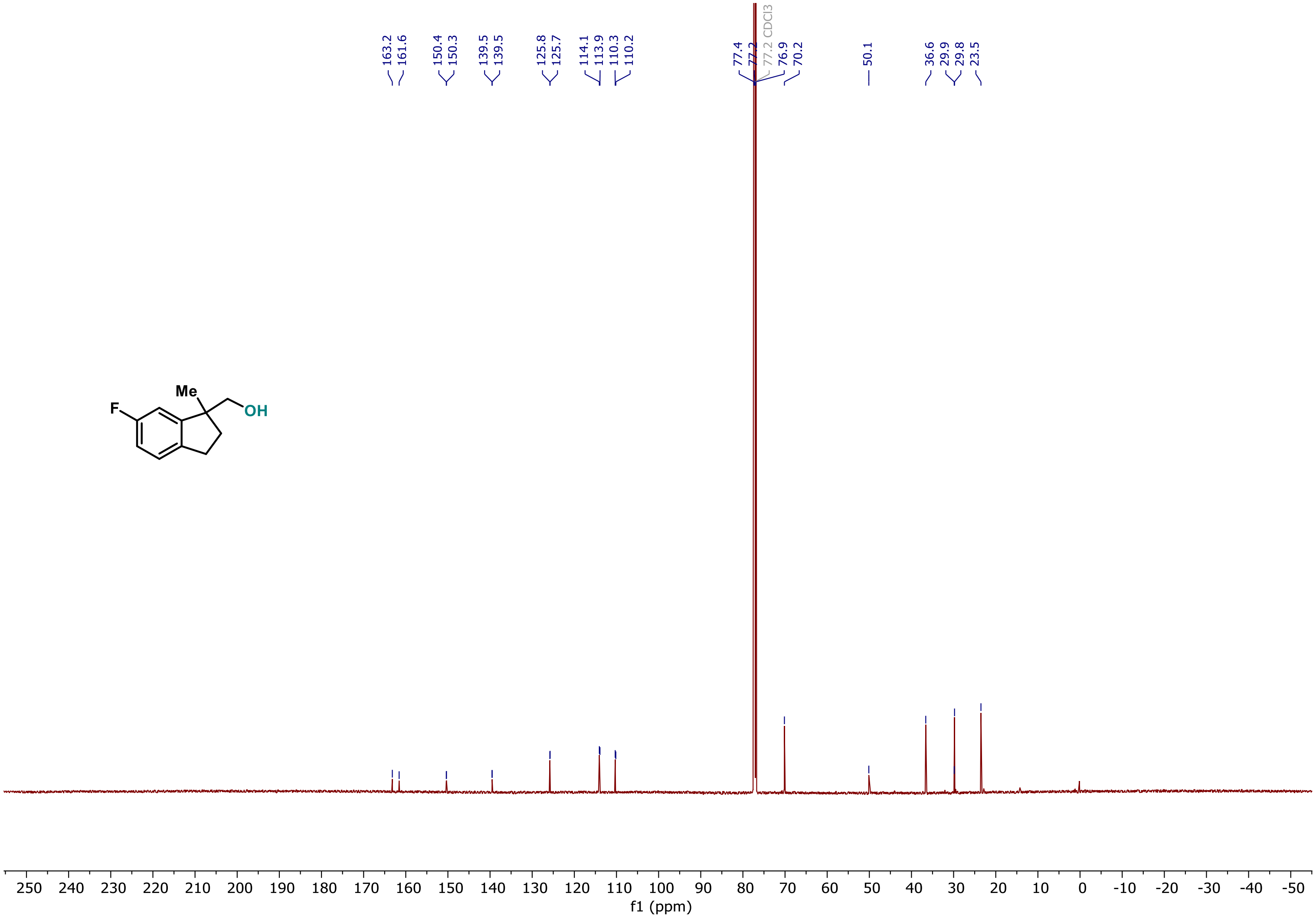

Compound 2l ^{19}F NMR

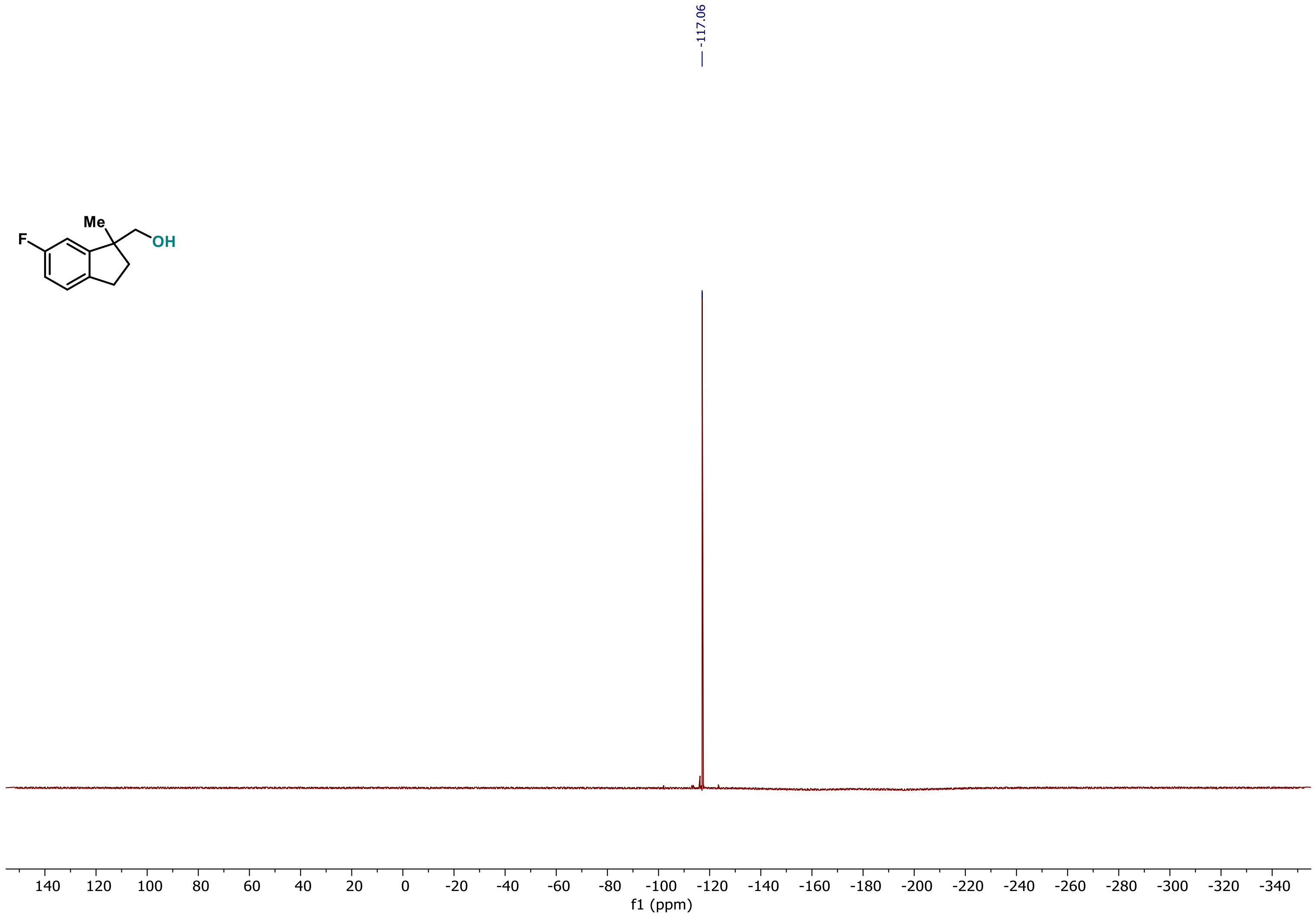

Compound 2m ^{1}H NMR

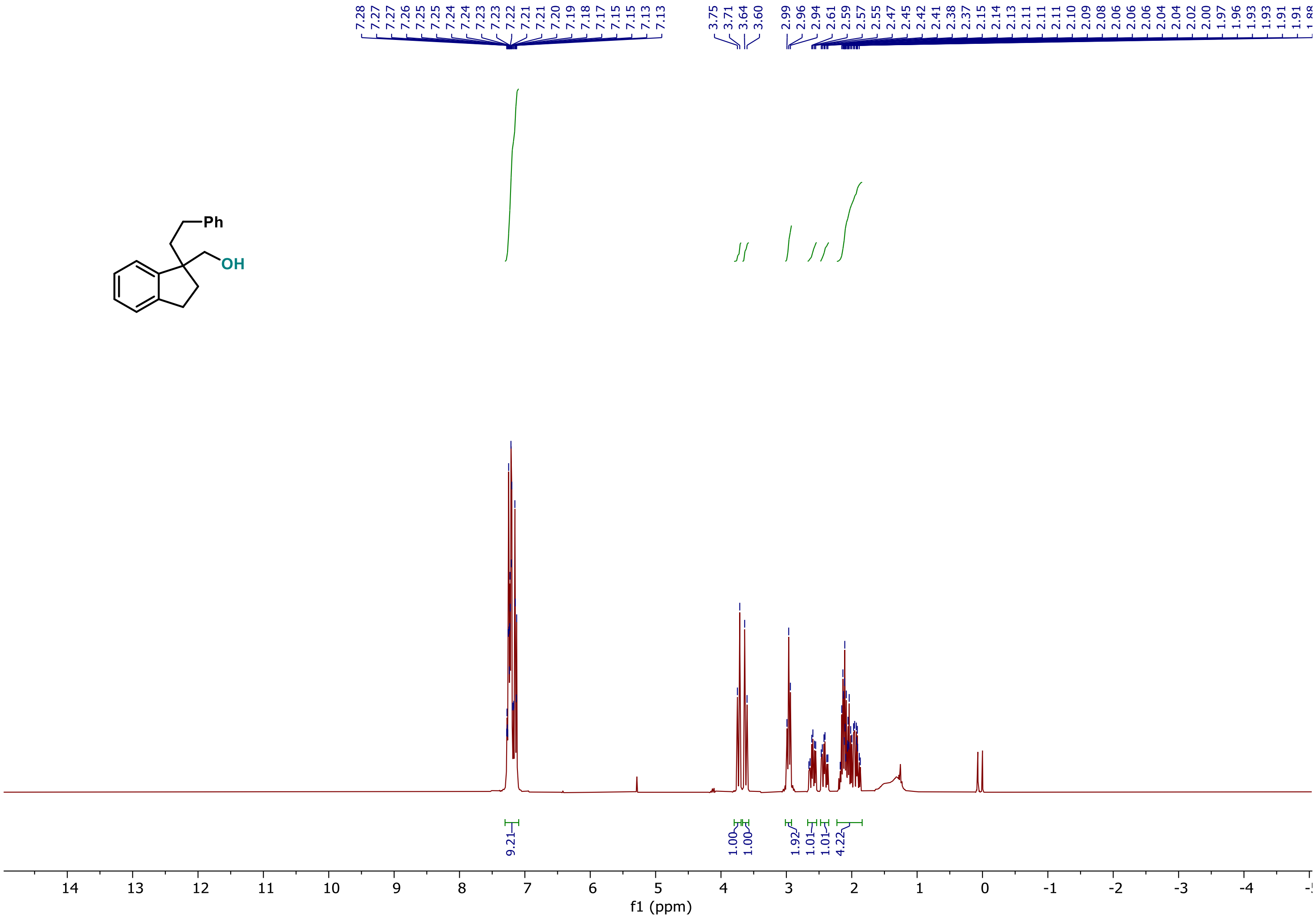

Compound 2m ^{13}C NMR

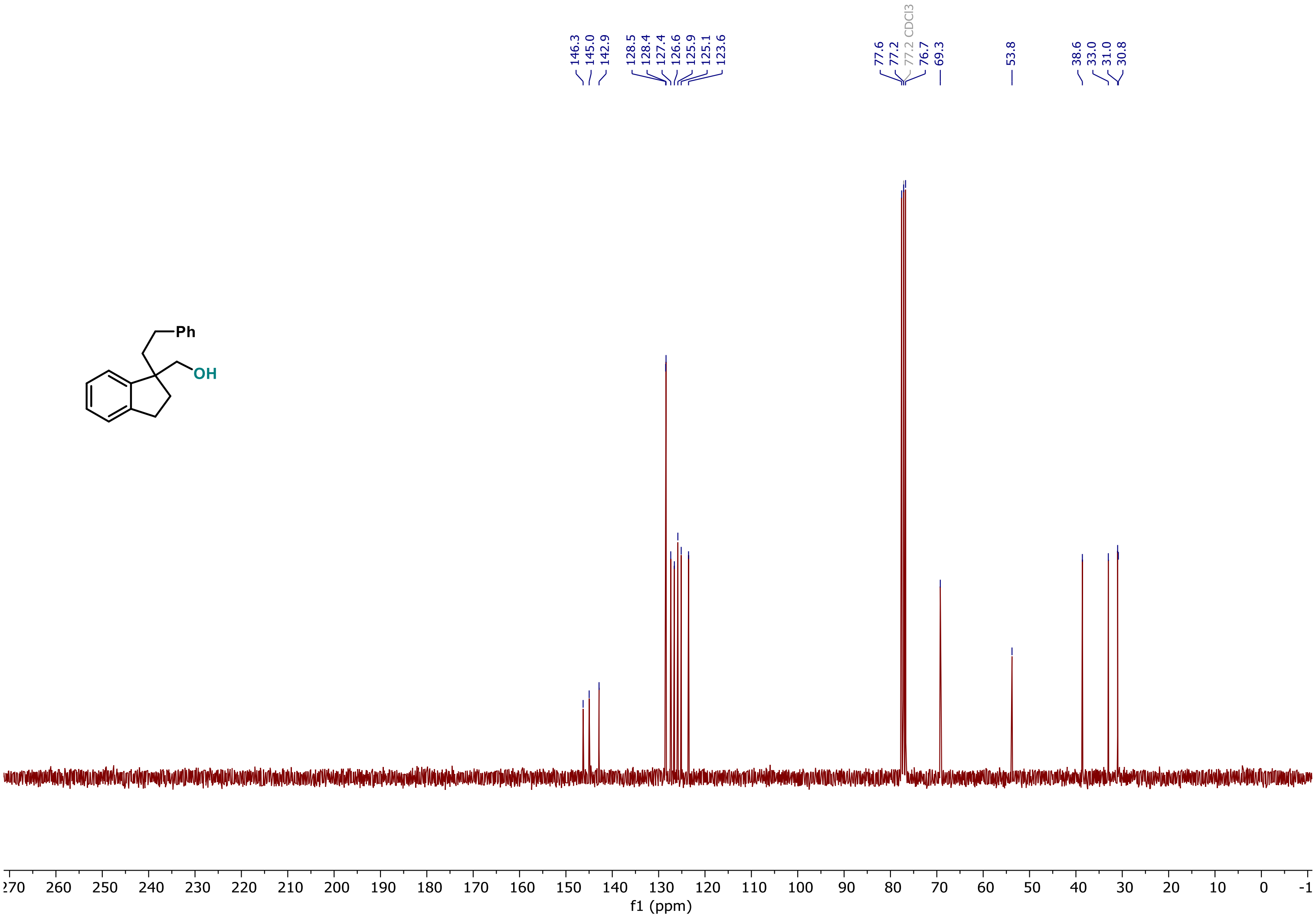

Compound 2n ^{1}H NMR

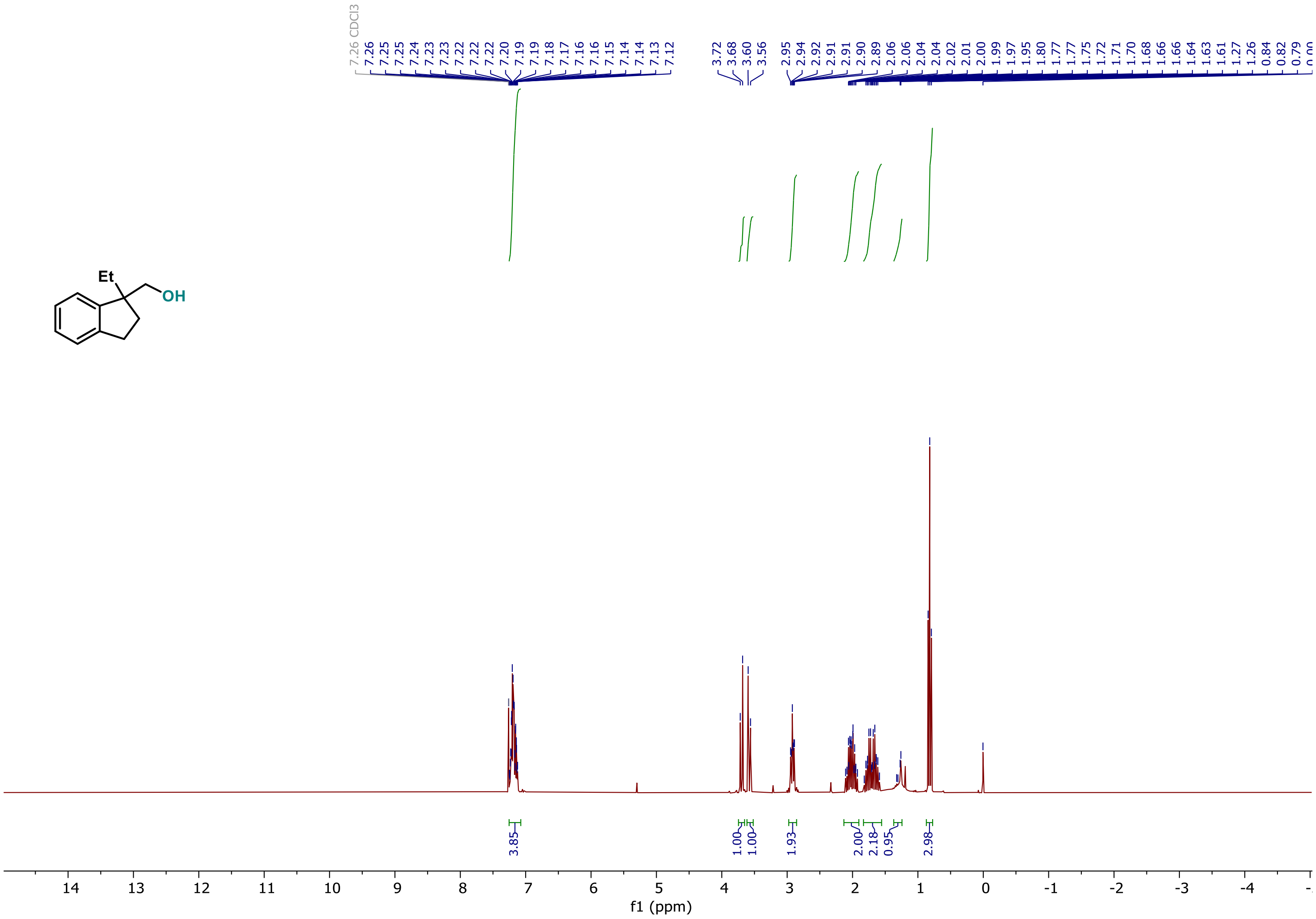

Compound 2n ^{13}C NMR

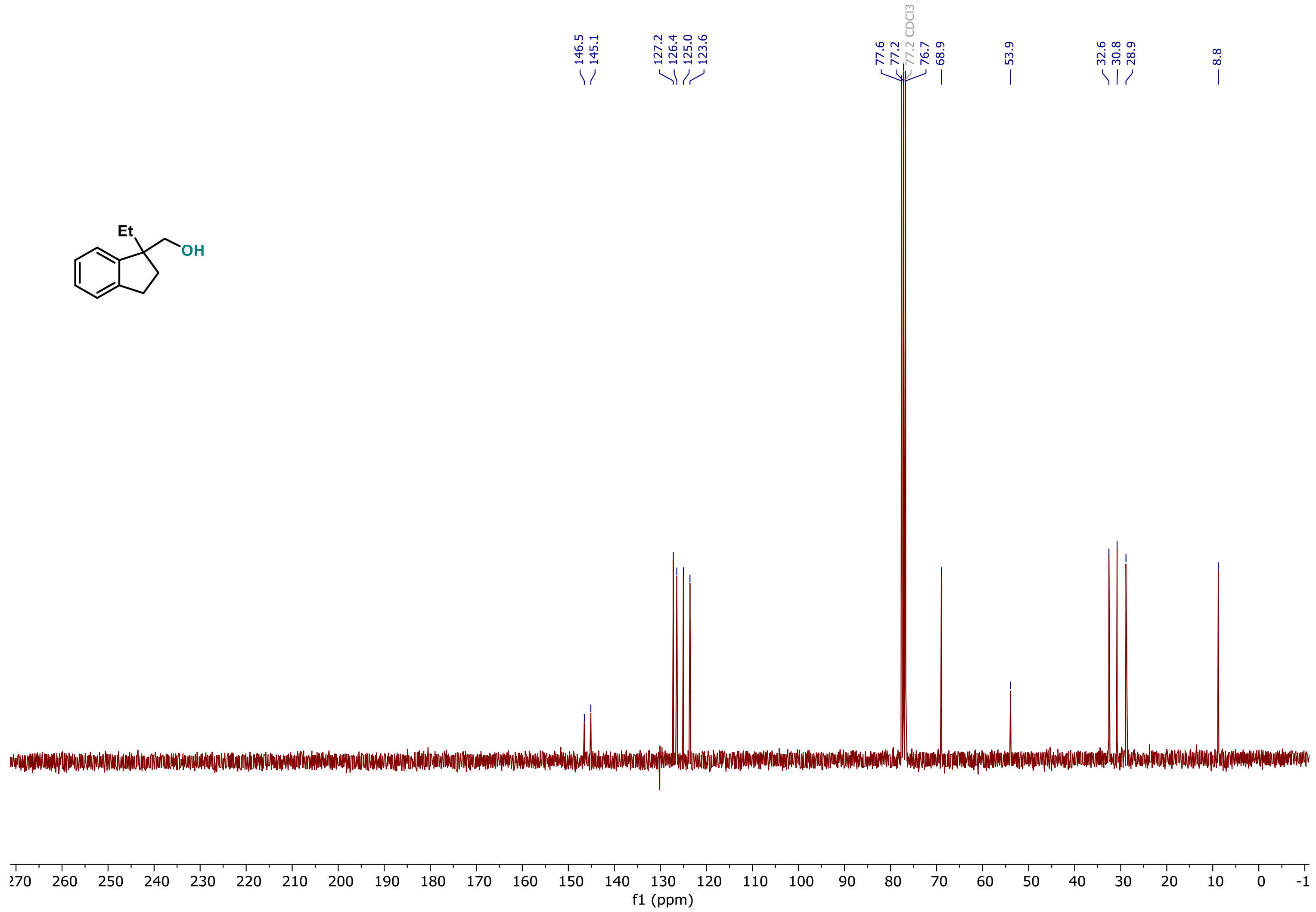

Compound 2o ^{1}H NMR

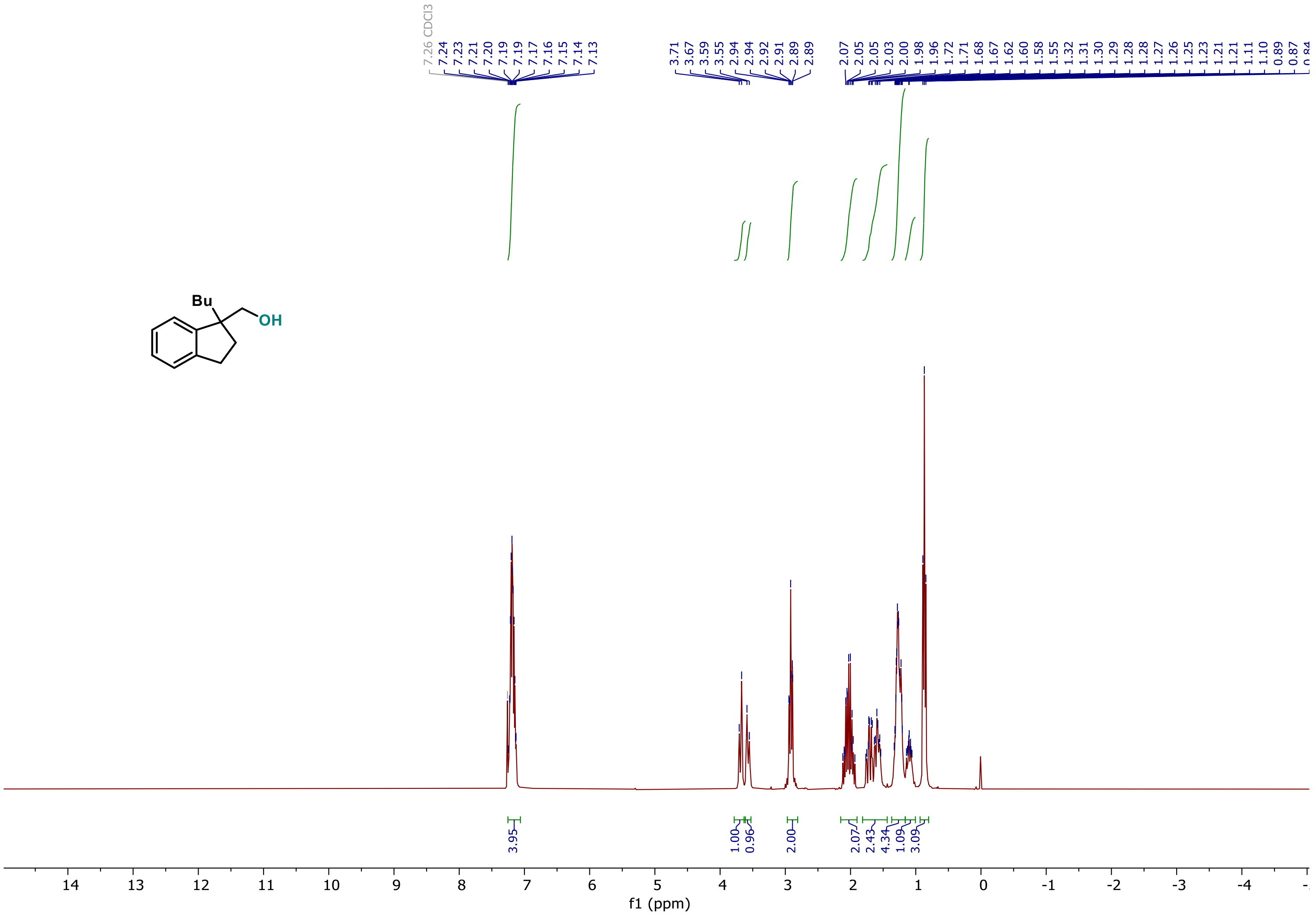

Compound 2o ^{13}C NMR

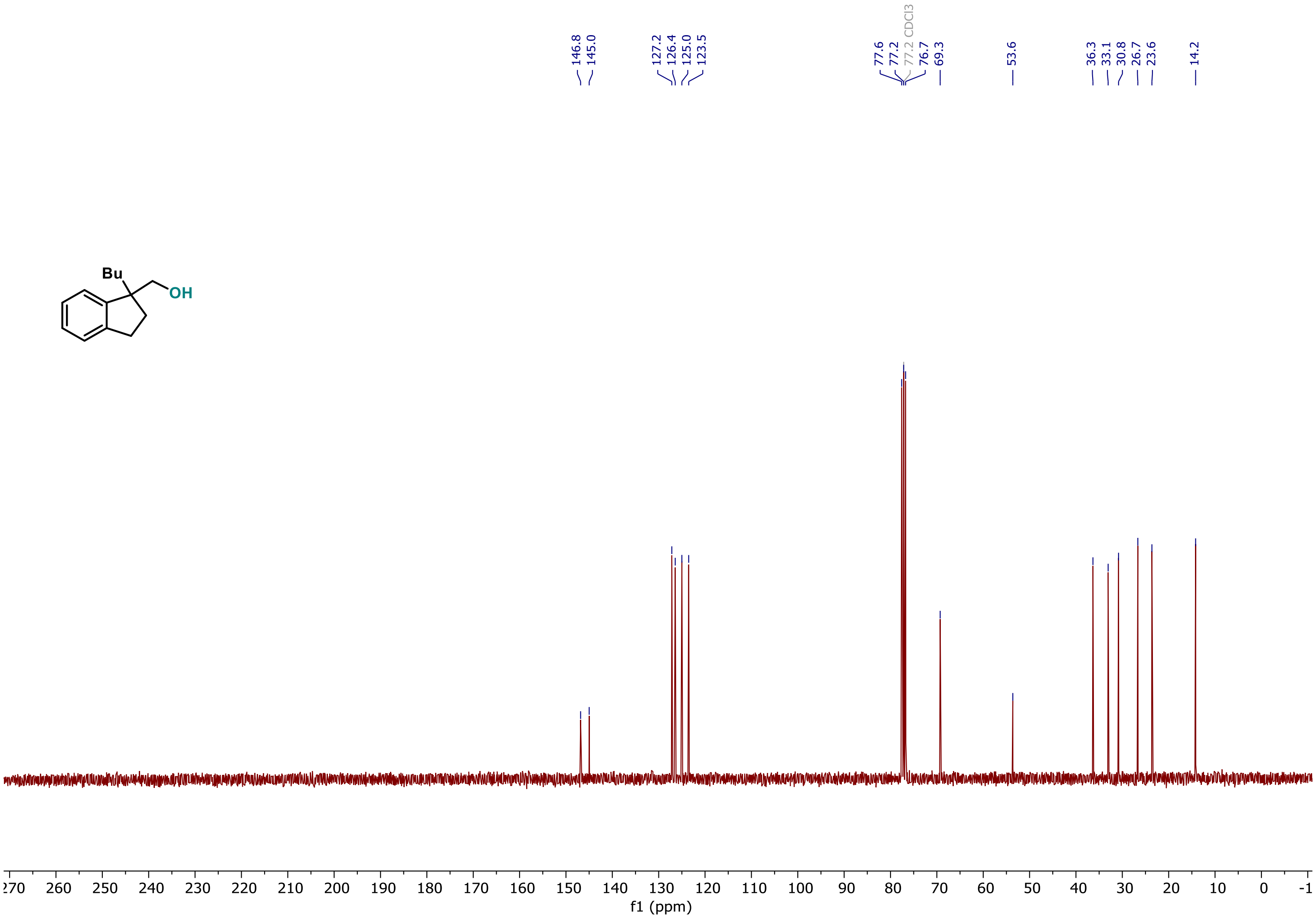

Compound 2p ^{1}H NMR

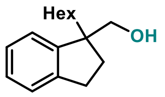

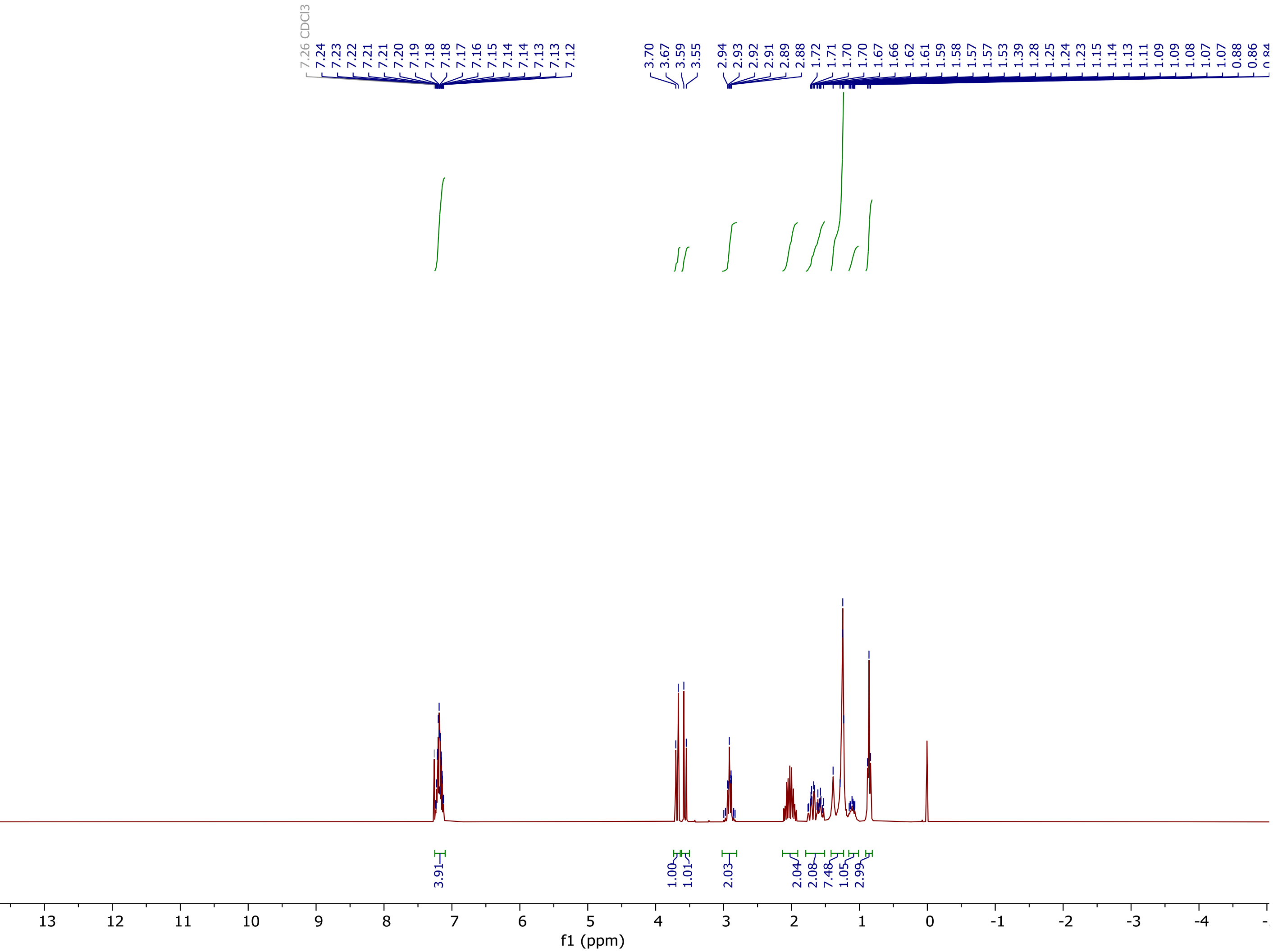

Compound 2p ^{13}C NMR

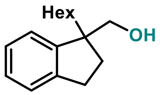

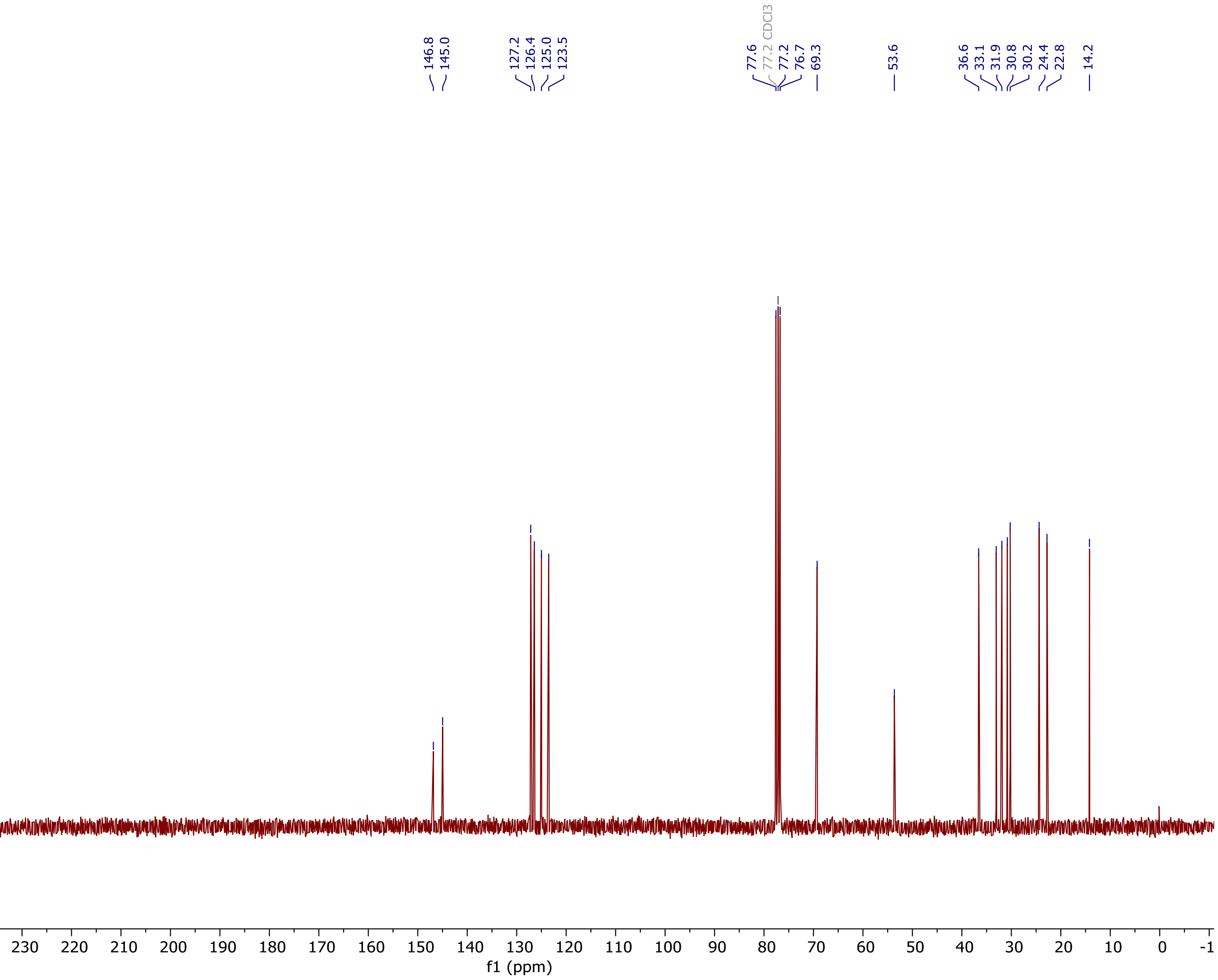

Compound 2q ^{1}H NMR

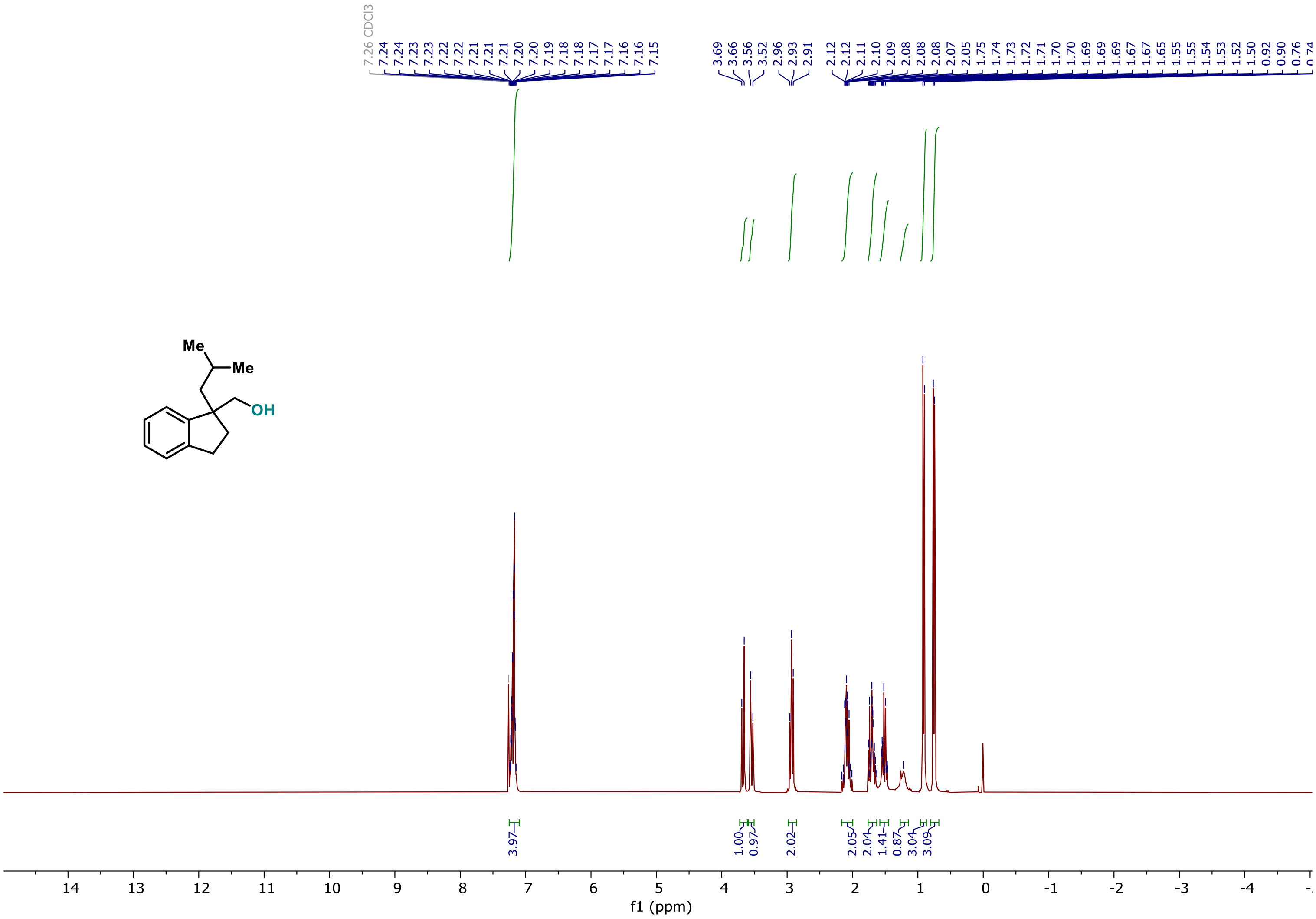

Compound 2q ^{13}C NMR

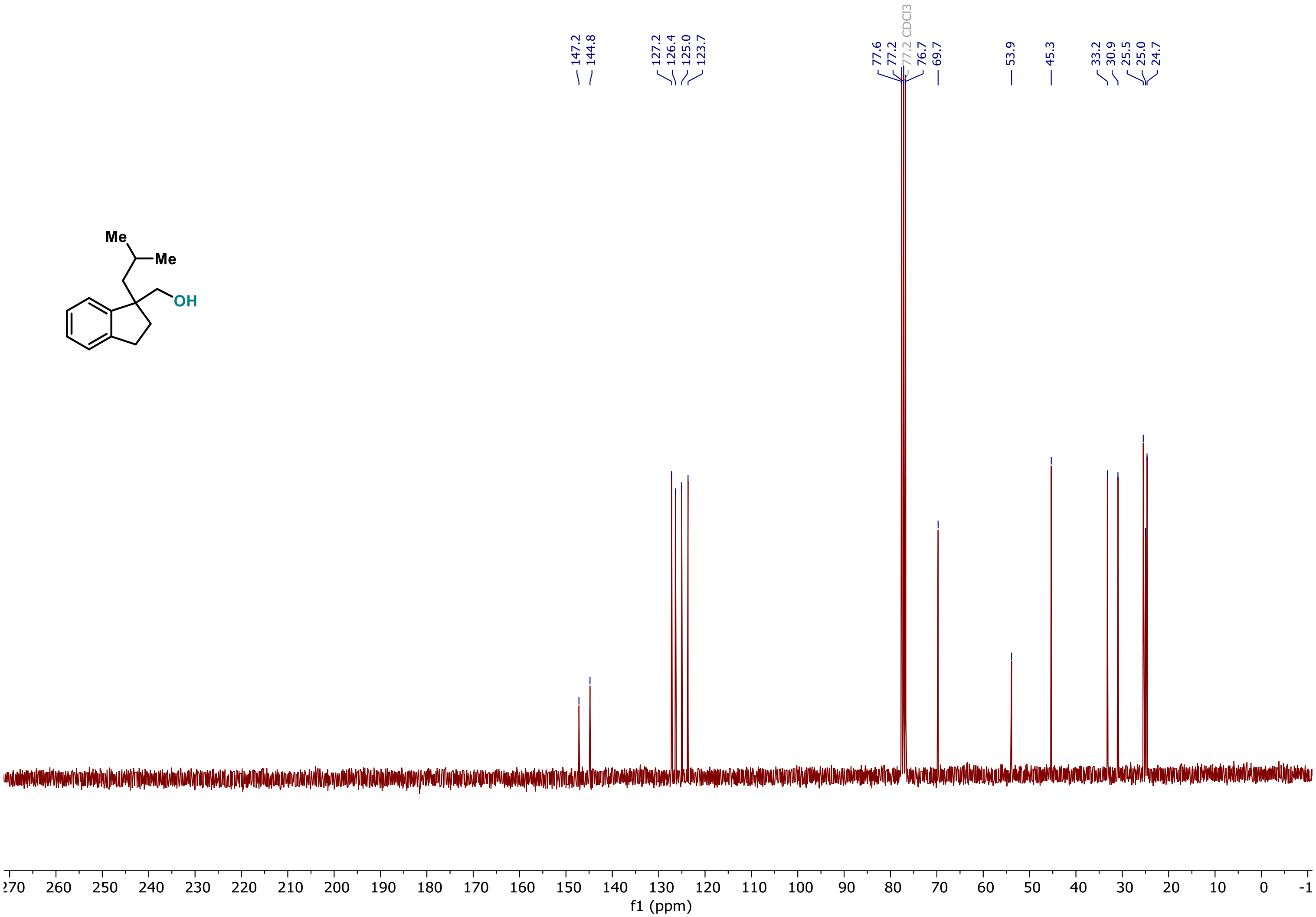

Compound 2r ¹H NMR

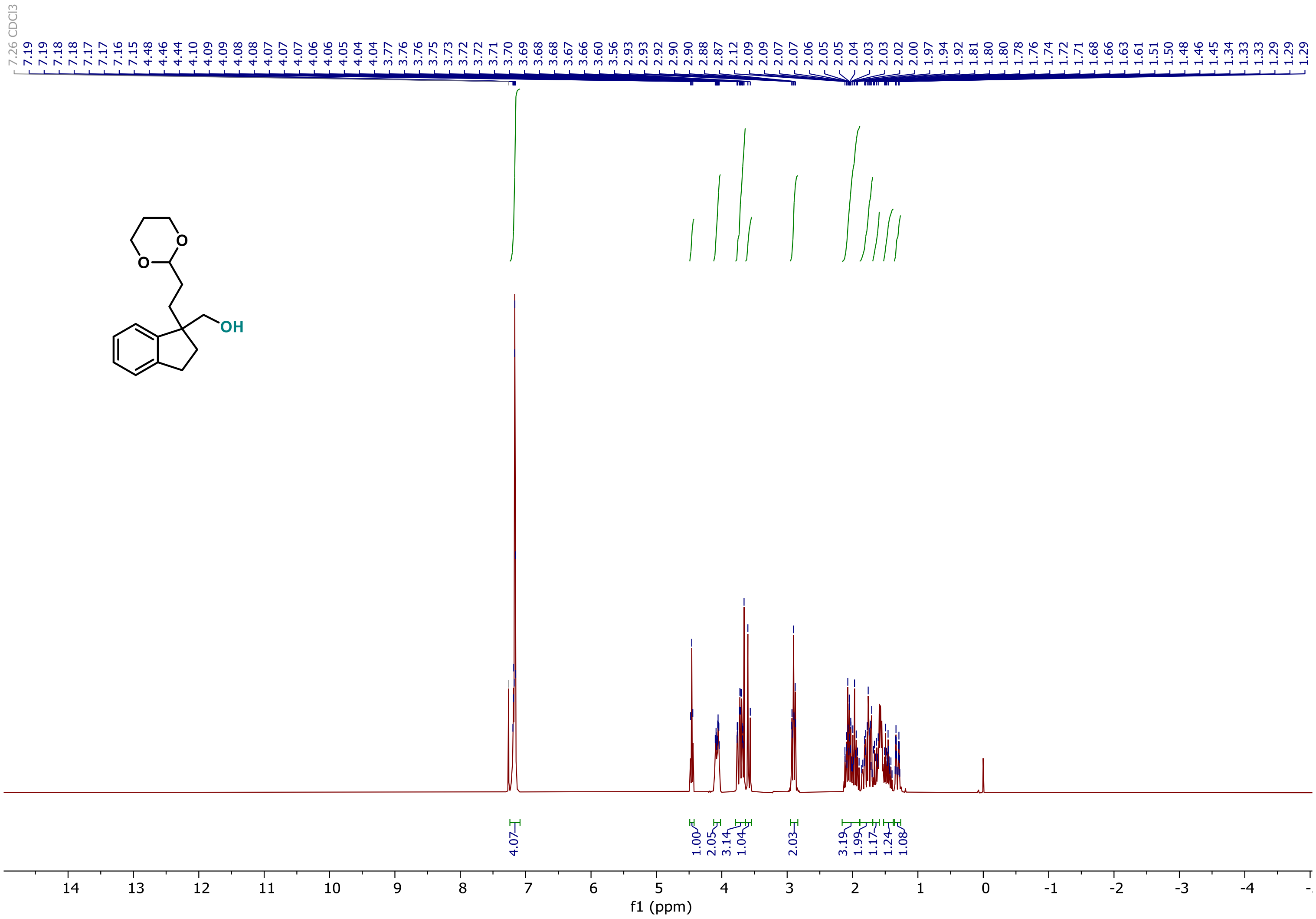

Compound 2r ^{13}C NMR

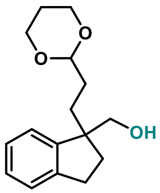

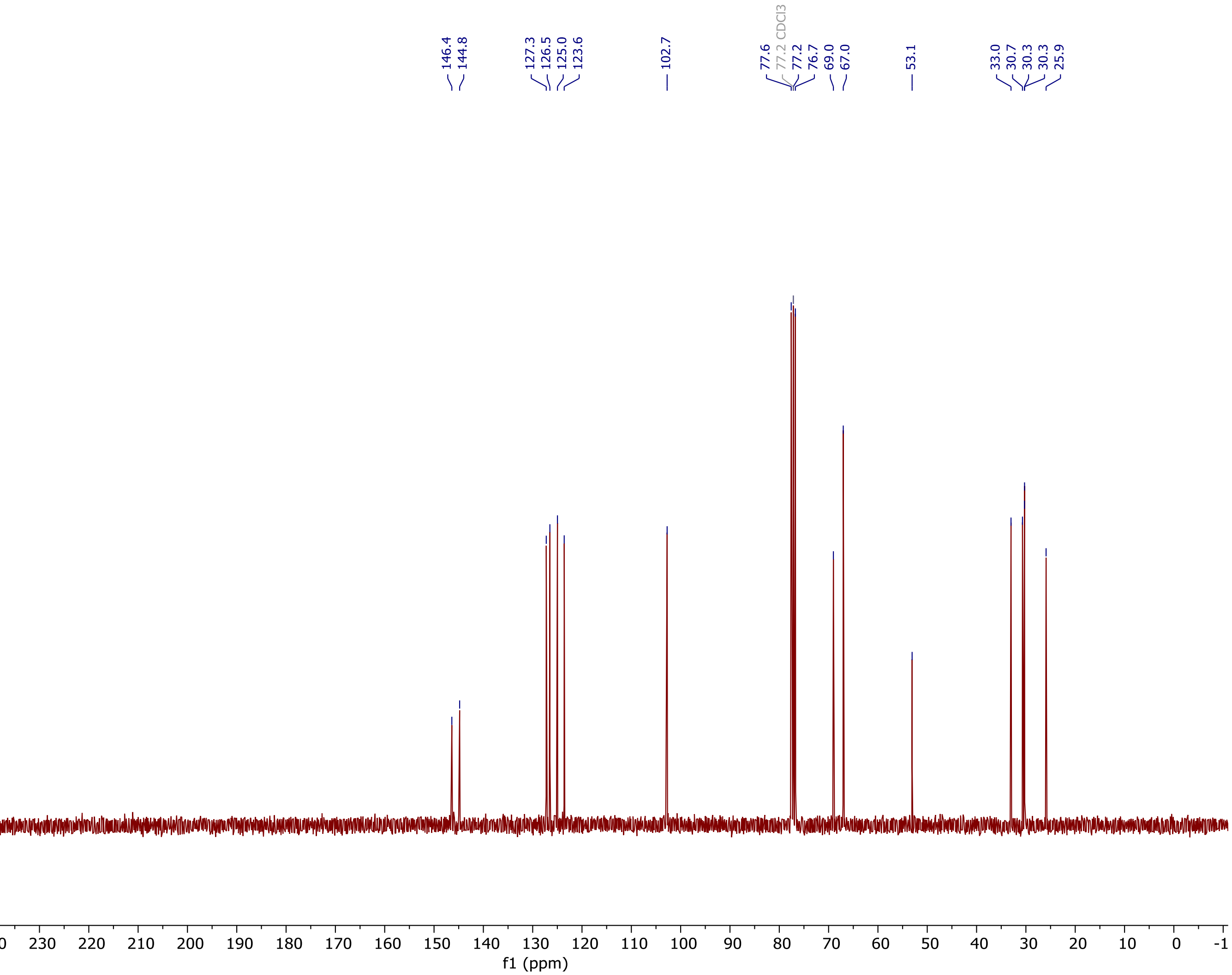

Compound 2s ¹H NMR

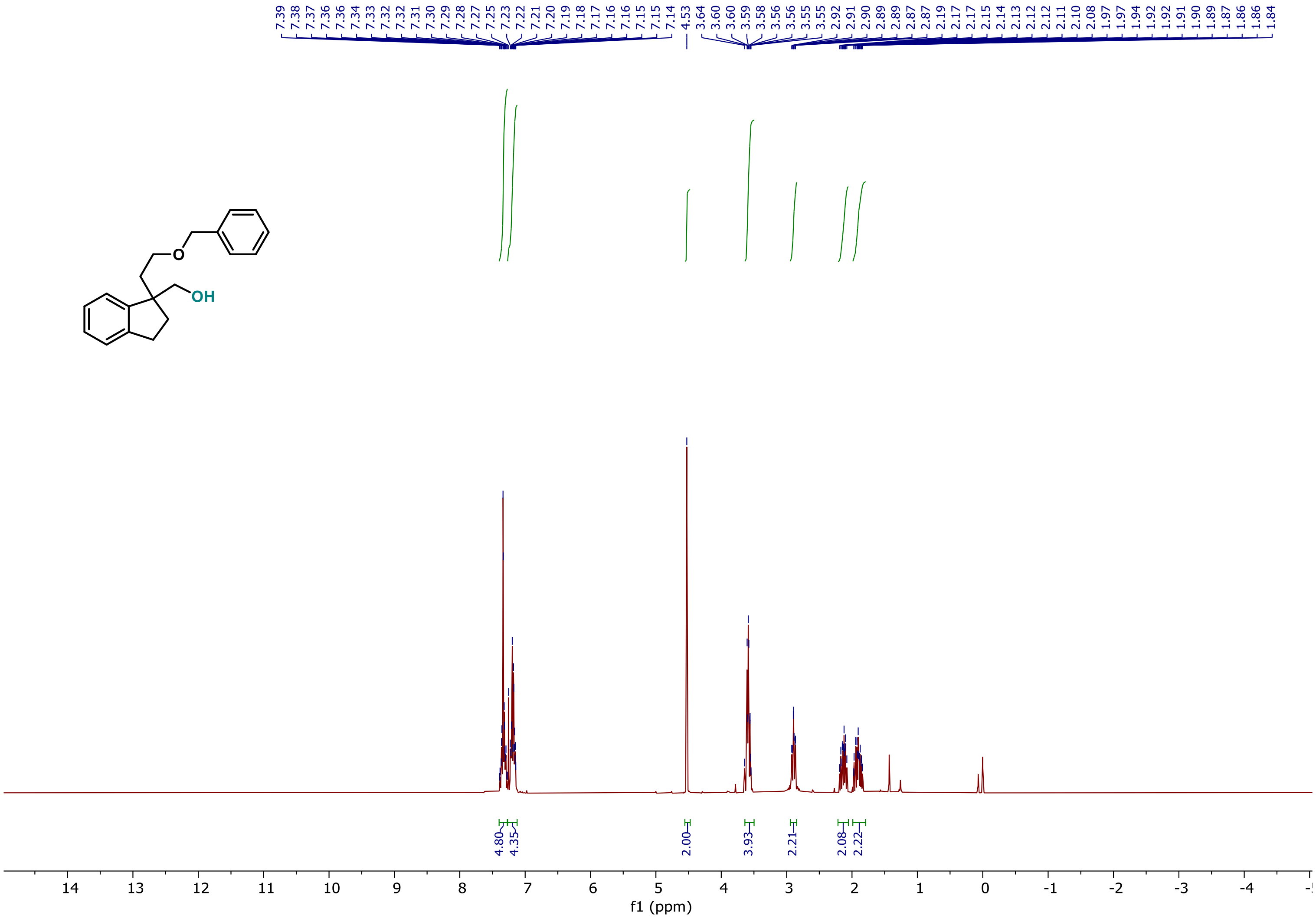

Compound 2s ^{13}C NMR

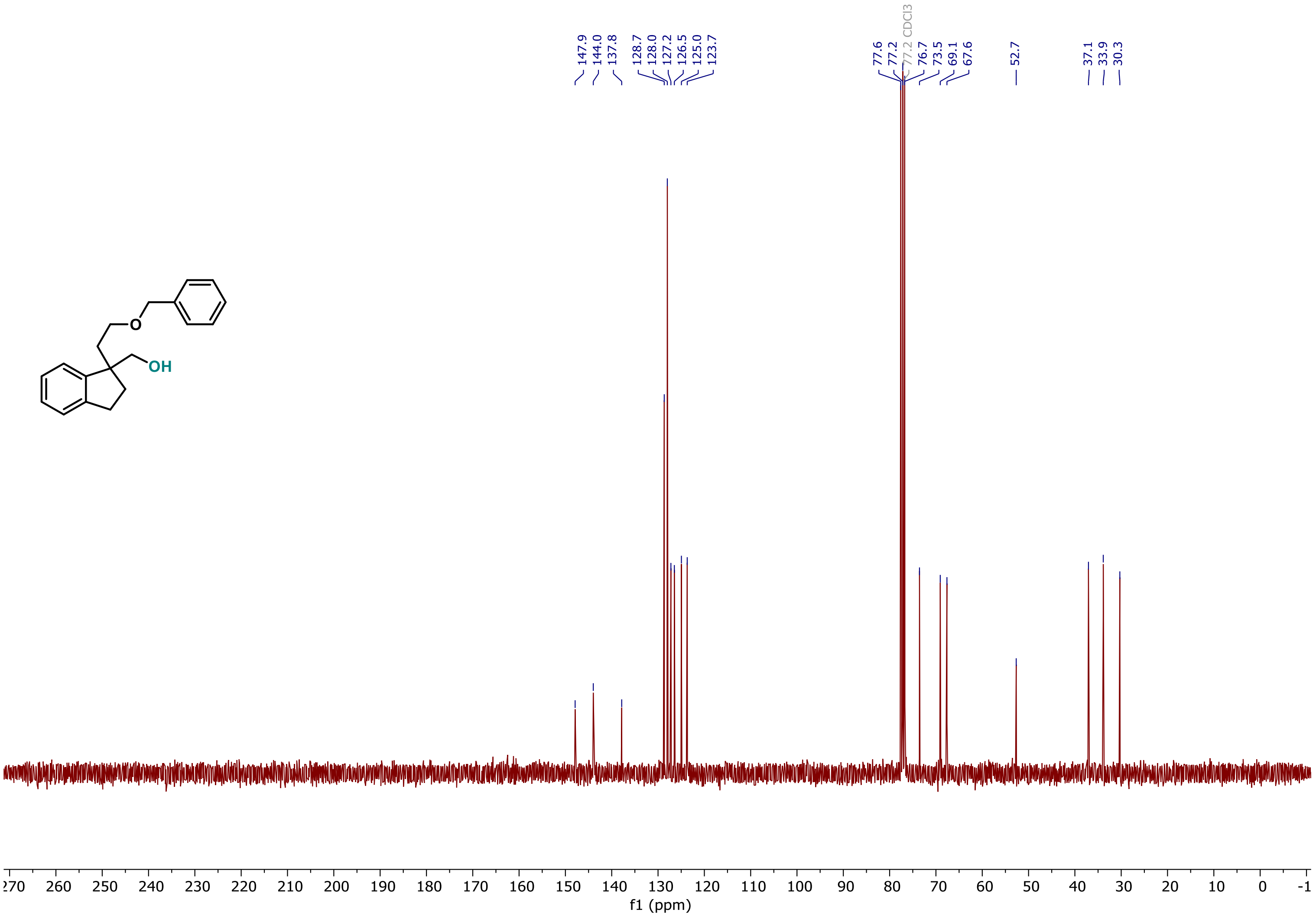

Compound 2t ^{1}H NMR

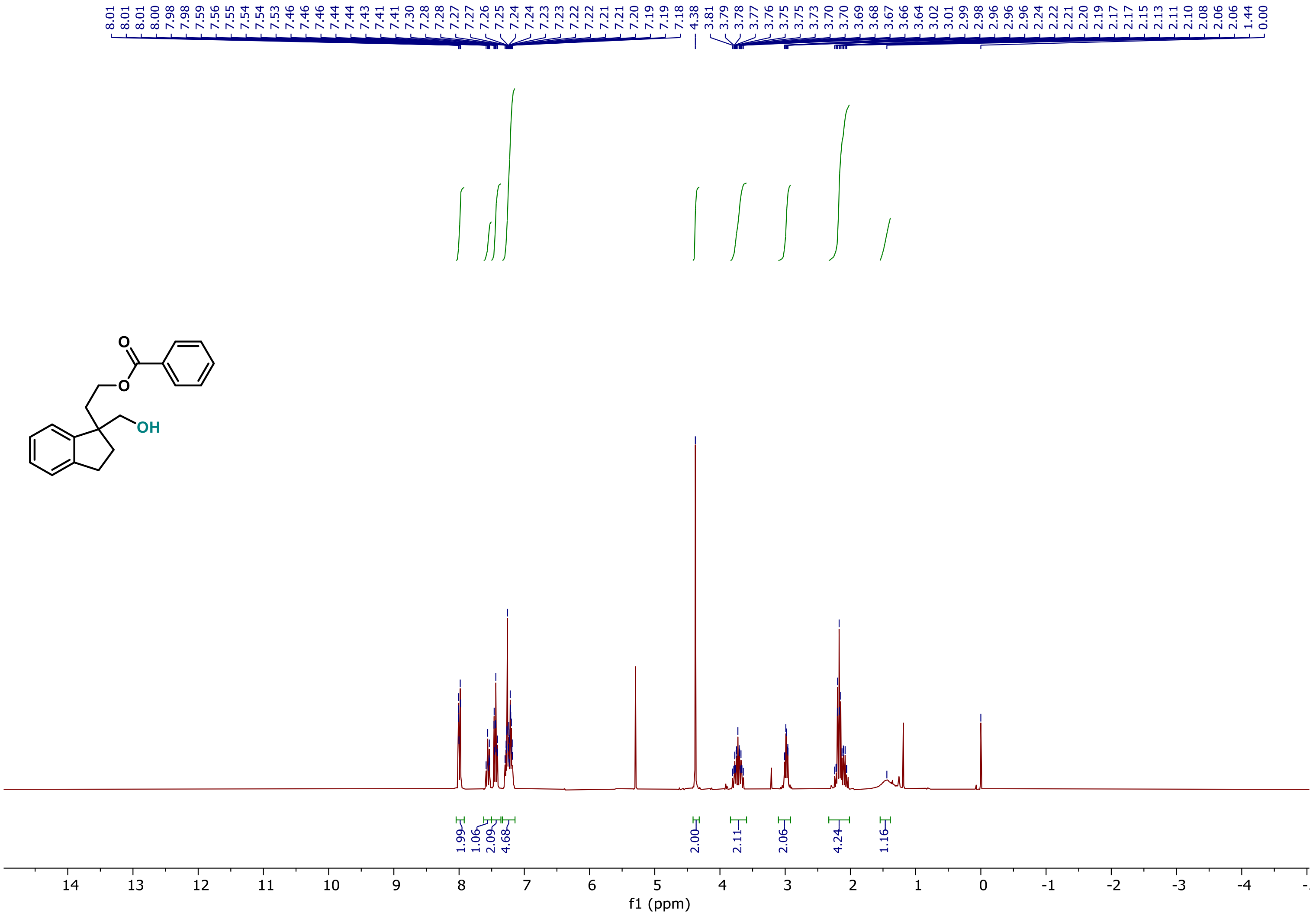

Compound 2t ^{13}C NMR

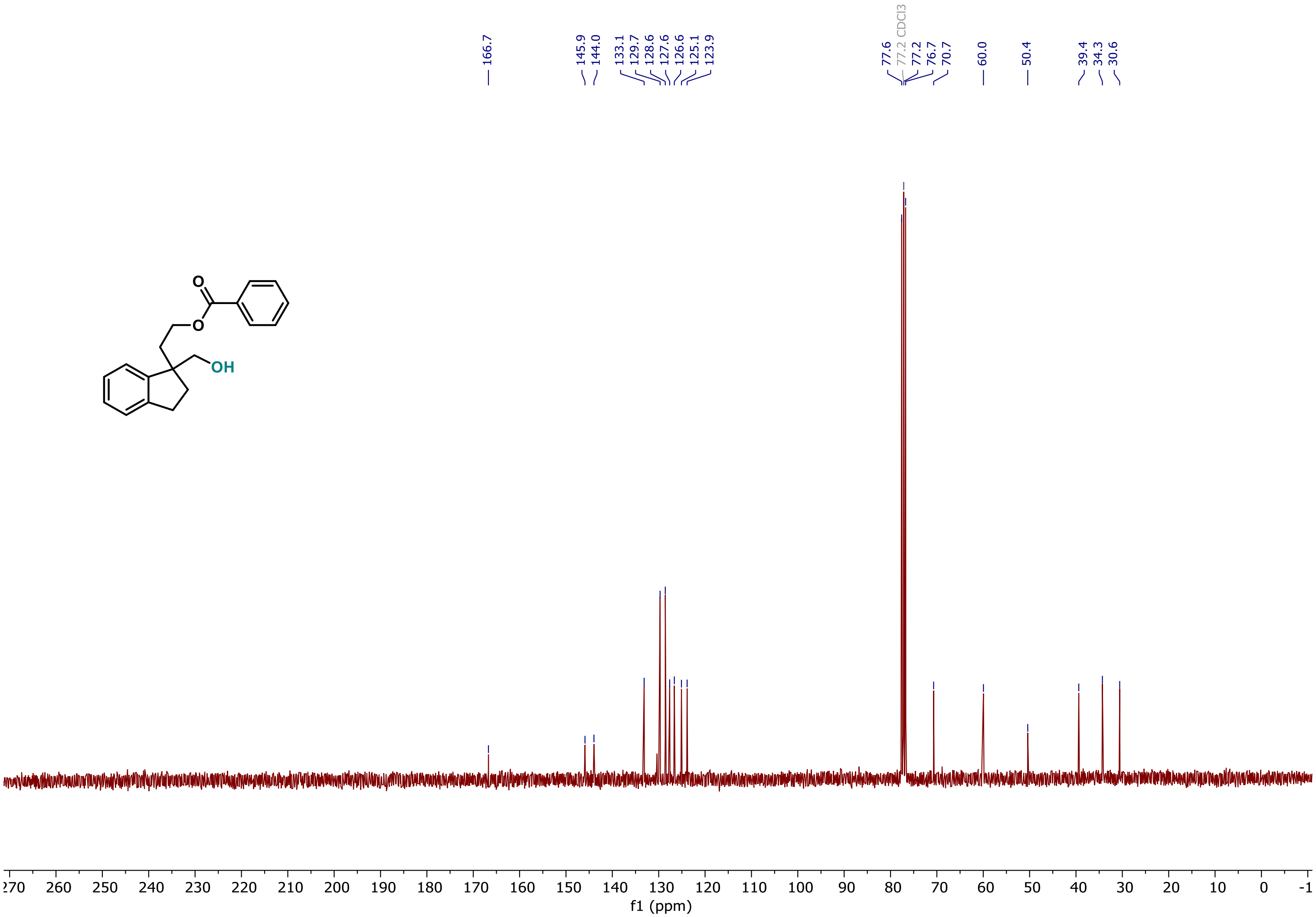

Compound 2u ^{1}H NMR

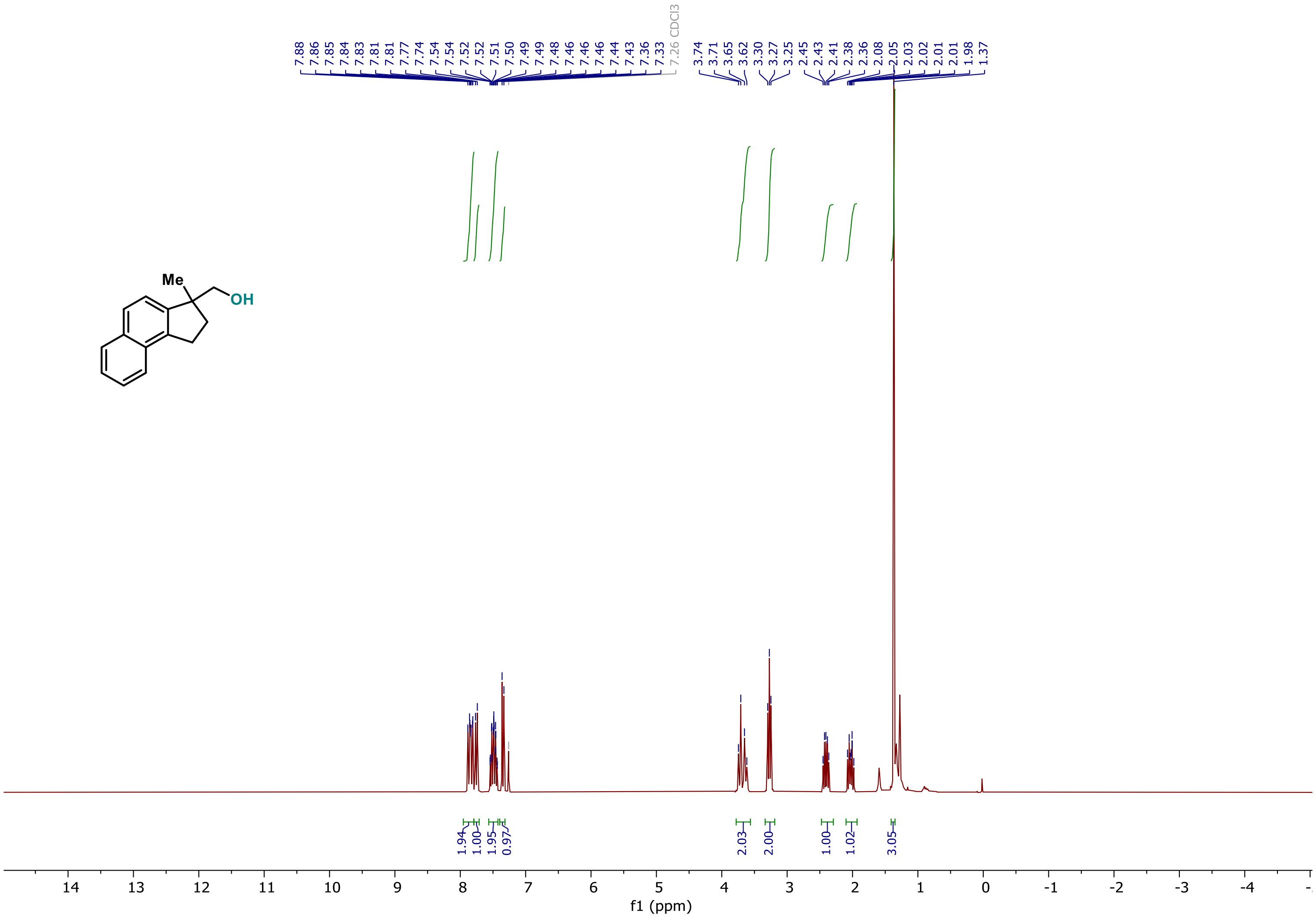

Compound 2u ^{13}C NMR

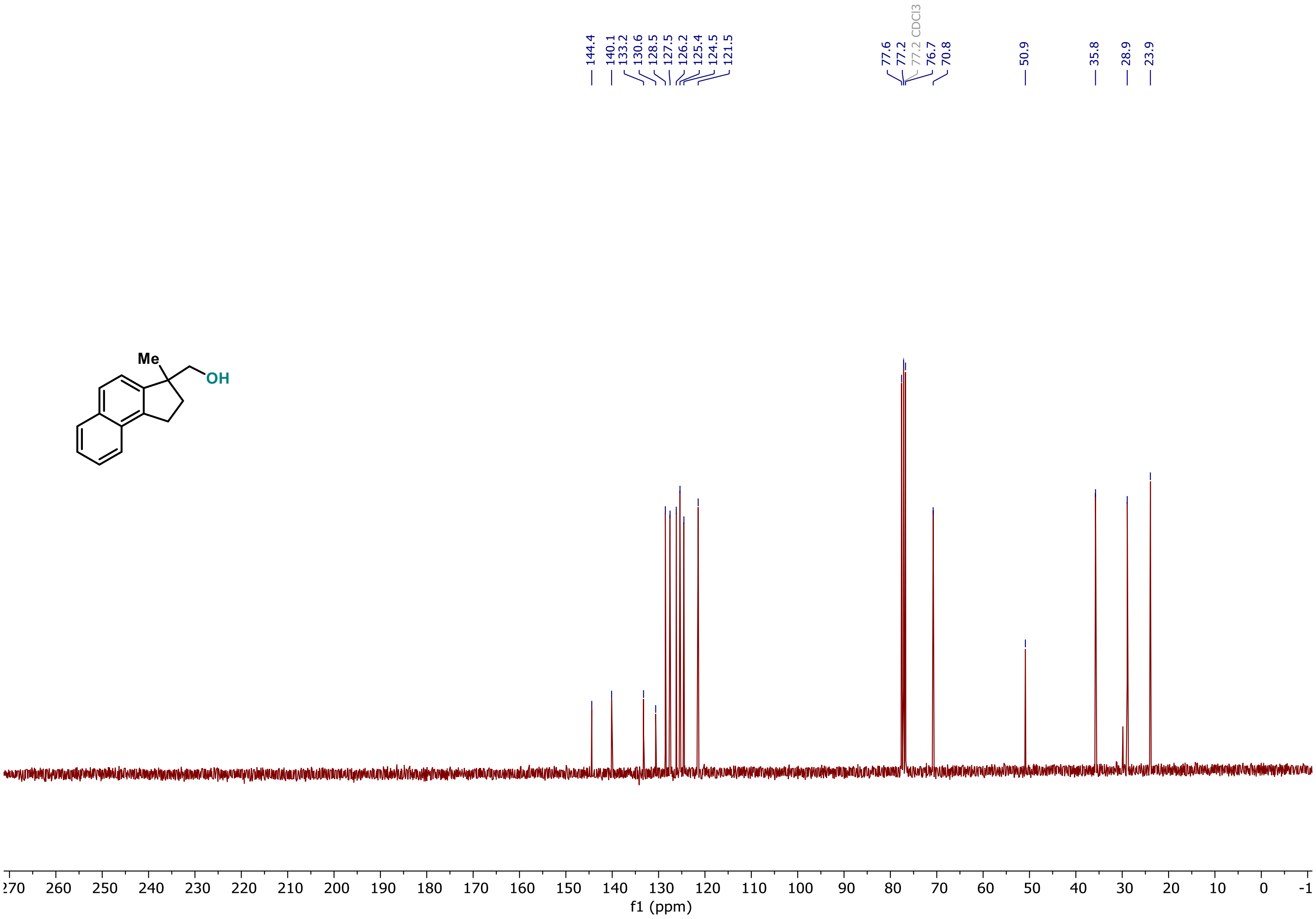

Compound 2v ^{1}H NMR

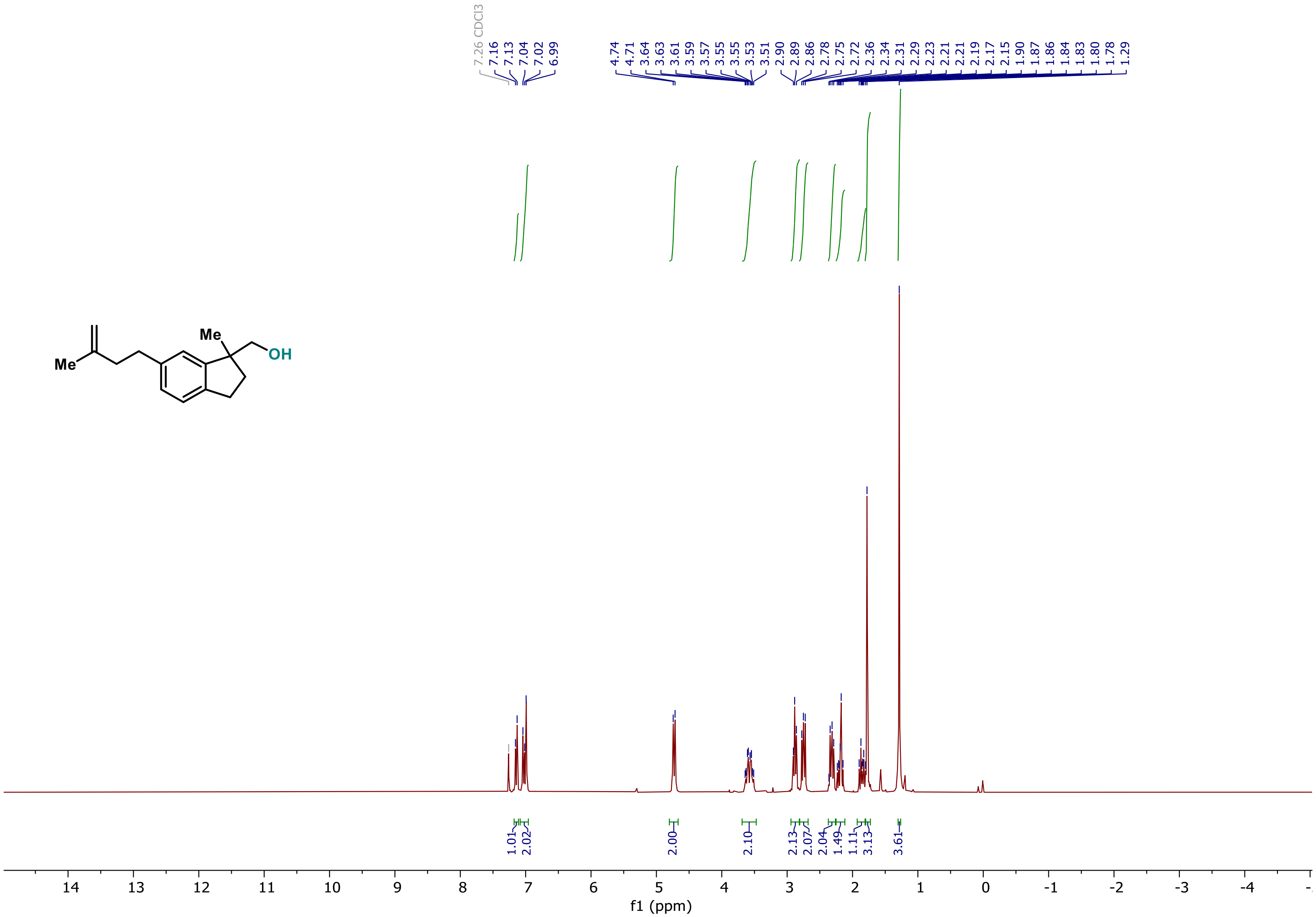

Compound 2v ^{13}C NMR

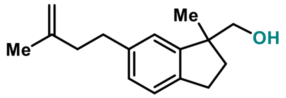

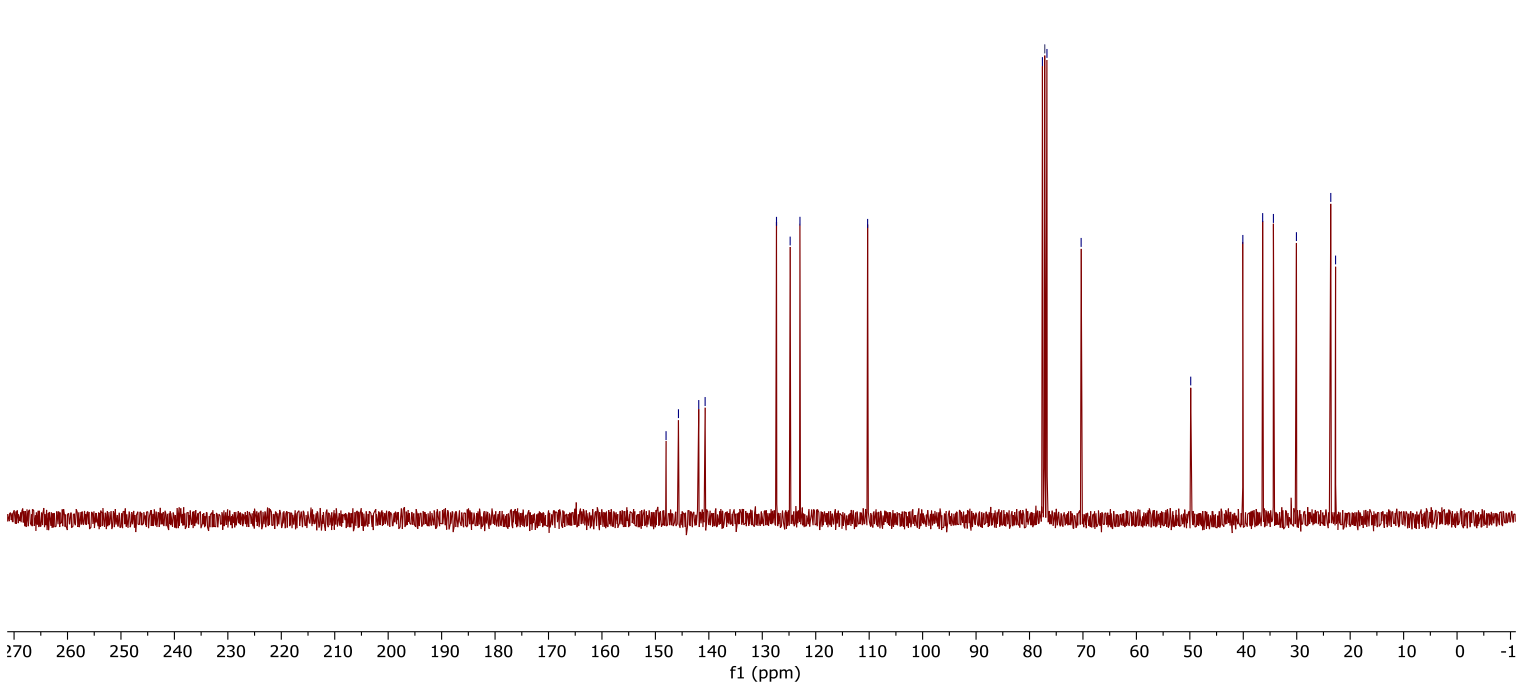

Compound 2w ^{1}H NMR

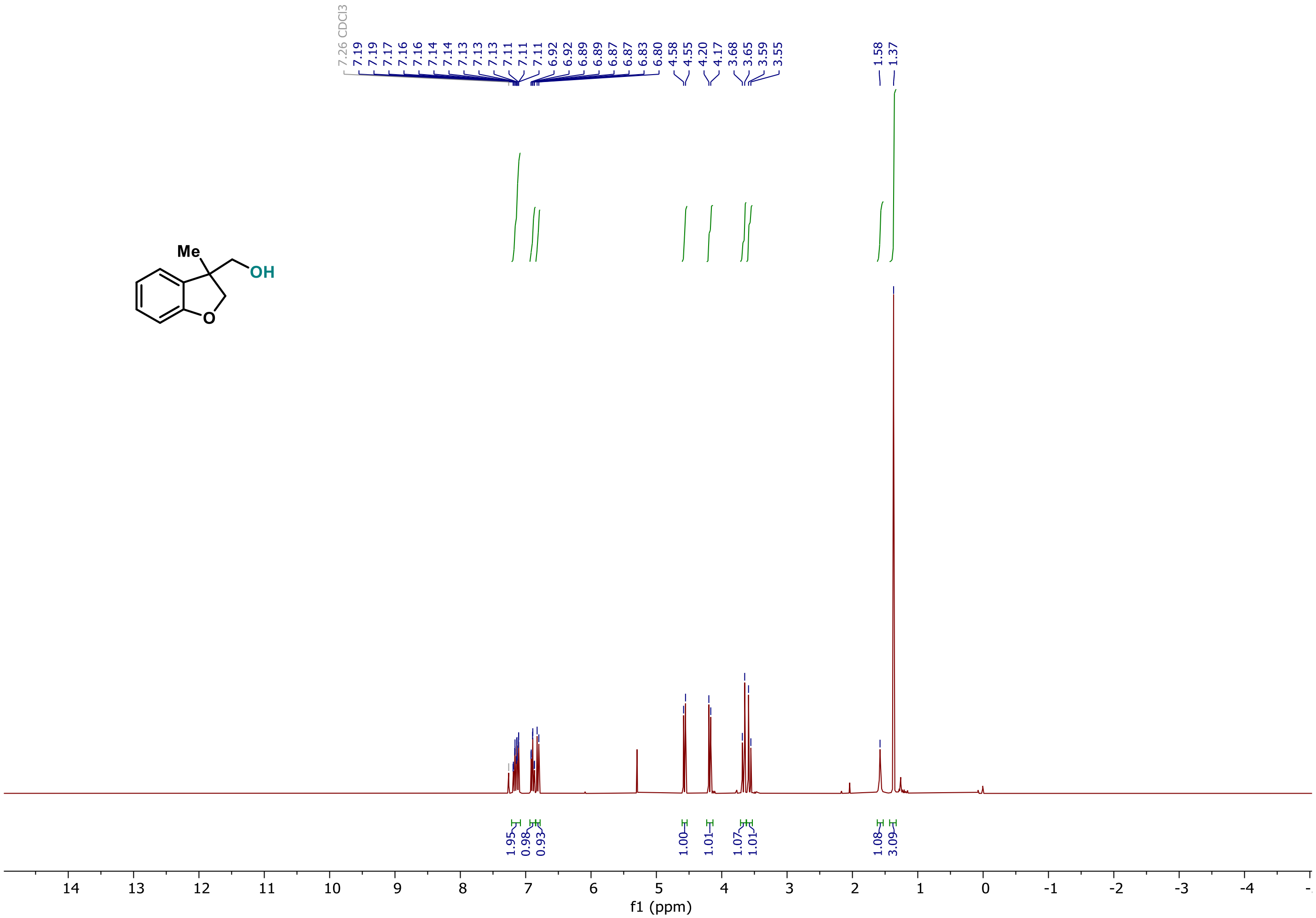

Compound 2w ^{13}C NMR

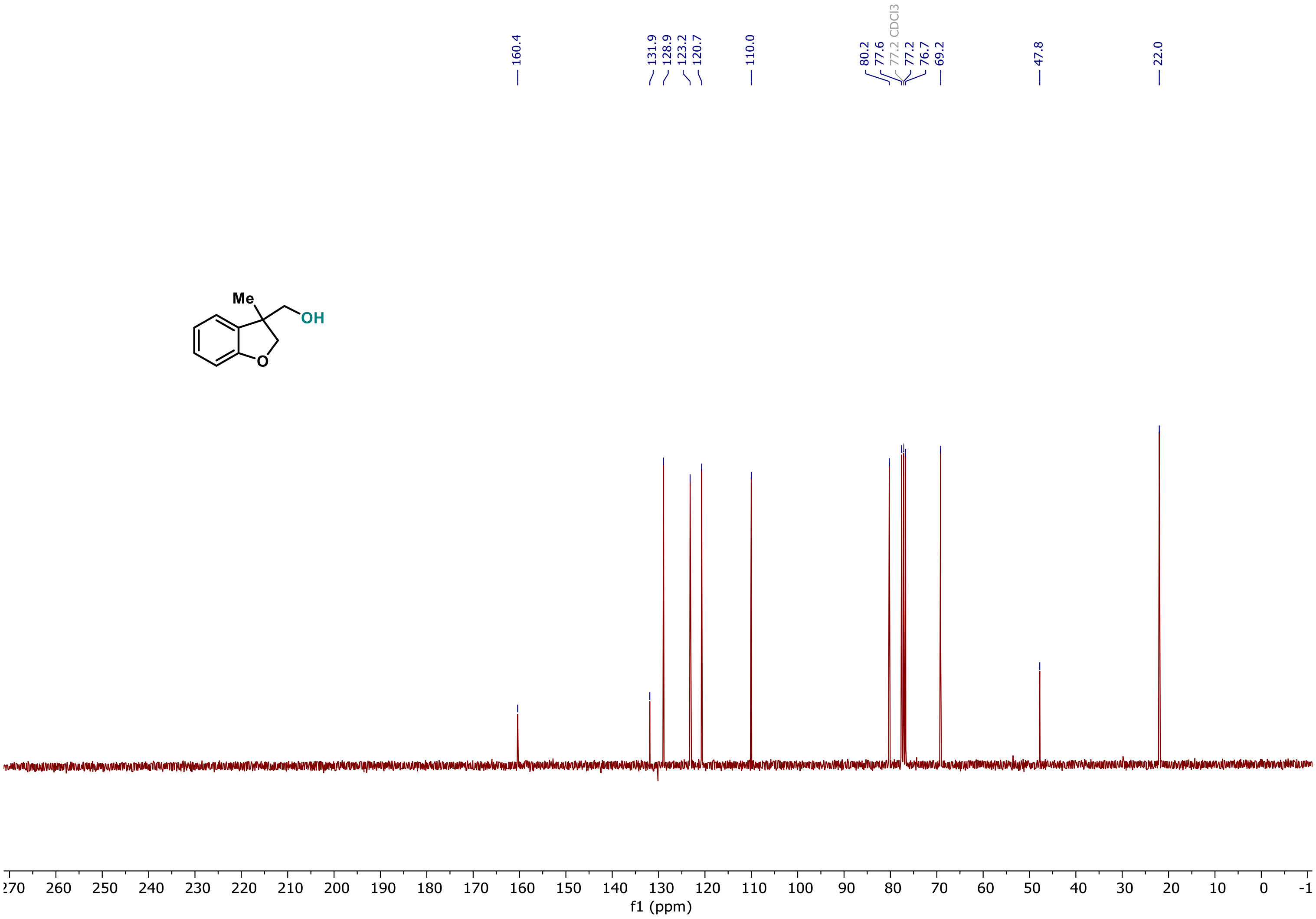

Compound 2x ^{1}H NMR

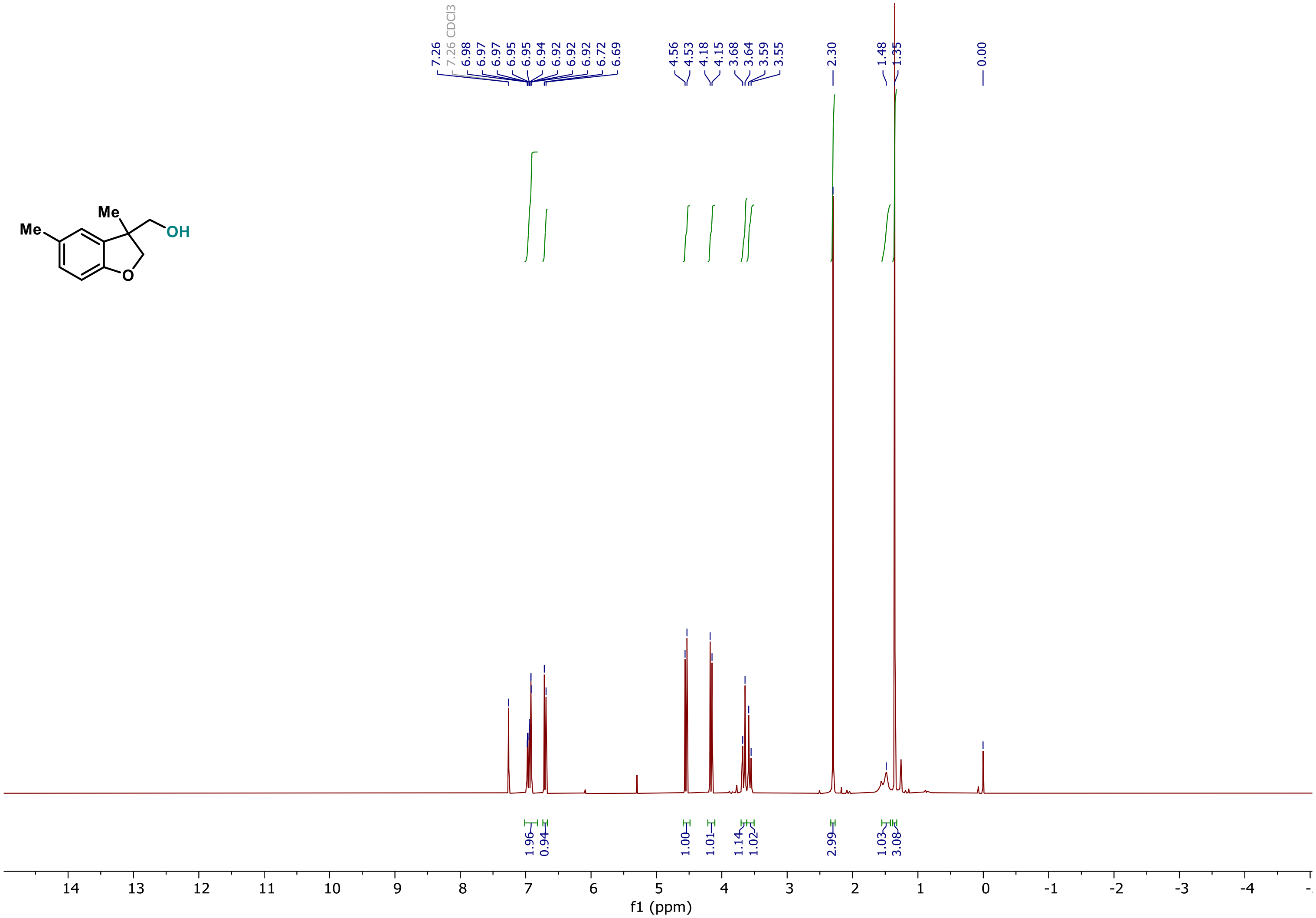

Compound 2x ^{13}C NMR

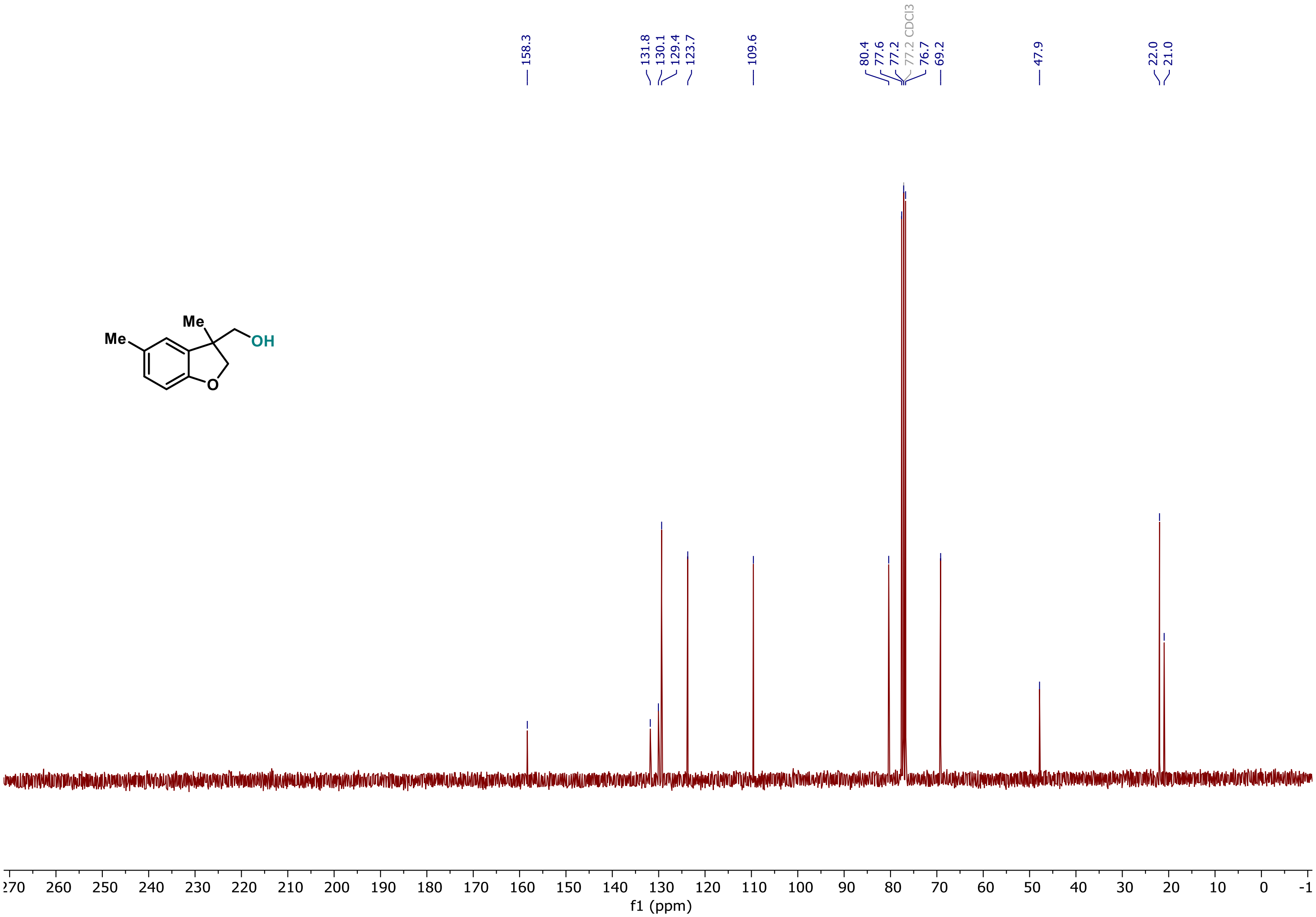

Compound 2aa ¹H NMR

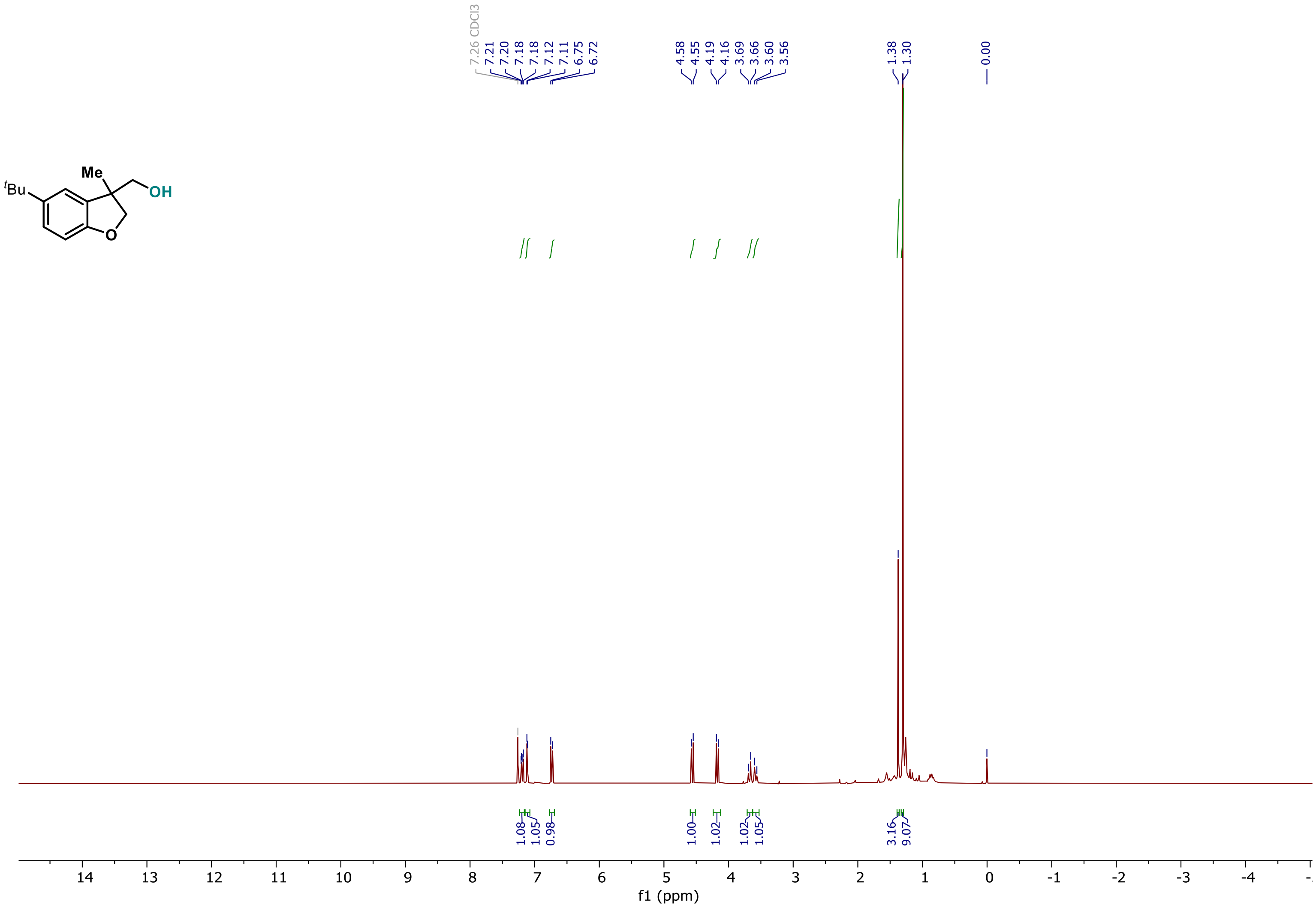

Compound 2aa ^{13}C NMR

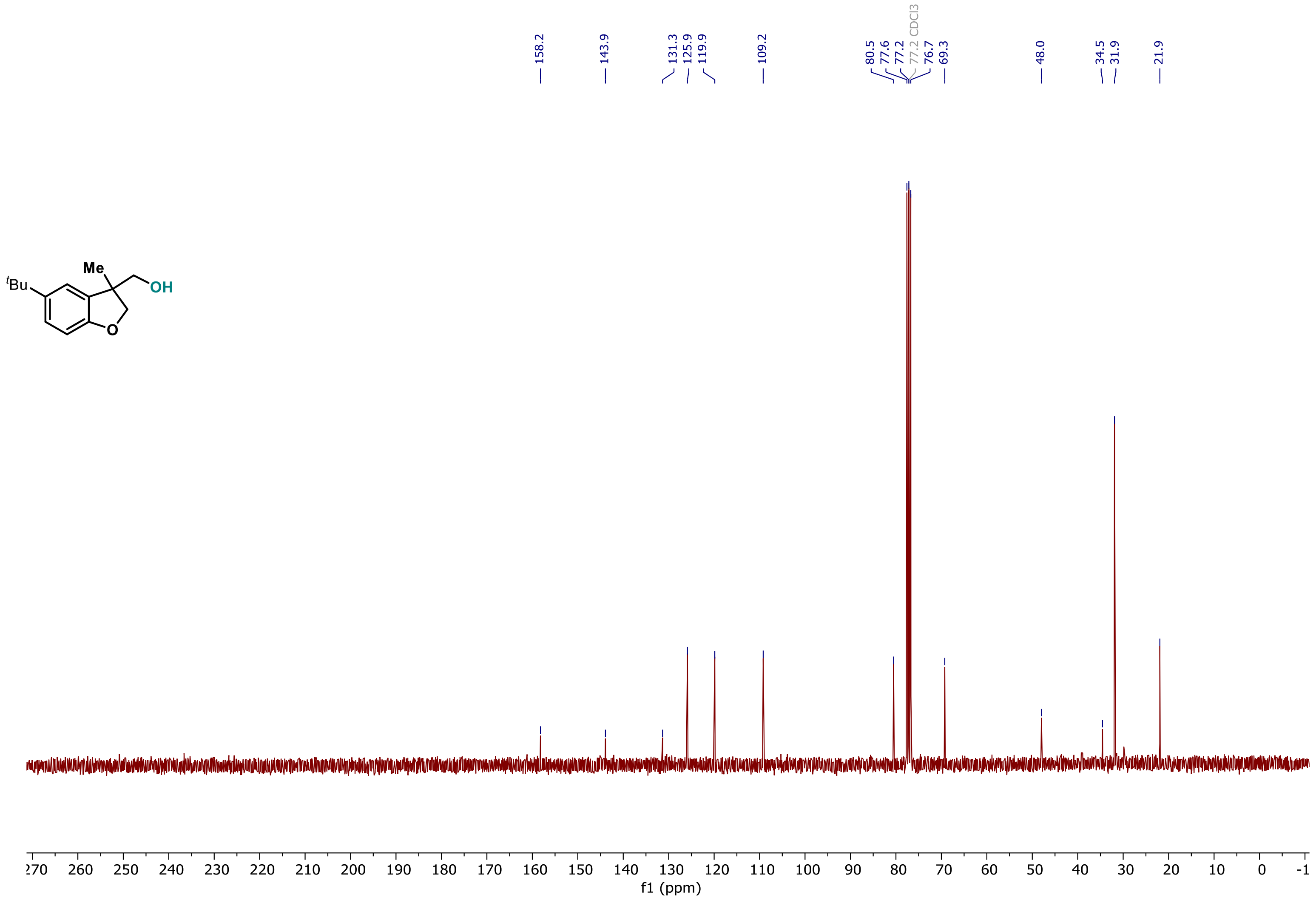

Compound 2ab ^{1}H NMR

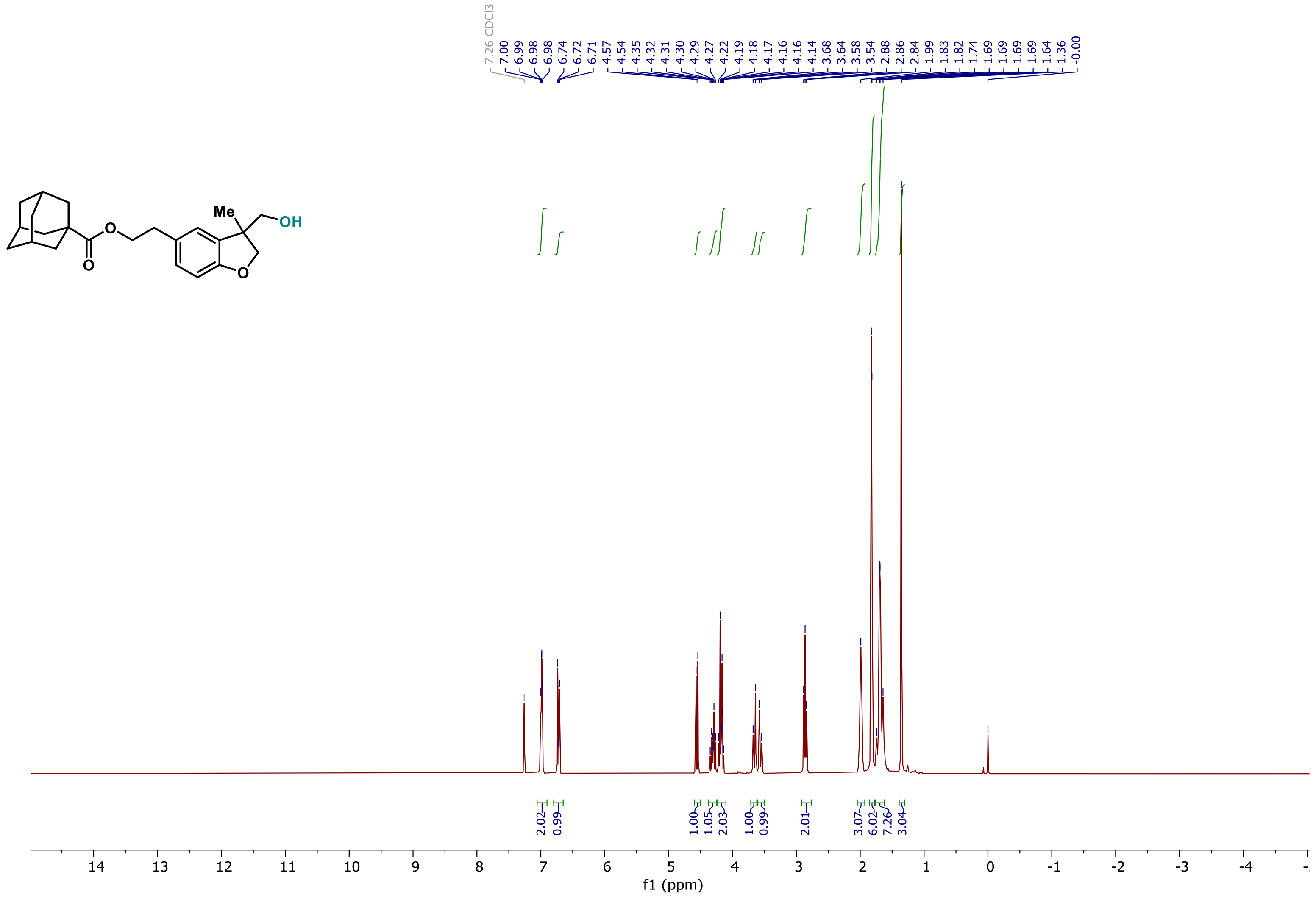

Compound 2ab ^{13}C NMR

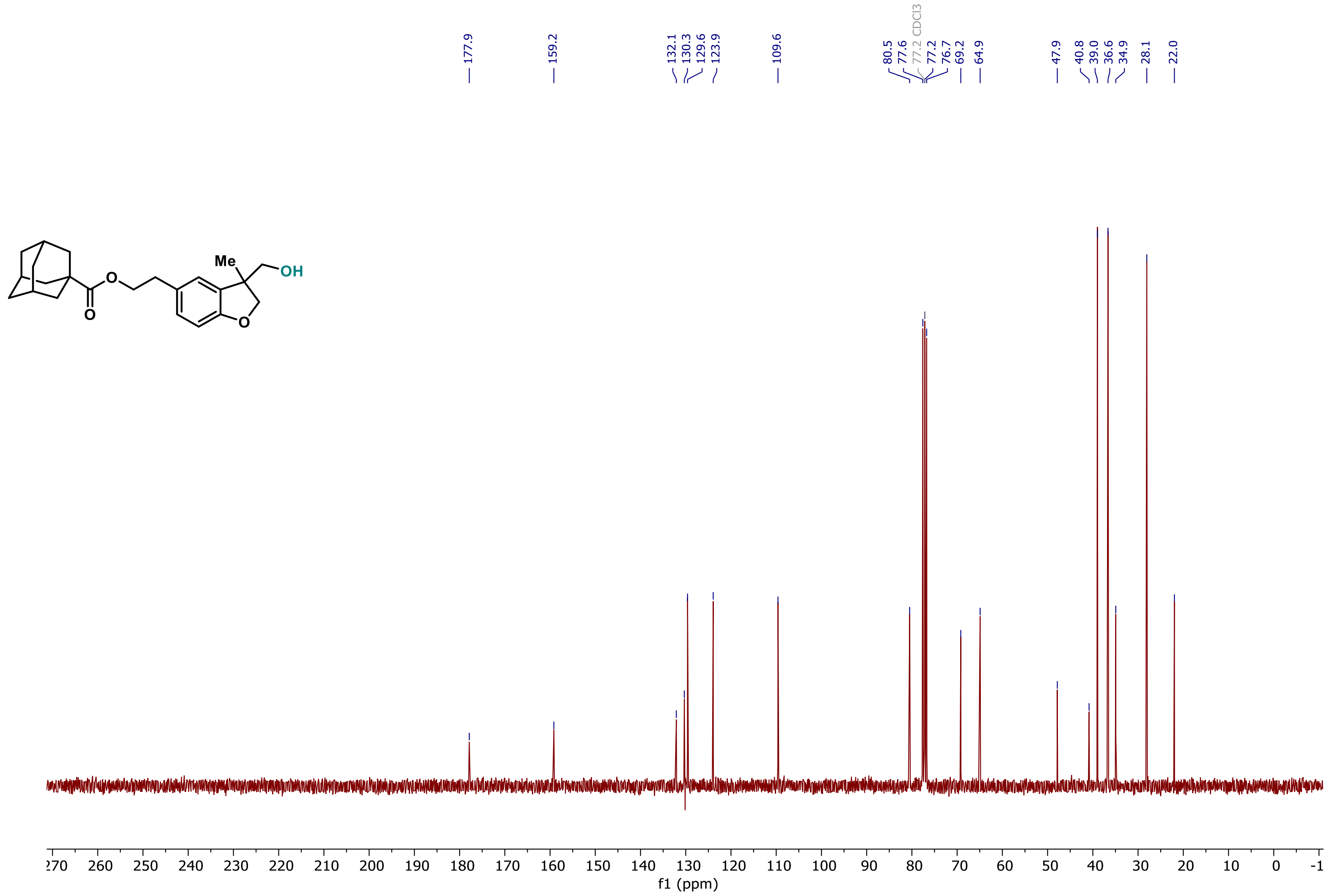

Compound 2ac ^{1}H NMR

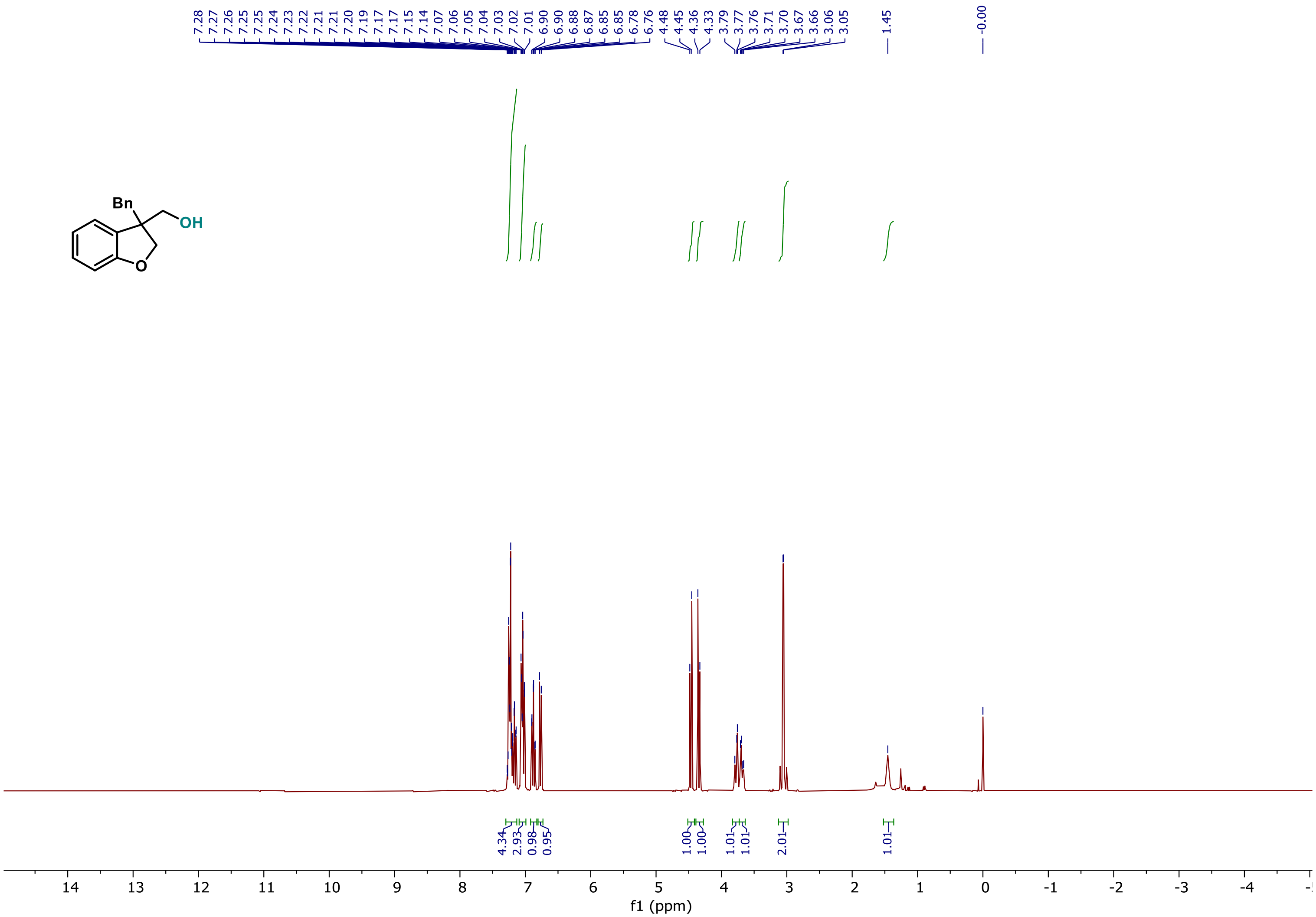

Compound 2ac ^{13}C NMR

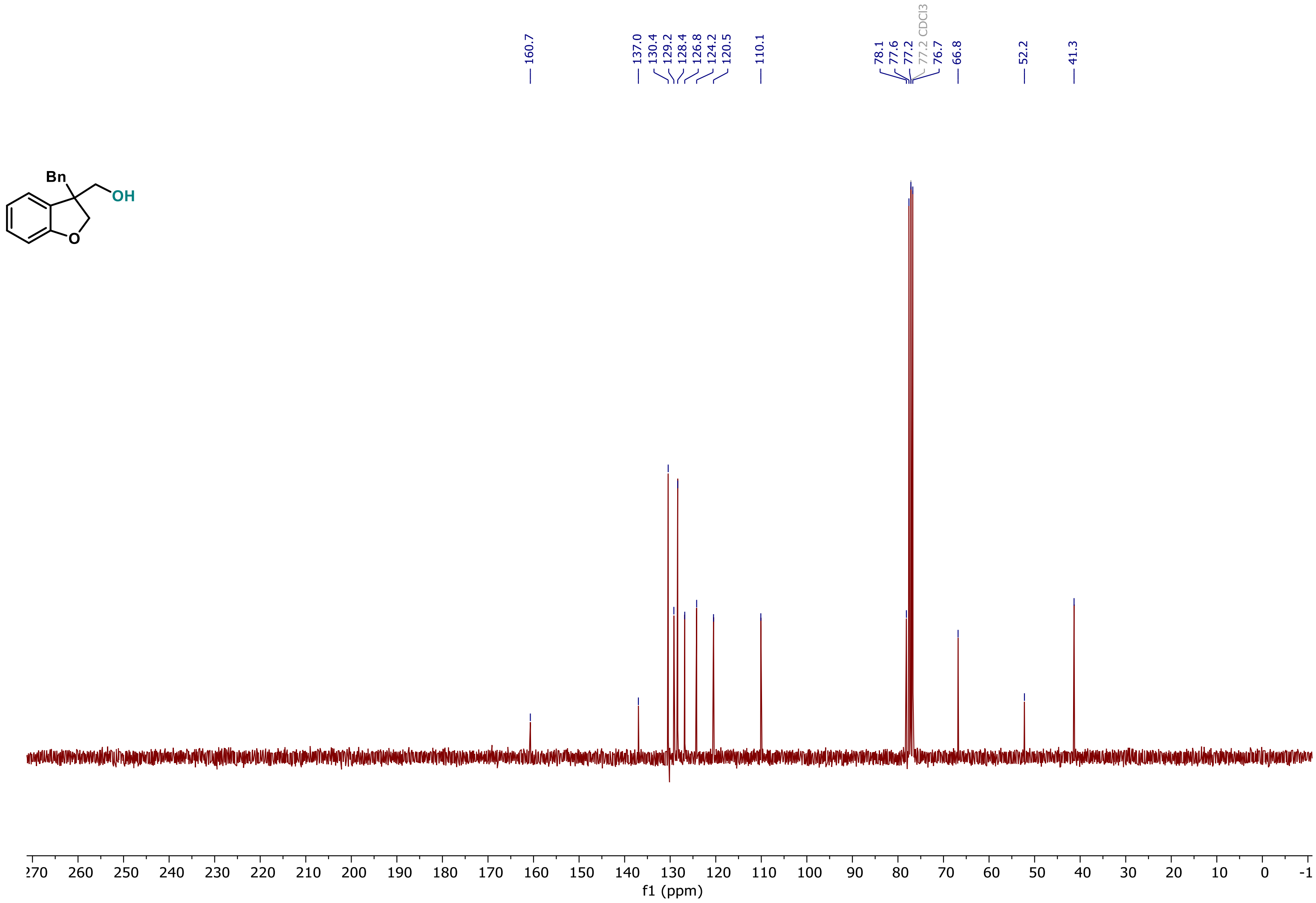

Compound 2ad ^{1}H NMR

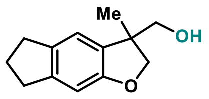

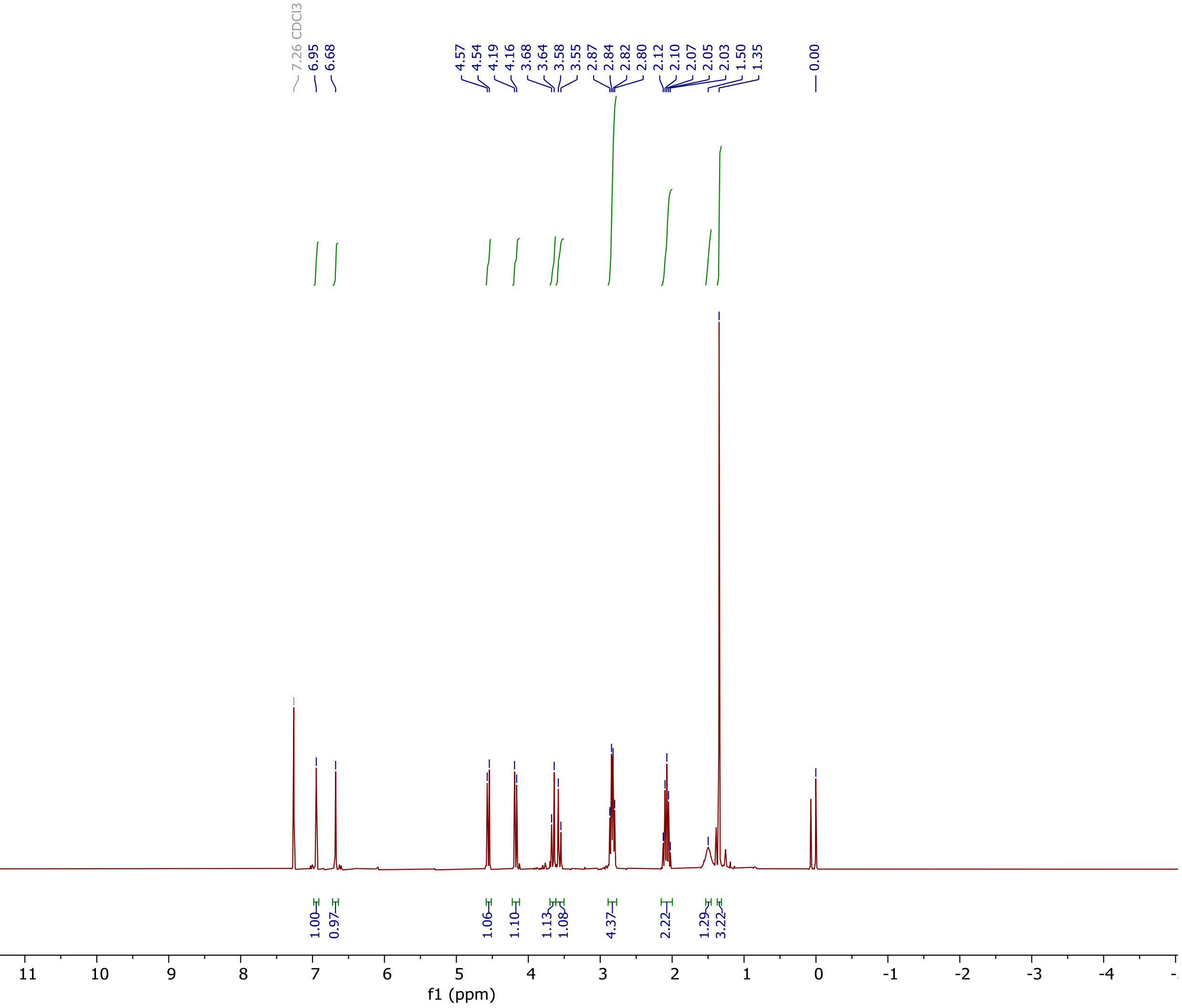

Compound 2ad ^{13}C NMR

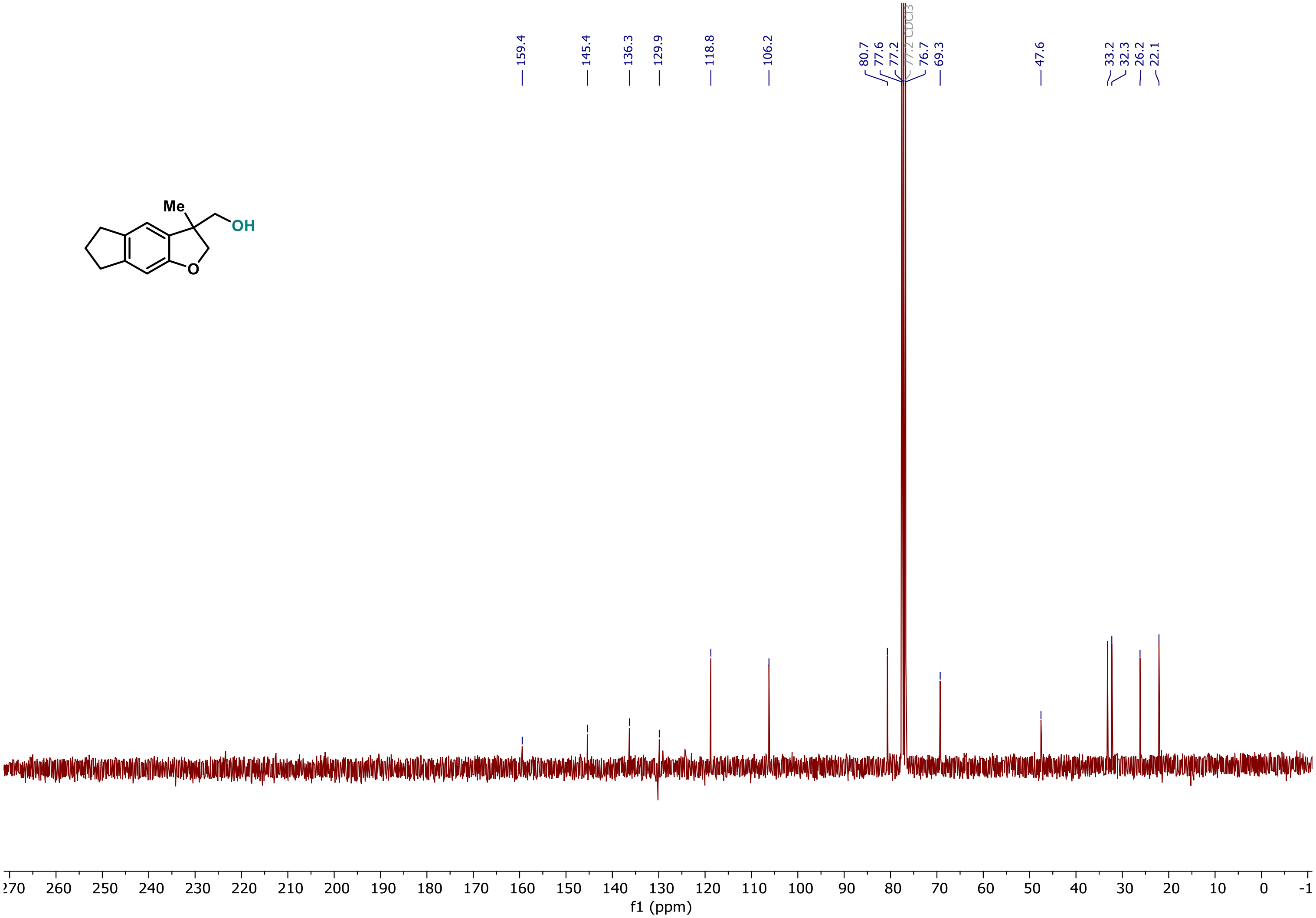

Compound 2ae ^{1}H NMR

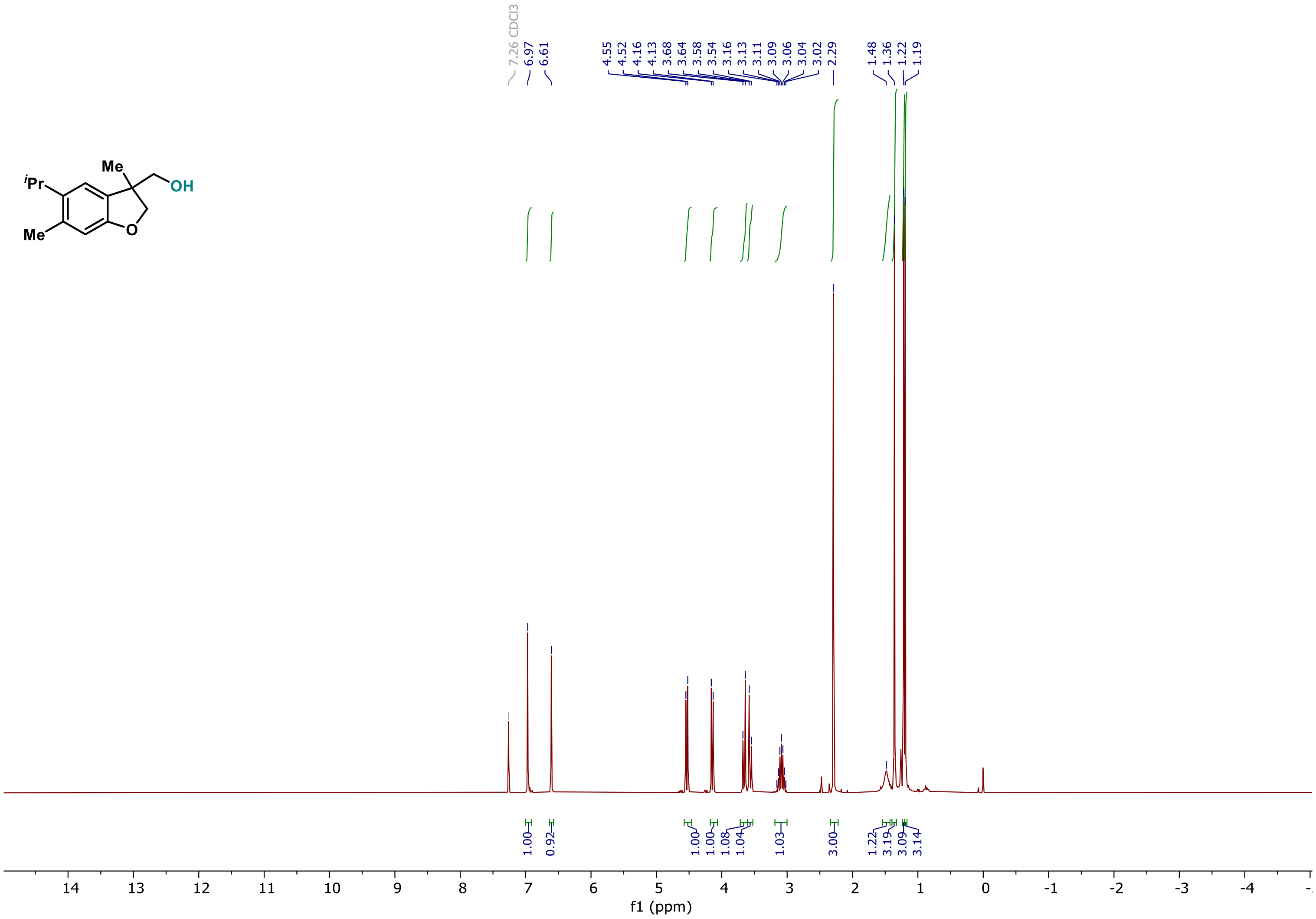

Compound 2ae ^{13}C NMR

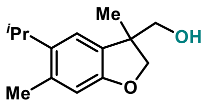

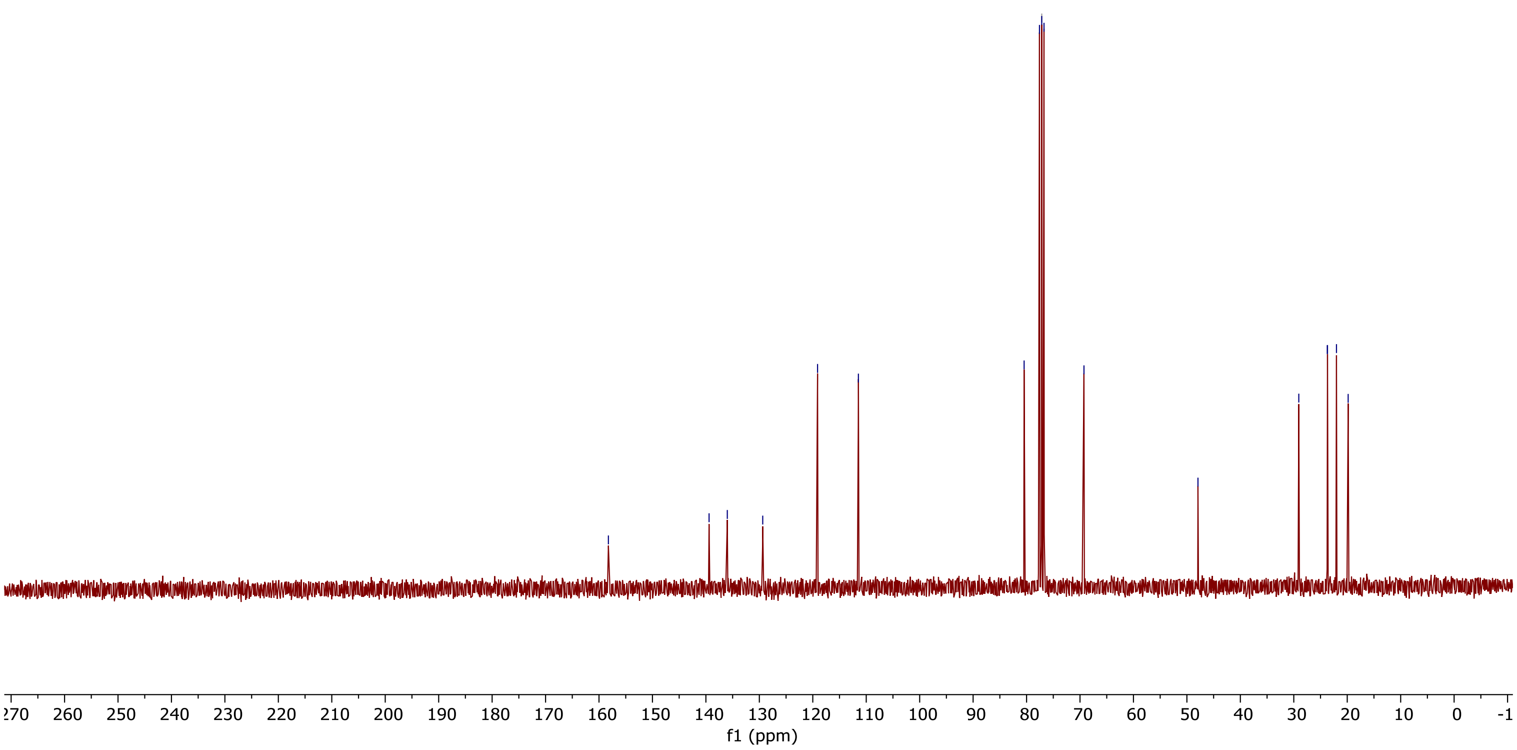

Compound (s)-2w-1 ^{1}H NMR

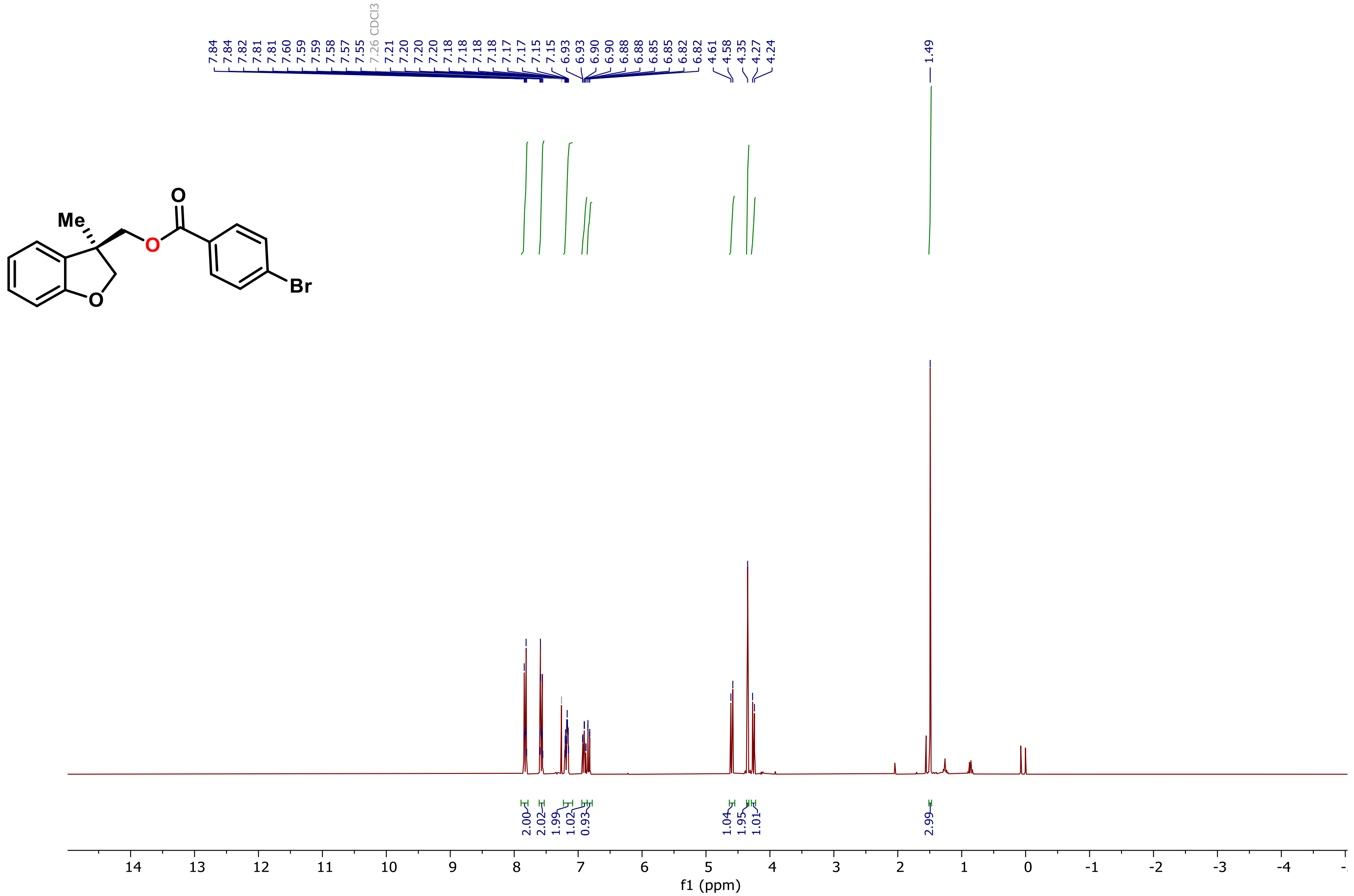

Compound (s)-2w-1 ^{13}C NMR

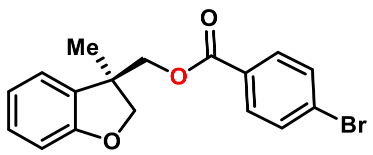

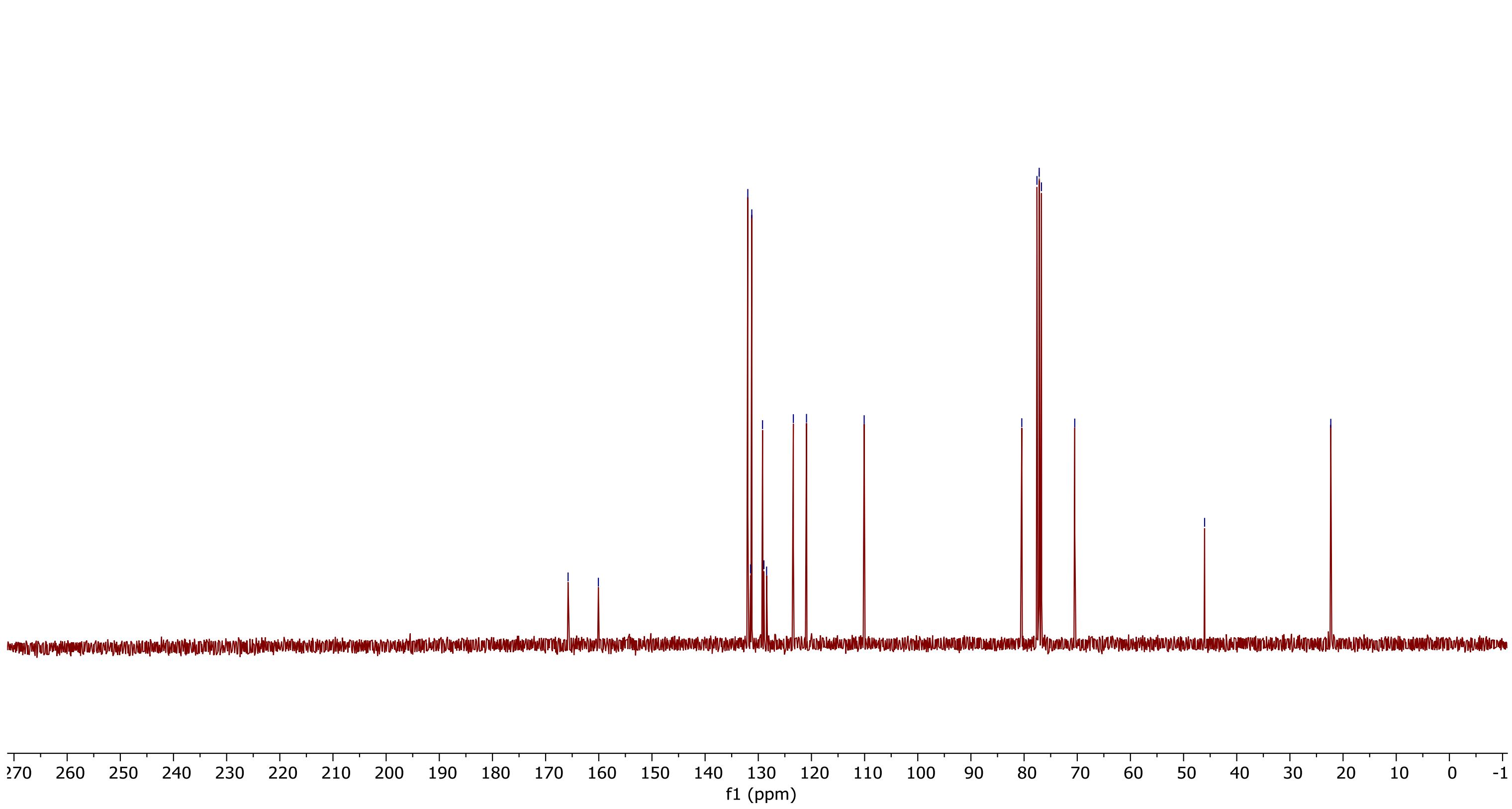

Supplement: Supplementary file 1 — ja2c06227_si_001.pdf [file ja2c06227_si_001.pdf]
